# Pd-catalyzed formal Mizoroki–Heck coupling of unactivated alkyl chlorides

**DOI:** 10.1038/s41467-021-21270-9

**Published:** 2021-02-12

**Authors:** Geun Seok Lee, Daeun Kim, Soon Hyeok Hong

**Affiliations:** Department of Chemistry, Korea Advanced Institute of Science and Technology (KAIST), Daejeon, Republic of Korea

**Keywords:** Homogeneous catalysis, Photocatalysis, Synthetic chemistry methodology

## Abstract

The use of alkyl chlorides in Pd-catalyzed Mizoroki–Heck coupling reactions remains an unsolved problem despite their significant potential for synthetic utility and applicability. The combination of the high thermodynamic barrier of alkyl chloride activation and kinetic propensity of alkylpalladium complexes to undergo undesired β-hydride elimination provides significant challenges. Herein, a variety of alkyl chlorides, even tertiary chlorides, are shown to efficiently participate in Mizoroki–Heck cross-coupling reactions with excellent functional group compatibility under mild reaction conditions via photoinduced Pd catalysis. The reaction is applied to late-stage functionalizations of diverse biologically significant scaffolds and iterative double Mizoroki–Heck annulations, affording high molecular complexity in a single step. Notably, studies on the kinetic isotope effects in combination with density functional theory (DFT)-computations completely exclude the involvement of a previously proposed β-hydride elimination in the catalytic cycle, revealing that the chlorine atom transfer process is the key catalytic turnover step. This distinctive single-electron transfer mediated reaction pathway resolves a longstanding challenge in traditional two-electron based Pd-catalyzed Mizoroki–Heck cross-coupling with alkyl electrophiles, wherein the β-hydride elimination is involved in the formation of both the desired product and undesired by-products.

## Abstract (web summary)

The use of alkyl chlorides in Pd-catalyzed Mizoroki–Heck coupling reactions remains an unsolved problem despite their significant synthetic potential. Here, the authors show that alkyl chlorides can efficiently participate in Mizoroki–Heck cross-coupling reactions with excellent functional group compatibility via photoinduced Pd catalysis.

## Introduction

**Fig. 1:**
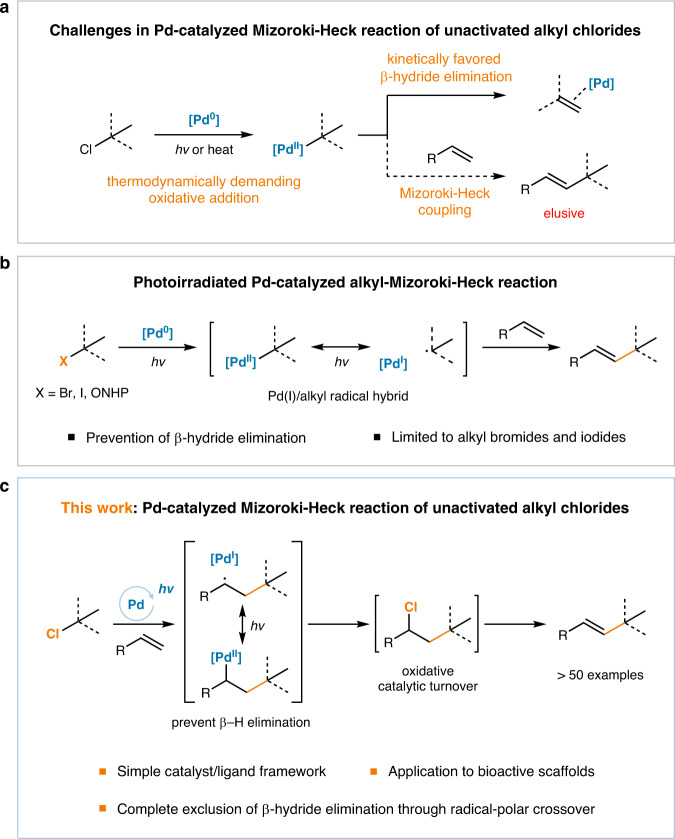
Alkyl Mizoroki–Heck reactions. **a** Challenges in Pd-catalyzed Mizoroki–Heck reaction of unactivated alkyl chlorides. **b** Photoirradiated Pd-catalyzed alkyl Mizoroki–Heck reactions. **c** Pd-catalyzed Mizoroki–Heck reaction of unactivated alkyl chlorides.

The Mizoroki–Heck reaction, discovered in the early 1970s, represents the first methodology to forge C–C bonds via Pd catalysis, enabling highly efficient synthesis of multi-substituted olefin products^1,2^. Owing to its high versatility, the Mizoroki–Heck reaction has been extensively applied in the field of organic chemistry from academic research to industrial processes^3–7^. Similar to other Pd-catalyzed cross-coupling reactions, aryl halides or pseudohalides are mainly utilized as electrophiles in the Mizoroki–Heck reactions, owing to their superior reactivity and robustness in comparison with those of alkyl analogs. The utilization of alkyl electrophiles for the Mizoroki–Heck reaction is significantly more challenging compared with the aryl congeners (Fig. 1a). The rates of Pd oxidative addition to alkyl electrophiles are sluggish and the resulting alkylpalladium species are prone to undesired β-hydride eliminations^8^. Unlike other cross-coupling reactions, because the β-hydride elimination is the key catalyst turnover step in the Mizoroki–Heck reactions, simple suppression of the undesired β-hydride elimination via catalyst or ligand design can reduce catalytic efficiency. Therefore, the control of the productive yet detrimental β-hydride elimination pathways is a significant hurdle that must be overcome^9,10^.

Over the decades, significant progress has been made in the field of Pd-catalyzed alkyl Mizoroki–Heck reactions, notably in single-electron catalysis, which can prevent undesired β-hydride eliminations through the formation of a Pd(I)/alkyl radical hybrid^11–14^. Thermal Pd-catalyzed alkyl Mizoroki–Heck reactions of primary and secondary alkyl bromides and iodides were developed by Fu^15^, Alexanian^16–18^, and Zhou^19^ groups. More recently, photoirradiated Pd catalysis, which harnesses visible light energy to generate Pd(I)/alkyl radical hybrid species via the single-electron oxidative addition of Pd(0) to alkyl halides^20^, has been highlighted as a versatile activation strategy that enables various salient transformations under mild reaction conditions (Fig. 1b)^21,22^. Such transformations include desaturation^23^, reduction^24^, addition to (hetero)arenes^25^, carbonylation^26^, and 1,4-difunctionalization of conjugated dienes^27–29^. By applying this strategy, the Gevorgyan group reported the first visible light-induced Pd-catalyzed Mizoroki–Heck reaction of alkyl bromides and iodides^30^. Subsequently, the Fu group reported the first Pd-catalyzed Mizoroki–Heck coupling of tertiary alkyl bromides, which are highly susceptible to β-hydride elimination^9^, expanding the reactivity to silyl enol ethers and enamides^31^. Similar reactions have also been reported by using other alkyl electrophiles, including N-hydroxyphthalimide esters or tertiary alkyl iodides, by Fu^32^, and Glorius^33^, and Gevorgyan^34^ groups.

In particular, alkyl chlorides are the preferred substrates of choice over bromides and iodides for a number of reasons^35,36^. Most notably, the diversity and the abundant supply of naturally occurring and commercial chemicals^37–39^, high economic preference owing to their low costs^8^, diminished toxicity compared with other alkyl halides^40^, exceptional stability during multistep synthesis, and late-stage functionalization capability^41^ make these compounds attractive synthetic building blocks. However, the stronger C–Cl bond strength, both homolytically and heterolytically, compared with C–Br and C–I bonds has limited the utilization of alkyl chlorides in transition metal catalysis^42,43^. This has resulted in difficulties in both transition metal-mediated oxidative additions and direct reductions via single-electron transfer (SET)^44^.

Regarding the use of alkyl chlorides for Mizoroki–Heck reactions, to overcome the aforementioned challenges, early first-row transition metal catalysts such as a titanocene^45^ or a Co^46,47^ catalyst accompanied by over-stoichiometric Grignard reagents as a reductant were developed with limited applicability. Otherwise, activated alkyl chlorides such as benzyl chlorides^48^ or α-acyl chlorides^49^ were required with Ni or Pd catalytic systems. Regarding unactivated alkyl chlorides, thermal Pd-catalyzed conditions reported by the Fu group were applicable but limited to intramolecular annulations with only four examples that proceed under the assistance of the Thorpe-Ingold effect^15^. The Zhou group reported a protocol for alkyl iodides amenable to primary alkyl chlorides using an in situ Finkelstein reaction with LiI to generate alkyl iodides under harsh reaction conditions at 110 °C^19^. Despite the recent advances, photoexcited Pd catalysis has failed to activate of unactivated alkyl chlorides with no reported example of utilizing alkyl chloride to achieve a C–C bond formation. Therefore, a general and effective method to activate alkyl chlorides for Mizoroki–Heck coupling is a longstanding challenge in Pd-catalyzed cross-coupling chemistry.

Herein, highly efficient Mizoroki–Heck reactions of various unactivated primary, secondary, and even tertiary alkyl chlorides are achieved under mild reaction conditions via visible light-mediated Pd catalysis (Fig. 1c). The reaction exhibits excellent functional group tolerance with wide synthetic applicability, including late-stage transformations of bioactive scaffolds and annulative double Mizoroki–Heck reactions, directly furnishing complex structures. From judiciously designed kinetic experiments and computational studies, the catalytic turnover is shown to occur through an oxidative mass transfer assisted by a chlorine atom, not the previously conjectured β-hydride elimination. This contradicts with the fundamental principle of excited Pd catalysis as the stabilization of alkylpalladium(II) species prevents β-hydride elimination via Pd(I)/alkyl radical hybrid formation. The SET-mediated mechanism provides a rationale for the photoinduced Pd catalysis to overcome the mechanistic dichotomy of two-electron-based Pd-catalyzed alkyl Mizoroki–Heck coupling, where β-hydride elimination is an essential elementary step for product formation and catalytic turnover, but also an undesired side-reaction which must be prevented.

## Results

### Reaction optimization

4-Methoxystyrene **1a** and 1.5 equiv of *tert*-butyl chloride **2a** were chosen as the model substrates for reaction condition optimization (Table 1). Thorough examination of the reaction variables showed that the desired product could be obtained quantitatively using a readily available Pd(0) precatalyst, Pd(PPh_3_)_4_ (entry 1). The commonly used Pd(PPh_3_)_2_Cl_2_ precatalyst, with or without a dual phosphine system involving both monodentate and bidentate phosphine, delivered only minimal product yield, as reported previously (entries 2 and 3)^9^. A quantitative yield could be also obtained with an additional 10 mol% of PPh_3_ ligand using the Pd(PPh_3_)_2_Cl_2_ precatalyst (entry 4). However, when Xantphos (4,5-bis(diphenylphosphino)-9,9-dimethylxanthene) or DPEPhos ((oxydi-2,1-phenylene)bis(diphenylphosphine)) were added to the Pd(0) precatalyst, the reactivity diminished, indicating that the bidentate phosphine-based system failed to activate **2a** (entry 5). Gratifyingly, this reaction condition was suitable for the Mizoroki–Heck coupling of *tert*-butyl bromide (93%) and cyclohexyl iodide (87%; entries 6 and 7), indicating that the reaction is expandable to alkyl bromides and iodides. However, reactions with tertiary alkyl iodides undergo rapid decomposition to furnish intractable mixtures. Simple thermal heating conditions failed to furnish the product, indicating the importance of the photoexcited Pd species in the transformation (entry 8). Finally, control experiments confirmed that Pd catalyst, base, and light are all essential for the reaction (entry 9).

**Table 1:** Optimization of the reaction conditions^a^.


Entry	Variation from standard conditions	Yield^b^ (%)
1	No deviation	>96 (96^c^)
2	Pd(PPh_3_)_2_Cl_2_ instead of Pd(PPh_3_)_4_	4
3	Pd(PPh_3_)_2_Cl_2_/XantPhos (1:2) instead of Pd(PPh_3_)_4_	7
4	Pd(PPh_3_)_2_Cl_2_/PPh_3_ (1:2) instead of Pd(PPh_3_)_4_	>96
5	with the addition of Xantphos or DPEPhos (10 mol %) as ligand	0, 8
6	*t*-BuBr instead of **2a**	93^d^
7	CyI instead of **2a**	87^d,e^
8	At 100 ^o^C, in the absence of light irradiation	0
9	Without Pd(PPh_3_)_4_ or K_2_CO_3_ or light irradiation	0

^a^Reaction conditions: **1a** (0.1 mmol), **2a** (0.15 mmol), Pd(PPh_3_)_4_ (5 mol %), K_2_CO_3_ (0.2 mmol), and DMA [0.1 M] under 40 W blue LED irradiation with fan cooling (30 ± 5 °C).

^b^GC yields using dodecane as an internal standard, unless otherwise noted.

^c^Isolated yield. *E*:*Z* > 20:1.

^d^NMR yields using 1,1,2,2-tetrachloroethane as an internal standard. *E*:*Z* > 20:1.

^e^Yield of the cyclohexyl-substituted product.

### Substrate scope evaluation

After determining the optimized reaction condition, the substrate scope of the reaction starting from alkyl chlorides was explored (Table 2). Simple *tert*-butyl chloride furnished the desired product in excellent yields (96%, **3a**), and scaling up the reaction 10 to 1.0 mmol scale did not affect the reaction outcome (95%). Adamantyl-containing tertiary alkyl chlorides were also compatible with the reaction conditions (73–96%, **3b**–**3d**). Other structurally diverse linear and cyclic alkyl chlorides all reacted smoothly to furnish the desired products (90–96%, **3e**–**3g**). For secondary alkyl chlorides, linear forms, such as isopropyl chloride (92%, **3h**) or isobutyl chloride (>96%, **3i**), produced the product in high yields. In addition, simple carbocyclic chlorides (90–96%, **3j**–**3l**) and 2-indanyl chloride (>96%, **3m**) were proficient coupling partners. Secondary alkyl chlorides nested in bridged cyclic motifs, such as adamantyl (63%, **3n**) and norbornyl (95%, **3o**) groups, were also reactive. Heterocyclic structures, including tetrahydropyran (>96%, **3p**) and piperidine (87%, **3q**) rings, were tolerated under standard reaction conditions. A chlorohydrin, which bears a vicinal hydroxyl group to the chloride, delivered the homoallylic alcohol product without any deterioration in reactivity (87%, **3r**). Finally, a series of primary alkyl chlorides bearing a wide array of functional groups were tested. Generally, primary alkyl chlorides showed slightly diminished yields of approximately 70%. Besides, simple aliphatic, aromatic, and silyl groups bearing alkyl chlorides were suitable substrates (74–83%, **3s**–**3u**). When two equiv of 1,4-dichlorobutane was incorporated, only a minimal amount of difunctionalization product was observed with selective generation of the mono-functionalized product (74%, **3v**). The reaction demonstrated exceptional functional group tolerance to polar functionalities, including ester (67%, **3w**), nitrile (61%, **3x**), ketone (70%, **3y**), and alcohol (69%, **3z**) groups. The tetrahydrofuran ring remained intact during the reaction (77%, **3aa**). Surprisingly, the alkyl chloride bearing a terminal alkyne group was compatible, demonstrating high chemoselectivity in the radical-based Mizoroki–Heck coupling reaction (91%, **3ab**).

**Table 2:**
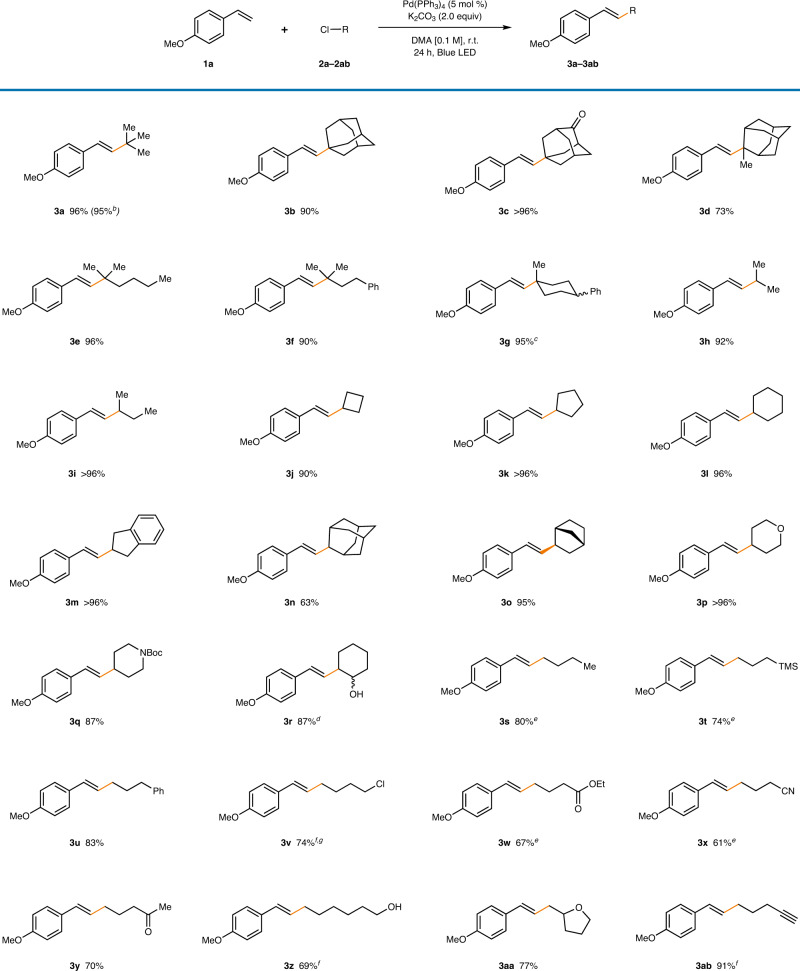
Alkyl chloride substrate scope^a^.

^a^Reaction conditions: **1a** (0.1 mmol), **2a**–**2ab** (0.15 mmol), Pd(PPh_3_)_4_ (5 mol %), K_2_CO_3_ (0.2 mmol), and DMA [0.1 M] under 40 W blue LED irradiation with fan cooling (30 ± 5 °C). All yields are isolated yields. *E*:*Z* > 20:1.

^b^Reaction conducted with 1.0 mmol scale of **1a**.

^c^d.r. = 2:1.

^d^d.r. = 2.7:1.

^e^*E*:*Z* > 10:1.

^f^*E*:*Z* = 7:1.

^g^With 0.3 mmol of 1,5-dichlorobutane (**2v**).

Next, the scope of olefins in the developed reaction was examined (Table 3). A series of electronically neutral alkyl-substituted styrenes underwent smooth functionalization (92–96%, **4a**–**7a**). A sterically hindered mesityl group was also tolerated, demonstrating high tolerance to sterics (58%, **8a**). Halide substituents remained intact, and alkyl chlorides reacted preferentially in the presence of aryl chloride (>96% for **9a**, 64% for **10a**). Mizoroki–Heck coupling of a piperonyl substrate also proceeded with excellent efficiency (>96%, **11a**). Other substituents including phenyl (90%, **12a**), hydroxymethyl (>96%, **13a**), methoxy (86%, **14a**), and acetoxy (96%, **15a**) groups were tolerated. The reaction proceeded chemoselectively with a pinacolboronate ester (64%, **16a**), which can allow for further functionalization. Furthermore, the thiomethyl group did not degrade the reactivity (>96%, **17a**), and vinylnaphthalene was also compatible (64%, **18a**). Apart from simple styrenes, 1,1-diphenylethene (83%, **19a**) and vinylcarbazole (75%, **20a**) could be applied in the developed protocol. Moreover, an α,β-unsaturated amide (54%, **21a**) and vinylamide (68%, **22a**) were reactive, and the utilization of divinylbenzene produced a doubly coupled product in good yields (77%, **23a**). Although a high stereoselectivity was generally observed (>20:1), for substrates bearing extended conjugated systems (**12a**, **18a**, **23a**), *E*/*Z* isomerization occurred after product formation, yielding a mixture of stereoisomers (Supplementary Fig. 1). Notably, in contrast to previous reports^9,32–34^, significantly electron-deficient styrenes, including those bearing a trifluoromethyl, cyano, or ester group, were not compatible with the developed reaction condition (Fig. 3a).

**Table 3:**
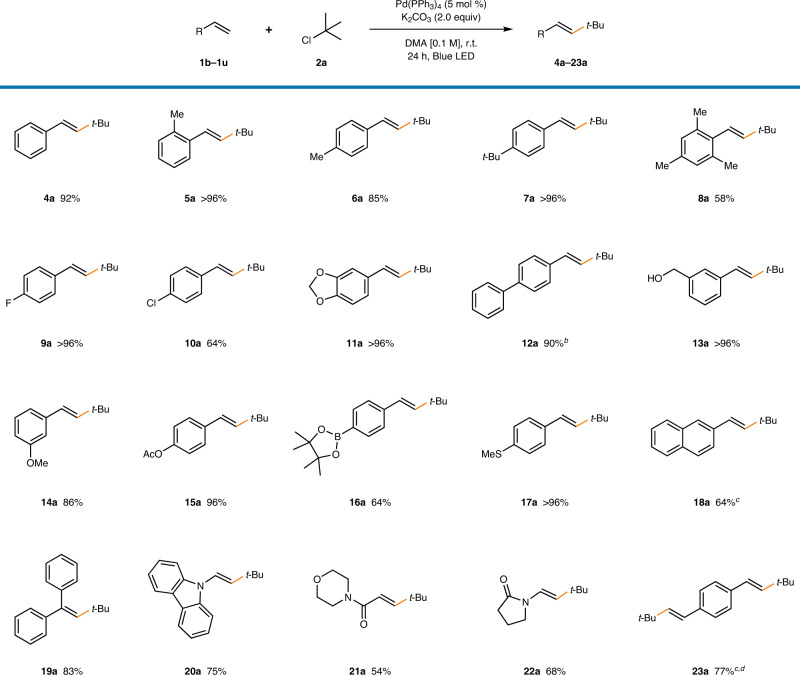
Olefin substrate scope^a^.

^a^Reaction conditions: **1b**–**1z** (0.1 mmol), **2a** (0.15 mmol), Pd(PPh_3_)_4_ (5 mol %), K_2_CO_3_ (0.2 mmol), and DMA [0.1 M] under 40 W blue LED irradiation with fan cooling (30 ± 5 °C). All yields are isolated yields. *E*:*Z* > 20:1.

^b^*E*:*Z* = 1:1.

^c^*E*:*Z* = 1:2.

^d^With 0.3 mmol **2a**.

Taking advantage of the high efficiency, exceptional functional group tolerance, mild reaction conditions, and operative simplicity of the developed reaction, the synthetic applicability of the alkyl chloride Mizoroki–Heck reaction was investigated (Table 4). First, the conditions were applied to complex bioactive molecules for bioconjugation and late-stage functionalization. To offer the highest practicality, the bioactive scaffold-derived compartment was used as the limiting reagent. Complex steroid carbon skeletons residing in either the olefin (vinylated estrone, 70%, **24a**) or alkyl chloride (cholesteryl chloride, 80%, **3ac**) reacted smoothly to furnish the desired products. It should be noted that a mixture of diastereomers was obtained due to the intermediary radical species, even though a single diastereomer was used. Terpenoid-derived menthyl chloride also showed good reactivity (68%, **3ad**). Other bioactive molecules and drug derivatives, including those of δ-tocopherol (66%, **25a**), gemfibrozil (75%, **3ae**), naproxen (70%, **3af**), and vanillin (95%, **26a**) showed good to excellent yields. To our delight, even a biotin derivative afforded styrylated biotin in a high yield, providing opportunities for bioconjugation (81%, **3ag**).

**Table. 4:**
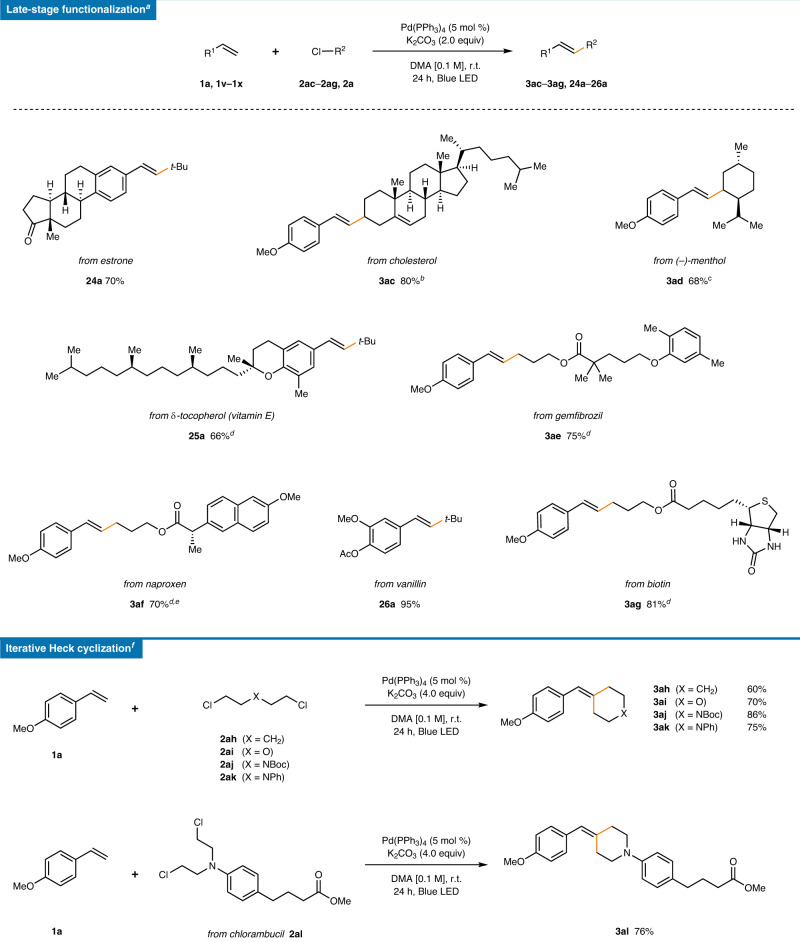
Synthetic applications^a^.

^a^Reaction conditions: **1a** (0.15 mmol), **2ac**–**2ag** (0.1 mmol) or **1aa**–**1ac** (0.1 mmol), **2a** (0.15 mmol), Pd(PPh_3_)_4_ (5 mol %), K_2_CO_3_ (0.2 mmol), and DMA [0.1 M] under 40 W blue LED irradiation with fan cooling (30 ± 5 °C). All yields are isolated yields.

^b^d.r. = 3:4.

^c^d.r. = 2:1.

^d^Pd(PPh_3_)_4_ (10 mol %), 48 h.

^e^*E*:*Z* = 1.3:1.

^f^Reaction conditions: **1a** (0.2 mmol), **2ah**–**2al** (0.1 mmol), Pd(PPh_3_)_4_ (5 mol %), K_2_CO_3_ (0.4 mmol), in DMA [0.1 M] under 40 W blue LED irradiation with fan cooling (30 ± 5 °C). All yields are isolated yields.

Notably, when 1,5-dichloropentane (**2ah**) was subjected to the reaction conditions with 2 equiv of **1a**, an iterative double Mizoroki–Heck coupling proceeded smoothly to furnish an exo-(arylmethylene)cyclohexane framework (60%, **3ah**) without the aid of the Thorpe-Ingold effect. In addition to simple 1,5-dichloropentane, oxygen containing 1,5-dichloride smoothly furnished the tetrahydropyran structure (70%, **3ai**). Similarly, a *tert*-butoxycarbonyl protected amine (86%, **3aj**) or an aniline (75%, **3ak**) analog could also be incorporated, resulting in 4-functionalized piperidine derivatives, which are key moieties in a variety of drugs including cyproheptadine and its analogs. With chlorambucil methyl ester **2al**, an N-arylpiperidine was prepared in a single step (76%, **3al**). However, the use of other olefin substrates except **1a** led to incomplete conversions leading to a mixture of the mono-Mizoroki–Heck product and the cyclized product. To the best of our knowledge, this type of one-step β,β-difunctionalization of styrenes has not been previously reported, which could provide a direct entry to six-membered cyclic compounds with an exocyclic double bond.

### Mechanistic investigations

**Fig. 2:**
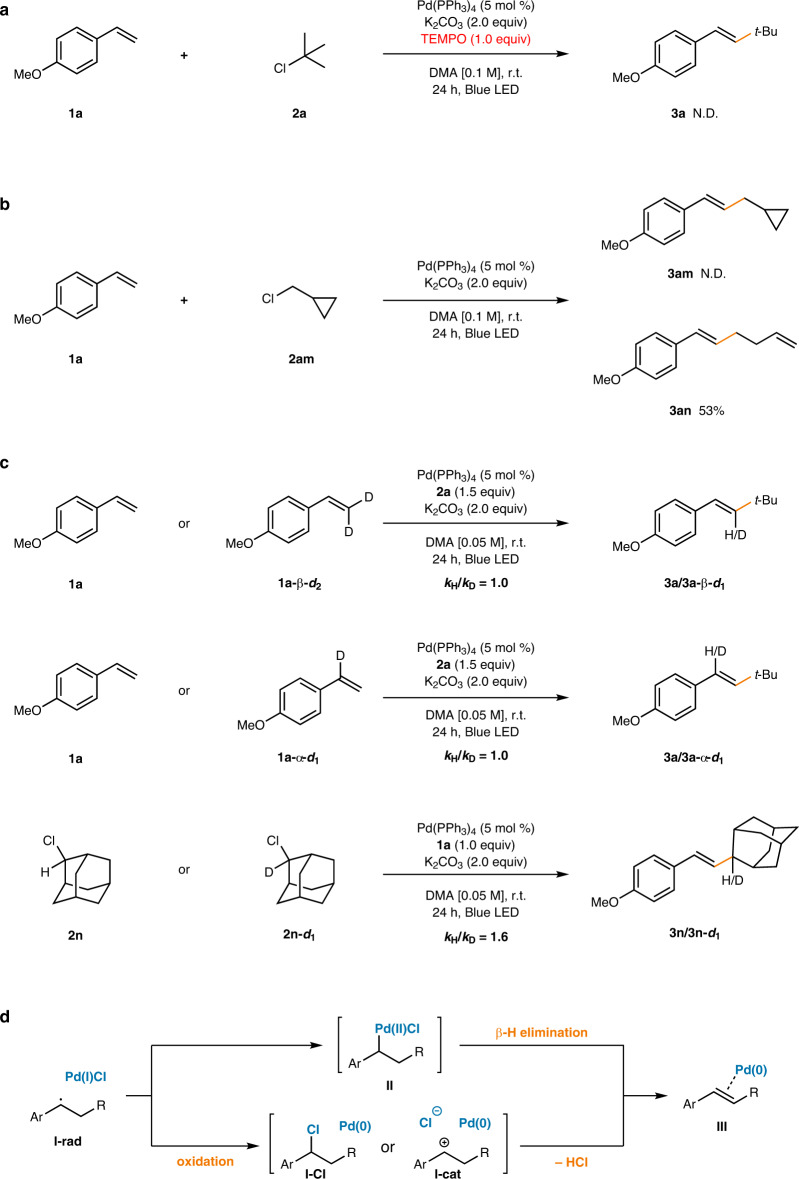
Mechanistic studies. **a** TEMPO as radical scavenger. **b** Radical clock experiment. **c** Kinetic isotope effect measurements. **d** Possible product forming catalyst turnover mechanisms.

To examine the reaction mechanism, it was first attempted to confirm that the reaction indeed proceeds through a radical pathway involving a Pd(I)/alkyl radical hybrid, as previously reported^20,21,31^. First, when a control experiment with a radical scavenger was performed, complete shutdown of reactivity was observed with the addition of 1 equiv of (2,2,6,6-tetramethylpiperidin-1-yl)oxyl (TEMPO) to the standard reaction conditions (Fig. 2a). Moreover, the addition of radical clock substrate **2am** furnished the ring-opening product **3an** exclusively and no direct Mizoroki–Heck product **3am** was detected, indicative of cyclopropylmethyl radical generation (Fig. 2b). Stern–Volmer quenching experiments with **2a** also revealed that photoinduced activation of alkyl chlorides through the excited Pd(0) catalyst was feasible, providing further evidence for a single-electron reduction event (Supplementary Fig. 3). Thorough kinetic studies using both **1a-*d***_**2**_ and **2n-*d***_**1**_ revealed that the initial reduction of the alkyl chloride is the rate-limiting step, as the kinetic isotope effects (KIEs) arising from both the α and β-position of the styrene were 1.0, whereas that from the alkyl chloride was 1.6, indicating a secondary deuterium effect (Supplementary Figs. 4–6, Fig. 2c).

Next, the more controversial step of the reaction mechanism, the product forming catalyst turnover step, was examined (Fig. 2d). After generation of the alkyl radical, it undergoes facile insertion to the olefin substrate, generating a radical adduct, **I-rad**. However, the subsequent formation of the olefin product and concomitant regeneration of a Pd(0) species may occur via a few possible mechanisms (**III**). First, the Pd(II) species **II** formed after radical ligation may undergo direct β-hydride elimination to furnish the product (Fig. 2d, upper pathway), as previously proposed by the Fu group^9^. Another reaction mechanism involving SET or mass transfer from the Pd(I) species to provide either **I-cat** or **I-Cl**, followed by base-assisted elimination is equally plausible (Fig. 2d, lower pathway)^25,50^.

**Fig. 3:**
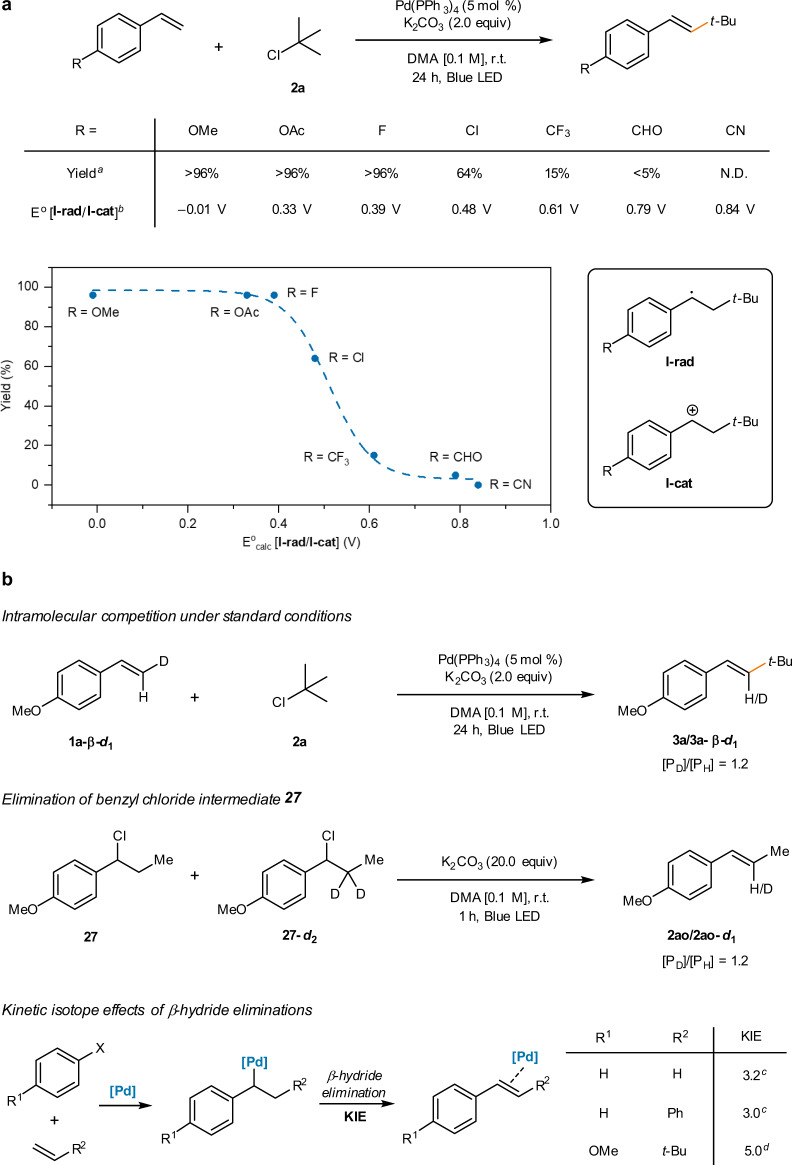
Mechanistic studies on the product forming step. **a** Computed oxidation potentials of I-rad and associated reactivity trends. **b** Kinetic isotope effect measurements/computations. ^a^Yields are isolated yields. ^b^DFT computations were performed at the B3LYP-D3/6-311 + +G**/SDD//B3LYP-D3/6-31 G**/LanL2DZ level of theory in DMA (IEFPCM). The potentials are shown vs N.H.E. ^c^From ref. ^52^. ^d^DFT-computed value.

The Fu group proposed that the β-hydride elimination pathway was operative in the photoirradiated Pd-catalyzed Mizoroki–Heck coupling of alkyl bromides^9^. However, we questioned its operation in the developed reaction because the photoirradiated Pd catalytic system inherently prevents the occurrence of β-hydride elimination^20^. Indeed, time-dependent density functional theory computations on the Pd–alkyl species (as in **II**) showed clear excitation into the Pd–alkyl antibonding orbitals in the blue light energy region (Supplementary Fig. 11). Moreover, contrasting observations regarding the substrate electronics were reported, where electron-poor styrenes failed to furnish the Mizoroki–Heck products under the reaction conditions herein, whereas they smoothly proceeded with alkyl bromides in dual phosphine-based photoinduced Pd catalytic systems^9,32–34^. This indicates that an oxidative process may be involved in the reaction because the reactivity trend is counterintuitive as radical addition to electron-poor olefins should be more facile. This substrate-dependent reactivity was further studied in a quantitative manner via density functional theory (DFT) computation of the oxidation potentials of various **I-rad** species to **I-cat** (Fig. 3a). A decreasing trend in yields was observed as the oxidation potential of **I-rad** increased. Notably, a clear cutoff potential region was identified between R = F and R = CF_3_, as commonly expected for an oxidative transformation^51,52^. The results suggest that an oxidation-based mechanism is likely operative in the product formation pathway.

Next, in order to independently investigate the catalyst turnover step, a number of kinetic studies using deuterated substrates were performed to observe and compare the KIE values in the proposed scenarios (Fig. 3b). As the identified rate-determining step, initial reduction of the alkyl chloride (Fig. 2b), is located prior to the elementary steps of interest, competition-based kinetic experiments and DFT computations were carefully designed to unambiguously determine the KIEs of each step^53^. First, an intramolecular competition experiment, analogous to that reported by the Fu group^9^, was conducted using **1a-β-*d***_**1**_ (Supplementary Fig. 7, Fig. 3b, first line). A KIE value of 1.2 was observed, similar to that obtained by the Fu group (1.4). The Fu group interpreted the result by comparison to a general E2 reaction, isopropyl bromide elimination with an ethoxide base (KIE = 6.7)^54^, and excluded a bromine atom transfer pathway. They assumed that the involvement of the benzylic brominated product followed by elimination would have a large KIE, similar to the E2 reaction. Based on the results, they proposed a β-hydride elimination pathway for their photoexcited Pd-catalyzed Mizoroki–Heck reaction with alkyl bromides.

However, the elimination reaction of benzyl halides would be significantly different from simple aliphatic alkyl halides. To the best of our knowledge, the KIEs of the elimination reaction of β-alkyl secondary benzyl halides remain largely unstudied. Alternatively, the Balachandran group demonstrated that the elimination reactions of cumyl chloride^55^ or 1-chloro-1,2-diarylethanes^56,57^ proceeded via a unimolecular E1 mechanism without rate dependence on the base concentration and with unimolecular rate dependence on the concentration of the alkyl chlorides. Hence, we confirmed the KIE value of the elimination reaction of a secondary benzyl chloride via competition kinetic experiments with independently prepared **27** and **27-*d***_**2**_ (Supplementary Fig. 8, Fig. 3b, second line). A secondary KIE value of 1.2 was obtained, likely originating from the carbon hybridization change during the chloride detachment, supporting an E1 pathway as in previous reports^55–57^. The observed KIE values of 1.2 (Fig. 3b, first line) is not consistent with previously reported KIE values observed for the two-electron based Pd-catalyzed Mizoroki–Heck coupling reactions with ethene (KIE = 3.2; Fig. 3b, third line, R^1^ = R^2^ = H), or with styrene (KIE = 3.0; R^1^ = H, R^2^ = Ph) where β-hydride elimination occurs via cleavage of the homobenzylic C–H bond from a benzylic Pd species^58^. DFT computations of the KIE value on the exact substrate (R^1^ = OMe, R^2^ = *t*-Bu) were performed for the β-hydride elimination pathway, yielding a primary KIE of 5.0 (Supplementary Table 2). Overall, these kinetic experiments, along with literature and computational data, strongly oppose the β-hydride elimination mechanism in the reaction, implying that an oxidative mechanism is more plausible. Notably, a benzyl chloride intermediate is smoothly converted to the desired elimination product under our standard reaction conditions, which is in clear contrast to previous reports of Pd-catalyzed Mizoroki–Heck reactions where the addition of halide transfer intermediates to the standard reaction conditions led to catalytic cycle poisoning^9^ or only low elimination yields^19^ (Supplementary Fig. 9). Moreover, considering that the observed KIE values from the two experiments in Fig. 3b (first and second lines) are almost identical, it is likely that the oxidation occurs via the mass transfer of a Cl atom to produce **I-Cl** as a key reaction intermediate. The computed redox potentials of Pd–Cl species are not sufficient to directly oxidize **I-rad** (Fig. 3a, *E*^o^_calc_[Pd(I)/Pd(0)] = −0.85 V, *E*^o^_calc_[Pd(II)/Pd(I)] = −0.83 V vs. N.H.E., Supplementary Fig. 10), further indicating that the direct oxidation pathway from **I-rad** to **I-cat** is unlikely. Moreover, when *tert*-butyl *N*-(acyloxy)phthalimide, a competent electrophile in the previously reported dual phosphine-based photoexcited Pd-catalyzed Heck reaction^32^, was employed, the reactivity was largely diminished (13% yield) under the standard conditions as a phthalimide group transfer is implausible. The reactivity was moderately recovered (22% yield) with the addition of lithium chloride, indirectly suggesting the role of chloride in the reaction mechanism (Supplementary Table 1). An analogous reaction mechanism has been recently reported in the identical Pd system to perform alkylation of unactivated arenes, which involves a bromine atom transfer as the key turnover process^59^. Unfortunately, even after numerous attempts, capturing the chlorine atom transfer transition state through DFT modeling was unsuccessful.

**Fig. 4:**
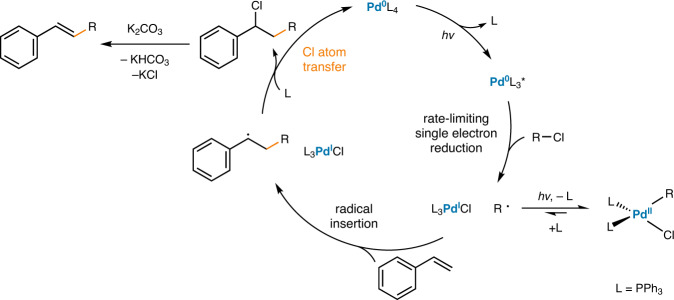
Proposed reaction mechanism. Reaction mechanism involving chlorine atom transfer as the key turnover step.

Combining the above mechanistic investigations, a plausible catalytic cycle was proposed (Fig. 4). After alkyl chloride reduction from the excited Pd(0) species to yield alkyl radicals, this radical is selectively inserted to the β-position of the styrene, yielding a Pd(I)/benzylic radical hybrid species. As β-hydride elimination is significantly prevented by irradiation, single-electron oxidation mediated by a chlorine atom occurs to furnish a benzyl chloride species, regenerating the Pd(0) catalyst. A base-assisted elimination then produces the Mizoroki–Heck coupling product. However, we cannot fully exclude the possibilities of the engagement of photoexcited Pd species or higher order Pd species in direct single-electron oxidation events.

In conclusion, a general intermolecular Pd-catalyzed Mizoroki–Heck coupling of alkyl chlorides enabled by photoexcited Pd catalysis was reported. The developed reaction conditions are applicable to a wide range of styrenes and other activated olefins as well as diverse primary, secondary, and even tertiary alkyl chlorides. The synthetic utility of this reaction was demonstrated via late-stage functionalization of bioactive molecules and pharmaceutical scaffolds in the context of both olefins and alkyl chlorides. Notably, an iterative double Mizoroki–Heck coupling of 1,5-dichlorides was possible under identical reaction conditions, yielding a series of complex structures in a single step. Concrete mechanistic studies using experimental and computational methods revealed that an oxidative pathway is operative rather than the previously reported β-hydride elimination, rationally overcoming the mechanistic challenge of two-electron-based Pd-catalyzed alkyl Mizoroki–Heck coupling. We anticipate that this study will provide mechanistic insights to utilize photoinduced Pd catalysis to achieve traditionally unreachable transformations and expand the synthetic utility of the widespread Mizoroki–Heck reaction.

## Methods

### General procedure for the alkyl chloride Mizoroki–Heck coupling

To a 4 mL vial equipped with a stirrer-bar were added Pd(PPh_3_)_4_ (5.8 mg, 0.0050 mmol, 0.050 equiv), K_2_CO_3_ (27.6 mg, 0.20 mmol, 2.0 equiv), the corresponding olefin (0.10 mmol, 1.0 equiv), the corresponding alkyl chloride (0.15 mmol, 1.5 equiv), and *N*,*N*-dimethylacetamide (1.0 mL). The resulting mixture was stirred for 24 h under 40 W blue light-emitting diode irradiation with fan cooling (~30 °C). The reaction mixture was added brine (10 mL), diluted with EtOAc or Et_2_O (10 mL), washed with brine (10 mL), dried (anhydrous Na_2_SO_4_), filtered, and concentrated under reduced pressure. The resulting residue was purified by flash column chromatography (silica gel, hexanes/EtOAc, or hexanes/Et_2_O gradient elution) to afford the desired product.

## Supplementary information

Supplementary Information

Description of Additional Supplementary Files

Supplementary Data 1

## Data Availability

Detailed experimental procedures, computational details, and all characterization data for new compounds are available from the Supplementary Information. Cartesian coordinates of DFT-optimized structures are available from the Supplementary Data 1 file. All data are available from the corresponding authors upon reasonable request.
