# Supplementary material for: Pd-catalyzed formal Mizoroki–Heck coupling of unactivated alkyl chlorides
Source: Nat Commun. 2021 Feb 12;12:991. doi: 10.1038/s41467-021-21270-9 (PMC7881129; doi:10.1038/s41467-021-21270-9)
Supplement: Supplementary file 1 — Supplementary Information [file 41467_2021_21270_MOESM1_ESM.pdf]

Pd-catalyzed Formal Mizoroki-Heck Coupling of Unactivated Alkyl Chlorides

Geun Seok Lee, Daeun Kim, and Soon Hyeok Hong*

Department of Chemistry, Korea Advanced Institute of Science and Technology (KAIST), Daejeon 34141, Republic of Korea

E-mail: Soon Hyeok Hong (soonhyeok.hong@kaist.ac.kr)

Table of contents

1. General information (S2)
2. Substrate preparation (S2)
3. General procedure for the alkyl chloride Mizoroki-Heck coupling reaction (S3)
4. Characterization data (S3)
5. *E*/*Z* isomerization experiment (S18)
6. Radical trap/clock experiments (S19)
7. Stern-Volmer quenching experiments (S20)
8. Kinetic isotope effect measurements (S21)
9. Elimination of benzyl chloride intermediate (S29)
10. Control experiment with *tert*-butyl *N*-(acyloxy)phthalimide (S30)
11. Computational details (S31)
12. ^{1}H and ^{13}C NMR spectra (S35)
13. Supplementary references (S78)

1. General information

Unless otherwise noted, all reactions were performed under inert conditions. Nuclear magnetic resonance (NMR) spectra were recorded in $CDCl_3$ on a Bruker AVANCE 300 (300 MHz), Bruker AVANCE 400 (400 MHz), or Bruker AVANCE III HD (400 MHz), and the residual solvent signal was used as a reference. High-resolution mass spectrometry (HRMS) was performed at the Organic Chemistry Research Center in Sogang University using the ESI method or the Korea Basic Science Institute (KBSI) using the EI method. Chemical shifts are reported in ppm and coupling constants are given in Hz. Gas chromatography (GC) was carried out using a 7890A or 7890B GC system (Agilent Technologies) equipped with an HP-5 column and a flame ionization detector (FID). Reactions were monitored by thin-layer chromatography (TLC) on EMD Silica Gel 60 F254 plates, and visualized either using UV light (254 nm) or by staining with potassium permanganate and heating. Anhydrous *N,N*-dimethylacetamide (DMA) was purchased from Sigma-Aldrich and used after typical degassing procedures. All chemicals were purchased from commercial sources (Sigma-Aldrich, Alfa Aesar, TCI, or Strem) and used without further purification. All photocatalytic reactions were conducted under irradiation by 40 W Blue LED lamps purchased from Kessil (Kessil A160WE) using the maximum light intensity and the shortest wavelength.

2. Substrate preparation

Alkyl chlorides **2d**,[1] **2f**,[2] **2g**,[2] **2q**,[3] **2ai**,[4] and **2aj**[5] were prepared following literature procedures. Other non-commercial alkyl chlorides **2ae**, **2af**, and **2ag** were prepared following the procedure provided below. Olefins **1v**[6] and **1x**[7] were prepared following literature procedures. Olefin **1w** was prepared following the procedure provided below. All other substrates were purchased from commercial sources and used directly without further purification and the removal of stabilizers from olefins was not necessary.

Preparation of alkyl chlorides 2ae–2ag

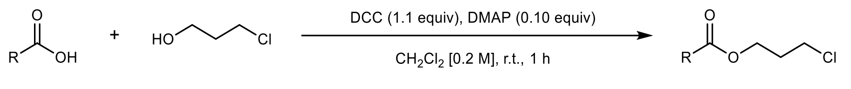

To a flask equipped with a stirrer-bar were added the corresponding carboxylic acid (1.0 mmol, 1.0 equiv), *N,N'*-dicyclohexylcarbodiimide (DCC, 227 mg, 1.1 mmol, 1.1 equiv), 4-(dimethylamino)pyridine (12.2 mg, 0.10 mmol, 0.10 equiv), 3-chloro-1-propanol (125 µL, 1.5 mmol, 1.5 equiv), and CH_2Cl_2 (5.0 mL). The resulting mixture was stirred at room temperature for 1 h and filtered to remove the urea by-product. The resulting mixture was concentrated under reduced pressure and purified by flash column chromatography (silica gel, hexanes/EtOAc gradient elution) to afford the desired products **2ae**–**2ag**.

Preparation of olefin 1w

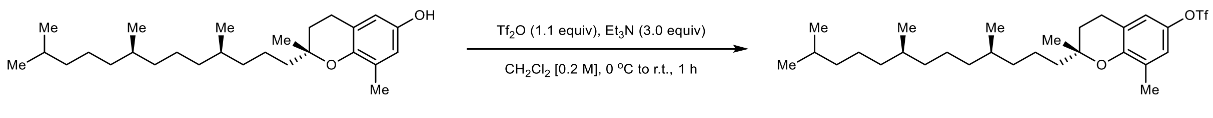

To a stirred solution of δ-tocopherol (403 mg, 1.0 mmol, 1.0 equiv), triethylamine (418 µL, 3.0 mmol, 3.0 equiv) in CH_2Cl_2 (5.0 mL) was added trifluoromethanesulfonic anhydride (310 mg, 1.1 mmol, 1.1 equiv) dropwise at 0 °C. The resulting mixture was warmed up to room temperature and stirred for 1 h. The resulting mixture was quenched with $NaHCO_3$ (sat. aq, 10 mL), extracted with CH_2Cl_2 (10 mL × 3), dried (anhydrous Na_2SO_4), filtered, and concentrated under reduced pressure. The resulting mixture was purified by flash column chromatography (silica gel, hexanes/EtOAc gradient elution) to afford the desired product as a yellow oil (530 mg, 99% yield).

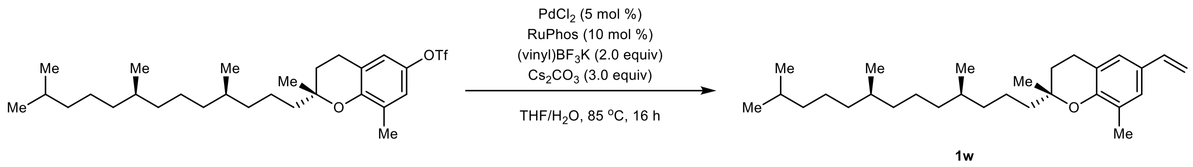

To a flask equipped with a stirrer-bar was added δ-tocopherol trifluoromethanesulfonate (428 mg, 0.80 mmol, 1.0 equiv), $PdCl_2$ (7.1 mg, 0.040 mmol, 0.050 equiv), RuPhos (37.3 mg, 0.080 mmol, 0.10 equiv), potassium vinyltrifluoroborate (214 mg, 2.0 mmol, 2.0 equiv), cesium carbonate (977 mg, 3.0 mmol, 3.0 equiv), THF (2.0 mL) and water (0.30 mL). The resulting mixture was stirred for 16 h at 85 °C and cooled to room temperature before the reaction mixture was quenched with $NaHCO_3$ (sat. aq, 10 mL), extracted with CH_2Cl_2 (10 mL × 3), dried (anhydrous Na_2SO_4), filtered, and concentrated under reduced pressure. The resulting mixture was purified by flash column chromatography (silica gel, hexanes/EtOAc gradient elution) to afford the desired product **1x** as a viscous colorless oil (248 mg, 75% yield).

3. General procedure for the alkyl chloride Mizoroki-Heck coupling reaction

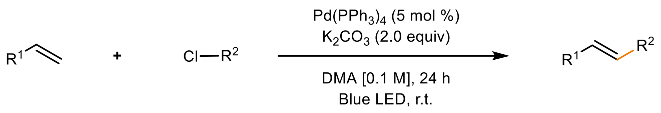

To a 4 mL vial equipped with a stirrer-bar were added $Pd(PPh_3)_4$ (5.8 mg, 0.0050 mmol, 0.050 equiv), K_2CO_3 (27.6 mg, 0.20 mmol, 2.0 equiv), the corresponding olefin (0.10 mmol, 1.0 equiv), the corresponding alkyl chloride (0.15 mmol, 1.5 equiv), and *N*,*N*-dimethylacetamide (1.0 mL). The resulting mixture was stirred for 24 h under 40 W blue LED irradiation with fan cooling (~30 °C). The reaction mixture was added brine (10 mL), diluted with EtOAc or Et_2O (10 mL), washed with brine (10 mL), dried (anhydrous Na_2SO_4), filtered, and concentrated under reduced pressure. The resulting residue was purified by flash column chromatography (silica gel, hexanes/EtOAc or hexanes/Et_2O gradient elution) to afford the desired product.

4. Characterization data

New substrates (**2ae, 2af, 2ag, 1w**) were characterized by ^1H NMR, ^{13}C NMR and HRMS. The identity of the reported Heck coupling products (**3a–3c, 3f, 3h–3s, 3u–3ab, 4a–7a, 9a–20a, 22a–24a, 3ac, 3ah, 3ai**) were confirmed by ^1H NMR and spectral comparison with literature data, while new products (**3d, 3e, 3g, 3t, 3y, 8a, 21a, 3ad, 25a, 3ae, 3af, 26a, 3ag, 3aj**) were characterized by ^1H NMR, ^{13}C NMR and HRMS.

References to characterization data for the reported compounds:

(**3a**, **3b**, **3l**, **3n**, **3o**, **3w**, **3z**, **3aa**, **7a**, **9a**, **12a**, **16a**, **18a**, **20a**, **23a**, **3ac**)[8]; **3c**[9]; (**3f**, **3p**)[10]; (**3h**, **3i**, **3k**, **3s**, **11a**, **17a**)[11]; **3j**[12]; **3m**[13]; (**3q**, **15a**)[14]; **3r**[15]; (**3u**, **3v**, **3x**)[16]; **3ab**[17]; (**4a**, **10a**, **13a**)[18]; **5a**[19]; **6a**[20]; **14a**[21]; **19a**[22]; **22a**[23]; **3ah**[24]; **3ai**[25].

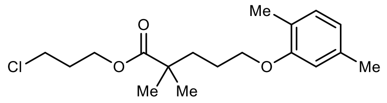

3-Chloropropyl 5-(2,5-dimethylphenoxy)-2,2-dimethylpentanoate (2ae)

Colorless oil, 261 mg (0.80 mmol, 80% yield); ^1H NMR (400 MHz, $CDCl_3$): δ = 7.06 (dd, J = 7.5, 1.1 Hz, 1H),

6.71 (d, J = 7.5 Hz, 1H), 6.66 (s, 1H), 4.27 (td, J = 6.1, 0.9 Hz, 2H), 3.98 (tt, J = 4.9, 1.2 Hz, 2H), 3.65 (td, J = 6.4, 0.8 Hz, 2H), 2.36 (s, 3H), 2.24 (s, 3H), 2.18 – 2.08 (m, 2H), 1.82 – 1.76 (m, 4H), 1.28 (d, J = 1.2 Hz, 6H); ^{13}C NMR (100 MHz, $CDCl_3$): δ = 177.6, 156.9, 136.5, 130.3, 123.6, 120.8, 112.0, 67.9, 61.1, 42.2, 41.2, 37.2, 31.6, 25.2, 25.2, 21.4, 15.8; HRMS-ESI (m/z) $[M+Na]^+$ calcd for $C_{18}H_{27}ClO_3Na$, 349.1541; found: 349.1543.

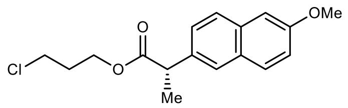

3-Chloropropyl (S)-2-(6-methoxynaphthalen-2-yl)propanoate (2af)

White solid, 278 mg (0.91 mmol, 91% yield); ^{1}H NMR (600 MHz, $CDCl_3$): δ = 7.61 (dd, J = 9.2, 4.6 Hz, 2H), 7.57 (s, 1H), 7.31 (d, J = 8.8 Hz, 1H), 7.06 (d, J = 9.0 Hz, 1H), 7.02 (s, 1H), 4.13 (q, J = 6.1 Hz, 2H), 3.78 (s, 3H), 3.33 (td, J = 6.6, 6.0, 3.3 Hz, 2H), 1.89 (t, J = 6.4 Hz, 2H), 1.49 (d, J = 7.4 Hz, 2H); ^{13}C NMR (75 MHz, $CDCl_3$): δ = 174.5, 157.7, 135.6, 135.6, 133.7, 129.3, 129.2, 128.9, 127.2, 127.2, 126.2, 126.1, 125.9, 125.9, 119.1, 119.0, 105.6, 105.5, 61.4, 61.3, 55.3, 55.2, 55.2, 45.4, 45.4, 45.4, 41.1, 41.0, 31.5, 31.4, 18.5, 18.4; HRMS-ESI (m/z) $[M+Na]^+$ calcd for $C_{17}H_{19}ClO_3Na$, 329.0915; found: 329.0916.

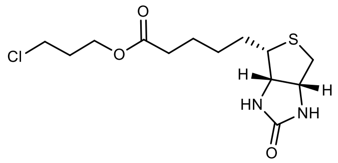

3-Chloropropyl 5-((3aS,4S,6aR)-2-oxohexahydro-1H-thieno[3,4-d]imidazol-4-yl)pentanoate (2ag)

White solid, 317 mg (0.98 mmol, 98% yield); ^{1}H NMR (400 MHz, $CDCl_3$) δ 6.32 (s, 1H), 5.95 (s, 1H), 4.47 (t, J = 6.4 Hz, 1H), 4.33 – 4.22 (m, 1H), 4.18 (t, J = 6.1 Hz, 2H), 3.58 (t, J = 6.4 Hz, 2H), 3.17 – 3.05 (m, 1H), 2.87 (dd, J = 12.7, 4.6 Hz, 1H), 2.70 (d, J = 12.5 Hz, 1H), 2.31 (t, J = 7.5 Hz, 2H), 2.06 (p, J = 6.2 Hz, 2H), 1.74 – 1.53 (m, J = 7.8 Hz, 4H), 1.49 – 1.32 (m, 2H); ^{13}C NMR (100 MHz, $CDCl_3$) δ 173.6, 62.0, 61.1, 60.2, 55.6, 41.4, 40.6, 33.9, 31.5, 28.4, 28.3, 24.8; HRMS-ESI (m/z) $[M+Na]^+$ calcd for $C_{13}H_{21}ClN_2O_3SNa$, 343.0854; found: 343.0856.

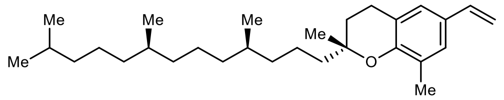

(R)-2,8-Dimethyl-2-((4R,8R)-4,8,12-trimethyltridecyl)-6-vinylchromane (1w)

Viscous colorless oil, 272 mg (0.53 mmol, 66% yield); ^{1}H NMR (300 MHz, $CDCl_3$): δ = 7.03 (d, J = 2.2 Hz, 1H), 6.93 (d, J = 2.3 Hz, 1H), 6.57 (dd, J = 17.6, 10.9 Hz, 1H), 5.53 (dd, J = 17.6, 1.2 Hz, 1H), 5.03 (dd, J = 10.8, 1.2 Hz, 1H), 2.72 (t, J = 6.7 Hz, 2H), 2.14 (s, 3H), 1.87 – 1.67 (m, 2H), 1.62 – 0.97 (m, 21H), 0.88 – 0.79 (m, 15H); ^{13}C NMR (100 MHz, $CDCl_3$): δ = 152.4, 137.0, 128.7, 126.6, 126.4, 125.3, 120.5, 110.7, 76.5, 40.3, 39.6, 37.7, 37.7, 37.5, 33.1, 32.9, 31.5, 28.2, 25.1, 24.7, 24.5, 23.0, 22.9, 22.6, 21.2, 20.0, 19.9, 16.4; HRMS-ESI (m/z) $[M+H]^+$ calcd for $C_{29}H_{49}O$, 413.3778; found: 413.3782.

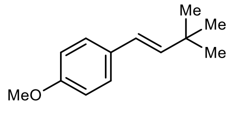

(*E*)-1-(3,3-Dimethylbut-1-en-1-yl)-4-methoxybenzene (3a)

Colorless oil, 18.2 mg (96% yield); ^{1}H NMR (300 MHz, $CDCl_3$): δ = 7.32 – 7.20 (m, 2H), 6.87 – 6.78 (m, 2H), 6.23 (d, *J* = 16.2 Hz, 1H), 6.10 (d, *J* = 16.2 Hz, 1H), 3.78 (s, 3H), 1.09 (s, 9H).

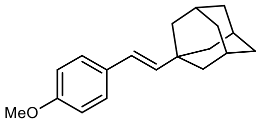

1-((*E*)-4-Methoxystyryl)adamantane (3b)

Colorless oil, 24.2 mg (90% yield); ^{1}H NMR (300 MHz, $CDCl_3$): δ = 7.28 (d, *J* = 8.7 Hz, 2H), 6.82 (d, *J* = 8.8 Hz, 2H), 6.17 (d, *J* = 16.3 Hz, 1H), 5.95 (d, *J* = 16.3 Hz, 1H), 3.78 (s, 3H), 2.06 – 1.94 (m, 3H), 1.78 – 1.60 (m, 12H).

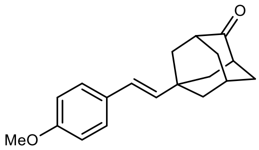

5-((*E*)-4-Methoxystyryl)adamantan-2-one (3c)

White solid, 28.1 mg (>96% yield); ^{1}H NMR (300 MHz, $CDCl_3$): δ = 7.30 – 7.24 (m, 2H), 6.82 (d, *J* = 8.8 Hz, 2H), 6.23 (d, *J* = 16.3 Hz, 1H), 5.94 (d, *J* = 16.3 Hz, 1H), 3.78 (s, 3H), 2.58 (s, 2H), 2.18 (d, *J* = 3.2 Hz, 1H), 2.05 – 1.89 (m, 10H).

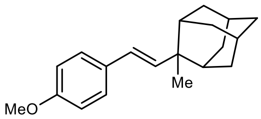

5-((*E*)-4-Methoxystyryl)adamantan-2-one (3d)

White solid, 20.6 mg (73% yield); ^{1}H NMR (300 MHz, $CDCl_3$): δ = 7.32 – 7.26 (m, 2H), 6.89 – 6.79 (m, 2H), 6.24 (d, *J* = 16.6 Hz, 1H), 6.12 (d, *J* = 16.6 Hz, 1H), 3.85 – 3.74 (m, 3H), 2.09 (d, *J* = 11.7 Hz, 4H), 1.87 – 1.49 (m, 10H), 1.15 (s, 3H); ^{13}C NMR (100 MHz, $CDCl_3$): δ = 158.7, 140.6, 131.5, 127.0, 125.6, 114.1, 114.1, 55.6, 41.4, 39.2, 36.8, 34.3, 33.2, 28.3, 28.1, 27.3; HRMS-EI (m/z) $[M]^+$ calcd for $C_{20}H_{26}O$, 282.1984; found: 282.1982.

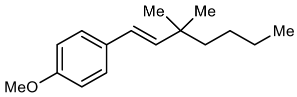

(*E*)-1-(3,3-Dimethylhept-1-en-1-yl)-4-methoxybenzene (3e)

Colorless oil, 22.3 mg (96% yield); ^{1}H NMR (400 MHz, $CDCl_3$): δ = 7.34 – 7.26 (m, 2H), 6.83 (d, *J* = 8.8 Hz, 2H), 6.20 (d, *J* = 16.3 Hz, 1H), 6.03 (d, *J* = 16.2 Hz, 1H), 3.79 (s, 3H), 1.36 – 1.30 (m, 2H), 1.30 – 1.15 (m, 4H), 1.06 (s, 6H), 0.87 (t, *J* = 7.0 Hz, 3H); ^{13}C NMR (100 MHz, $CDCl_3$): δ = 158.8, 139.1, 131.2, 127.2, 125.1, 114.1, 55.5, 43.4, 36.3, 27.5, 27.2, 23.7, 14.4; HRMS-EI (m/z) $[M]^+$ calcd for $C_{16}H_{24}O$, 232.1827; found: 232.1827.

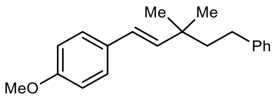

(*E*)-1-(3,3-Dimethyl-5-phenylpent-1-en-1-yl)-4-methoxybenzene (3f)

White solid, 25.3 mg (90% yield); ^{1}H NMR (300 MHz, $CDCl_3$): δ = 7.36 – 7.21 (m, 4H), 7.16 (d, *J* = 7.2 Hz, 3H), 6.94 – 6.76 (m, 2H), 6.28 (d, *J* = 16.2 Hz, 1H), 6.08 (d, *J* = 16.2 Hz, 1H), 3.80 (s, 3H), 2.64 – 2.48 (m, 2H), 1.73 – 1.60 (m, 2H), 1.15 (s, 6H).

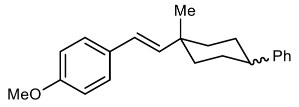

(*E*)-1-Methoxy-4-(2-(1-methyl-4-phenylcyclohexyl)vinyl)benzene (3g)

White solid, 29.1 mg (95% yield, 2:1 mixture of diastereomers); ^{1}H NMR (600 MHz, $CDCl_3$, mixture of diastereomers): δ = 7.38 – 7.31 (m, 8H), 7.30 – 7.25 (m, 5H), 7.23 – 7.15 (m, 8H), 6.90 – 6.85 (m, 6H), 6.36 (d, *J* = 16.4 Hz, 2H), 6.32 (d, *J* = 16.3 Hz, 1H), 6.15 (d, *J* = 16.1 Hz, 1H), 6.13 (d, *J* = 16.5 Hz, 2H), 3.82 (s, 6H), 3.82 (s, 3H), 2.55 – 2.47 (m, 3H), 1.97 – 1.91 (m, 4H), 1.84 – 1.46 (m, 20H), 1.21 (s, 3H), 1.11 (s, 6H).; ^{13}C NMR (150 MHz, $CDCl_3$, mixture of diastereomers): δ = 158.7, 158.7, 147.5, 147.5, 141.2, 136.2, 131.0, 130.9, 128.3, 128.3, 127.1, 127.0, 127.0, 126.9, 126.9, 125.9, 125.8, 123.9, 114.0, 113.9, 55.3, 55.3, 44.4, 44.3, 38.6, 37.5, 36.0, 35.1, 31.9, 30.5, 29.7, 22.2; HRMS-EI (m/z) $[M]^+$ calcd for $C_{22}H_{26}O$, 306.1984; found: 306.1985.

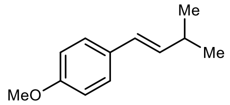

(*E*)-1-Methoxy-4-(3-methylbut-1-en-1-yl)benzene (3h)

Colorless oil, 16.2 mg (92% yield); ^{1}H NMR (300 MHz, $CDCl_3$): δ = 7.35 – 7.21 (m, 2H), 6.87 – 6.77 (m, 2H), 6.26 (d, *J* = 15.9 Hz, 1H), 6.03 (dd, *J* = 15.9, 6.8 Hz, 1H), 3.78 (s, 3H), 2.50 – 2.33 (m, 1H), 1.06 (d, *J* = 6.8 Hz, 6H).

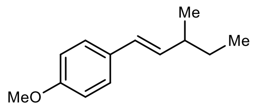

(*E*)-1-Methoxy-4-(3-methylpent-1-en-1-yl)benzene (3i)

Colorless oil, 19.0 mg (>96% yield); ^{1}H NMR (300 MHz, $CDCl_3$): δ = 7.29 – 7.25 (m, 2H), 6.82 (d, *J* = 8.8 Hz, 2H), 6.27 (d, *J* = 15.9 Hz, 1H), 5.93 (dd, *J* = 15.8, 7.8 Hz, 1H), 3.78 (s, 3H), 2.15 (p, *J* = 6.9 Hz, 1H), 1.44 – 1.32 (m, 2H), 1.04 (d, *J* = 6.7 Hz, 3H), 0.88 (t, *J* = 7.4 Hz, 3H).

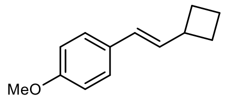

(*E*)-1-(2-Cyclobutylvinyl)-4-methoxybenzene (3j)

Colorless oil, 17.0 mg (90% yield); ^{1}H NMR (300 MHz, $CDCl_3$): δ = 7.32 – 7.23 (m, 2H), 6.88 – 6.75 (m, 2H), 6.27 – 6.16 (m, 2H), 3.78 (s, 3H), 3.13 – 2.95 (m, 1H), 2.24 – 2.05 (m, 2H), 2.00 – 1.73 (m, 4H).

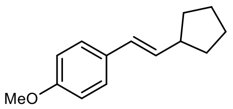

(*E*)-1-(2-Cyclopentylvinyl)-4-methoxybenzene (3k)

Colorless oil, 19.6 mg (>96% yield); ^{1}H NMR (300 MHz, $CDCl_3$): δ = 7.31 – 7.20 (m, 2H), 6.89 – 6.77 (m, 2H), 6.29 (d, *J* = 15.8 Hz, 1H), 6.04 (dd, *J* = 15.8, 7.7 Hz, 1H), 3.78 (s, 4H), 2.62 – 2.48 (m, 1H), 1.90 – 1.76 (m, 2H), 1.70 – 1.51 (m, 4H), 1.45 – 1.27 (m, 2H).

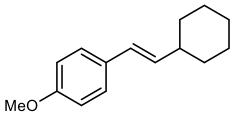

(*E*)-1-(2-Cyclohexylvinyl)-4-methoxybenzene (3l)

Colorless oil, 20.7 mg (96% yield); ^{1}H NMR (300 MHz, $CDCl_3$): δ = 7.26 (dd, *J* = 8.5, 1.8 Hz, 2H), 6.93 – 6.75 (m, 2H), 6.33 – 6.21 (m, 1H), 6.01 (dd, *J* = 16.0, 6.9 Hz, 1H), 3.78 (s, 3H), 2.16 – 1.99 (m, 1H), 1.85 – 1.59 (m, 5H), 1.40 – 1.07 (m, 5H).

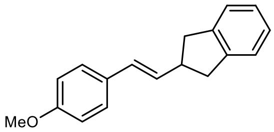

(*E*)-2-(4-Methoxystyryl)-2,3-dihydro-1H-indene (3m)

Bright yellow solid, 24.5 mg (>96% yield); ^{1}H NMR (300 MHz, $CDCl_3$): δ = 7.38 – 7.13 (m, 8H), 6.88 – 6.80 (m, 2H), 6.43 (d, *J* = 15.8 Hz, 1H), 6.22 (dd, *J* = 15.8, 7.6 Hz, 1H), 3.79 (s, 3H), 3.30 – 3.20 (m, 1H), 3.20 – 3.06 (m, 2H), 2.85 (dd, *J* = 15.1, 8.0 Hz, 2H).

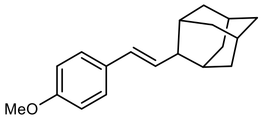

2-((*E*)-4-Methoxystyryl)adamantane (3n)

Colorless solid, 17.0 mg (63% yield); ^{1}H NMR (300 MHz, $CDCl_3$): δ = 7.36 – 7.27 (m, 2H), 6.87 – 6.80 (m, 2H), 6.41 – 6.31 (m, 2H), 3.78 (s, 3H), 2.52 (s, 1H), 1.99 (d, *J* = 12.7 Hz, 2H), 1.93 – 1.80 (m, 6H), 1.73 (s, 2H), 1.55 (d, *J* = 12.5 Hz, 2H).

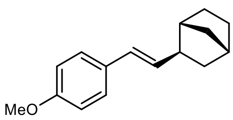

2-((*E*)-4-Methoxystyryl)bicyclo[2.2.1]heptane (3o)

Colorless oil, 21.6 mg (95% yield); ^{1}H NMR (300 MHz, $CDCl_3$): δ = 7.30 – 7.19 (m, 2H), 6.86 – 6.77 (m, 2H), 6.22 (d, *J* = 15.8 Hz, 1H), 5.96 (dd, *J* = 15.8, 8.1 Hz, 1H), 3.78 (s, 3H), 2.29 – 2.14 (m, 2H), 2.09 (s, 1H), 1.57 – 1.45 (m, 3H), 1.45 – 1.38 (m, 1H), 1.37 – 1.26 (m, 2H), 1.18 – 1.07 (m, 2H).

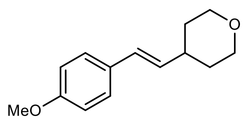

(*E*)-4-(4-Methoxystyryl)tetrahydro-2H-pyran (3p)

Yellow oil, 22.1 mg (>96% yield); ^{1}H NMR (300 MHz, $CDCl_3$): δ = 7.27 (d, *J* = 8.7 Hz, 2H), 6.82 (d, *J* = 8.7 Hz, 2H), 6.31 (dd, *J* = 15.9, 1.3 Hz, 1H), 6.00 (dd, *J* = 16.0, 6.8 Hz, 1H), 3.98 (ddd, *J* = 11.4, 4.5, 2.0 Hz, 2H), 3.78 (s, 3H), 3.44 (td, *J* = 11.6, 2.3 Hz, 2H), 2.43 – 2.21 (m, 1H), 1.77 – 1.63 (m, 2H), 1.63 – 1.49 (m, 2H).

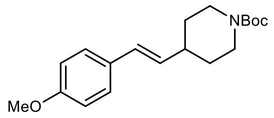

***tert*-Butyl (*E*)-4-(4-methoxystyryl)piperidine-1-carboxylate (3q)**

Yellow oil, 27.6 mg (87% yield); ^{1}H NMR (300 MHz, $CDCl_3$): δ = 7.25 (d, *J* = 8.8 Hz, 2H), 6.82 (d, *J* = 8.7 Hz, 2H), 6.31 (d, *J* = 15.9 Hz, 1H), 5.98 (dd, *J* = 16.0, 6.9 Hz, 1H), 4.10 (s, 2H), 3.78 (s, 3H), 2.75 (t, *J* = 12.6 Hz, 2H), 2.33 – 2.14 (m, 1H), 1.72 (d, *J* = 13.2 Hz, 2H), 1.45 (s, 9H), 1.35 (dt, *J* = 12.5, 6.0 Hz, 2H).

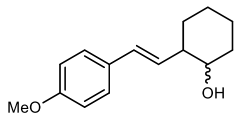

(*E*)-2-(4-Methoxystyryl)cyclohexan-1-ol (3r)

Yellow oil, 20.1 mg (87% yield, 1:2.7 mixture of diastereomers); ^{1}H NMR (400 MHz, $CDCl_3$, reported for the major isomer): δ = 7.38 – 7.31 (m, 2H), 6.91 – 6.84 (m, 2H), 6.50 (d, *J* = 15.9 Hz, 1H), 5.94 (dd, *J* = 15.8, 8.9 Hz, 1H), 3.83 (s, 3H), 3.35 (td, *J* = 10.0, 4.3 Hz, 1H), 2.07 (t, *J* = 12.7 Hz, 2H), 1.89 – 1.60 (m, 4H), 1.40 – 1.20 (m, 4H).

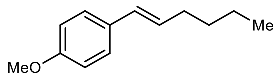

(*E*)-1-(Hex-1-en-1-yl)-4-methoxybenzene (3s)

Yellow oil, 15.2 mg (80% yield); ^{1}H NMR (300 MHz, $CDCl_3$): δ = 7.25 (d, *J* = 8.6 Hz, 2H), 6.81 (d, *J* = 8.7 Hz, 2H), 6.30 (d, *J* = 15.8 Hz, 1H), 6.06 (dt, *J* = 15.7, 6.9 Hz, 1H), 3.78 (s, 3H), 2.24 – 2.11 (m, 2H), 1.48 – 1.31 (m, 4H), 0.90 (t, *J* = 7.0 Hz, 3H).

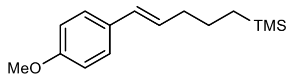

(*E*)-(5-(4-Methoxyphenyl)pent-4-en-1-yl)trimethylsilane (3t)

Yellow oil, 18.3 mg (74% yield); ^{1}H NMR (300 MHz, $CDCl_3$): δ = 7.27 (d, *J* = 8.8 Hz, 2H), 6.82 (d, *J* = 8.7 Hz, 2H), 6.37 – 6.25 (m, 1H), 6.06 (dt, *J* = 15.8, 6.9 Hz, 1H), 3.78 (s, 3H), 2.18 (qd, *J* = 7.1, 1.4 Hz, 2H), 1.54 – 1.36 (m, 2H), 0.62 – 0.45 (m, 2H), -0.03 (s, 9H);^{13}C NMR (100 MHz, $CDCl_3$): δ = 158.8, 131.0, 129.5, 129.1, 127.2, 114.1, 55.5, 37.1, 24.3, 16.6, –1.4; HRMS-EI (m/z) $[M]^+$ calcd for $C_{15}H_{24}OSi$, 248.1596; found: 248.1595.

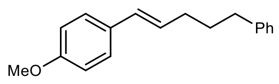

(*E*)-1-Methoxy-4-(5-phenylpent-1-en-1-yl)benzene (3u)

Yellow oil, 21.0 mg (83% yield); ^{1}H NMR (300 MHz, $CDCl_3$): δ = 7.31 – 7.22 (m, 4H), 7.22 – 7.14 (m, 3H), 6.85 – 6.77 (m, 2H), 6.32 (d, *J* = 15.8 Hz, 1H), 6.08 (dt, *J* = 15.6, 6.8 Hz, 1H), 3.78 (s, 3H), 2.72 – 2.59 (m, 2H), 2.26 – 2.17 (m, 2H), 1.88 – 1.71 (m, 2H).

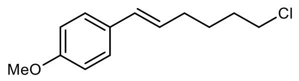

(*E*)-1-(6-Chlorohex-1-en-1-yl)-4-methoxybenzene (3v)

Yellow oil, 16.7 mg (74% yield); ^{1}H NMR (300 MHz, $CDCl_3$) δ 7.29 – 7.22 (m, 2H), 6.85 – 6.78 (m, 2H), 6.32 (d, *J* = 15.8 Hz, 1H), 6.03 (dt, *J* = 15.8, 6.9 Hz, 1H), 3.78 (s, 3H), 3.55 (t, *J* = 6.7 Hz, 2H), 2.25 – 2.15 (m, 2H), 1.86 – 1.74 (m, 2H), 1.66 – 1.56 (m, 3H).

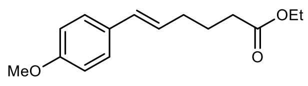

Ethyl (*E*)-6-(4-methoxyphenyl)hex-5-enoate (3w)

Yellow oil, 16.7 mg (67% yield); ^{1}H NMR (300 MHz, $CDCl_3$): δ = 7.27 – 7.23 (m, 2H), 6.81 (d, *J* = 8.7 Hz, 2H), 6.32 (d, *J* = 15.8 Hz, 1H), 6.01 (dt, *J* = 15.7, 6.9 Hz, 1H), 4.10 (q, *J* = 7.1 Hz, 2H), 3.78 (s, 3H), 2.32 (t, *J* = 7.5 Hz, 2H), 2.21 (q, *J* = 6.6 Hz, 2H), 1.78 (p, *J* = 7.3 Hz, 2H), 1.23 (t, *J* = 7.1 Hz, 3H).

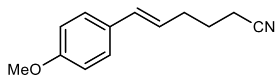

(*E*)-6-(4-Methoxyphenyl)hex-5-enenitrile (3x)

Yellow oil, 16.7 mg (67% yield); ^{1}H NMR (300 MHz, $CDCl_3$): δ = 7.28 – 7.20 (m, 2H), 6.83 (d, *J* = 8.7 Hz, 2H), 6.38 (d, *J* = 15.8 Hz, 1H), 5.96 (dt, *J* = 15.7, 7.1 Hz, 1H), 3.79 (s, 3H), 2.43 – 2.27 (m, 4H), 1.81 (p, *J* = 7.1 Hz, 2H).

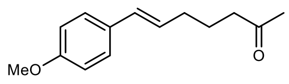

(*E*)-7-(4-Methoxyphenyl)hept-6-en-2-one (3y)

Yellow oil, 15.3 mg (70% yield); ^{1}H NMR (400 MHz, $CDCl_3$): δ = 7.25 (d, *J* = 7.4 Hz, 2H), 6.82 (d, *J* = 8.8 Hz, 2H), 6.31 (d, *J* = 15.8 Hz, 1H), 6.00 (dt, *J* = 15.8, 7.0 Hz, 1H), 3.78 (s, 3H), 2.45 (t, *J* = 7.4 Hz, 2H), 2.18 (qd, *J* = 7.1, 1.4 Hz, 2H), 2.11 (s, 3H), 1.73 (p, *J* = 7.3 Hz, 2H).;^{13}C NMR (100 MHz, $CDCl_3$): δ = 209.2, 159.0, 130.6, 130.2, 127.8, 127.3, 114.1, 55.5, 43.1, 32.5, 30.2, 23.6; HRMS-EI (m/z) $[M]^+$ calcd for $C_{14}H_{18}O_2$, 218.1307; found: 218.1304.

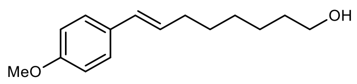

(*E*)-8-(4-Methoxyphenyl)oct-7-en-1-ol (3z)

Yellow oil, 16.1 mg (69% yield); ^{1}H NMR (300 MHz, $CDCl_3$): δ = 7.30 – 7.20 (m, 2H), 6.81 (d, *J* = 8.7 Hz, 2H), 6.41 – 6.22 (m, 1H), 6.05 (dt, *J* = 15.7, 6.9 Hz, 1H), 3.78 (s, 3H), 3.63 (t, *J* = 6.6 Hz, 2H), 2.17 (qd, *J* = 7.0, 1.4 Hz, 2H), 1.58 – 1.33 (m, 8H).

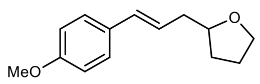

(*E*)-2-(3-(4-Methoxyphenyl)allyl)tetrahydrofuran (3aa)

Yellow oil, 16.7 mg (77% yield); ^{1}H NMR (300 MHz, $CDCl_3$): δ = 7.31 – 7.22 (m, 2H), 6.81 (d, *J* = 8.8 Hz, 2H), 6.45 – 6.30 (m, 1H), 6.07 (dt, *J* = 15.7, 7.1 Hz, 1H), 3.99 – 3.84 (m, 2H), 3.78 (d, *J* = 0.7 Hz, 3H), 3.77 – 3.66 (m, 1H), 2.56 – 2.28 (m, 2H), 2.06 – 1.75 (m, 3H), 1.64 – 1.48 (m, 1H).

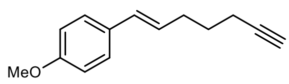

(*E*)-1-(Hept-1-en-6-yn-1-yl)-4-methoxybenzene (3ab)

Yellow oil, 18.3 mg (91% yield); ^{1}H NMR (300 MHz, $CDCl_3$): δ = 7.25 (d, *J* = 8.9 Hz, 2H), 6.82 (d, *J* = 8.8 Hz, 2H), 6.34 (d, *J* = 15.8 Hz, 1H), 6.03 (dt, *J* = 15.8, 7.0 Hz, 1H), 3.78 (s, 3H), 2.39 – 2.17 (m, 4H), 1.95 (t, *J* = 2.6 Hz, 1H), 1.68 (p, *J* = 7.2 Hz, 2H).

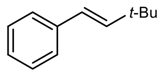

(*E*)-(3,3-Dimethylbut-1-en-1-yl)benzene (4a)

Yellow oil, 14.8 mg (92% yield); ^{1}H NMR (300 MHz, $CDCl_3$): δ = 7.37 – 7.30 (m, 2H), 7.30 – 7.24 (m, 2H), 7.19 – 7.12 (m, 1H), 6.36 – 6.14 (m, 2H), 1.10 (s, 9H).

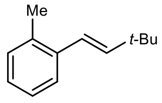

(*E*)-1-(3,3-Dimethylbut-1-en-1-yl)-2-methylbenzene (5a)

Yellow oil, 17.4 mg (>96% yield); ^{1}H NMR (300 MHz, $CDCl_3$): δ = 7.47 – 7.33 (m, 1H), 7.19 – 7.04 (m, 3H), 6.47 (d, *J* = 16.0 Hz, 1H), 6.10 (d, *J* = 16.0 Hz, 1H), 2.32 (s, 3H), 1.15 – 1.09 (m, 9H).

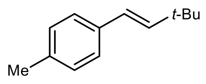

(*E*)-1-(3,3-Dimethylbut-1-en-1-yl)-4-methylbenzene (6a)

Yellow oil, 14.8 mg (85% yield); ^{1}H NMR (400 MHz, $CDCl_3$): δ = 7.31 – 7.21 (m, 2H), 7.08 (d, *J* = 7.9 Hz,

2H), 6.26 (d, J = 16.1 Hz, 1H), 6.18 (d, J = 16.2 Hz, 1H), 2.31 (s, 3H), 1.10 (s, 9H).

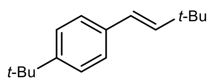

(*E*)-1-(*tert*-Butyl)-4-(3,3-dimethylbut-1-en-1-yl)benzene (7a)

Yellow oil, 21.6 mg (>96% yield); ^{1}H NMR (300 MHz, $CDCl_3$): δ = 7.29 (d, J = 1.2 Hz, 4H), 6.34 – 6.12 (m, 2H), 1.29 (s, 9H), 1.09 (s, 9H).

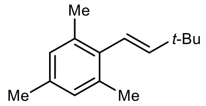

(*E*)-2-(3,3-Dimethylbut-1-en-1-yl)-1,3,5-trimethylbenzene (8a)

Yellow oil, 11.8 mg (58% yield); ^{1}H NMR (600 MHz, $CDCl_3$): δ = 6.85 (s, 2H), 6.18 (d, J = 16.6 Hz, 1H), 5.66 (d, J = 16.6 Hz, 1H), 2.26 (s, 3H), 2.24 (s, 6H), 1.13 (s, 9H); ^{13}C NMR (150 MHz, $CDCl_3$): δ = 146.4, 146.4, 135.8, 135.4, 128.3, 121.7, 33.6, 29.6, 20.9, 20.7; HRMS-EI (m/z) $[M]^+$ calcd for $C_{15}H_{22}$, 202.1722; found: 202.1722.

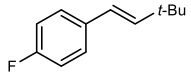

(*E*)-1-(3,3-Dimethylbut-1-en-1-yl)-4-fluorobenzene (9a)

Yellow oil, 17.4 mg (>96% yield); ^{1}H NMR (300 MHz, $CDCl_3$): δ = 7.34 – 7.26 (m, 2H), 7.01 – 6.91 (m, 2H), 6.29 – 6.20 (m, 1H), 6.19 – 6.08 (m, 1H), 1.09 (d, J = 1.3 Hz, 9H).

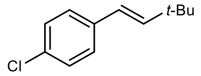

(*E*)-1-(3,3-Dimethylbut-1-en-1-yl)-4-chlorobenzene (10a)

Yellow oil, 12.4 mg (64% yield); ^{1}H NMR (300 MHz, $CDCl_3$): δ = 7.26 – 7.23 (m, 4H), 6.22 (d, J = 0.6 Hz, 2H), 1.09 (s, 9H).

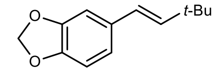

(*E*)-5-(3,3-Dimethylbut-1-en-1-yl)benzo[d][1,3]dioxole (11a)

Yellow oil, 20.6 mg (>96% yield); ^{1}H NMR (300 MHz, $CDCl_3$): δ = 6.90 (d, J = 1.6 Hz, 1H), 6.81 – 6.67 (m, 2H), 6.19 (d, J = 16.1 Hz, 1H), 6.06 (d, J = 16.2 Hz, 1H), 5.91 (s, 2H), 1.08 (s, 9H).

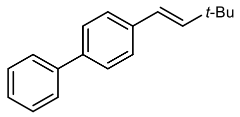

(*E*)-4-(3,3-Dimethylbut-1-en-1-yl)-1,1'-biphenyl (12a)

Yellow oil, 21.3 mg (90% yield, 1.2:1 ratio of stereoisomers); ^{1}H NMR (300 MHz, $CDCl_3$, mixture of isomers, calibrated to the minor isomer): δ = 7.64 – 7.21 (m, 19.8H), 6.41 (d, *J* = 12.7 Hz, 1H), 6.31 (d, *J* = 2.1 Hz, 2.4H), 5.62 (d, *J* = 12.7 Hz, 1H), 1.13 (s, 10.8H), 1.01 (s, 9H).

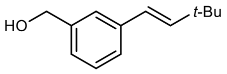

(*E*)-(3-(3,3-Dimethylbut-1-en-1-yl)phenyl)methanol (13a)

Yellow oil, 19.2 mg (>96% yield); ^{1}H NMR (300 MHz, $CDCl_3$): δ = 7.37 (s, 1H), 7.31 – 7.23 (m, 2H), 7.17 (ddd, *J* = 5.4, 2.9, 1.7 Hz, 1H), 6.28 (d, *J* = 0.9 Hz, 2H), 4.67 (s, 2H), 1.10 (s, 9H).

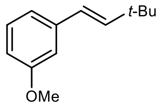

(*E*)-1-(3,3-Dimethylbut-1-en-1-yl)-3-methoxybenzene (14a)

Yellow oil, 16.3 mg (86% yield); ^{1}H NMR (300 MHz, $CDCl_3$): δ = 7.19 (t, *J* = 7.9 Hz, 1H), 6.99 – 6.89 (m, 1H), 6.92 – 6.85 (m, 1H), 6.78 – 6.68 (m, 1H), 6.24 (d, *J* = 0.8 Hz, 2H), 3.80 (s, 3H), 1.10 (s, 9H).

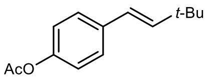

(*E*)-4-(3,3-Dimethylbut-1-en-1-yl)phenyl acetate (15a)

Yellow oil, 21.2 mg (>96% yield); ^{1}H NMR (300 MHz, $CDCl_3$): δ = 7.34 (d, *J* = 8.6 Hz, 2H), 6.99 (d, *J* = 8.6 Hz, 2H), 6.31 – 6.12 (m, 2H), 2.27 (s, 3H), 1.09 (s, 9H).

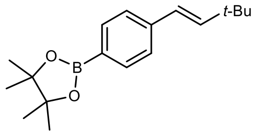

(*E*)-2-(4-(3,3-Dimethylbut-1-en-1-yl)phenyl)-4,4,5,5-tetramethyl-1,3,2-dioxaborolane (16a)

Yellow oil, 18.3 mg (64% yield); ^{1}H NMR (300 MHz, $CDCl_3$): δ = 7.77 – 7.64 (m, 2H), 7.38 – 7.31 (m, 2H), 6.29 (d, *J* = 1.0 Hz, 2H), 1.32 (s, 12H), 1.10 (s, 9H)

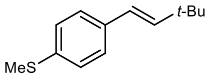

(*E*)-(4-(3,3-Dimethylbut-1-en-1-yl)phenyl)(methyl)sulfane (17a)

Yellow solid, 20.9 mg (>96% yield); ^{1}H NMR (300 MHz, $CDCl_3$): δ = 7.31 – 7.22 (m, 2H), 7.21 – 7.13 (m, 2H), 6.28 – 6.14 (m, 2H), 2.46 (s, 3H), 1.09 (s, 9H).

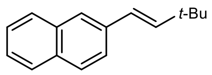

(*E*)-2-(3,3-Dimethylbut-1-en-1-yl)naphthalene (18a)

White solid, 13.5 mg (64% yield, 1:2 ratio of stereoisomers); ^{1}H NMR (300 MHz, $CDCl_3$, mixture of isomers, calibrated to the minor isomer): δ = 7.85 – 7.67 (m, 10H), 7.66 – 7.57 (m, 3H), 7.54 – 7.37 (m, 6H), 7.31 (dd, *J* = 8.4, 1.7 Hz, 2H), 6.53 (d, *J* = 12.6 Hz, 2H), 6.50 – 6.32 (m, 2H), 5.68 (d, *J* = 12.6 Hz, 2H), 1.15 (s, 9H), 0.99 (s, 18H).

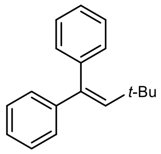

(3,3-dimethylbut-1-ene-1,1-diyl)dibenzene (19a)

Colorless oil, 19.7 mg (83% yield); ^{1}H NMR (300 MHz, $CDCl_3$): δ = 7.37 – 7.26 (m, 5H), 7.21 – 7.14 (m, 5H), 6.07 (s, 1H), 0.95 (s, 9H)

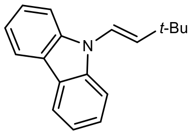

(*E*)-9-(3,3-Dimethylbut-1-en-1-yl)-9H-carbazole (20a)

Yellow solid, 18.8 mg (75% yield); ^{1}H NMR (300 MHz, $CDCl_3$): δ = 8.12 – 8.02 (m, 2H), 7.58 – 7.53 (m, 2H), 7.53 – 7.36 (m, 2H), 7.30 – 7.20 (m, 2H), 6.86 (dd, *J* = 14.5, 1.8 Hz, 1H), 6.19 (dd, *J* = 14.4, 2.0 Hz, 1H), 1.27 (d, *J* = 1.9 Hz, 9H).

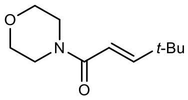

(*E*)-4,4-Dimethyl-1-morpholinopent-2-en-1-one (21a)

Yellow solid, 10.6 mg (54% yield); ^{1}H NMR (600 MHz, $CDCl_3$, mixture of rotamers): δ = 7.06 (d, *J* = 15.3 Hz, 1H), 6.22 (d, *J* = 15.3 Hz, 1H), 3.90 – 3.66 (m, 8H), 1.23 (s, 9H); ^{13}C NMR (150 MHz, $CDCl_3$, mixture of rotamers): δ = 166.2, 157.1, 114.3, 33.8, 28.8 (The morpholine-derived carbon signals did not appear due to the formation of rotamers); HRMS-ESI (m/z) $[M+Na]^+$ calcd for $C_{11}H_{19}NO_2Na$, 220.1308; found: 220.1310.

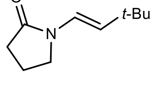

(*E*)-1-(3,3-Dimethylbut-1-en-1-yl)pyrrolidin-2-one (22a)

Colorless oil, 11.3 mg (68% yield); ^{1}H NMR (300 MHz, $CDCl_3$): δ = 6.83 (d, *J* = 14.8 Hz, 1H), 4.97 (d, *J* = 14.8 Hz, 1H), 3.56 – 3.40 (m, 2H), 2.52 – 2.41 (m, 2H), 2.14 – 1.98 (m, 2H), 1.04 (s, 9H).

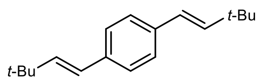

1,4-Bis((*E*)-3,3-dimethylbut-1-en-1-yl)benzene (23a)

Colorless oil, 18.6 mg (77% yield, 2:1 ratio *E*/*Z*); ^{1}H NMR (400 MHz, $CDCl_3$): δ = 7.28 – 7.23 (m, 4H), 7.14 – 7.07 (m, 2H), 6.36 (d, *J* = 12.6 Hz, 1H), 6.27 – 6.22 (m, 4H), 5.57 (d, *J* = 12.6 Hz, 1H), 1.10 (s, 18H), 0.98 (s, 9H).

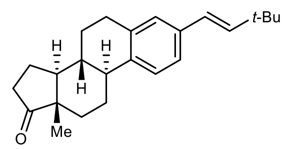

(8R,9S,13S,14S)-3-((*E*)-3,3-Dimethylbut-1-en-1-yl)-13-methyl-6,7,8,9,11,12,13,14,15,16-decahydro-17H-cyclopenta[a]phenanthren-17-one (24a)

Colorless oil, 23.6 mg (70% yield); ^{1}H NMR (400 MHz, $CDCl_3$): δ = 7.30 – 7.22 (m, 1H), 7.22 – 7.16 (m, 1H), 7.13 (s, 1H), 6.34 – 6.18 (m, 2H), 2.93 (dd, *J* = 9.1, 4.2 Hz, 2H), 2.53 (dd, *J* = 18.8, 8.7 Hz, 1H), 2.44 (dd, *J* = 9.9, 4.7 Hz, 1H), 2.32 (dt, *J* = 14.3, 7.3 Hz, 1H), 2.25 – 1.92 (m, 4H), 1.73 – 1.41 (m, 6H), 1.14 (s, 9H), 0.94 (s, 3H); ^{13}C NMR (100 MHz, $CDCl_3$): δ = 220.9, 141.4, 138.4, 136.5, 135.7, 126.6, 125.5, 124.2, 123.5, 50.5, 48.0, 44.4, 38.3, 35.9, 33.3, 31.6, 29.6, 29.4, 26.6, 25.8, 21.6, 13.9; HRMS-ESI (m/z) [M+Na]$^{+}$ calcd for $C_{24}H_{32}ONa$, 359.2345; found: 359.2348.

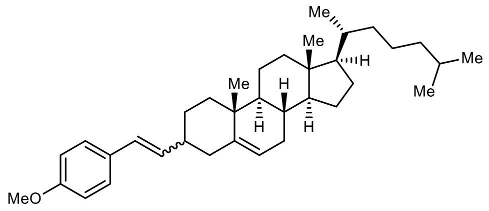

(8S,9S,10R,13R,14S,17R)-3-(4-Methoxystyryl)-10,13-dimethyl-17-((R)-6-methylheptan-2-yl)-2,3,4,7,8,9,10,11,12,13,14,15,16,17-tetradecahydro-1H-cyclopenta[a]phenanthrene (3ac)

White solid, 41.5 mg (80% yield, 3:4 ratio of stereoisomers); ^{1}H NMR (300 MHz, $CDCl_3$, shown only for the major isomer): δ = 7.29 – 7.22 (m, 2H), 6.81 (d, *J* = 8.4 Hz, 2H), 6.33 (d, *J* = 16.0 Hz, 1H), 6.18 (dd, *J* = 16.0, 7.1 Hz, 1H), 5.32 (t, *J* = 4.9 Hz, 2H), 3.78 (s, 3H), 2.60 (d, *J* = 10.9 Hz, 2H), 2.15 – 1.79 (m, 4H), 1.70 – 0.79 (m, 35H), 0.68 (s, 3H).

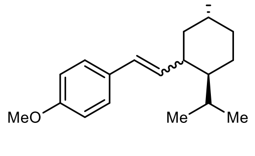

1-(2-((2S,5R)-2-isopropyl-5-methylcyclohexyl)vinyl)-4-methoxybenzene (3ad)

White solid, 18.4 mg (68% yield, 1:2 ratio of stereoisomers, calibrated to the minor isomer); ^{1}H NMR (400 MHz, $CDCl_3$): δ = 7.39 – 7.29 (m, 6H), 6.94 – 6.83 (m, 6H), 6.42 – 6.28 (m, 4H), 5.96 – 5.85 (m, 2H), 3.84 (d, *J* = 1.3 Hz, 9H), 2.81 – 2.70 (m, 1H), 2.16 – 2.02 (m, 2H), 1.99 – 1.89 (m, 2H), 1.87 – 1.61 (m, 10H), 1.52 – 1.23 (m, 6H), 1.18 – 0.86 (m, 30H), 0.81 – 0.76 (m, 6H); ^{13}C NMR (100 MHz, $CDCl_3$, mixture of isomers): δ = 158.7, 158.6, 133.9, 131.1, 130.9, 129.5, 129.4, 128.0, 127.1, 127.0, 113.9, 113.9, 55.3, 47.6, 47.5, 45.2, 43.3, 43.2, 40.7, 35.9, 35.2, 32.5, 30.5, 28.3, 27.0, 25.7, 24.3, 22.9, 22.7, 21.5, 21.1, 20.8, 15.5; HRMS-EI (m/z) [M]$^{+}$ calcd for

$C_{19}H_{28}O$, 272.2410; found: 272.2410.

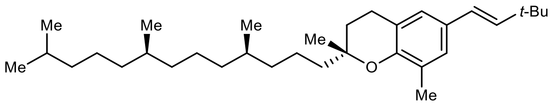

(R)-6-((*E*)-3,3-Dimethylbut-1-en-1-yl)-2,8-dimethyl-2-((4R,8R)-4,8,12-trimethyltridecyl)chromane (25a)

Yellow oil, 30.8 mg (66% yield); ^{1}H NMR (300 MHz, $CDCl_3$): δ = 6.98 (d, *J* = 2.2 Hz, 1H), 6.89 (d, *J* = 2.3 Hz, 1H), 6.17 (d, *J* = 16.2 Hz, 1H), 6.12 – 5.99 (m, 1H), 2.71 (t, *J* = 6.8 Hz, 2H), 2.14 (s, 3H), 1.87 – 1.63 (m, 2H), 1.64 – 1.41 (m, 4H), 1.41 – 0.99 (m, 26H), 0.90 – 0.75 (m, 15H); ^{13}C NMR (150 MHz, $CDCl_3$): δ = 151.3, 138.8, 128.8, 126.3, 125.9, 124.5, 124.2, 120.4, 76.0, 39.9, 39.4, 37.4, 37.3, 33.1, 32.8, 32.7, 31.4, 29.7, 28.0, 24.8, 24.4, 24.2, 22.7, 22.6, 22.3, 21.0, 19.7, 19.6, 16.1; HRMS-ESI (m/z) $[M+Na]^+$ calcd for $C_{33}H_{56}ONa$, 469.4404; found: 469.4408.

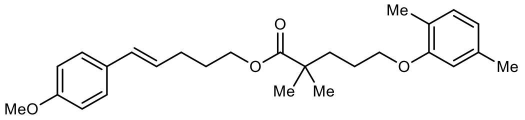

(*E*)-5-(4-Methoxyphenyl)pent-4-en-1-yl 5-(2,5-dimethylphenoxy)-2,2-dimethylpentanoate (3ae)

Yellow oil, 28.2 mg (75% yield); ^{1}H NMR (600 MHz, $CDCl_3$): δ = 7.29 – 7.21 (m, 2H), 7.00 (dd, *J* = 7.5, 2.7 Hz, 1H), 6.86 – 6.80 (m, 2H), 6.65 (d, *J* = 7.5 Hz, 1H), 6.61 (d, *J* = 3.1 Hz, 1H), 6.34 (d, *J* = 15.7 Hz, 1H), 6.05 (dt, *J* = 15.8, 7.0 Hz, 1H), 4.11 (t, *J* = 6.5 Hz, 2H), 3.92 (t, *J* = 5.6 Hz, 2H), 3.79 (s, 3H), 2.30 (s, 3H), 2.29 – 2.25 (m, 2H), 2.18 (s, 3H), 1.83 – 1.77 (m, 2H), 1.75 – 1.72 (m, 4H), 1.23 (s, 6H). ^{13}C NMR (150 MHz, $CDCl_3$): δ = 177.8, 158.8, 156.9, 136.4, 130.3, 130.3, 130.1, 127.1, 127.1, 123.6, 120.7, 113.9, 111.9, 67.9, 63.8, 55.3, 42.1, 37.1, 29.4, 28.5, 25.2, 21.4, 15.8; HRMS-ESI (m/z) $[M+Na]^+$ calcd for $C_{27}H_{36}O_4Na$, 447.2506; found: 447.2507.

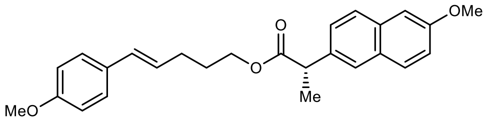

(*E*)-5-(4-Methoxyphenyl)pent-4-en-1-yl (S)-2-(6-methoxynaphthalen-2-yl)propanoate (3af)

Yellow oil, 28.2 mg (70% yield, 1:1.3 ratio of stereoisomers); ^{1}H NMR (600 MHz, $CDCl_3$, only the major isomer indicated): δ = 8.16 – 8.05 (m, 3H), 7.82 (dd, *J* = 26.1, 8.4 Hz, 1H), 7.62 – 7.49 (m, 4H), 7.23 (ddd, *J* = 17.6, 8.7, 1.8 Hz, 2H), 6.56 (d, *J* = 15.8 Hz, 1H), 6.36 (dt, *J* = 15.0, 6.9 Hz, 1H), 4.53 (q, *J* = 6.9 Hz, 2H), 4.32 (s, 3H), 4.28 (q, *J* = 7.2 Hz, 1H), 4.20 (s, 3H), 2.54 (q, *J* = 7.3 Hz, 2H), 2.14 (p, *J* = 7.2 Hz, 2H), 2.00 (d, *J* = 7.2 Hz, 3H); ^{13}C NMR (150 MHz, $CDCl_3$, mixture of stereoisomers, overlapping peaks not repeated): δ = 175.2, 158.1, 134.1, 130.5, 130.3, 129.7, 129.5, 129.4, 127.5, 126.7, 126.4, 119.4, 114.3, 114.0, 106.0, 64.6, 55.7, 46.0, 29.6, 29.3, 28.8, 25.4, 18.9.; HRMS-ESI (m/z) $[M+Na]^+$ calcd for $C_{26}H_{28}O_4Na$, 427.1880; found: 427.1882.

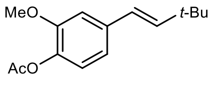

(*E*)-4-(3,3-Dimethylbut-1-en-1-yl)-2-methoxyphenyl acetate (26a)

Yellow oil, 23.7 mg (95% yield); ^{1}H NMR (600 MHz, $CDCl_3$): δ = 6.97 – 6.86 (m, 3H), 6.26 (d, *J* = 16.1 Hz, 1H), 6.19 (d, *J* = 16.1 Hz, 1H), 3.84 (s, 3H), 2.30 (s, 3H), 1.11 (s, 9H); ^{13}C NMR (150 MHz, $CDCl_3$): δ = 169.2, 151.0, 142.2, 138.5, 137.2, 124.0, 122.6, 118.5, 109.7, 55.8, 55.8, 33.4, 29.6, 20.7, 20.7; HRMS-ESI (m/z)

$[M+Na]^+$ calcd for $C_{15}H_{20}O_3Na$, 271.1305; found: 271.1306.

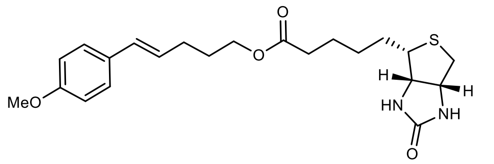

(E)-5-(4-Methoxyphenyl)pent-4-en-1-yl 5-((3aS,4S,6aR)-2-oxohexahydro-1H-thieno[3,4-d]imidazol-4-yl)pentanoate (3ag)

Yellow oil, 34.0 mg (81% yield); ^1H NMR (600 MHz, $CDCl_3$) δ 7.25 (d, *J* = 8.5 Hz, 2H), 6.82 (d, *J* = 8.4 Hz, 2H), 6.33 (d, *J* = 15.8 Hz, 1H), 6.03 (dt, *J* = 15.8, 6.9 Hz, 1H), 5.82 (brs, 1H), 5.43 (brs, 1H), 4.47 (t, *J* = 6.3 Hz, 1H), 4.34 – 4.23 (m, 1H), 4.10 (t, *J* = 6.6 Hz, 2H), 3.78 (s, 3H), 3.12 (brs, 1H), 2.88 (dd, *J* = 13.1, 4.8 Hz, 1H), 2.72 (d, *J* = 12.8 Hz, 1H), 2.32 (t, *J* = 7.5 Hz, 2H), 2.24 (q, *J* = 7.4 Hz, 2H), 1.78 (p, *J* = 6.8 Hz, 2H), 1.69 – 1.59 (m, 4H), 1.46 – 1.38 (m, 2H); ^{13}C NMR (150 MHz, $CDCl_3$) δ 173.9, 164.0, 158.9, 130.5, 130.1, 127.2, 127.2, 114.0, 64.0, 62.1, 60.3, 55.5, 55.4, 40.7, 34.1, 29.5, 28.5, 28.5, 28.4, 24.9. HRMS-ESI (m/z) $[M+Na]^+$ calcd for $C_{22}H_{30}N_2O_4SNa$, 441.1818; found: 441.1820.

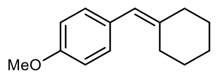

1-(Cyclohexylidenemethyl)-4-methoxybenzene (3ah)

Yellow oil, 12.2 mg (60% yield); ^1H NMR (600 MHz, $CDCl_3$): δ = 7.12 (d, *J* = 8.3 Hz, 2H), 6.84 (d, *J* = 8.4 Hz, 2H), 6.15 (s, 1H), 3.79 (s, 3H), 2.34 (t, *J* = 6.1 Hz, 2H), 2.22 (t, *J* = 5.9 Hz, 2H), 1.63 – 1.52 (m, 6H).

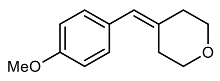

4-(4-Methoxybenzylidene)tetrahydro-2H-pyran (3ai)

Colorless oil, 14.4 mg (70% yield); ^1H NMR (400 MHz, $CDCl_3$): δ = 7.19 – 7.12 (m, 2H), 6.89 (d, *J* = 8.8 Hz, 2H), 6.29 (s, 1H), 3.83 (s, 3H), 3.82 – 3.78 (m, 2H), 3.68 (t, *J* = 5.6 Hz, 2H), 2.57 – 2.51 (m, 2H), 2.42 – 2.37 (m, 2H); ^{13}C NMR (150 MHz, $CDCl_3$) δ 158.0, 136.4, 130.0, 127.2, 123.3, 113.6, 69.5, 68.6, 55.3, 37.2, 30.6; HRMS-ESI (m/z) $[M]^+$ calcd for $C_{13}H_{16}O_2$, 204.1150; found: 204.1152.

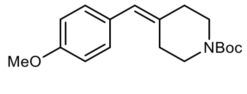

***tert*-Butyl 4-(4-methoxybenzylidene)piperidine-1-carboxylate (3aj)**

Yellow oil, 26.1 mg (86% yield); ^1H NMR (300 MHz, $CDCl_3$): δ = 7.17 – 7.03 (m, 2H), 6.84 (d, *J* = 8.7 Hz, 2H), 6.27 (s, 1H), 3.78 (s, 3H), 3.47 (t, *J* = 6.0 Hz, 2H), 3.37 (t, *J* = 5.9 Hz, 2H), 2.43 (t, *J* = 5.9 Hz, 2H), 2.29 (t, *J* = 5.9 Hz, 2H), 1.45 (s, 9H).

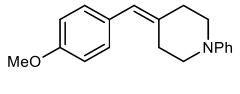

4-(4-Methoxybenzylidene)-1-phenylpiperidine (3ak)

White solid, 20.9 mg (75% yield); ^{1}H NMR (300 MHz, $CDCl_3$): δ = 7.39 – 7.26 (m, 2H), 7.24 – 7.16 (m, 2H), 7.07 – 6.96 (br, 2H), 6.96 – 6.90 (m, 3H), 6.35 (s, 1H), 3.85 (s, 3H), 3.38 (t, *J* = 5.8 Hz, 2H), 3.27 (t, *J* = 5.8 Hz, 2H), 2.70 (br, 2H), 2.55 (br, 2H); ^{13}C NMR (150 MHz, $CDCl_3$, mixture of rotamers) δ 158.0, 151.5, 137.8, 130.2, 130.1, 129.2, 123.3, 120.2, 116.6, 113.6, 55.3, 51.5, 50.7, 35.9, 28.9; HRMS-ESI (m/z) $[M]^+$ calcd for $C_{19}H_{21}NO$, 279.1623; found: 279.1622.

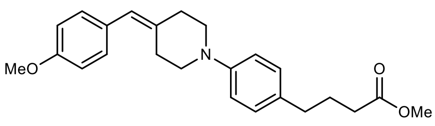

Methyl 4-(4-(4-(4-methoxybenzylidene)piperidin-1-yl)phenyl)butanoate (3al)

Yellow oil, 28.7 mg (76% yield); ^{1}H NMR (400 MHz, $CDCl_3$): δ = 7.18 – 7.10 (m, 2H), 7.06 (d, *J* = 8.5 Hz, 2H), 6.86 (d, *J* = 8.7 Hz, 4H), 6.29 (s, 1H), 3.80 (s, 3H), 3.65 (s, 3H), 3.27 (t, *J* = 5.8 Hz, 2H), 3.16 (t, *J* = 5.8 Hz, 2H), 2.63 (t, *J* = 5.7 Hz, 2H), 2.56 (t, *J* = 7.5 Hz, 2H), 2.54 – 2.43 (m, 2H), 2.31 (t, *J* = 7.5 Hz, 2H), 2.00 – 1.82 (m, 2H); ^{13}C NMR (150 MHz, $CDCl_3$, mixture of rotamers, due to severe peak broadening, only tentatively assigned peaks are indicated): δ = 174.1, 158.0, 149.7, 138.2, 132.7, 130.0, 128.6, 123.2, 116.9, 113.6, 112.2, 55.3, 51.9, 51.5, 51.1, 36.0, 34.2, 33.1, 28.9, 26.6; HRMS-ESI (m/z) $[M+H]^+$ calcd for $C_{24}H_{30}NO_3$, 380.2220; found: 380.2223.

5. *E*/*Z* isomerization experiment

An isolated mixture of **18a** (*E*/*Z* = 1:2) was subjected to the original reaction condition to probe the origin of the formation of the *Z* product. The *E*/*Z* ratio and product recovery was measured by ^{1}H NMR using nitrobenzene as an internal standard.

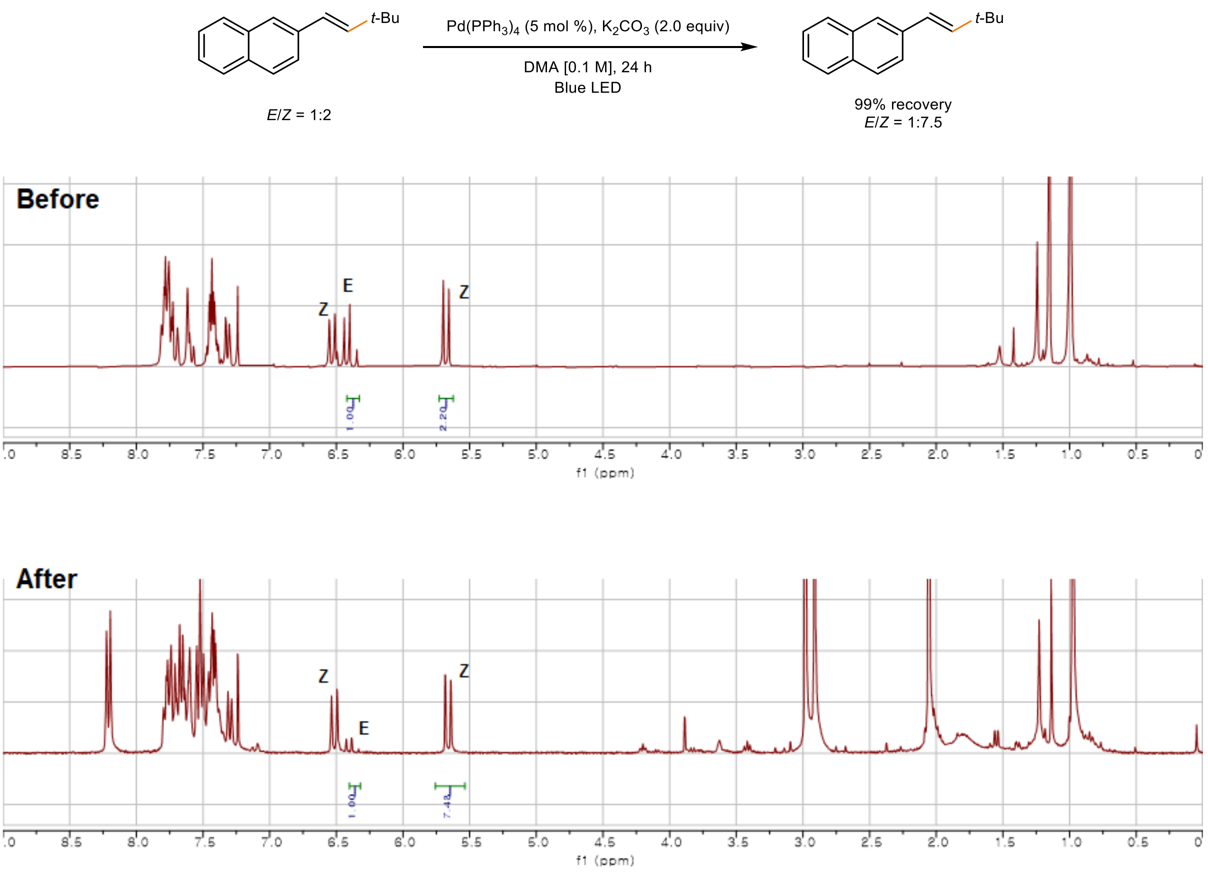

Supplementary Fig. 1. *E*/*Z* isomerization experiment.

By comparing the NMR spectra before and after irradiation, we confirmed that isomerization occurs after product formation. Similar visible-light mediated styrene *E*/*Z* isomerization reactions have been reported.[26-29]

6. Radical trapping/clock experiments

TEMPO trapping experiment

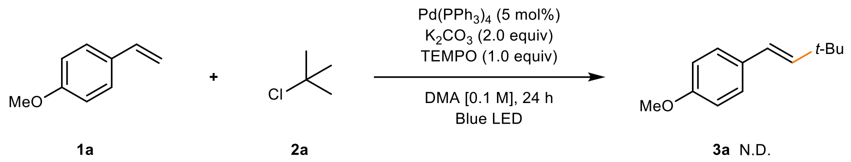

To a 4 mL vial equipped with a stirrer-bar were added $Pd(PPh_3)_4$ (5.8 mg, 0.0050 mmol, 0.050 equiv), K_2CO_3 (27.6 mg, 0.20 mmol, 2.0 equiv), **1a** (13.3 µL, 0.10 mmol, 1.0 equiv), **2a** (16.5 µL, 0.15 mmol, 1.5 equiv), (2,2,6,6-tetramethylpiperidin-1-yl)oxyl (TEMPO, 15.6 mg, 0.10 mmol, 1.0 equiv) and *N*,*N*-dimethylacetamide (1.0 mL). The resulting mixture was stirred for 24 h under 40 W blue LED irradiation with fan cooling (~30 ºC). The resulting residue was analyzed by gas chromatography after the addition of dodecane (10.0 µL) as an internal standard and revealed no presence of **3a**.

Radical Clock Experiment

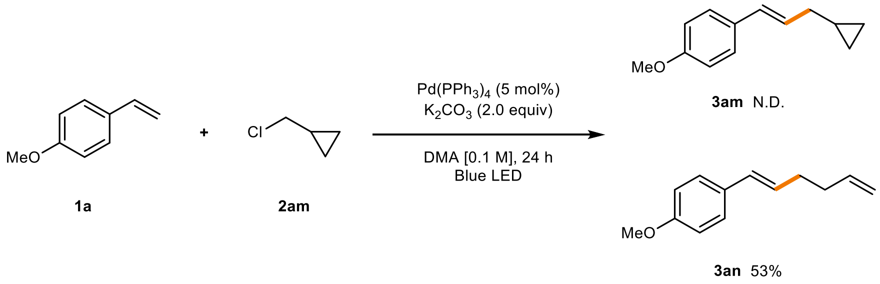

To a 4 mL vial equipped with a stirrer-bar were added $Pd(PPh_3)_4$ (5.8 mg, 0.0050 mmol, 0.050 equiv), K_2CO_3 (27.6 mg, 0.20 mmol, 2.0 equiv), **1a** (13.3 µL, 0.10 mmol, 1.0 equiv), **2am** (13.9 µL, 0.15 mmol, 1.5 equiv) and *N*,*N*-dimethylacetamide (1.0 mL). The resulting mixture was stirred for 24 h under 40 W blue LED irradiation with fan cooling (~30 ºC). The resulting residue was analyzed by ^1H NMR after the addition of nitrobenzene (10.0 µL) as an internal standard. No **3am** was detected and **3an** was generated in 53% yield as the sole product. A crude NMR of the reaction is presented in Supplementary Fig. 2 and all integrated peaks shown below corresponds to the product signals except for the internal standard.

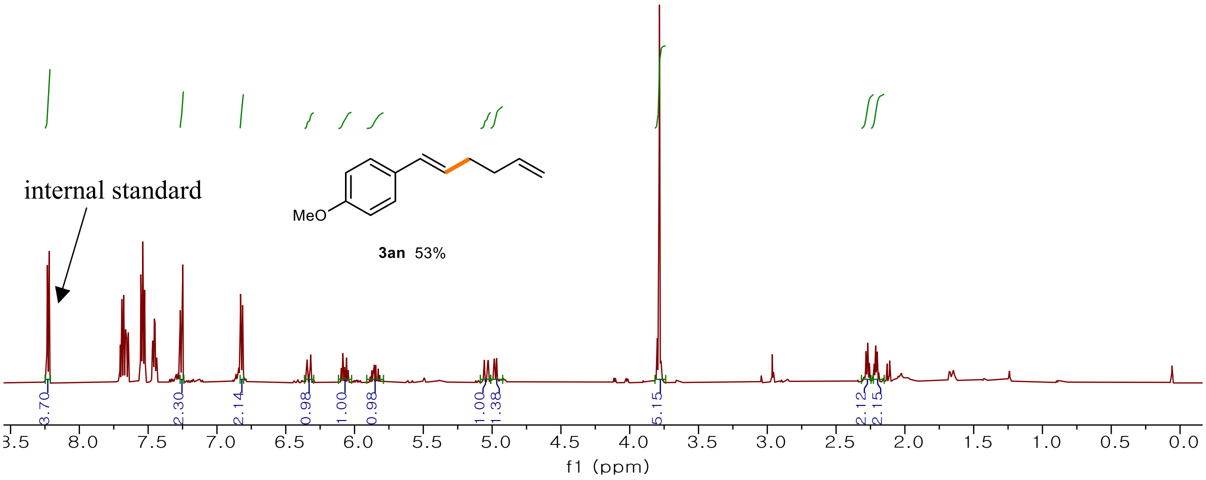

Supplementary Fig. 2. Crude NMR of the radical clock experiment.

7. Stern-Volmer quenching experiments

All emission spectra of the samples were collected under an argon atmosphere. A solution of $Pd(PPh_3)_4$ (1.0 mM) in DMA was prepared in a cuvette. *tert*-Butyl chloride (**2a**) was used as quenchers. The prepared solution was excited at 420 nm and the emission intensity at 650 nm was observed. The Stern-Volmer quenching experiments resulted in positive dependence between I_0/I and the concentration of quenchers. The results indicate that alkyl chlorides quench the excited state of $Pd(PPh_3)_4$, where it engages in a single-electron transfer (SET) event with the photoexcited Pd(0) complex.

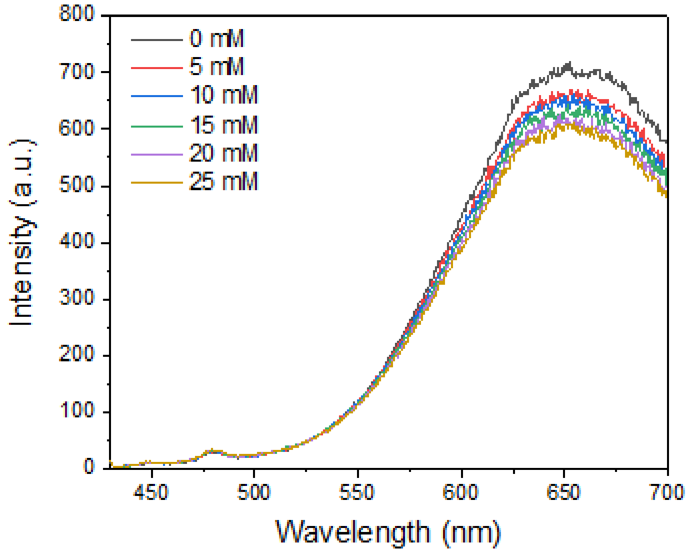

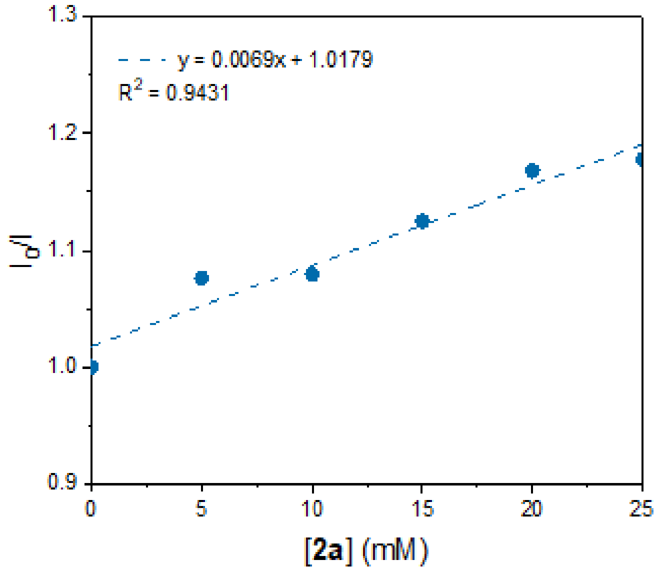

Supplementary Fig. 3. Stern-Volmer quenching experiment.

8. Kinetic isotope effect measurements

Preparation of 3a-β-d_2

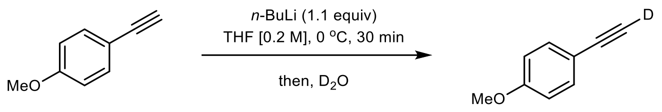

To a stirred solution of 4-methoxyphenylacetylene (132 mg, 1.0 mmol, 1.0 equiv) in THF (5.0 mL) at 0 °C was added *n*-BuLi (0.70 mL, 1.6 M in hexanes, 1.1 mmol, 1.1 equiv) dropwise. The reaction mixture was quenched with D_2O (0.20 mL) after 30 minutes and further stirred at room temperature for 5 minutes. The resulting solution was dried ($MgSO_4$), filtered and concentrated under reduced pressure to yield 4-methoxyphenylacetylene-d_1 (132 mg, 100% yield) as a colorless oil, which was used directly without further purification. The deuterium incorporation was determined to be 98% by ^1H NMR. The NMR spectrum was identical with the starting material with the acetylene proton being absent.

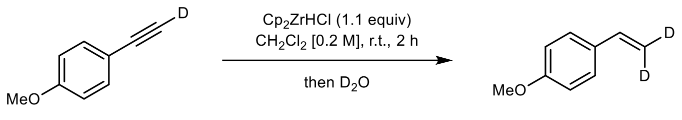

1a-β-d_2

To a stirred solution of 4-methoxyphenylacetylene-d_1 (132 mg, 1.0 mmol, 1.0 equiv) in CH_2Cl_2 (5.0 mL) at 0 °C was added Cp_2ZrHCl (273 mg, 1.1 mmol, 1.1 equiv). The resulting mixture was warmed to room temperature and stirred for 2 h before it was quenched with D_2O (0.20 mL) and stirred for further 30 min. The resulting solution was dried ($MgSO_4$), filtered and concentrated under reduced pressure. The resulting residue was purified by column chromatography (silica gel, hexanes:EtOAc gradient elution) to yield **1a-β-d_2** (130 mg, 99% yield) as a colorless oil. The deuterium incorporation was determined to be 95% by ^1H NMR. The NMR spectrum was identical with the starting material **1a** with the olefinic proton being absent. ^1H NMR (300 MHz, $CDCl_3$): $\delta = 7.39 - 7.29$ (m, 2H), 6.92 – 6.80 (m, 2H), 6.63 (s, 1H), 3.79 (s, 3H). *Residual H signal: 5.57 (d, $J = 17.6$ Hz, 0.06 H), 5.09 (d, $J = 10.9$ Hz, 0.05 H).

Preparation of 2n-d_1

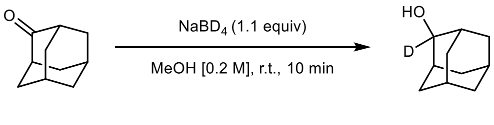

To a stirred solution of 2-adamantanone (285 mg, 1.9 mmol, 1.0 equiv) in MeOH (10 mL) at room temperature was added $NaBD_4$ (88 mg, 2.1 mmol, 1.1 equiv). The reaction mixture stirred for 10 min before it was quenched with NH_4Cl (sat. aq., 10 mL). The resulting mixture was extracted with CH_2Cl_2 (10 mL × 3), dried (anhydrous Na_2SO_4), filtered, and concentrated under reduced pressure. The resulting material was used directly for the next step.

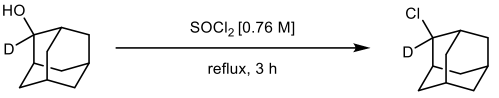

2n-*d*$_1$

To a stirred solution of thionyl chloride (2.5 mL) was added the crude mixture of 2-adamantanol (291 mg, 1.9 mmol, 1.0 equiv). The reaction mixture was stirred for 3 h at 75 °C before it was quenched with MeOH (5 mL) and $NaHCO_3$ (sat. aq., 10 mL) in a cooling bath. The resulting mixture was extracted with CH_2Cl_2 (10 mL × 3), dried (anhydrous Na_2SO_4), filtered, and concentrated under reduced pressure. The resulting residue was purified by column chromatography (silica gel, hexanes) to afford the desired product **2n-*d*$_1$** (260 mg, 80% over two steps). The deuterium incorporation was determined to be >99% by ^{1}H NMR. ^{1}H NMR (600 MHz, $CDCl_3$) δ = 2.26 (d, *J* = 12.2 Hz, 2H), 2.07 (brs, 2H), 1.97 – 1.92 (m, 2H), 1.88 – 1.84 (m, 2H), 1.81 – 1.77 (m, 2H), 1.75 (m, 2H), 1.59 – 1.55 (m, 2H).

Preparation of 3a-β-*d*$_1$

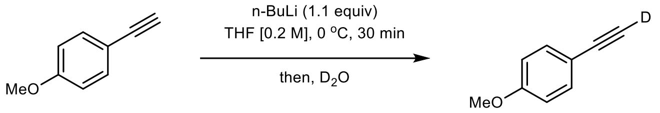

To a stirred solution of 4-methoxyphenylacetylene (132 mg, 1.0 mmol, 1.0 equiv) in THF (5.0 mL) at 0 °C was added n-BuLi (0.70 mL, 1.6 M in hexanes, 1.1 mmol, 1.1 equiv) dropwise. The reaction mixture was quenched with D_2O (0.20 mL) after 30 minutes and further stirred at room temperature for 5 minutes. The resulting solution was dried ($MgSO_4$), filtered and concentrated under reduced pressure to yield 4-methoxyphenylacetylene-*d*$_1$ (132 mg, 100% yield) as a colorless oil, which was used directly without further purification. The deuterium incorporation was determined to be 98% by ^{1}H NMR. The NMR spectrum was identical with the starting material with the acetylene proton being absent.

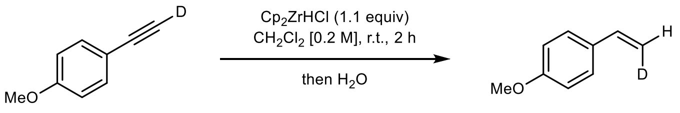

1a-β-*d*$_1$

To a stirred solution of 4-methoxyphenylacetylene-*d*$_1$ (132 mg, 1.0 mmol, 1.0 equiv) in CH_2Cl_2 (5.0 mL) at 0 °C was added Cp_2ZrHCl (273 mg, 1.1 mmol, 1.1 equiv). The resulting mixture was warmed to room temperature and stirred for 2 h before it was quenched with H_2O (0.20 mL) and stirred for further 30 min. The resulting solution was dried ($MgSO_4$), filtered and concentrated under reduced pressure. The resulting residue was purified by column chromatography (silica gel, hexanes:EtOAc gradient elution) to yield **1a-β-*d*$_1$** (130 mg, 99% yield) as a colorless oil. The deuterium incorporation was determined to be 98% by ^{1}H NMR. The NMR spectrum was identical with the starting material **1a** with the olefinic proton being absent. ^{1}H NMR (300 MHz, $CDCl_3$): δ = 7.37 – 7.29 (m, 2H), 6.88 – 6.81 (m, 2H), 6.63 (dt, *J* = 10.9, 2.6 Hz, 1H), 5.09 (d, *J* = 10.9 Hz, 1H), 3.79 (s, 4H). *Residual H signal: 5.59 (dd, *J* = 17.7 Hz, 1.1 Hz, 0.02 H).

Preparation of 27-d_2

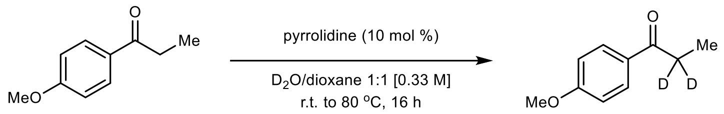

To a stirred solution of 4'-methoxypropiophenone (164 mg, 1.0 mmol, 1.0 equiv) in D_2O (1.5 mL) and dioxane (1.5 mL) at room temperature was added pyrrolidine (7.1 mg, 0.10 mmol, 0.10 equiv). The reaction mixture was heated to 80 °C and stirred for 16 h. The reaction mixture was quenched with water (5 mL), extracted with Et_2O (10 mL), washed with brine (10 mL), dried (anhydrous Na_2SO_4), filtered, and concentrated under reduced pressure to afford 1-(4-methoxyphenyl)propan-1-one-2,2-d_2 (160 mg, 99% yield) as a colorless oil which was used directly for the next step. The deuterium incorporation was determined to be 98.5% by ^{1}H NMR. ^{1}H NMR (300 MHz, $CDCl_3$): δ = 7.99 – 7.88 (m, 2H), 6.97 – 6.81 (m, 2H), 3.84 (d, J = 0.7 Hz, 3H), 1.17 (s, 3H). Residual proton signal: 2.90 (s, 0.03H).

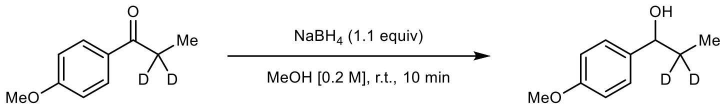

To a stirred solution of 1-(4-methoxyphenyl)propan-1-one-2,2-d_2 (160 mg, 0.99 mmol, 1.0 equiv) in MeOH (5.0 mL) at room temperature was added $NaBH_4$ (41.6 mg, 1.1 mmol, 1.1 equiv). The reaction mixture stirred for 10 min before it was quenched with NH_4Cl (sat. aq., 10 mL). The resulting mixture was extracted with CH_2Cl_2 (10 mL × 3), dried (anhydrous Na_2SO_4), filtered, and concentrated under reduced pressure. The resulting residue was purified by column chromatography (silica gel hexanes:EtOAc gradient elution) to afford 1-(4-methoxyphenyl)propan-2,2-d_2-1-ol (145 mg, 86% yield) as a colorless oil. ^{1}H NMR (300 MHz, $CDCl_3$): δ = 7.24 (d, J = 8.8 Hz, 2H), 6.86 (d, J = 8.7 Hz, 2H), 4.51 (s, 1H), 3.78 (s, 3H).

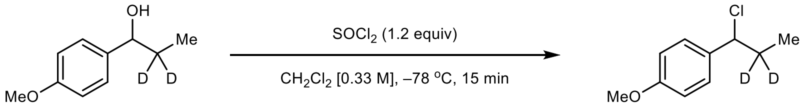

To a stirred solution of 1-(4-methoxyphenyl)propan-1-one-2,2-d_2 (145 mg, 0.86 mmol, 1.0 equiv) in MeOH (3.0 mL) at –78 °C was added $SOCl_2$ (75 µL, 1.03 mmol, 1.2 equiv) dropwise. The reaction mixture stirred for 15 min before it was quenched with water (10 mL). The resulting mixture was extracted with CH_2Cl_2 (10 mL × 3), dried (anhydrous Na_2SO_4), filtered, and concentrated under reduced pressure. The resulting residue was pure enough and unstable upon silica gel column chromatography and was used directly for the kinetic study. ^{1}H NMR (300 MHz, $CDCl_3$): δ = 7.31 – 7.24 (m, 2H), 6.89 – 6.82 (m, 2H), 4.74 (s, 1H), 3.79 (s, 3H).

KIE measurement with 1a-β-d_2

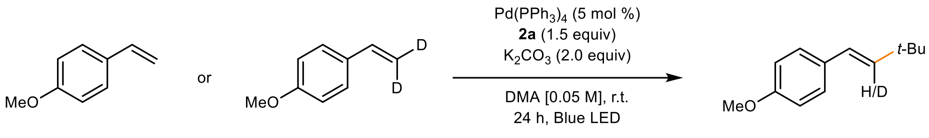

To two 4 mL vial equipped with a stirrer-bar were added $Pd(PPh_3)_4$ (5.8 mg, 0.0050 mmol, 0.050 equiv), K_2CO_3 (27.6 mg, 0.20 mmol, 2.0 equiv), dodecane (10.0 µL), **2a** (16.5 µL, 0.15 mmol, 1.5 equiv), **1a** or **1a-β-d_2** respectively (13.3 µL, 0.10 mmol, 1.0 equiv) and *N*,*N*-dimethylacetamide (2.0 mL). The resulting mixture was kept stirring under 40 W blue LED irradiation and aliquots of both reaction mixtures were taken every 10 minutes. They were analyzed by gas chromatography to determine the individual reaction rates of **1a** and **1a-β-d_2**.

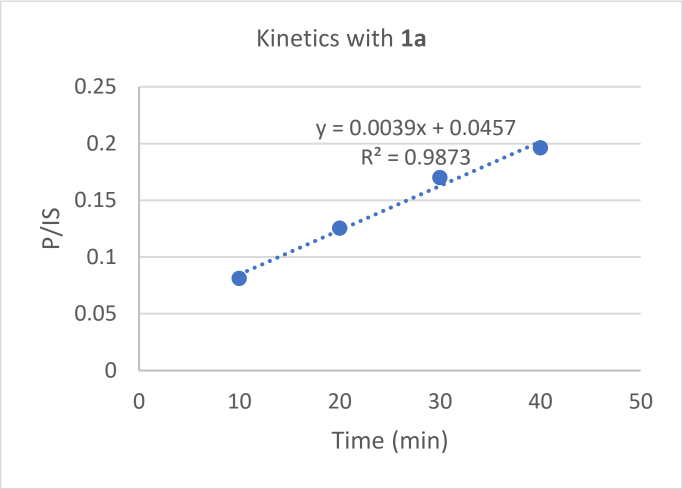

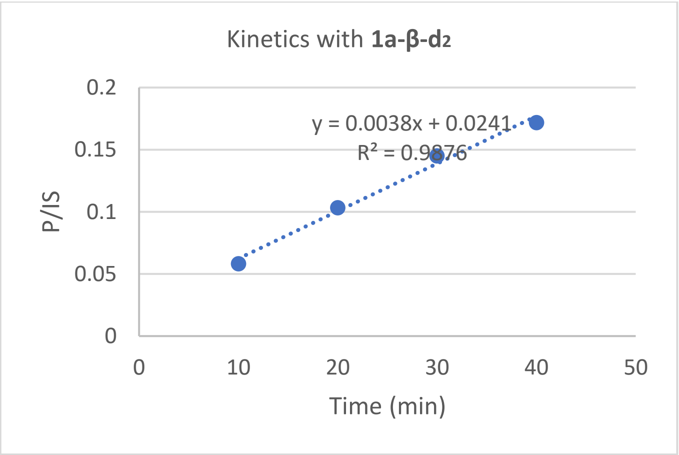

Supplementary Fig. 4. KIE measurement with 1a-β-d_2.

The KIE was determined to be 1.0.

KIE measurement with 3a-α-d_1

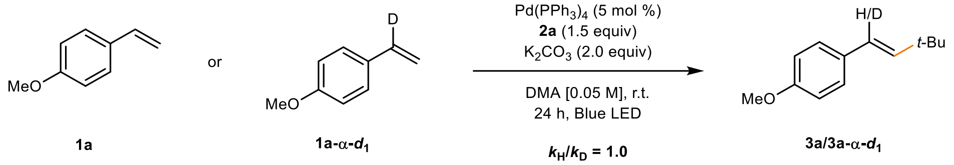

Substrate **1a-α-*d*₁** was prepared following literature procedures.[30] To two 4 mL vial equipped with a stirrer-bar were added $Pd(PPh_3)_4$ (5.8 mg, 0.0050 mmol, 0.050 equiv), K_2CO_3 (27.6 mg, 0.20 mmol, 2.0 equiv), dodecane (10.0 µL), **2a** (16.5 µL, 0.15 mmol, 1.5 equiv), **1a** or **1a-α-*d*₁** respectively (13.3 µL, 0.10 mmol, 1.0 equiv) and *N*,*N*-dimethylacetamide (2.0 mL). The resulting mixture was kept stirring under 40 W blue LED irradiation and aliquots of both reaction mixtures were taken every 10 minutes. They were analyzed by gas chromatography to determine the individual reaction rates of **1a** and **1a-α-*d*₁**.

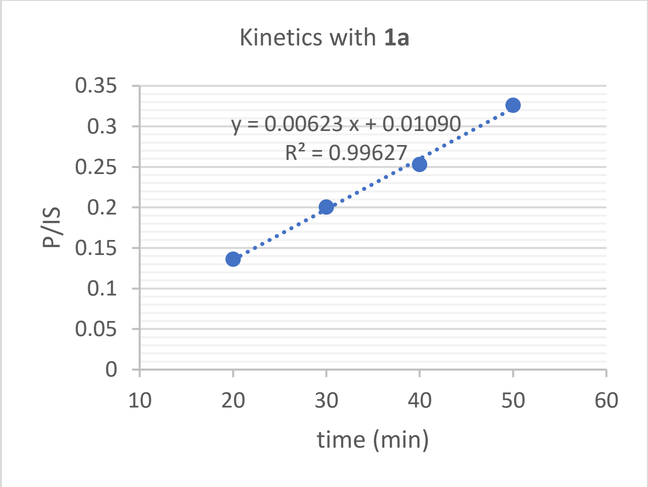

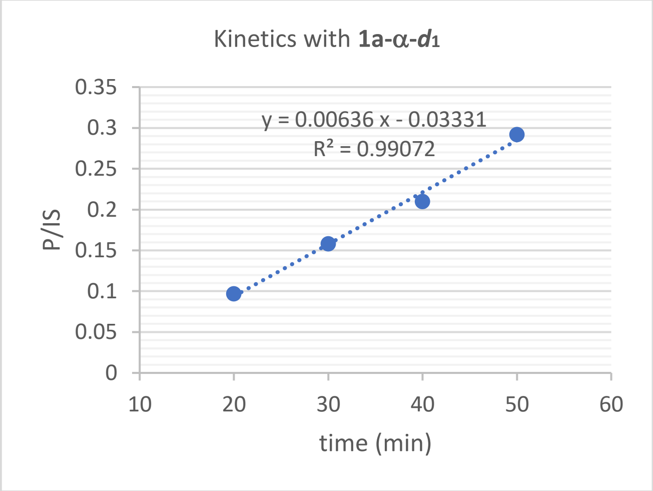

Supplementary Fig. 5. KIE measurement with 1a-α-*d*₁.

The KIE was determined to be 1.0.

KIE measurement with 2n-*d*1

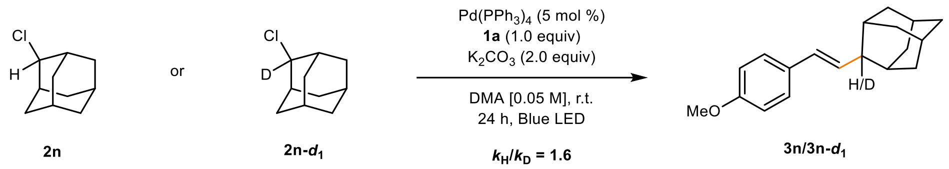

To two 4 mL vial equipped with a stirrer-bar were added $Pd(PPh_3)_4$ (5.8 mg, 0.0050 mmol, 0.050 equiv), K_2CO_3 (27.6 mg, 0.20 mmol, 2.0 equiv), dodecane (10.0 µL), **2n** or **2n-*d*1**, respectively, (25.6 mg, 0.15 mmol, 1.5 equiv), **1a** (13.3 µL, 0.10 mmol, 1.0 equiv) and *N*,*N*-dimethylacetamide (2.0 mL). The resulting mixture was kept stirring under 40 W blue LED irradiation and aliquots of both reaction mixtures were taken every 10 minutes. They were analyzed by gas chromatography to determine the individual reaction rates of **1a** and **1a-α-*d*1**.

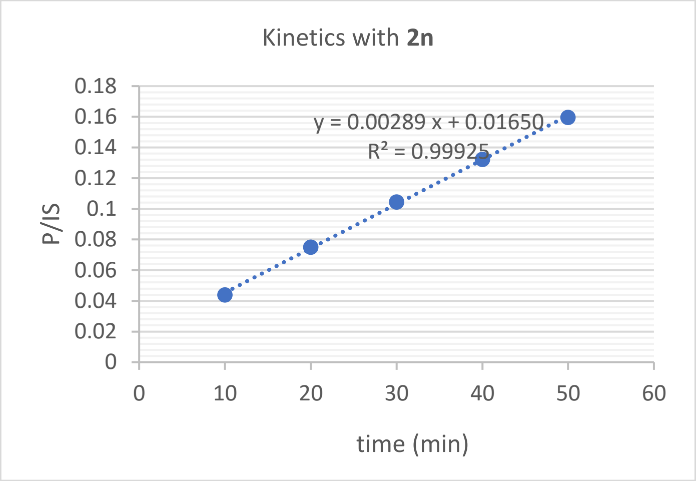

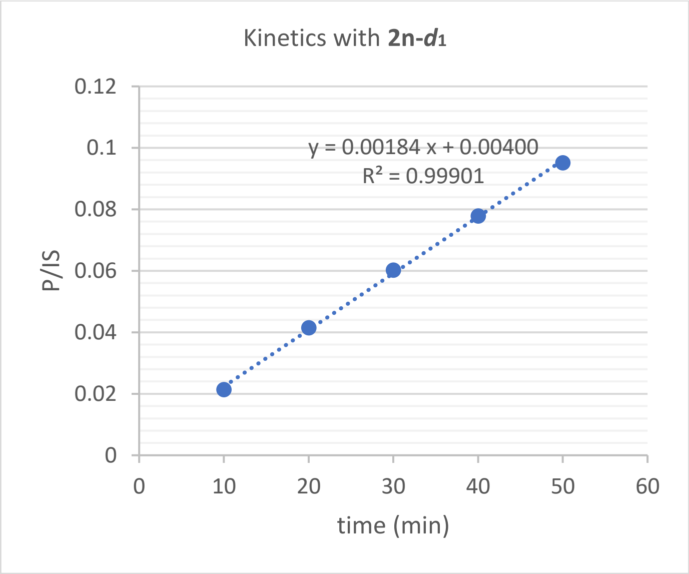

Supplementary Fig. 6. KIE measurement with 2n-*d*1.

The KIE was determined to be 1.6.

Intramolecular competition experiment

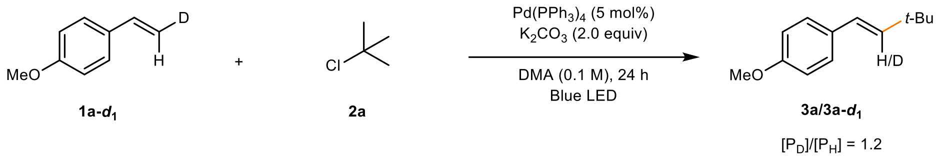

To a 4 mL vial equipped with a stirrer-bar were added $Pd(PPh_3)_4$ (5.8 mg, 0.0050 mmol, 0.050 equiv), K_2CO_3 (27.6 mg, 0.20 mmol, 2.0 equiv), **1a-β-*d*₁** (13.3 µL, 0.10 mmol, 1.0 equiv), **2a** (16.5 µL, 0.15 mmol, 1.5 equiv) and *N*,*N*-dimethylacetamide (1.0 mL). The resulting mixture was stirred for 24 h under 40 W blue LED irradiation with fan cooling (~30 ºC). The reaction mixture was added brine (10 mL), diluted with EtOAc or Et_2O (10 mL), washed with brine (10 mL), dried (anhydrous Na_2SO_4), filtered, and concentrated under reduced pressure. The resulting residue was analyzed by ^1H NMR to determine the ratio of **3a** and **3a-d_1** to be 0.45:0.55 by integrating the olefinic protons ($[P_D]/[P_H]$ = 1.2).

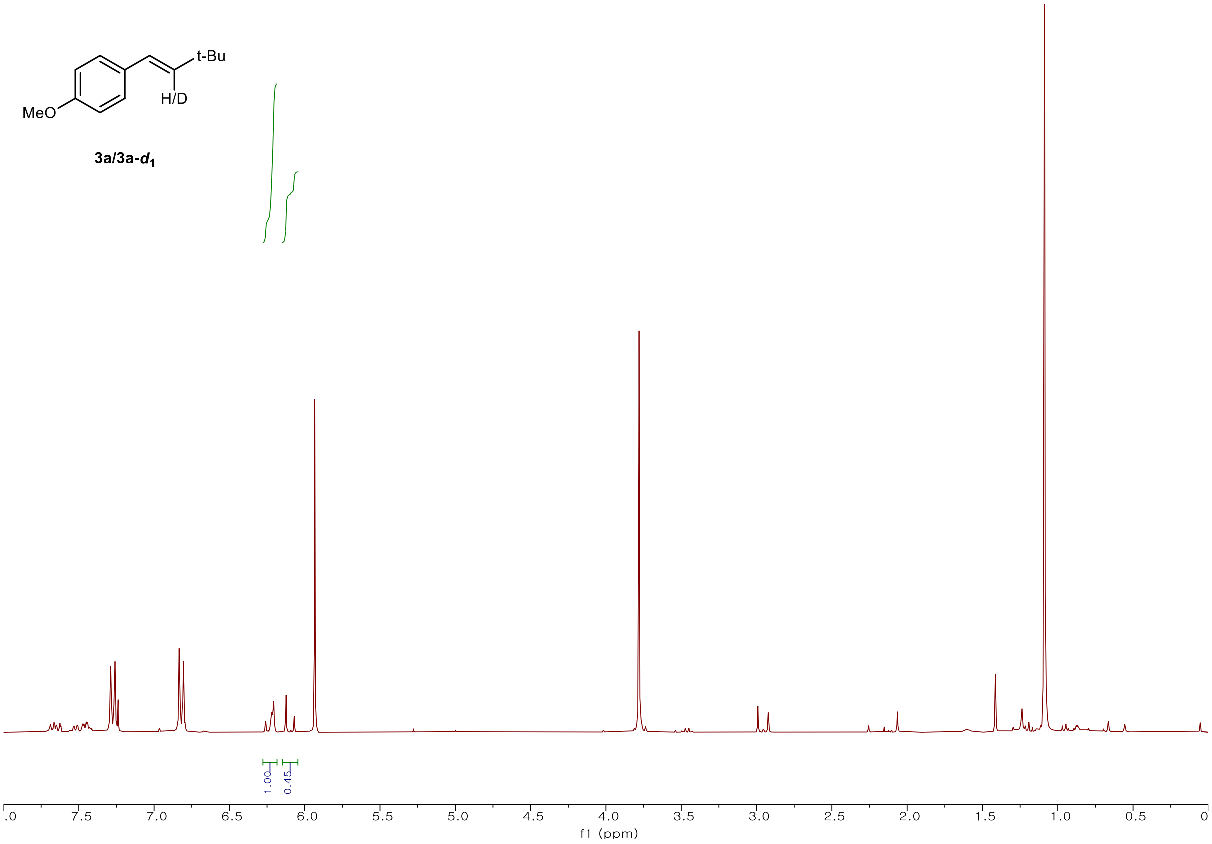

Supplementary Fig. 7. Intramolecular competition experiment.

Elimination kinetic isotope effect experiment

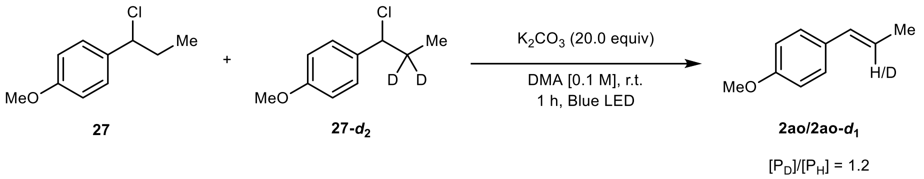

To a 4 mL vial equipped with a stirrer-bar were added **27** and **27-*d*2** (9.3 mg each, 0.10 mmol, 1.0 equiv), K_2CO_3 (276 mg, 2.0 mmol, 20.0 equiv) and *N*,*N*-dimethylacetamide (1.0 mL). The resulting mixture was stirred for 1 h under 40W blue LED irradiation with fan cooling (~30 °C) to ensure identical physical conditions. The reaction mixture was added brine (10 mL), diluted with Et_2O (10 mL), washed with brine (10 mL), dried (anhydrous Na_2SO_4), filtered, and concentrated under reduced pressure. The resulting residue was analyzed by ^1H NMR to determine the ratio of **2am** and **2am-*d*1** to be 0.46:0.54 by integrating the olefinic protons ($[P_D]/[P_H]$ = 1.2).

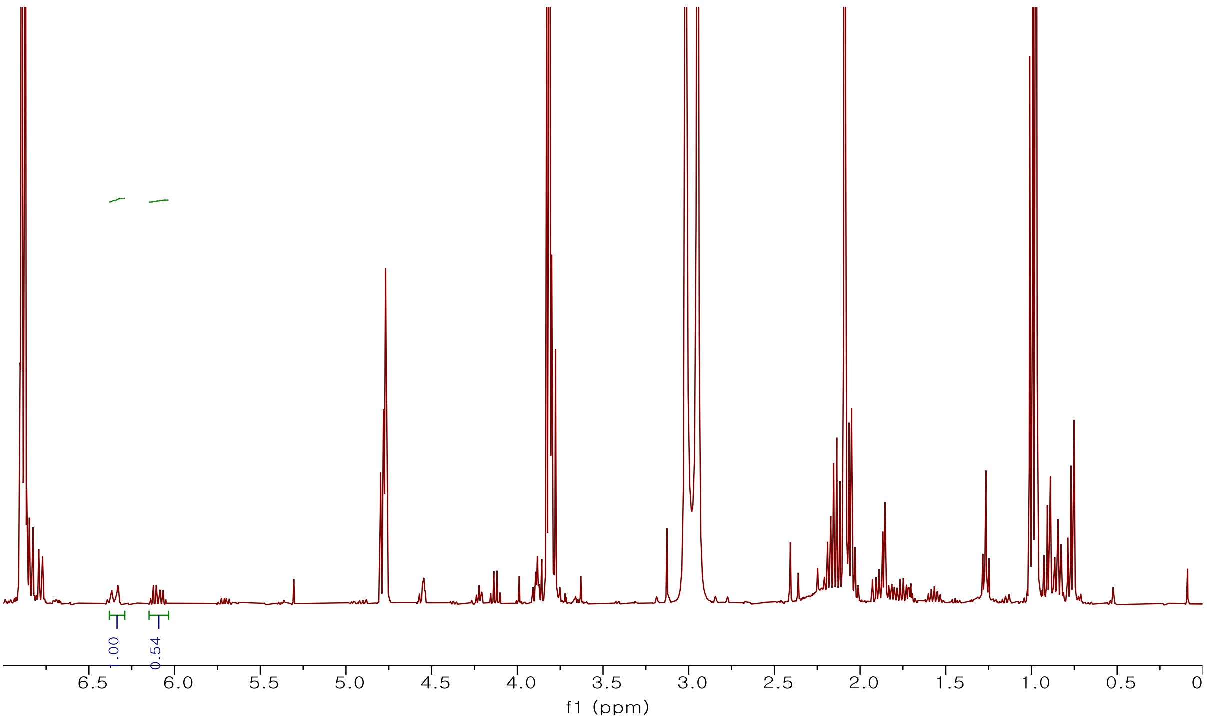

Supplementary Fig. 8. Elimination KIE measurement.

9. Elimination of benzyl chloride intermediate

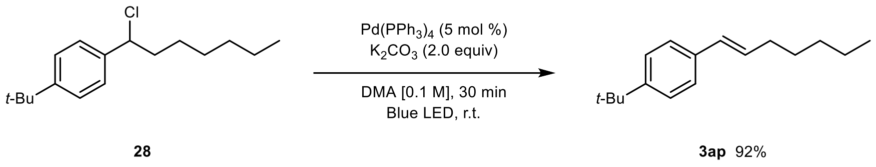

To a 4 mL vial equipped with a stirrer-bar were added $Pd(PPh_3)_4$ (5.8 mg, 0.0050 mmol, 0.050 equiv), K_2CO_3 (27.6 mg, 0.20 mmol, 2.0 equiv), **28** (26.7 mg, 0.10 mmol, 1.0 equiv), and *N*,*N*-dimethylacetamide (1.0 mL). The resulting mixture was stirred for 24 h under 40 W blue LED irradiation with fan cooling (~30 ºC). The reaction mixture was analyzed by ^{1}H NMR with nitrobenzene (10 µL) as an internal standard to determine the yield (92%) of **3ap**. The crude NMR of the reaction mixture is shown in Supplementary Fig. 9.

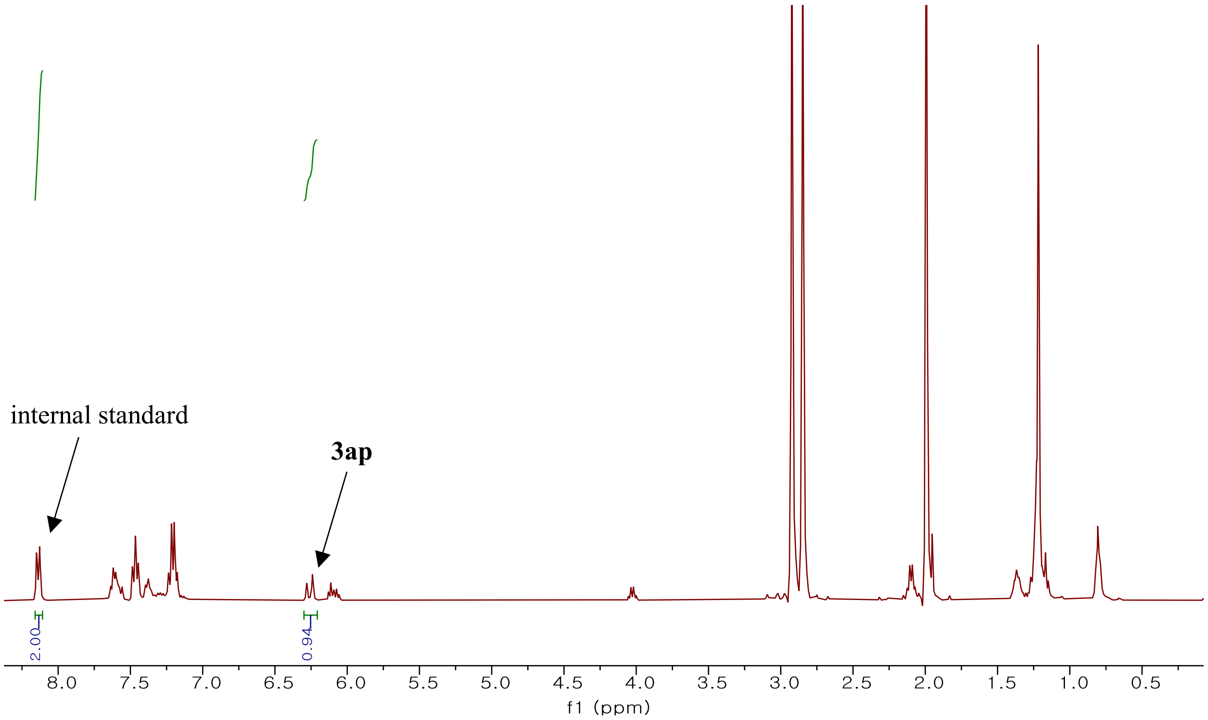

Supplementary Fig. 9. Crude NMR of the elimination experiment.

10. Control experiment with *tert*-butyl *N*-(acyloxy)phthalimide

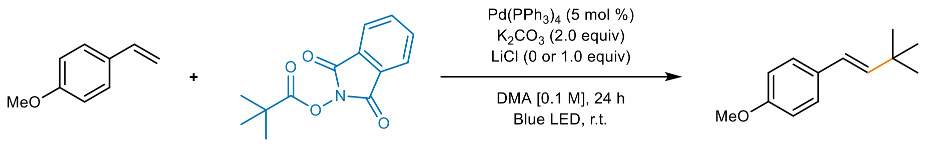

1a **2al** **3a**

To a 4 mL vial equipped with a stirrer-bar were added $Pd(PPh_3)_4$ (5.8 mg, 0.0050 mmol, 0.050 equiv), K_2CO_3 (27.6 mg, 0.20 mmol, 2.0 equiv), **1a** (13.3 µL, 0.10 mmol, 1.0 equiv), **2al** (37.1 mg, 0.15 mmol, 1.5 equiv), LiCl if appropriate (4.2 mg, 0.10 mmol, 1.0 equiv), and *N*,*N*-dimethylacetamide (1.0 mL). The resulting mixture was stirred for 24 h under 40 W blue LED irradiation with fan cooling (~30 ºC). The reaction mixture was analyzed by gas chromatography using dodecane as an internal standard to determine the yield of product **3a**. The results are shown below.

Supplementary Table 1. Control experiment with 2al

Entry	LiCl	Yield
**1**	–	13%
2	1.0 equiv	22%

11. Computational details

General information

All calculations were carried out using DFT as implemented in the Gaussian 09[31] program packages. Gas phase geometry optimizations were conducted with the B3LYP[32] hybrid functional including Grimme's D3 dispersion correction[33] and the 6-31G** basis set and LanL2DZ[34,35] basis set for Pd. The energies of the optimized structures were reevaluated by additional single point calculations using B3LYP hybrid functional including Grimme's D3 dispersion correction and the 6-311++G** basis set and the SDD basis set for Pd. The integral equation formalism variant of the Polarizable Continuum Model (IEFPCM) was employed as implemented to account for the solvation effects for DMA (ε = 37.781). Analytical vibrational frequencies within the harmonic approximation were computed with the 6-31G**/LanL2DZ(Pd) basis set to confirm proper convergence to well-defined minima (no imaginary frequency) or saddle points (one and only one imaginary frequency) on the potential energy surface. All thermal corrections from the vibrational frequency calculations were performed at 25 °C (298.15 K).

Time-dependent density functional theory (TD-DFT) calculations were carried out as implemented in the Gaussian 09 program package using the hybrid exchange/correlation CAM-B3LYP[36] functional with long-range corrections and the 6-311++G**/SDD(Pd) basis set. The solvation effects for DMA (ε = 37.781) were accounted for using the integral equation formalism variant of the Polarizable Continuum Model (IEFPCM) as implemented.

Computation of redox potentials

The standard reduction potentials, E^{o}_{red} were obtained from the electron attachment energy in the solution phase, ΔG^{EA}(sol), and subsequent application of the following relationships, where n is the number of electrons, E is the absolute potential and F is the Faraday constant. ΔG^{EA}(sol) was computed by subtracting the Gibbs free energies of the oxidized species from those of the reduced species.

$$\Delta G^{EA}(\text{sol}) = -nFE$$

$$E^{o}_{red}\ (\text{V vs. N.H.E.}) = -E - E^{o}\,(\text{N.H.E.}) = -E - 4.43\ \text{V}$$

Here, we employed the absolute potential that was measured to be 4.43 V for the normal hydrogen electrode.[37] The species are denoted as "**I-OMe-rad**", "**I-OMe-cat**" and their analogues according to the substituent on the aryl group. The reduction potentials of $Pd(PPh_3)_3Cl$ and $Pd(PPh_3)_2Cl_2$ were also computed accordingly.

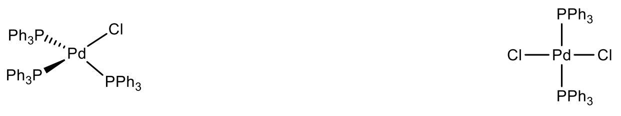

E^{o}_{calc} $[Pd(PPh_3)_3Cl/Pd(PPh_3)_3Cl^{-}]$ = –0.85 V E^{o}_{calc} $[Pd(PPh_3)_2Cl_2/Pd(PPh_3)_2Cl_2^{-}]$ = –0.83 V

Supplementary Fig. 10. Computed reduction potentials of Pd complexes.

Kinetic isotope effect computations

Kinetic isotope effect computations were conducted by using the (Iso=2) keyword on the hydrogen atom(s) of interest and re-performing the frequency calculations. The KIE value was calculated from the following reaction through intramolecular competition, H-TS and D-TS, and applying the Boltzmann distribution.

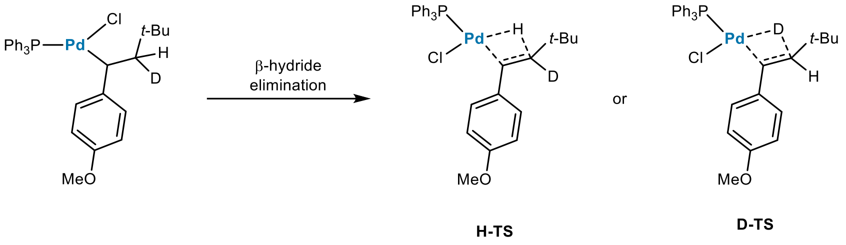

Supplementary Table 2. KIE computations

G (H-TS) (kcal/mol)	G (D-TS) (kcal/mol)	ΔG (kcal/mol)	KIE
-1384623.614	-1384622.662	0.952	5.0

Time-dependent density functional theory computations

Time-dependent density functional theory (TD-DFT) computations were conducted on a model complex of **II** shown below. The molecular orbitals diagrams and TD-DFT results are summarized below.

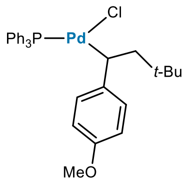

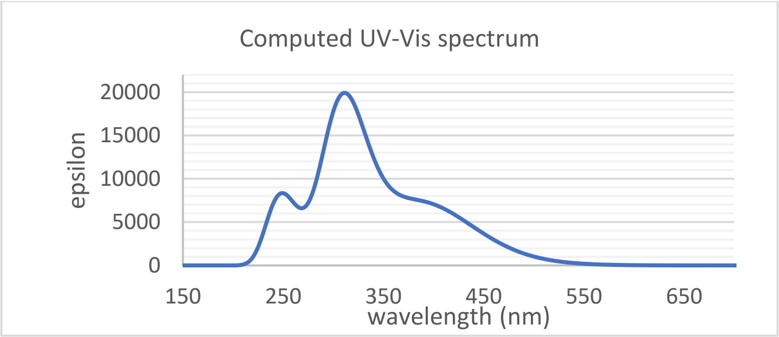

State	Wavelength (nm)	f	Transitions
1 (4)	423.37	0.0787	**H–3 → L** (7.8%)<br>**H–2 → L** (2.7%)<br>**H–1 → L** (38.2%)<br>**H → L** (35.8%)
2 (7)	392.54	0.0368	**H–13 → L** (8.7%)<br>**H–2 → L** (56.7%)<br>**H–1 → L** (18.0%)<br>**H → L** (5.4%)

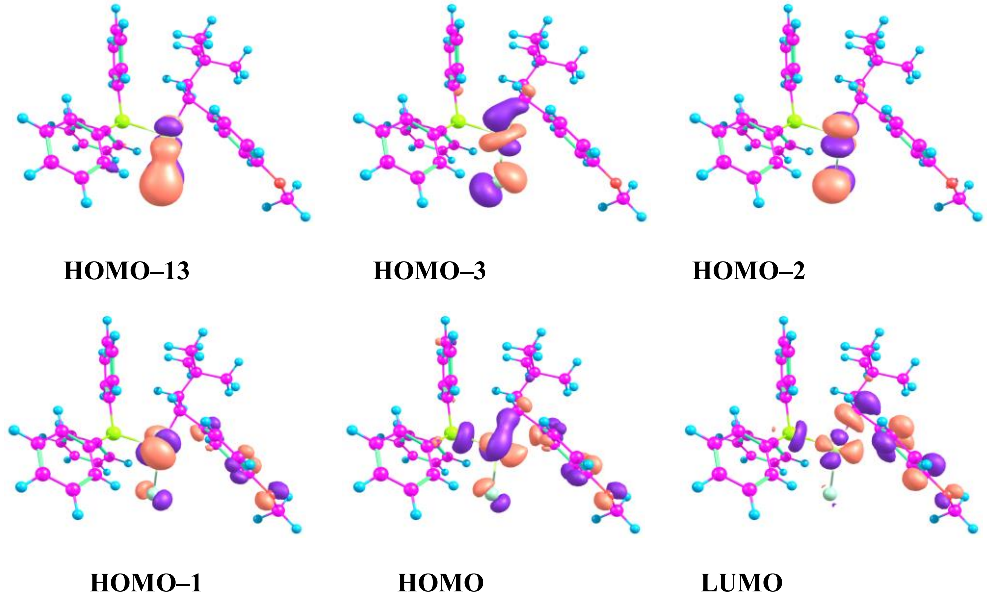

Supplementary Fig. 11. Time-dependent density functional theory computations.

Energy components of DFT-optimized structures

	E(SCF)/(Hartree)	Thermal Corr. to G (Hartree)	G(sol)/(kcal/mol)
	6-311++G**/SDD	6-31G**/LanL2DZ	
**I-OMe-rad**	-582.2024828	0.246983	-365182.3137
**I-OMe-cat**	-582.043615	0.250666	-365080.3116
**I-OAc-rad**	-695.5966132	0.252701	-436334.563
**I-OAc-cat**	-695.4228344	0.253819	-436224.8137
**I-F-rad**	-566.9106881	0.209082	-355610.3581
**I-F-cat**	-566.7356712	0.211211	-355499.1975
**I-Cl-rad**	-927.2670096	0.206688	-581738.6953
**I-Cl-cat**	-927.089249	0.209216	-581625.5626
**I-CF_3-rad**	-804.792516	0.216696	-504878.5682
**I-CF_3-cat**	-804.6095457	0.218972	-504762.3245
**I-CHO-rad**	-581.0109057	0.225376	-364448.1469
**I-CHO-cat**	-580.8199019	0.226123	-364327.8216
**I-CN-rad**	-559.9180952	0.214513	-351219.0351
**I-CN-cat**	-559.7262494	0.216342	-351097.5025
$Pd(PPh_3)_3Cl$	-3697.953287	0.737484	-2320036.191
$Pd(PPh_3)_3Cl^-$	-3698.082242	0.734904	-2320118.731
$Pd(PPh_3)_2Cl_2$	-3121.647	0.476436	-1958562.619
$Pd(PPh_3)_2Cl_2^-$	-3121.779546	0.476796	-1958645.567
**H-TS**	-2207.028208	0.48836	-1384623.614
**D-TS**	-2207.028208	0.489876	-1384622.662

12. ^{1}H and ^{13}C NMR spectra

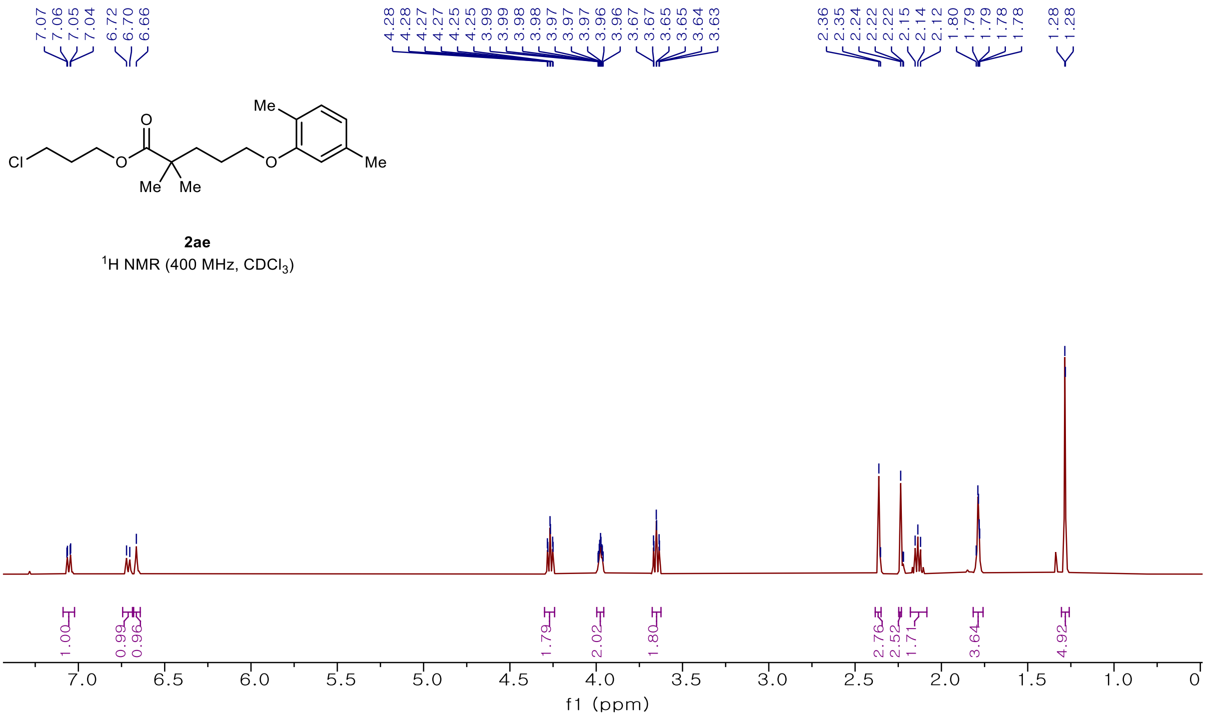

Supplementary Fig. 12. ^{1}H NMR spectrum of compound 2ae.

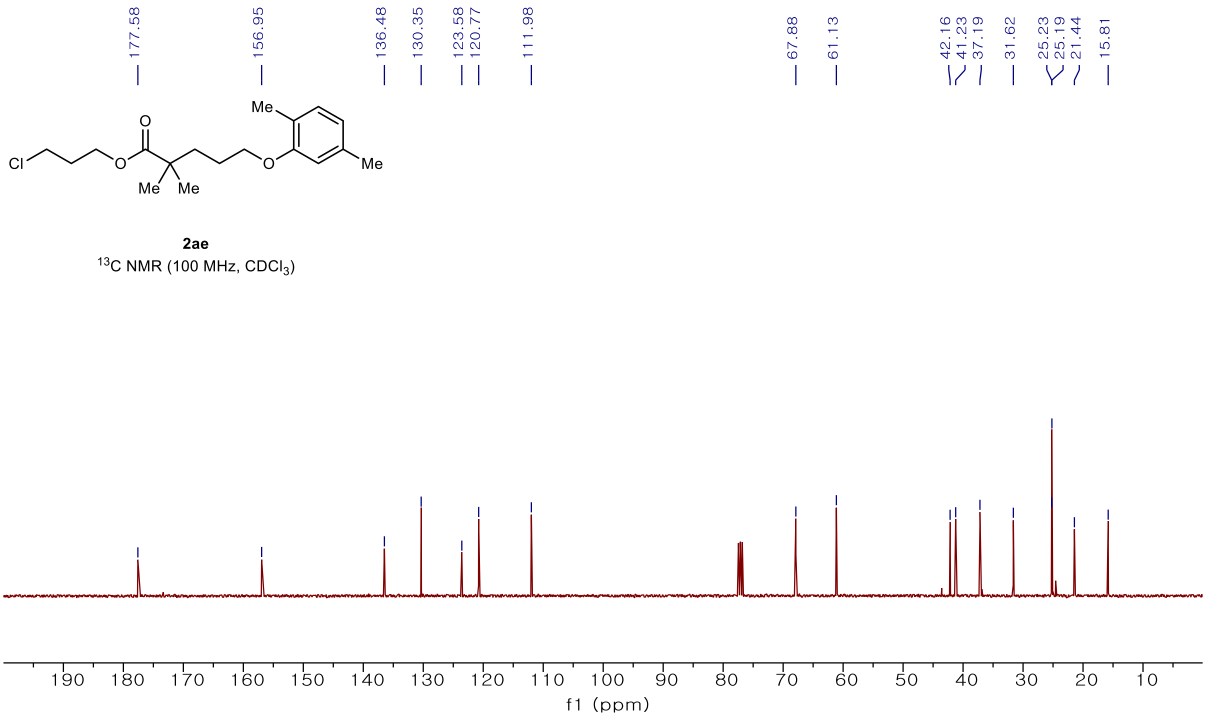

Supplementary Fig. 13. ^{13}C NMR spectrum of compound 2ae.

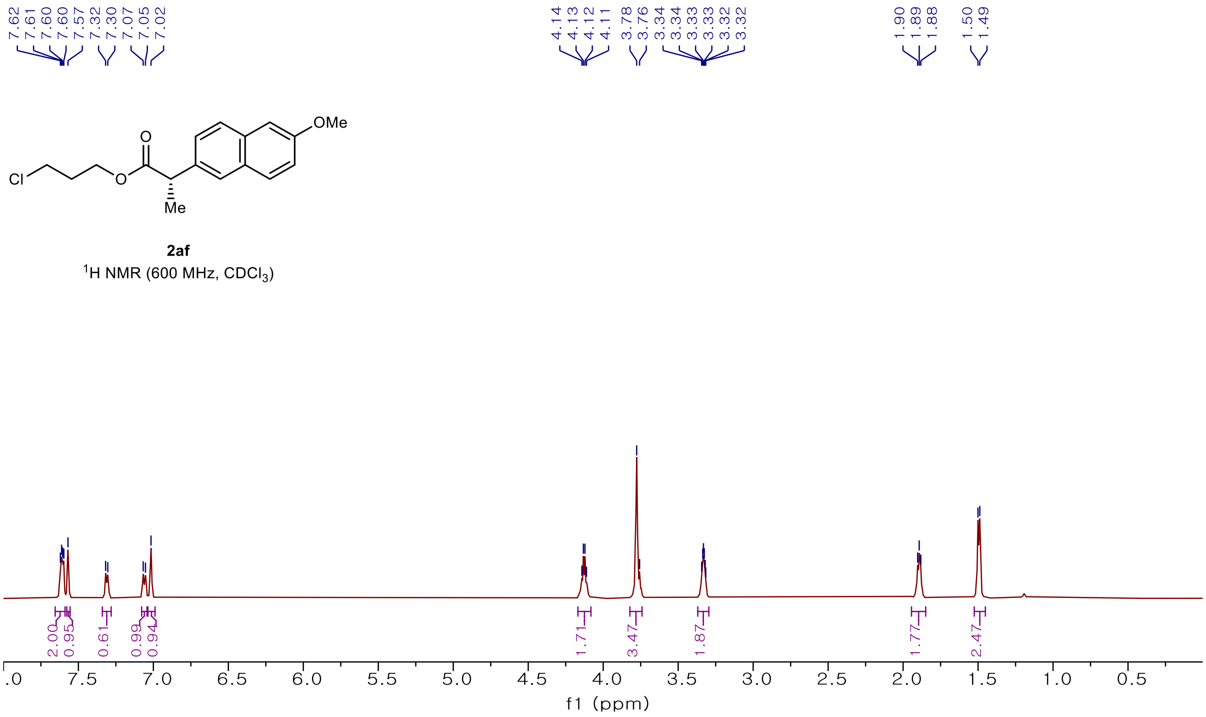

Supplementary Fig. 14. ^{1}H NMR spectrum of compound 2af.

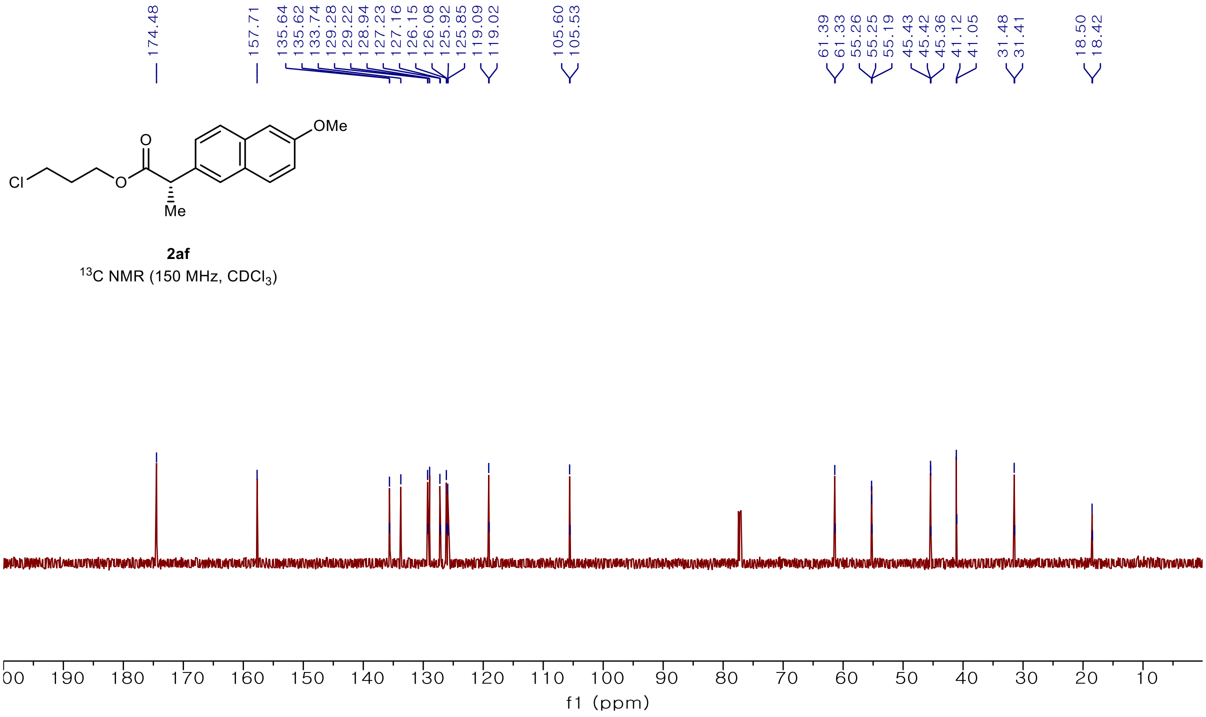

Supplementary Fig. 15. ^{13}C NMR spectrum of compound 2af.

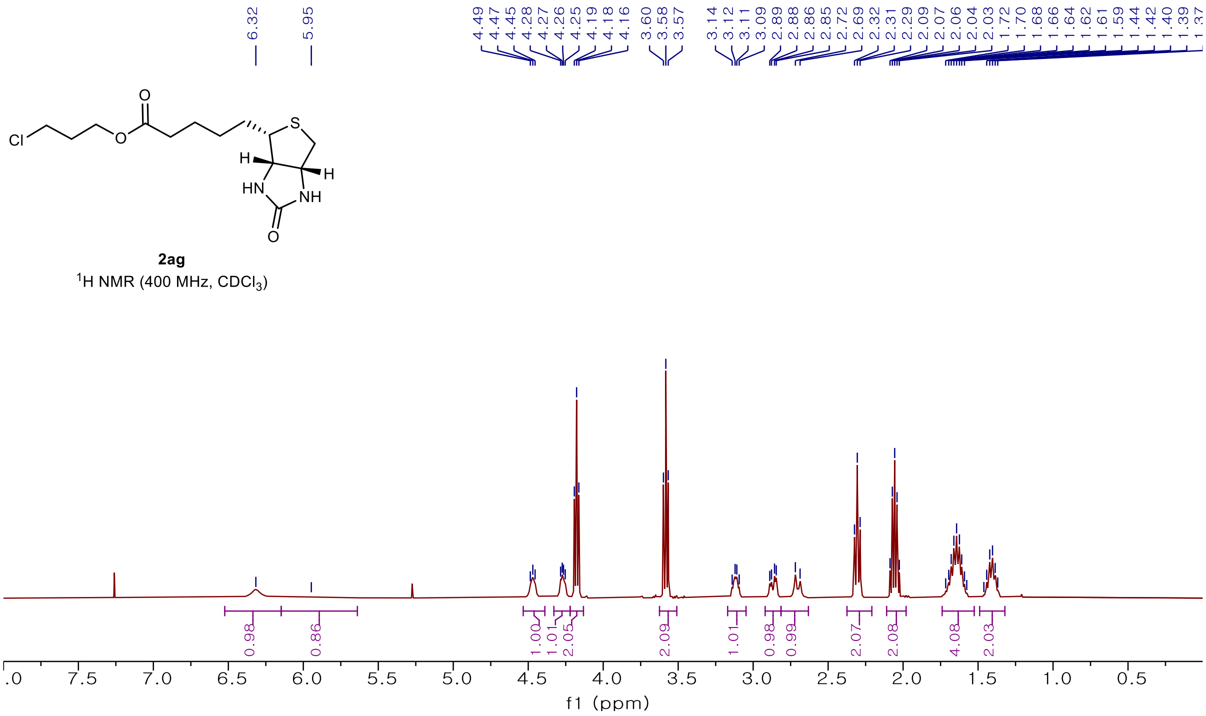

Supplementary Fig. 16. ^{1}H NMR spectrum of compound 2ag.

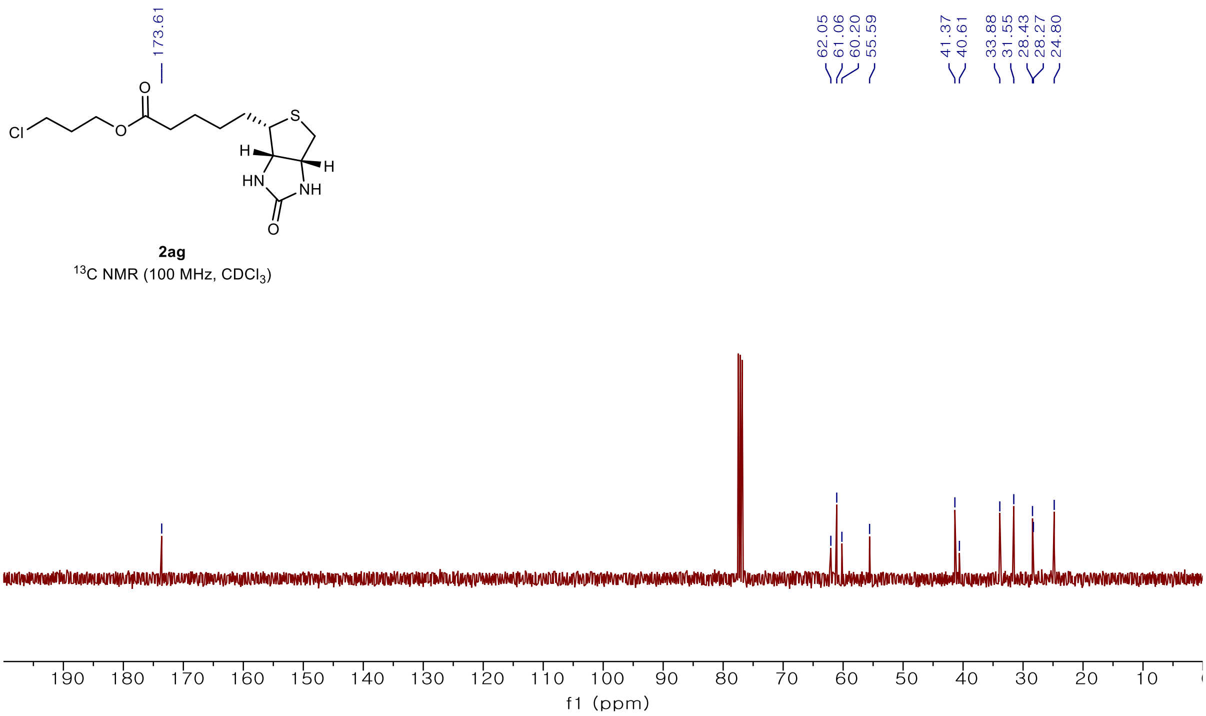

Supplementary Fig. 17. ^{13}C NMR spectrum of compound 2ag.

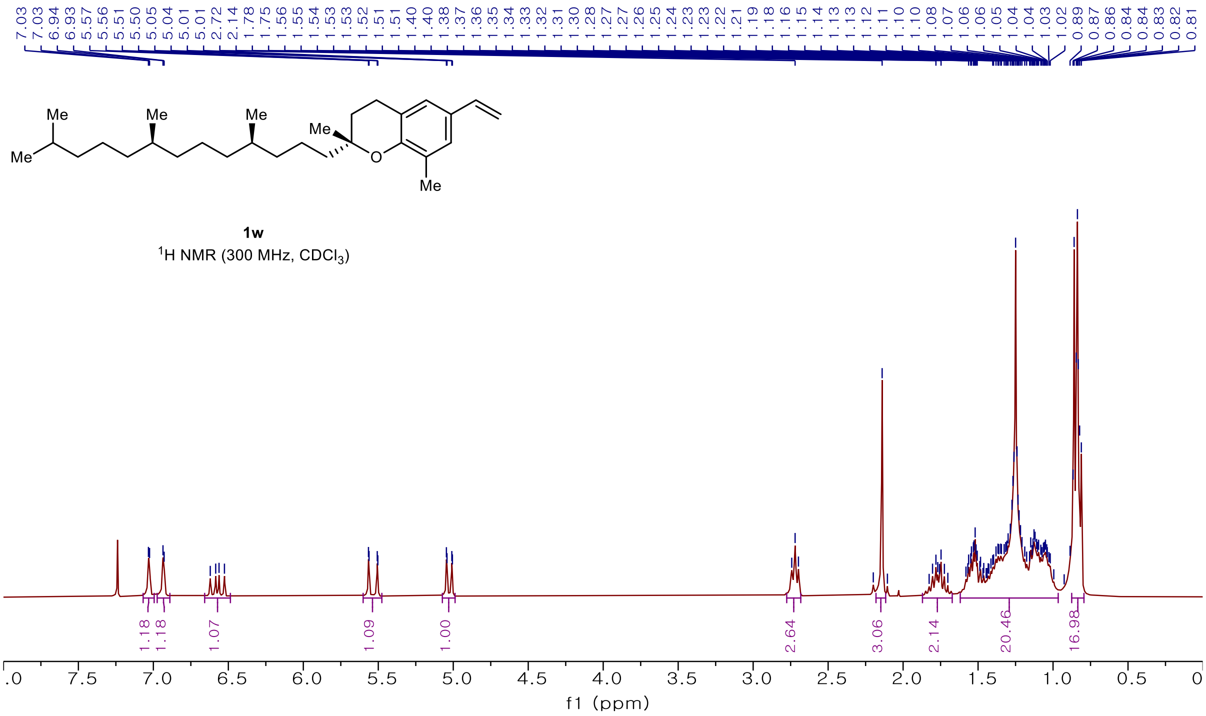

Supplementary Fig. 18. ^{1}**H NMR spectrum of compound 1w.**

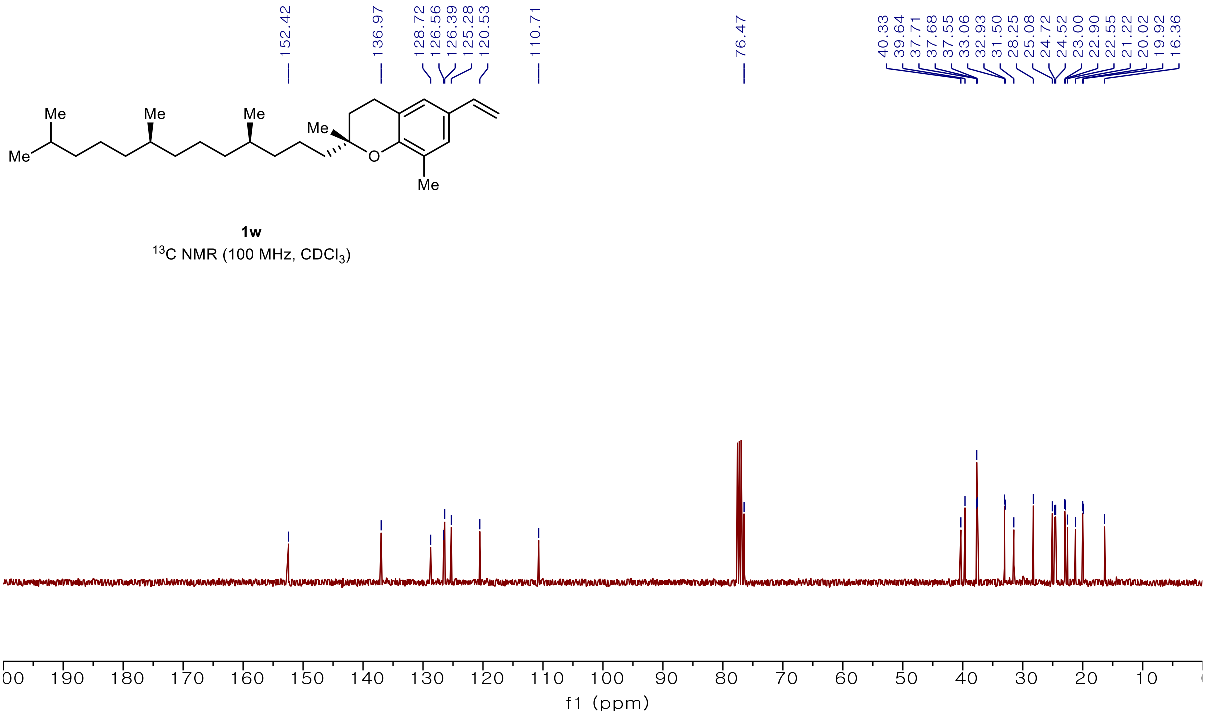

Supplementary Fig. 19. ^{13}**C NMR spectrum of compound 1w.**

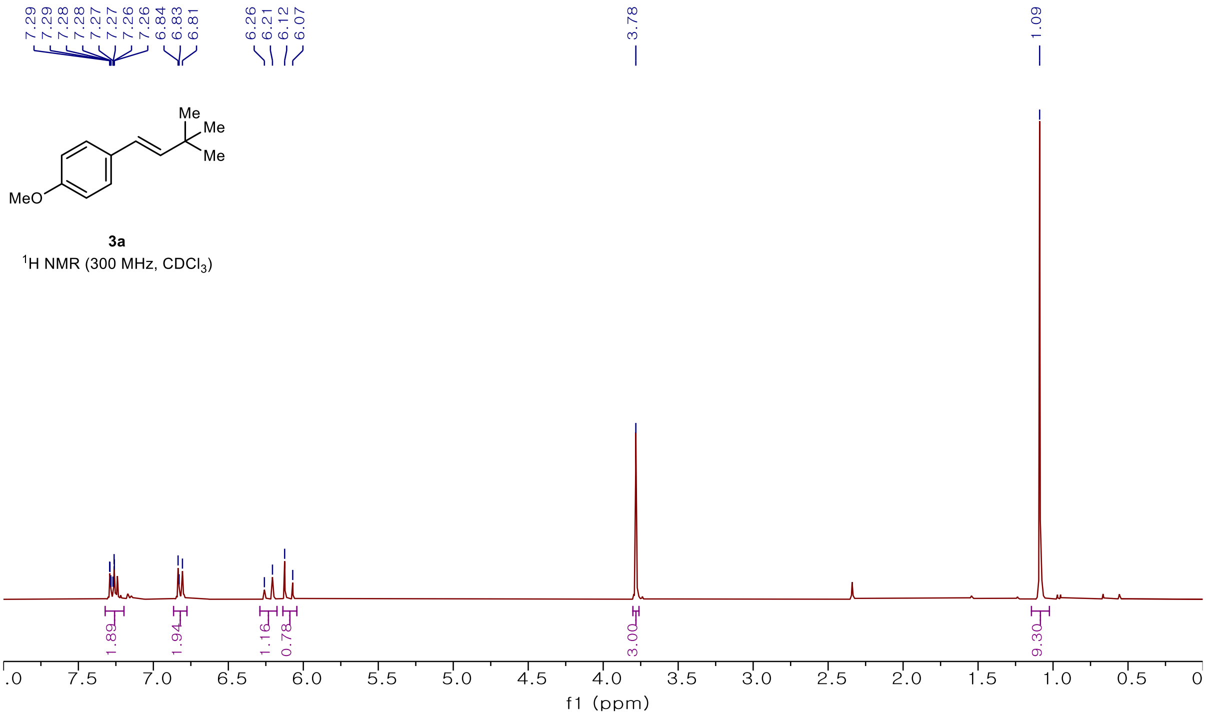

Supplementary Fig. 20. ^{1}**H NMR spectrum of compound 3a.**

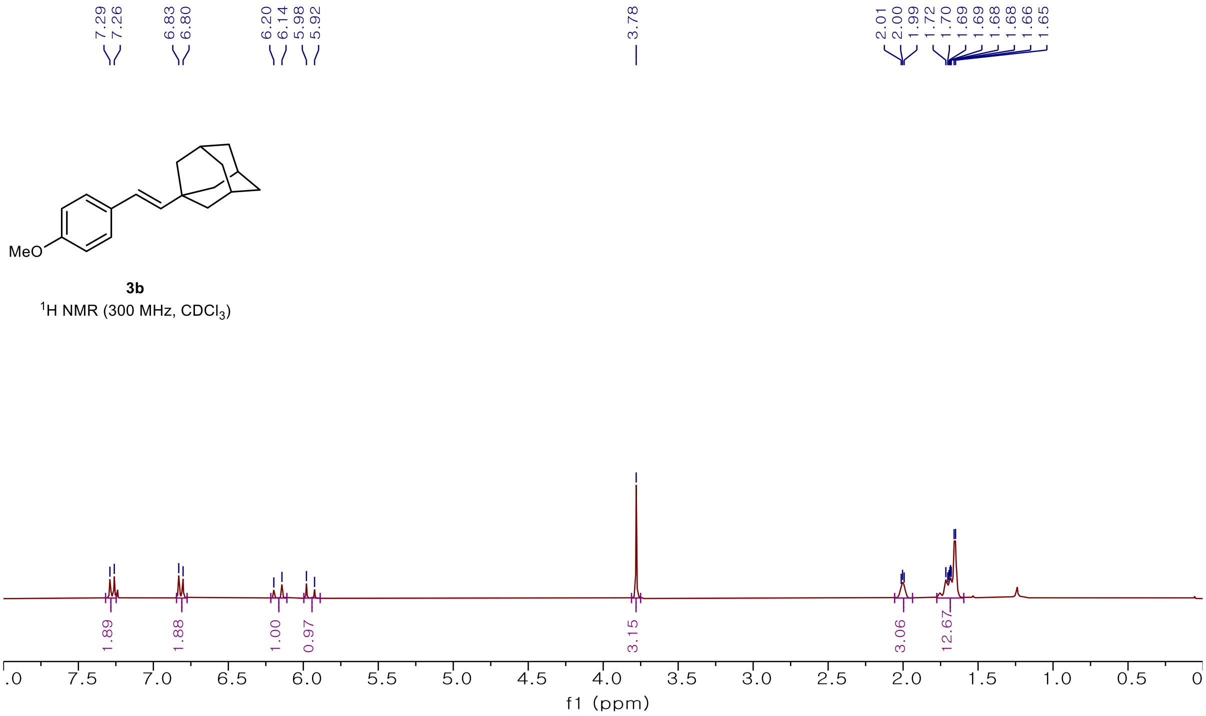

Supplementary Fig. 21. ^{1}**H NMR spectrum of compound 3b.**

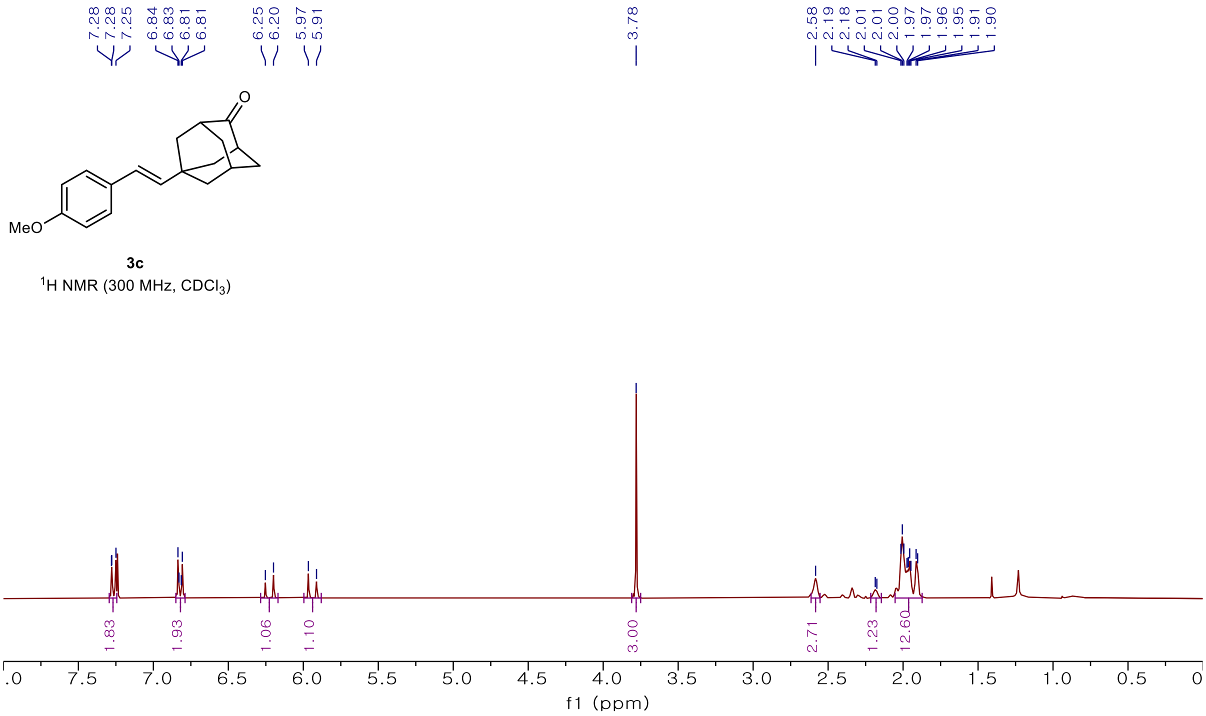

Supplementary Fig. 22. ^{1}H NMR spectrum of compound 3c.

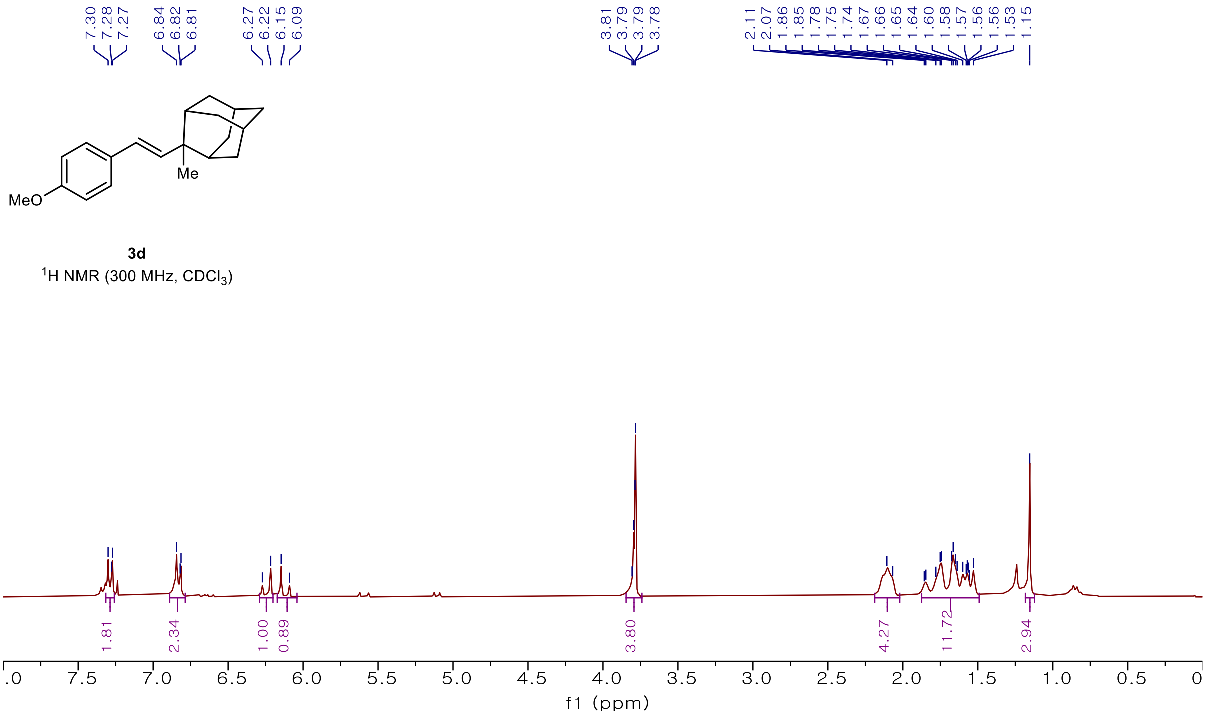

Supplementary Fig. 23. ^{1}H NMR spectrum of compound 3d.

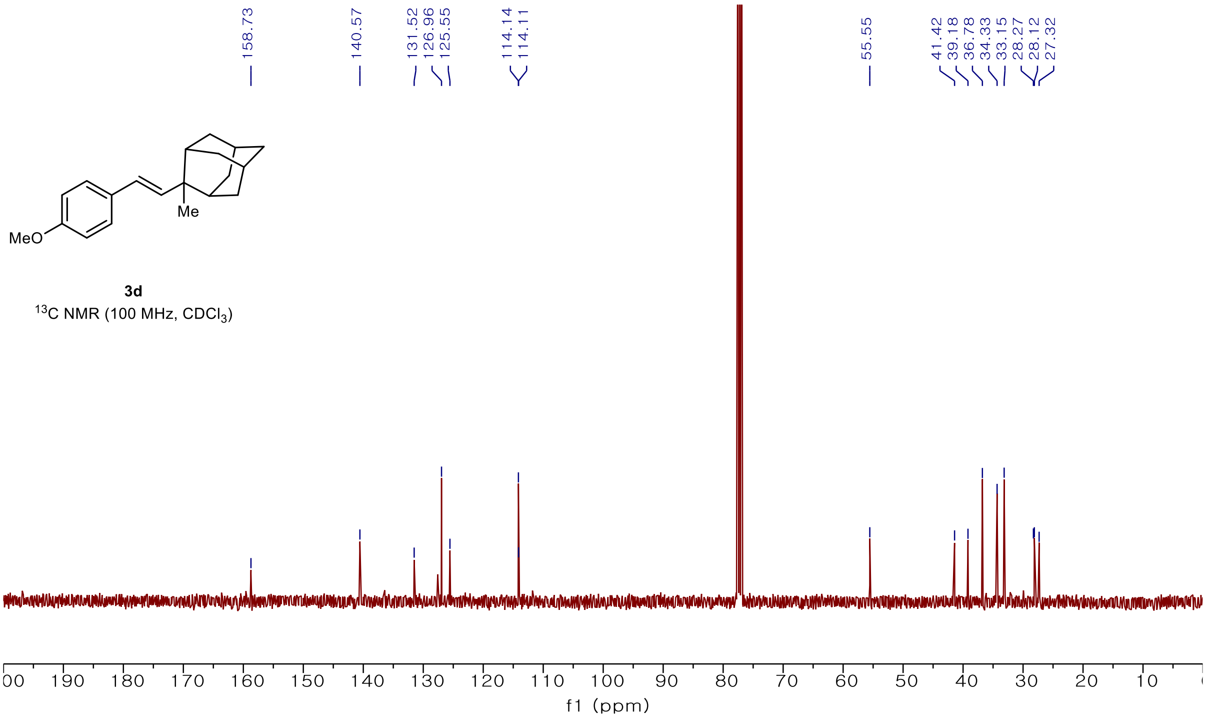

Supplementary Fig. 24. ^{13}C NMR spectrum of compound 3d.

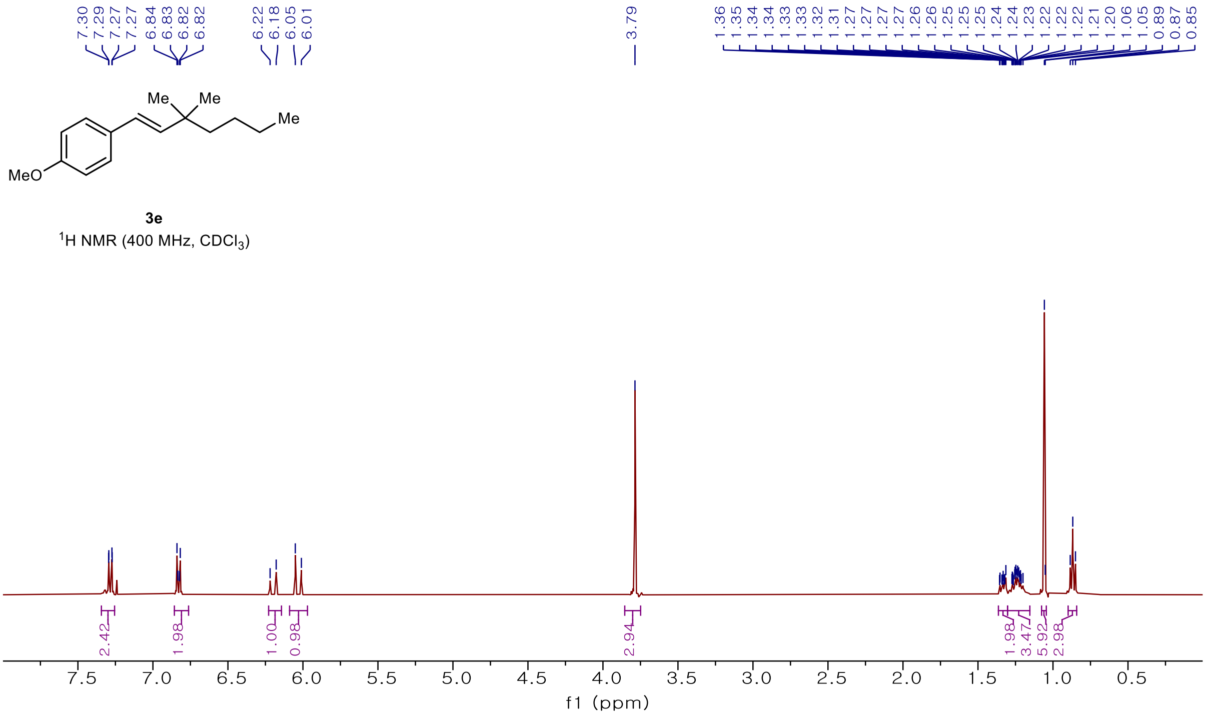

Supplementary Fig. 25. ^{1}H NMR spectrum of compound e3.

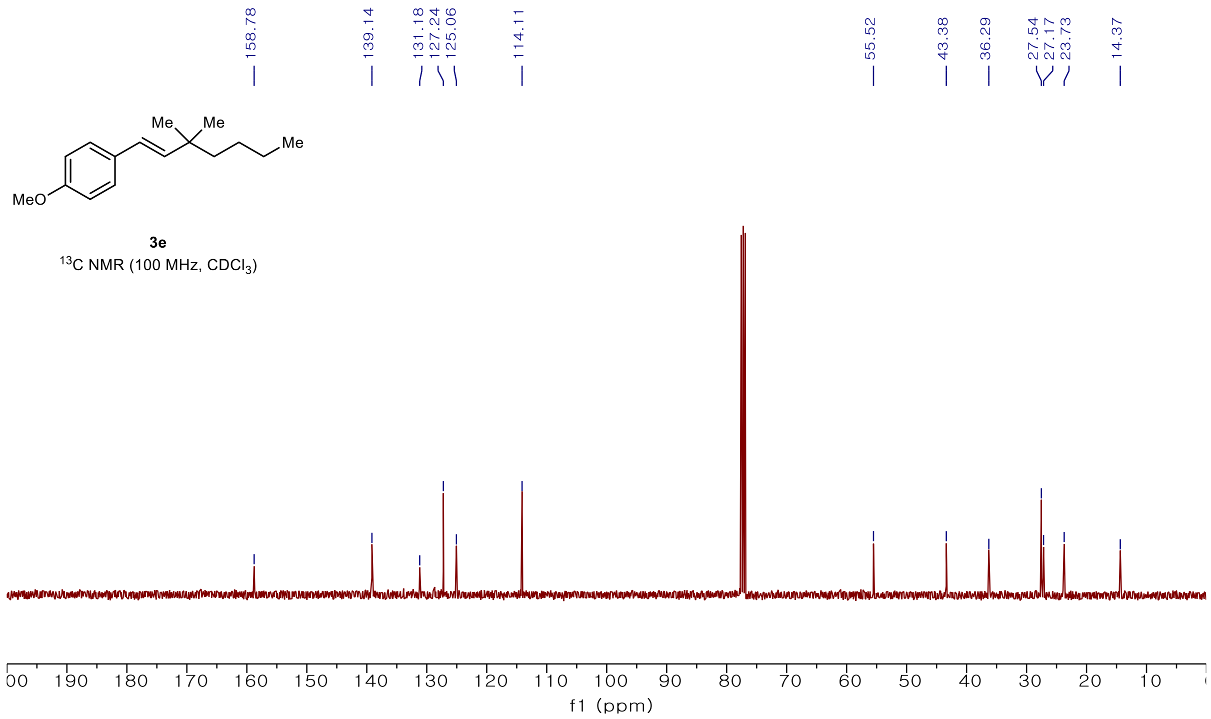

Supplementary Fig. 26. ^{13}C NMR spectrum of compound 3e.

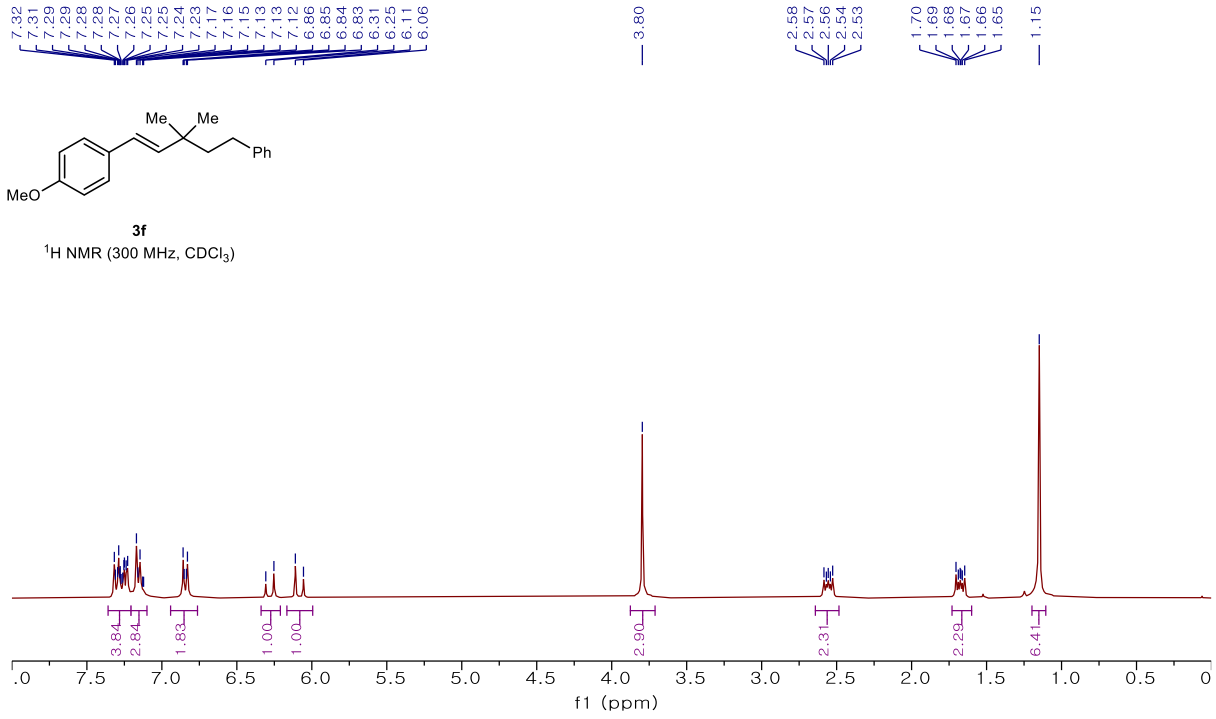

Supplementary Fig. 27. ^{1}H NMR spectrum of compound 3f.

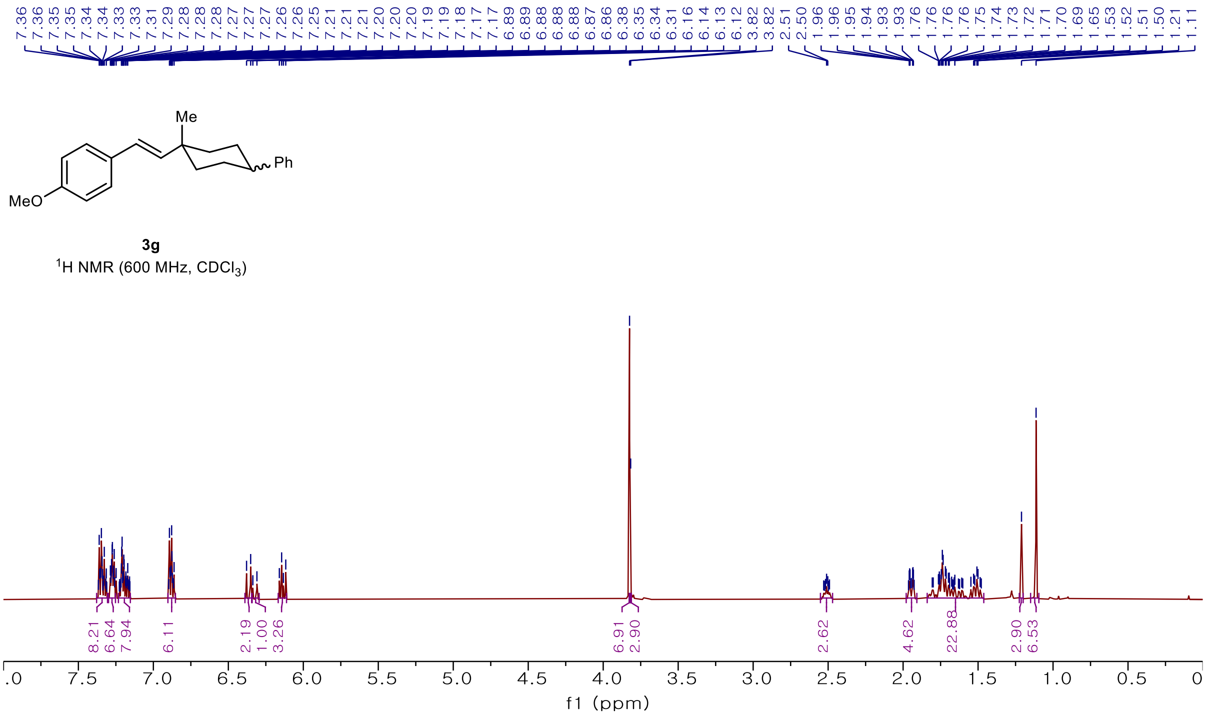

Supplementary Fig. 28. ^{1}H NMR spectrum of compound 3g.

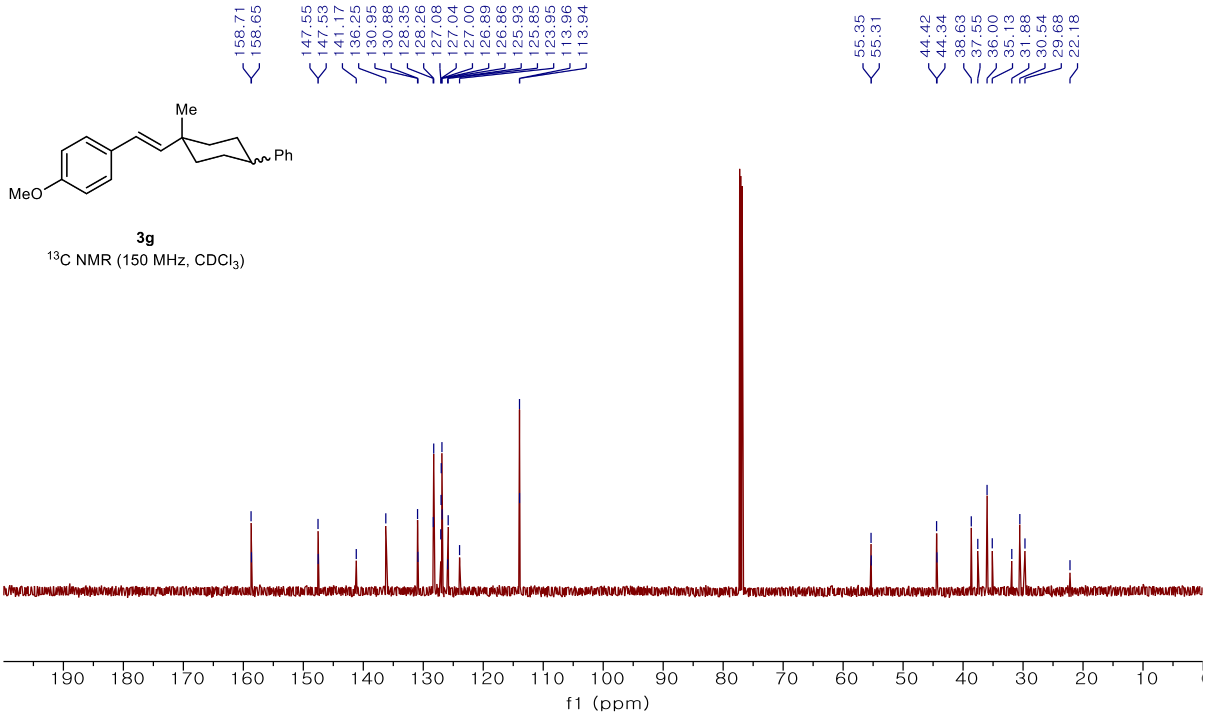

Supplementary Fig. 29. ^{13}C NMR spectrum of compound 3g.

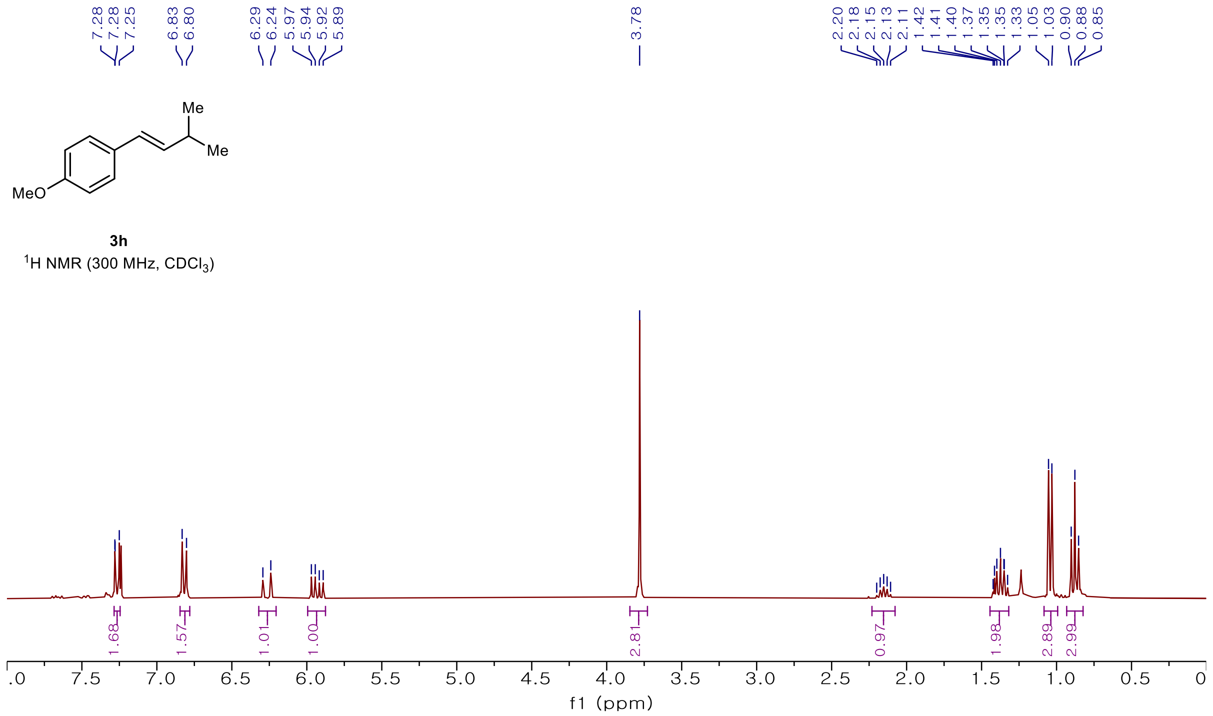

Supplementary Fig. 30. ^{1}H NMR spectrum of compound 3h.

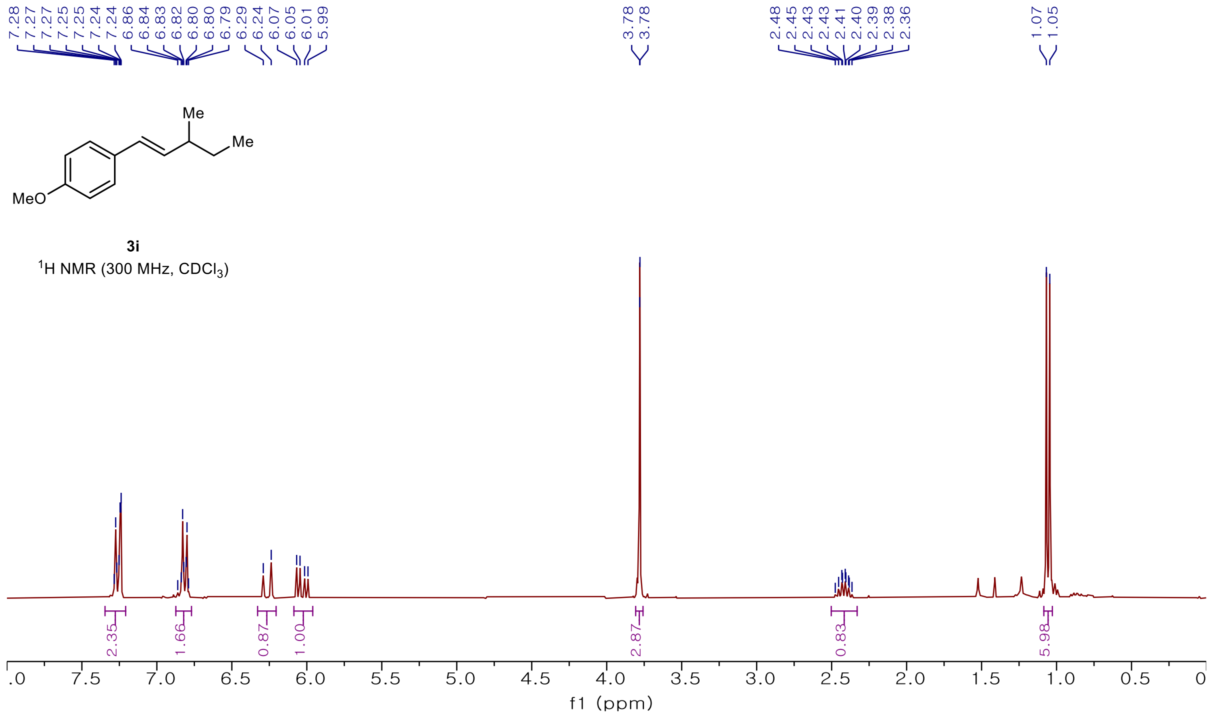

Supplementary Fig. 31. ^{1}H NMR spectrum of compound 3i.

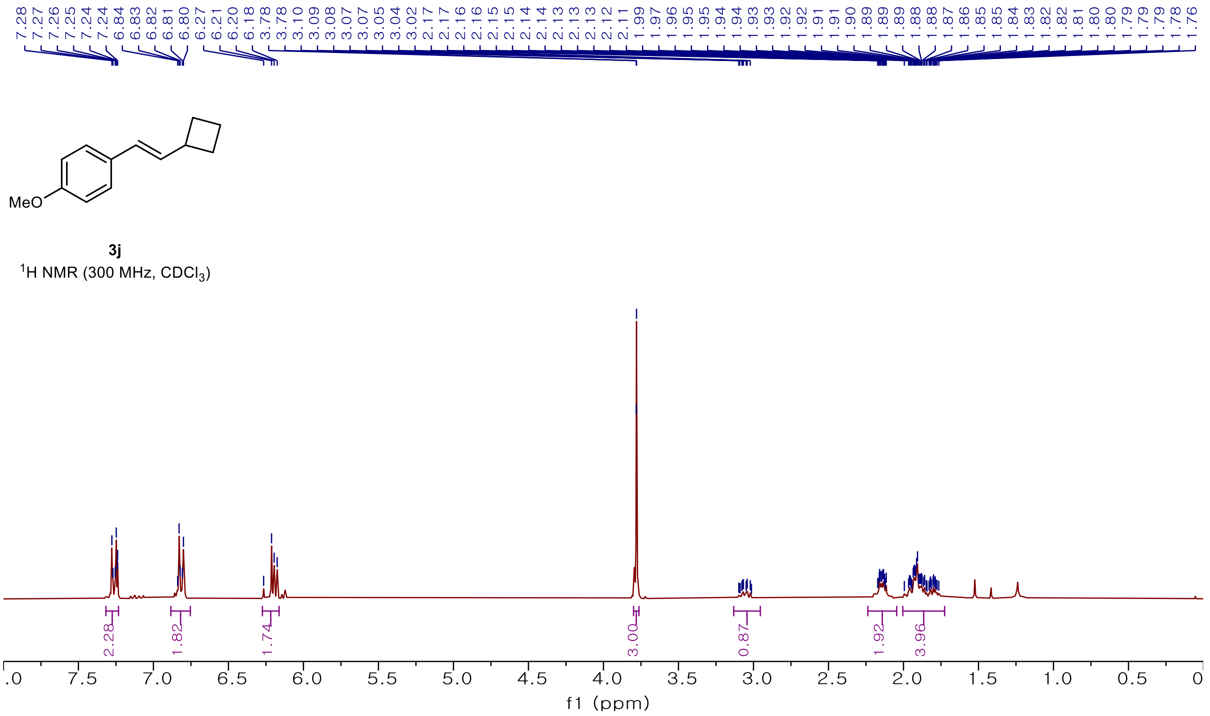

Supplementary Fig. 32. ^{1}H NMR spectrum of compound 3j.

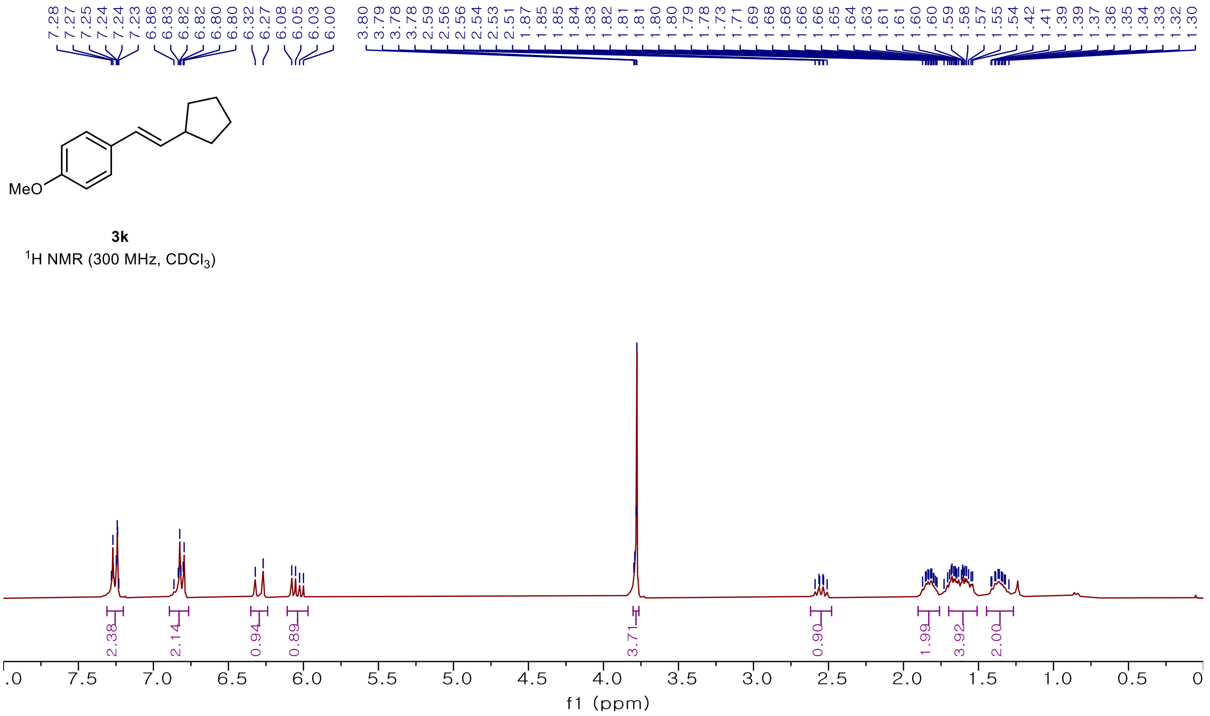

Supplementary Fig. 33. ^{1}H NMR spectrum of compound 3k.

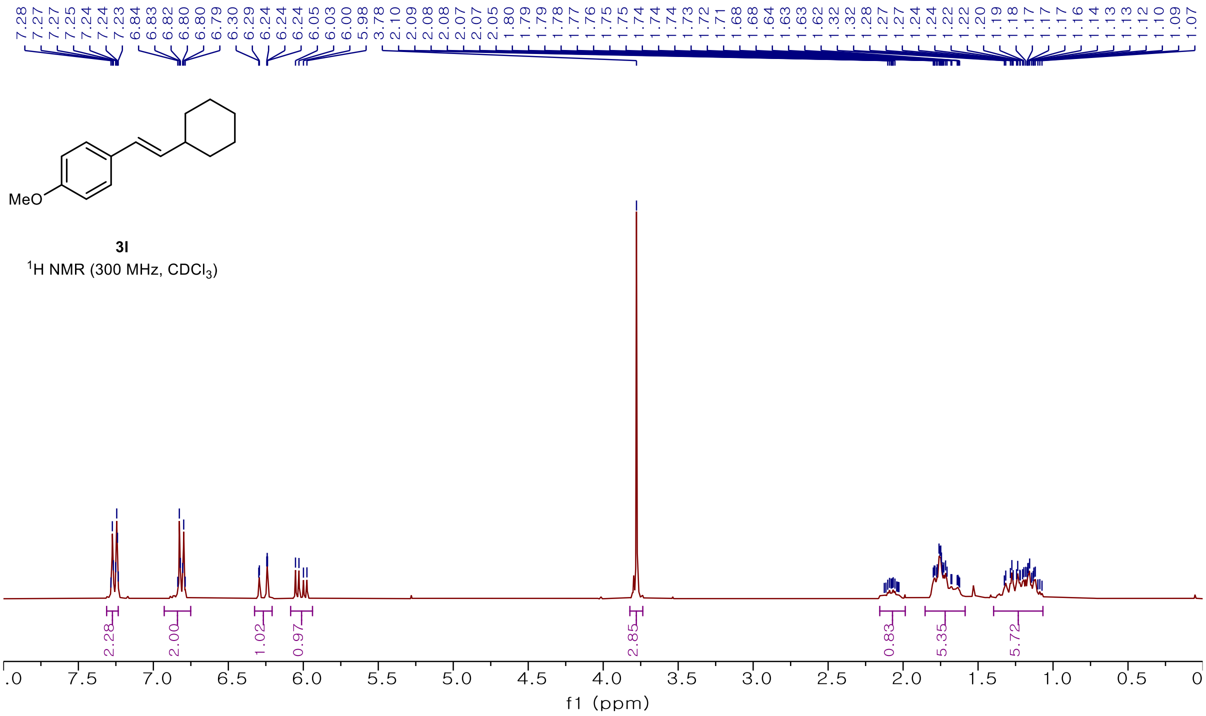

Supplementary Fig. 34. ^{1}H NMR spectrum of compound 3l.

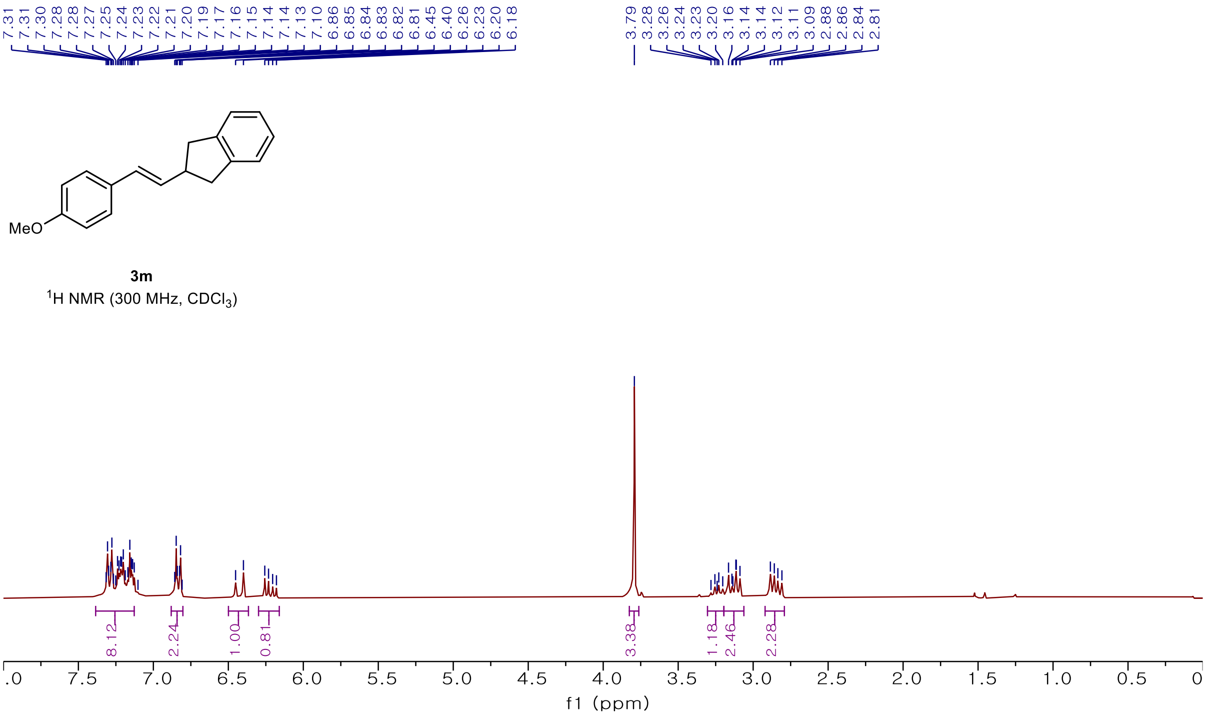

Supplementary Fig. 35. ^{1}H NMR spectrum of compound 3m.

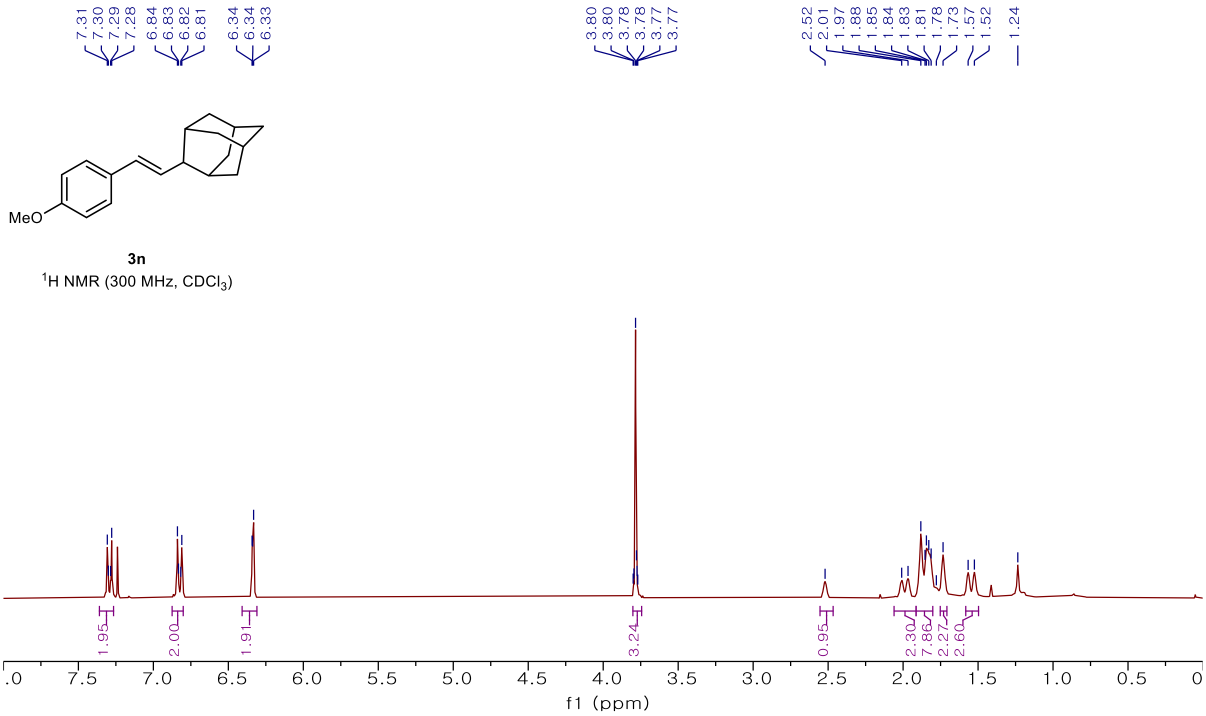

Supplementary Fig. 36. ^{1}H NMR spectrum of compound 3n.

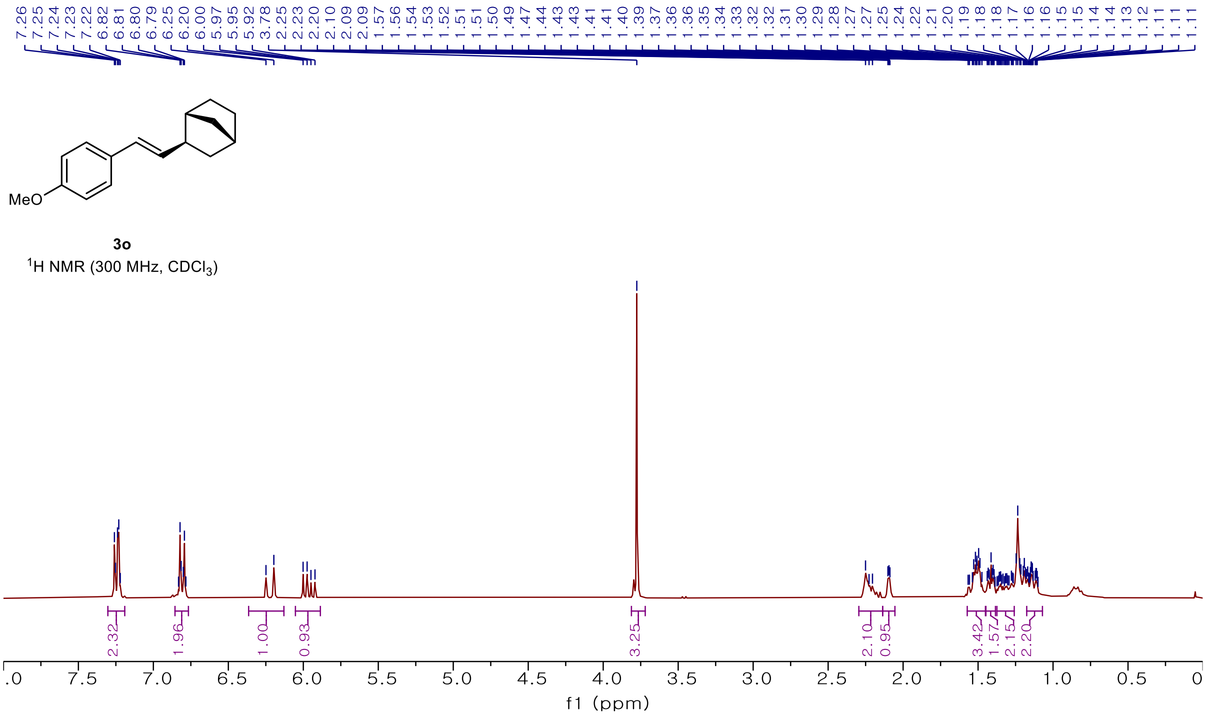

Supplementary Fig. 37. ^{1}H NMR spectrum of compound 3o.

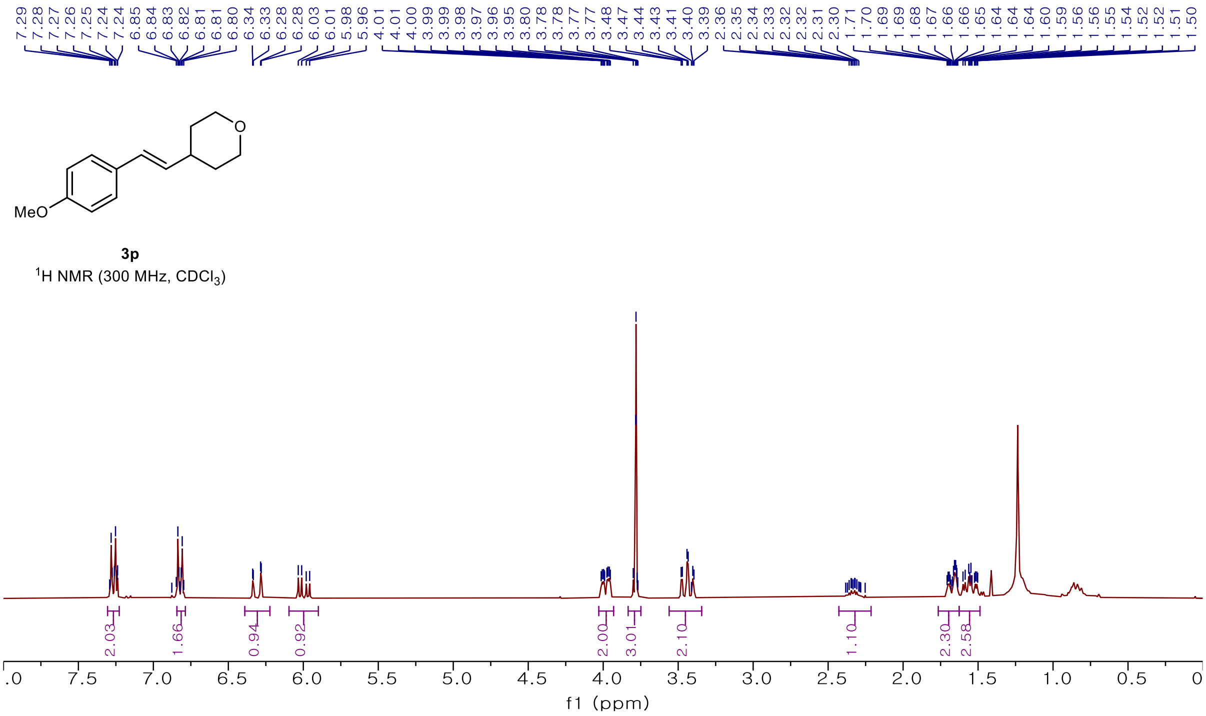

Supplementary Fig. 38. ^{1}**H NMR spectrum of compound 3p.**

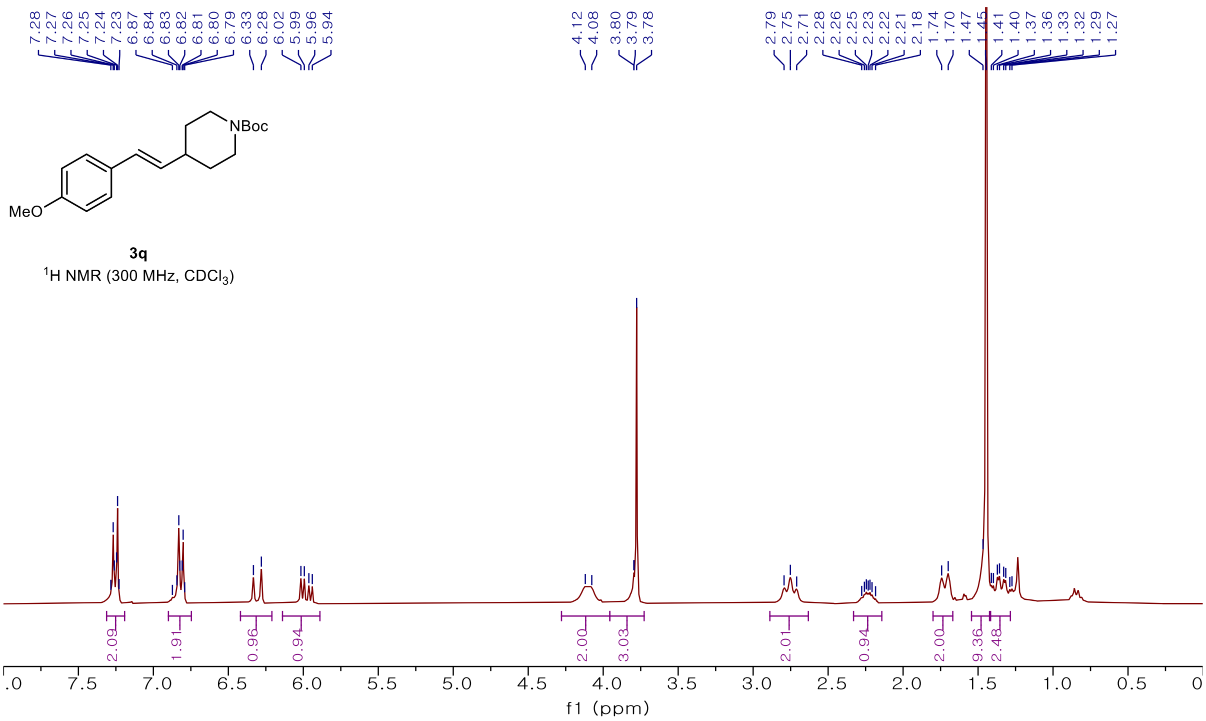

Supplementary Fig. 39. ^{1}**H NMR spectrum of compound 3q.**

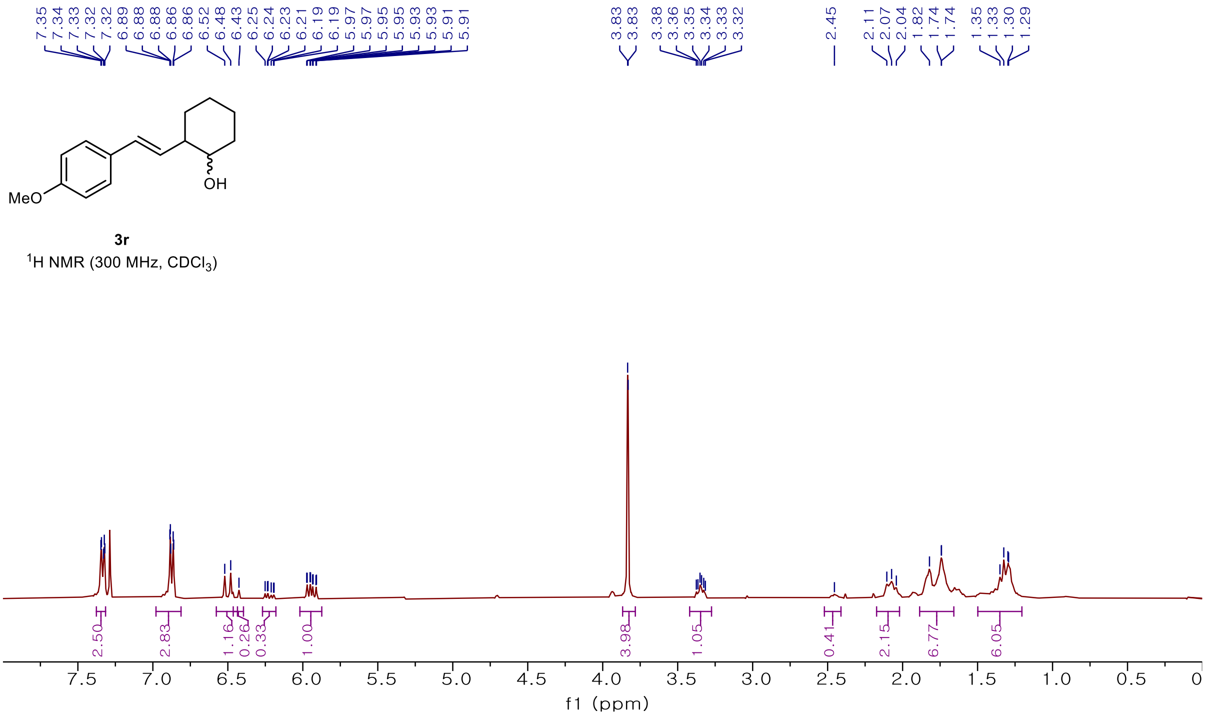

Supplementary Fig. 40. ^{1}H NMR spectrum of compound 3r.

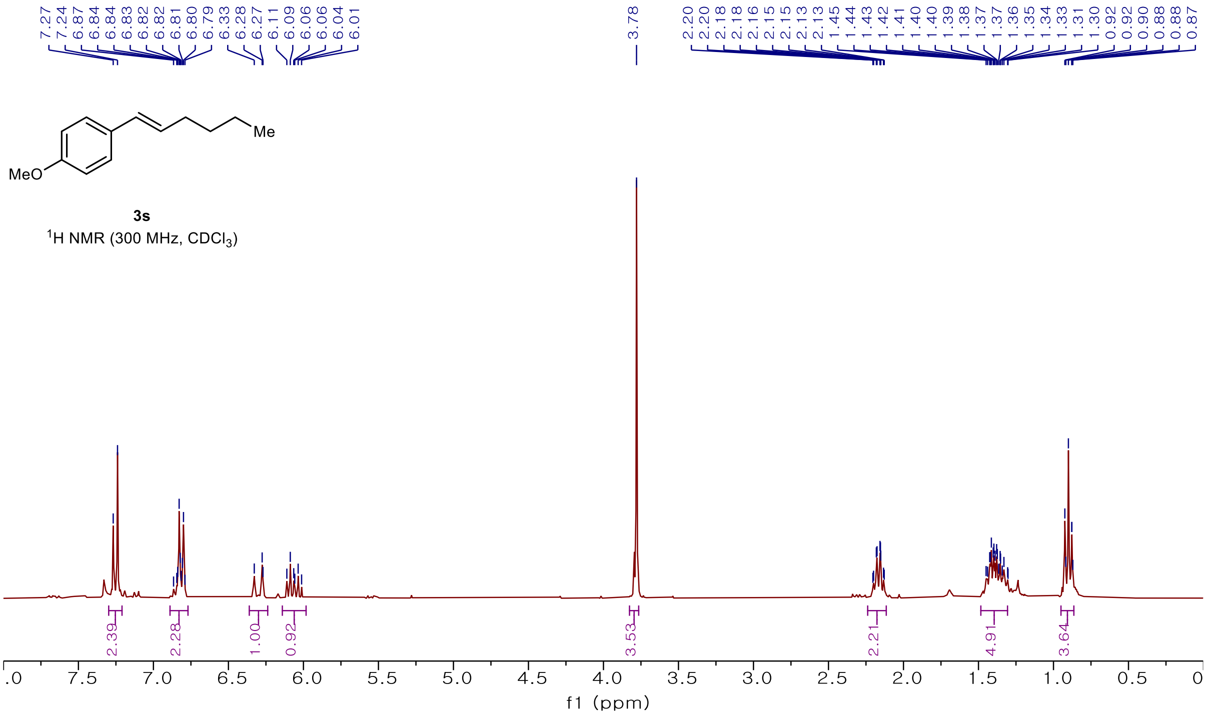

Supplementary Fig. 41. ^{1}H NMR spectrum of compound 3s.

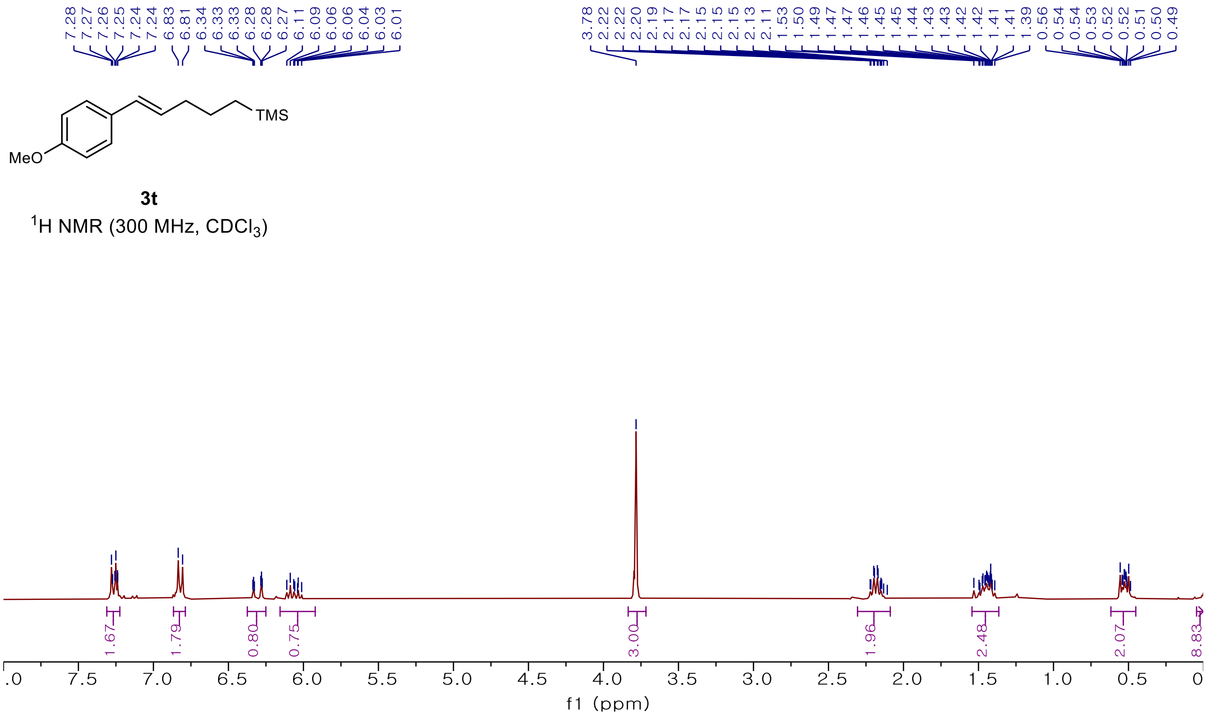

Supplementary Fig. 42. ^{1}H NMR spectrum of compound 3t.

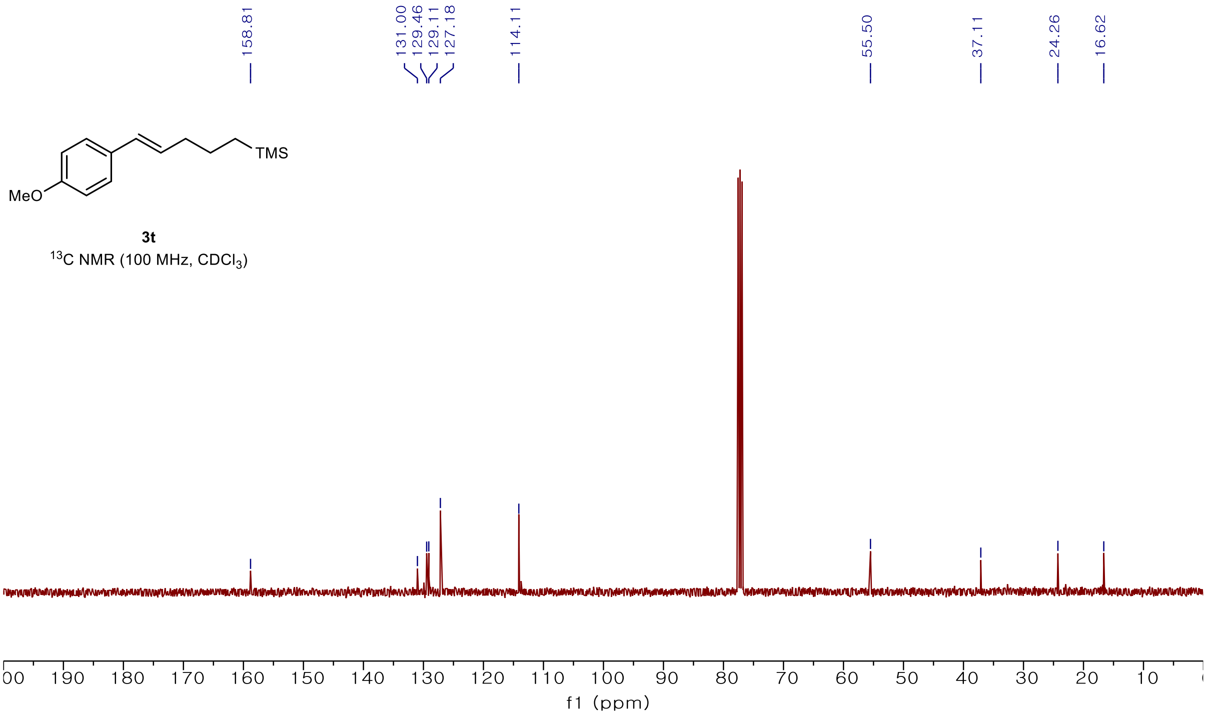

Supplementary Fig. 43. ^{13}C NMR spectrum of compound 3t.

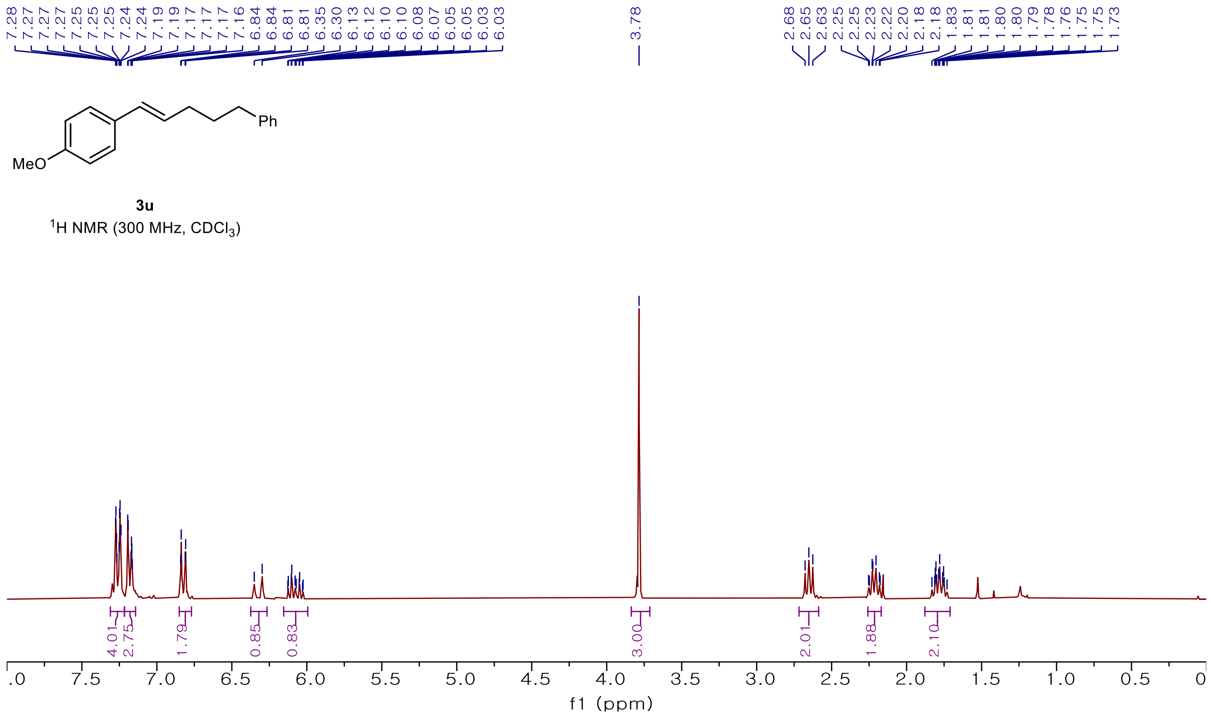

Supplementary Fig. 44. ^{1}H NMR spectrum of compound 3u.

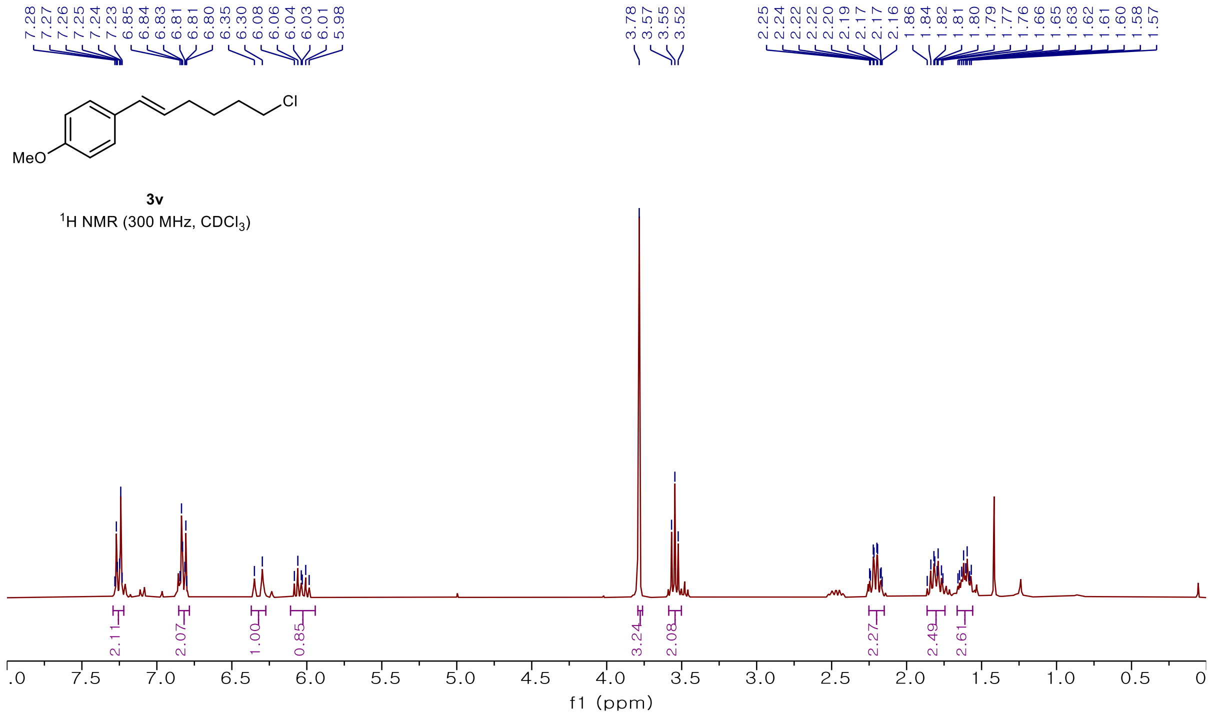

Supplementary Fig. 45. ^{1}H NMR spectrum of compound 3v.

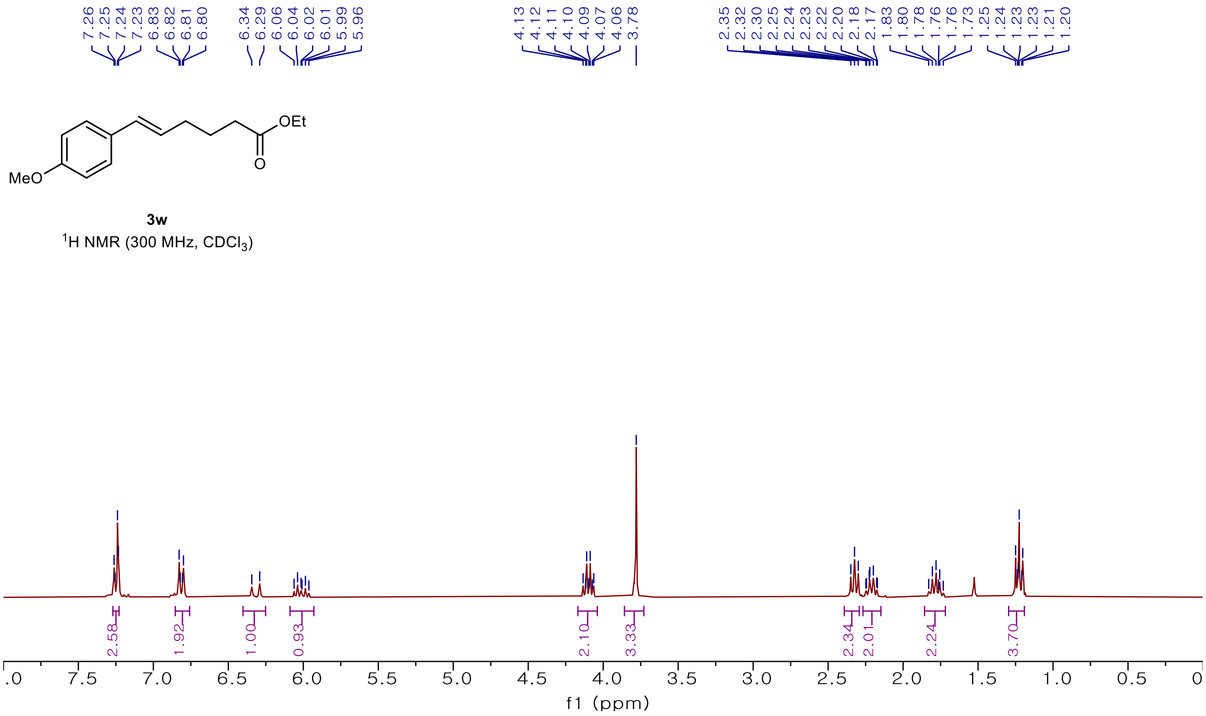

Supplementary Fig. 46. ^{1}H NMR spectrum of compound 3w.

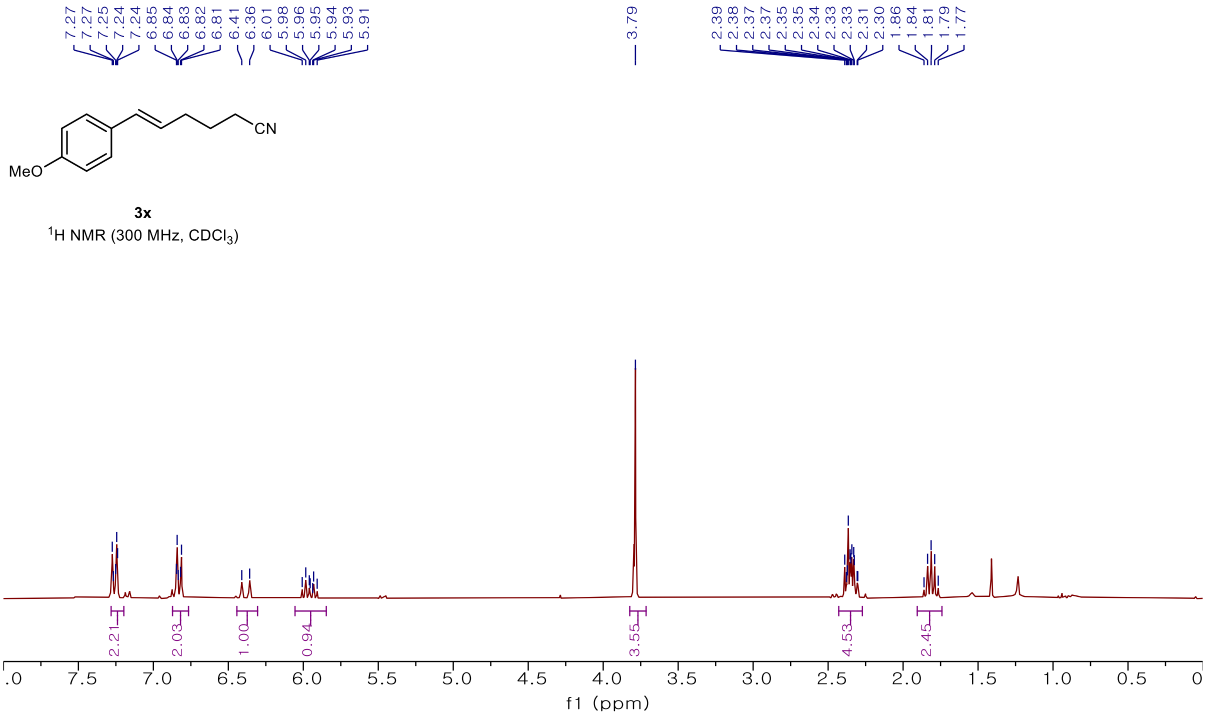

Supplementary Fig. 47. ^{1}H NMR spectrum of compound 3x.

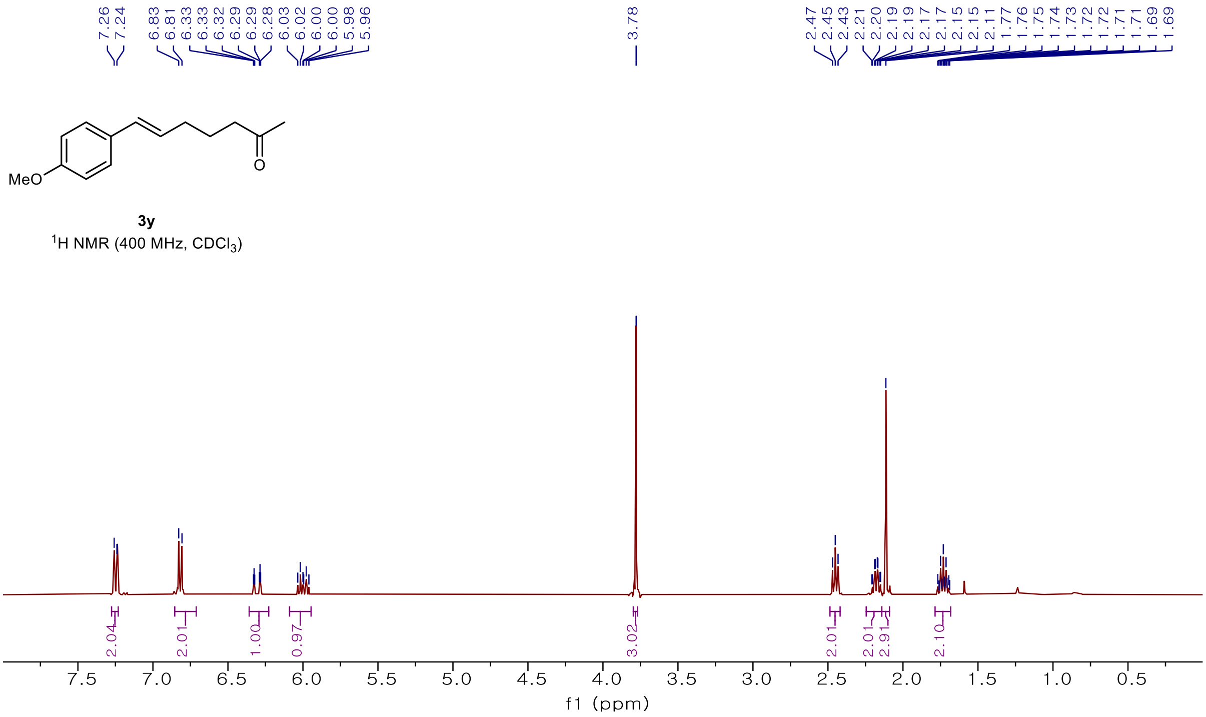

Supplementary Fig. 48. **^{1}H NMR spectrum of compound 3y.**

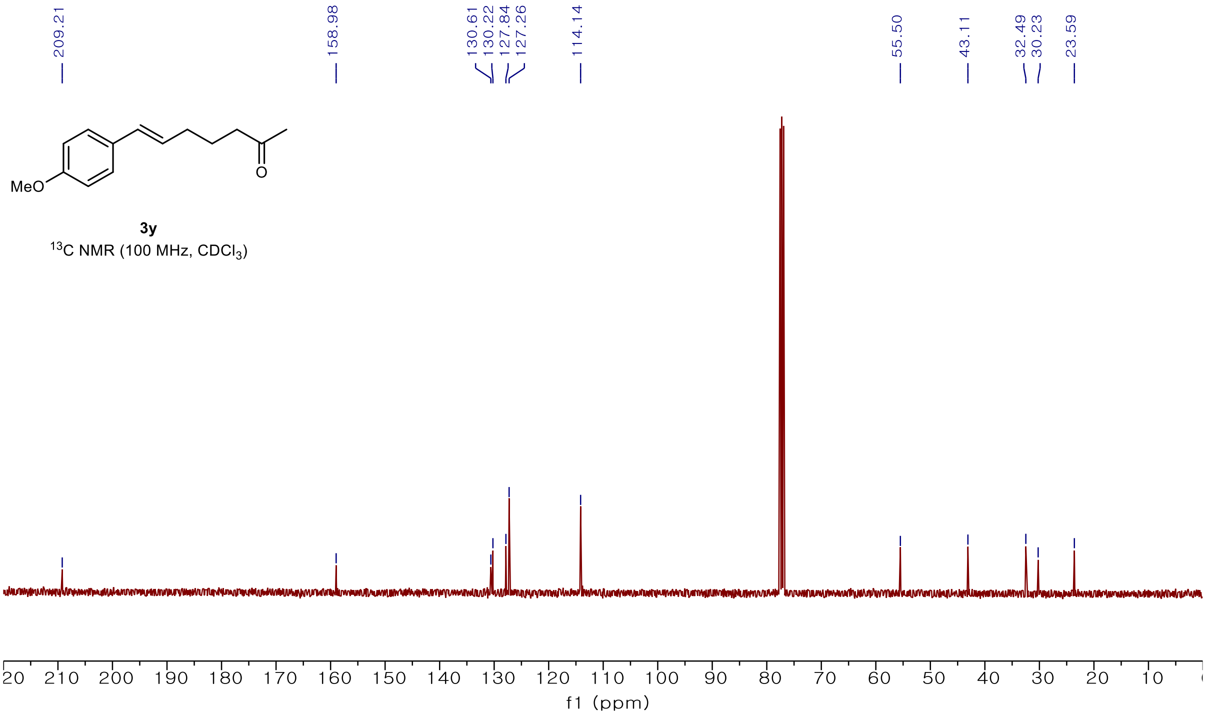

Supplementary Fig. 49. **^{13}C NMR spectrum of compound 3y.**

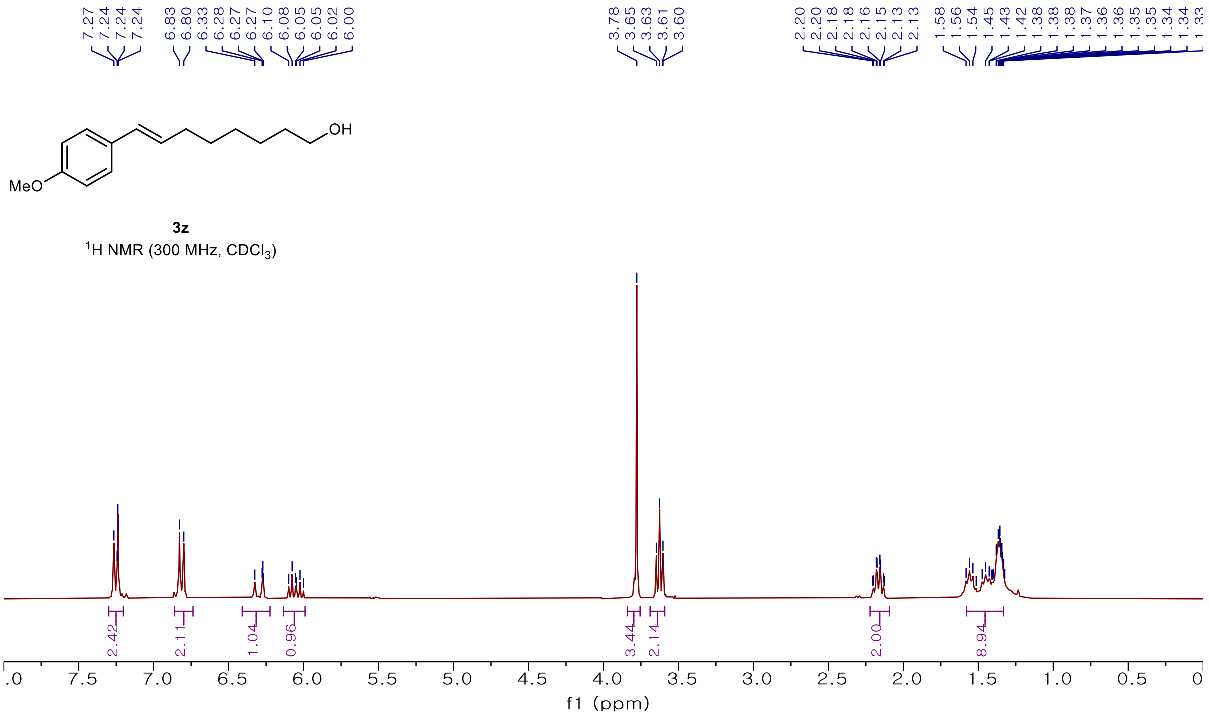

Supplementary Fig. 50. ^{1}H NMR spectrum of compound 3z.

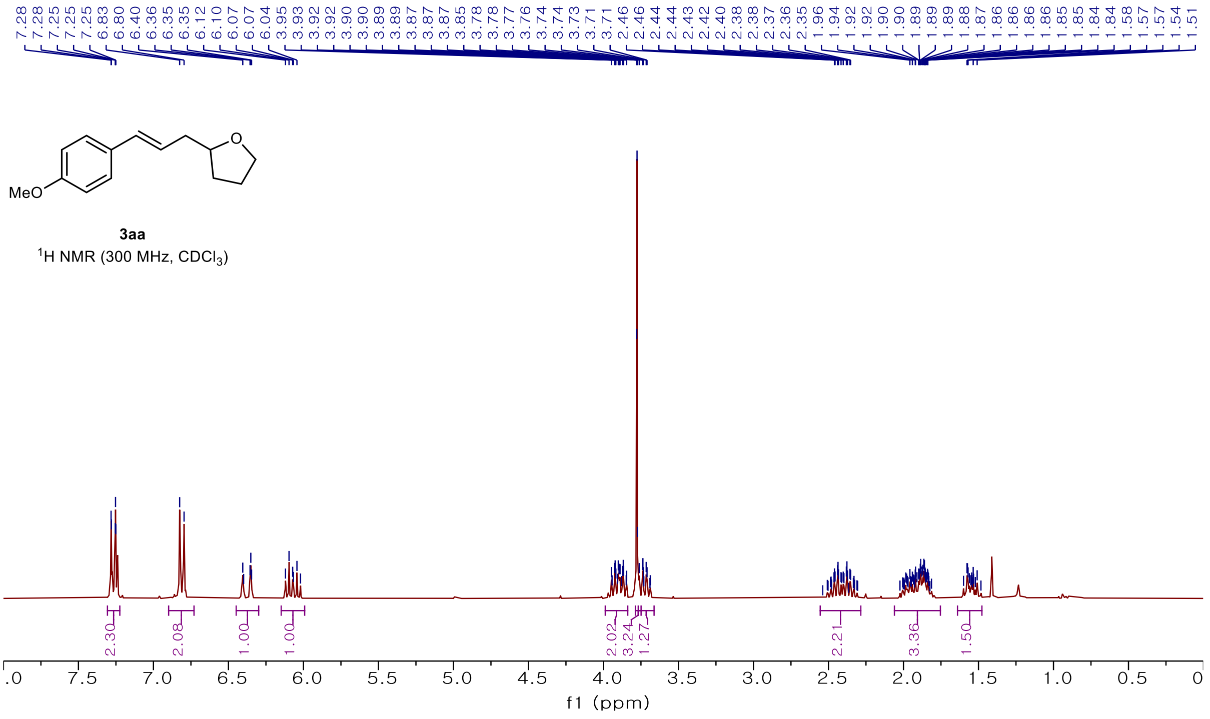

Supplementary Fig. 51. ^{1}H NMR spectrum of compound 3aa.

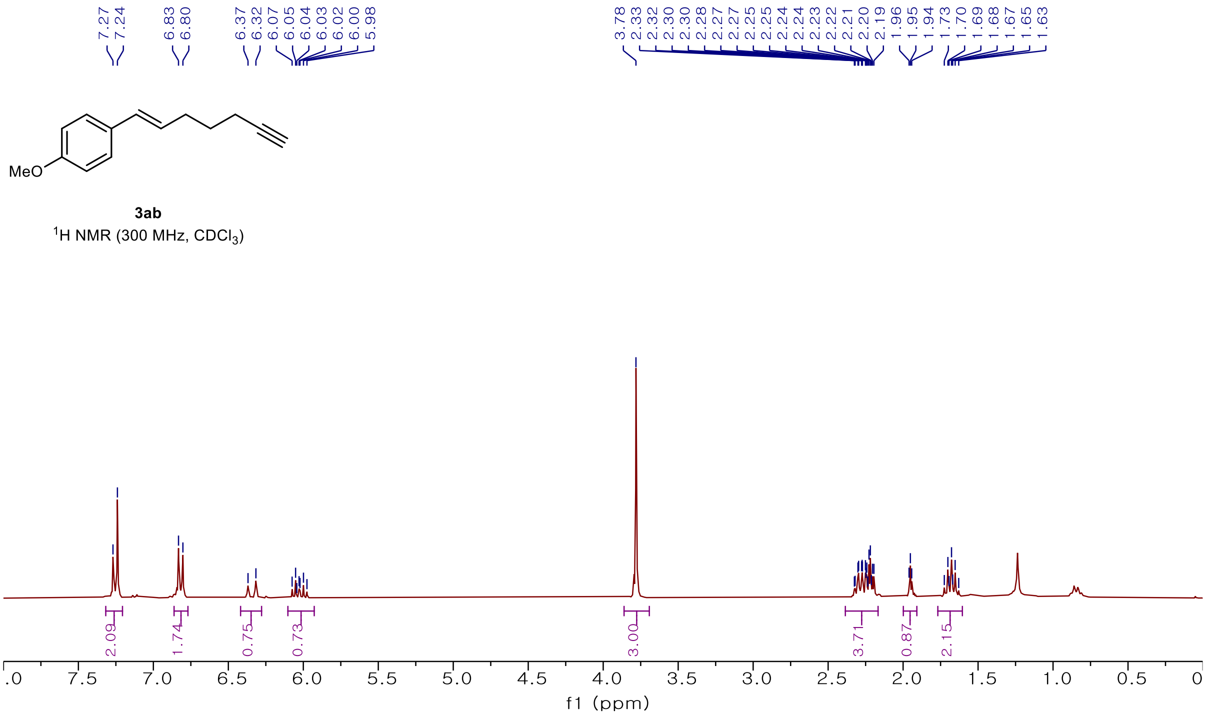

Supplementary Fig. 52. ^{1}H NMR spectrum of compound 3ab.

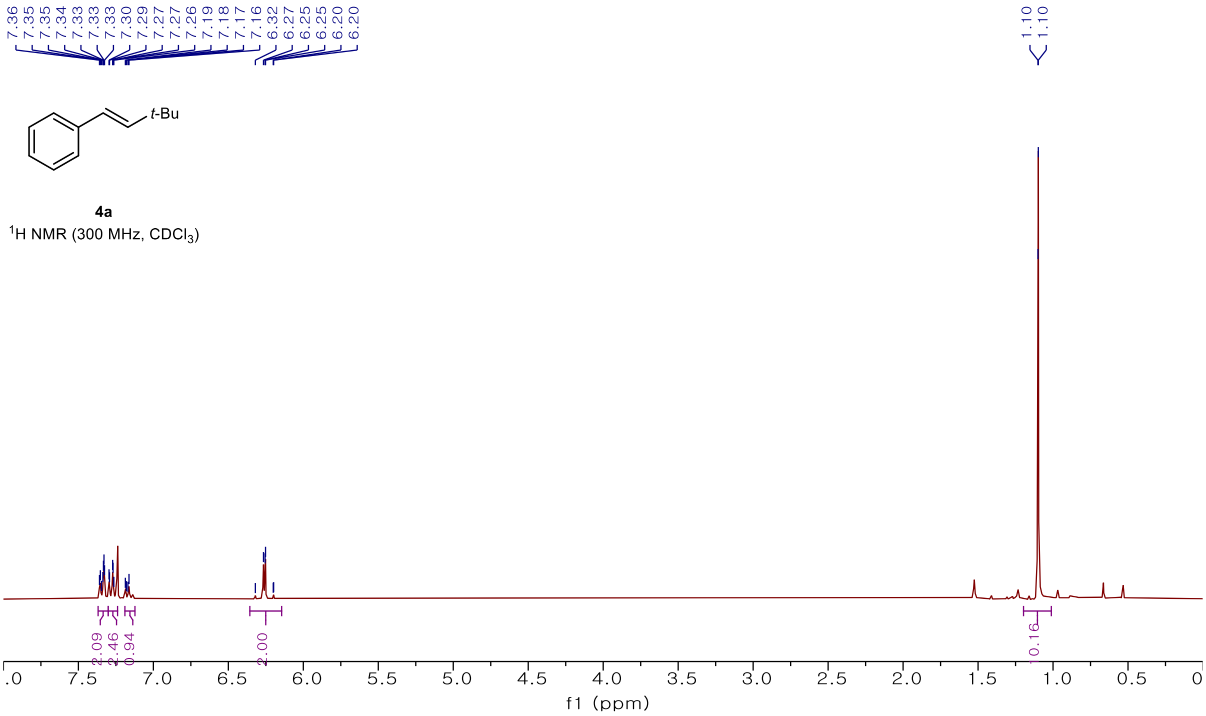

Supplementary Fig. 53. ^{1}H NMR spectrum of compound 4a.

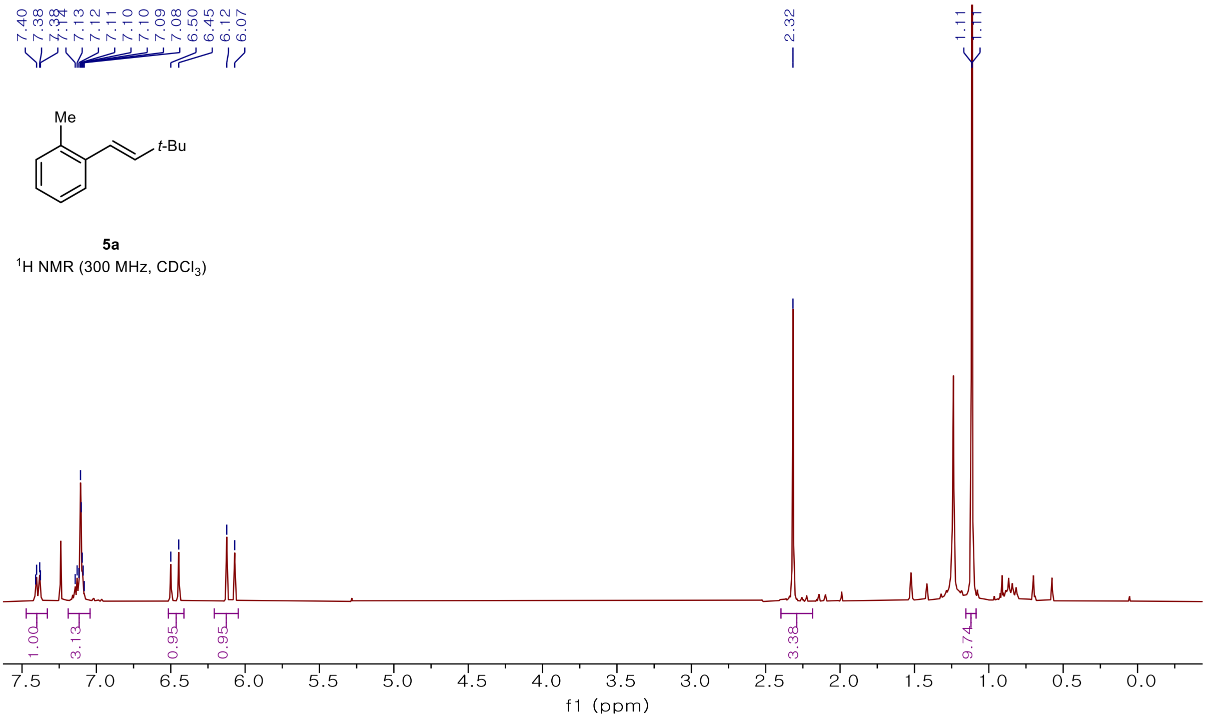

Supplementary Fig. 54. ^{1}H NMR spectrum of compound 5a.

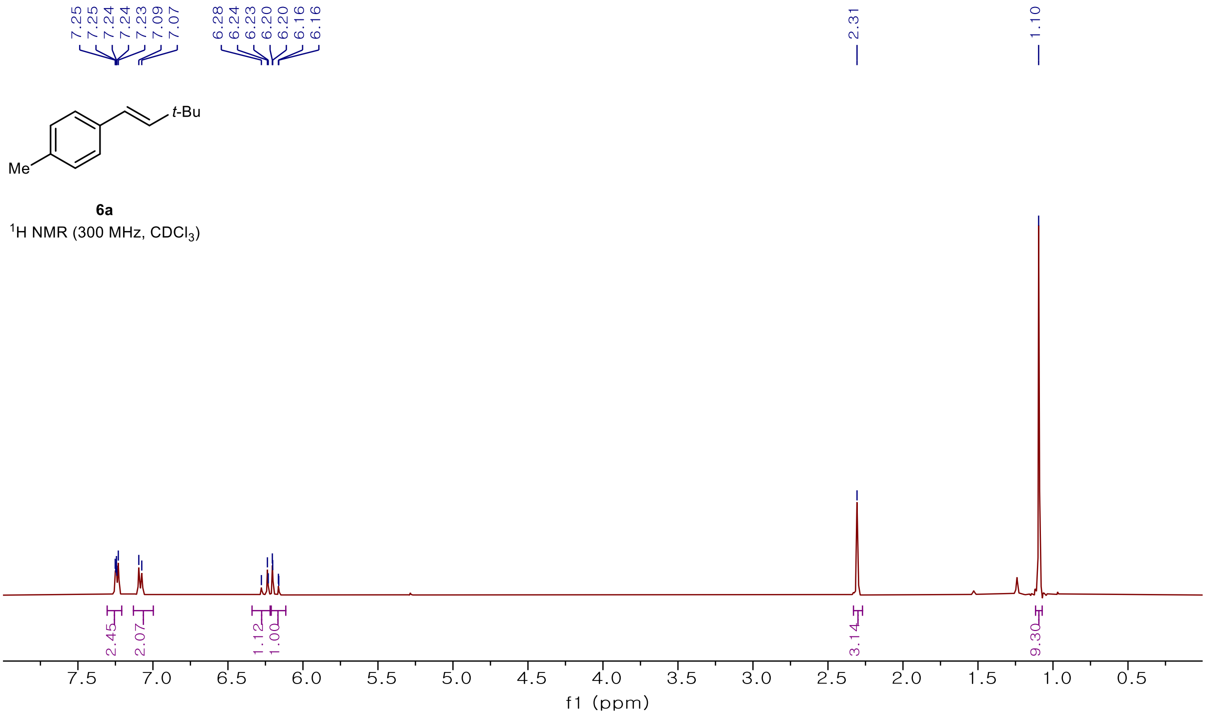

Supplementary Fig. 55. ^{1}H NMR spectrum of compound 6a.

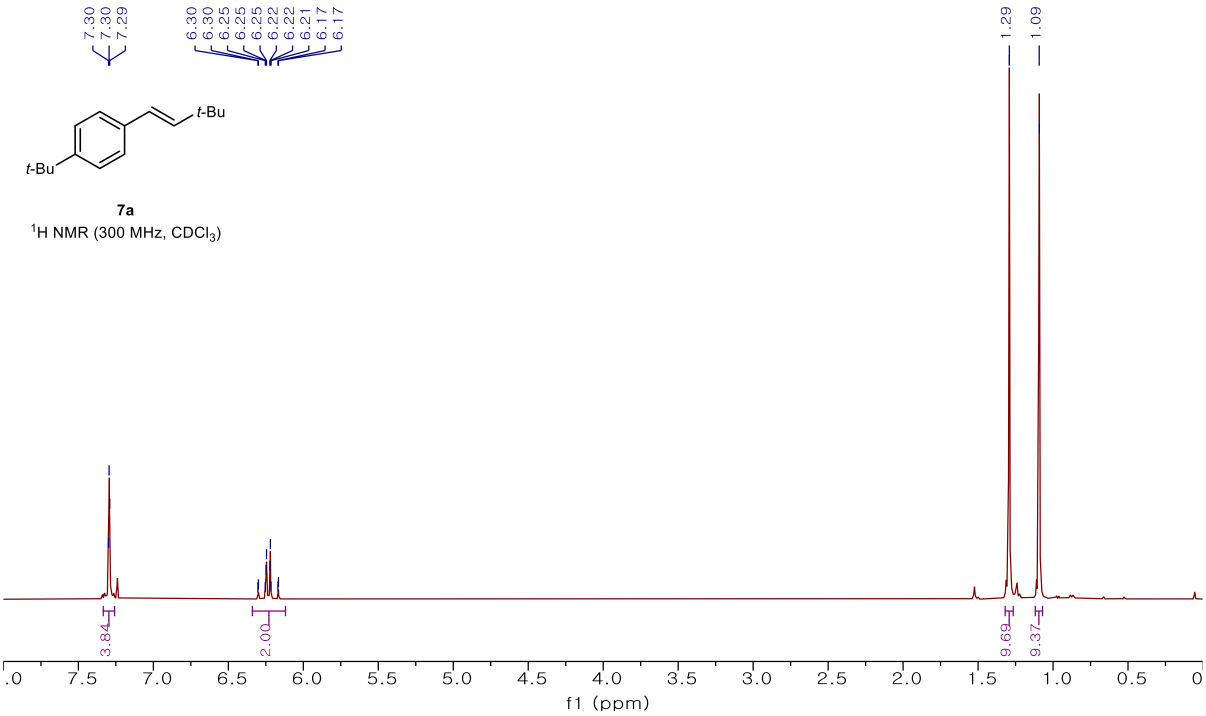

Supplementary Fig. 56. ^{1}H NMR spectrum of compound 7a.

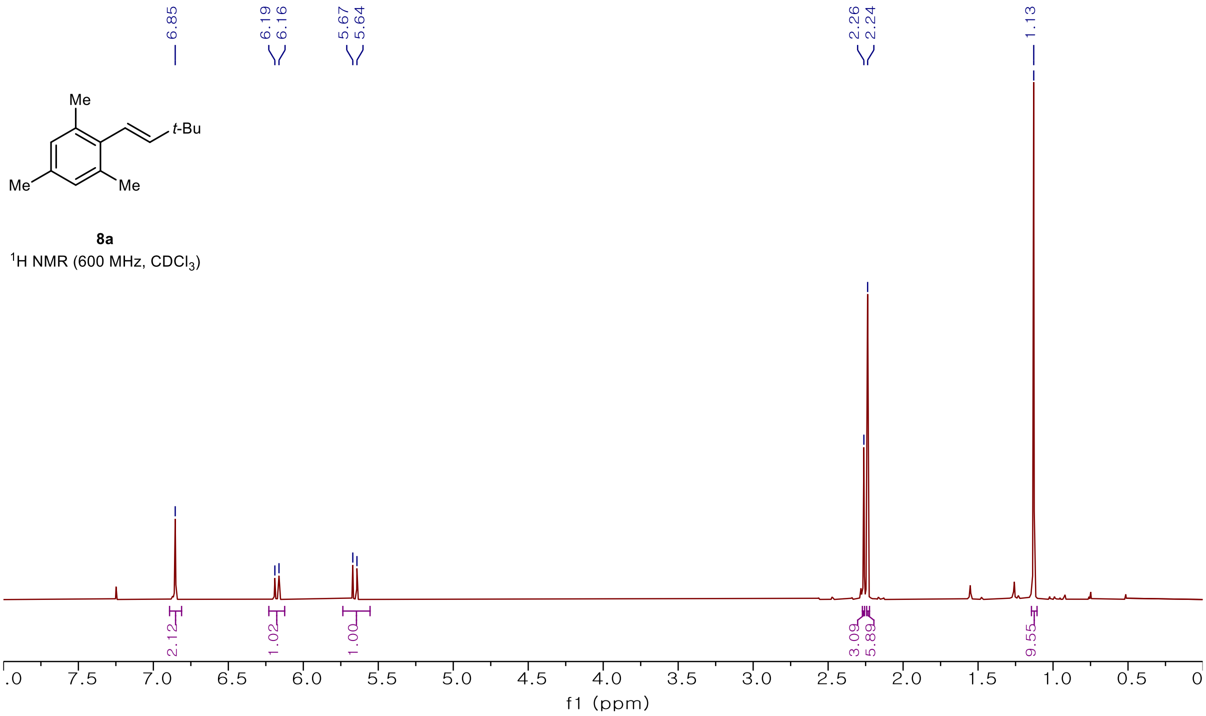

Supplementary Fig. 57. ^{1}H NMR spectrum of compound 8a.

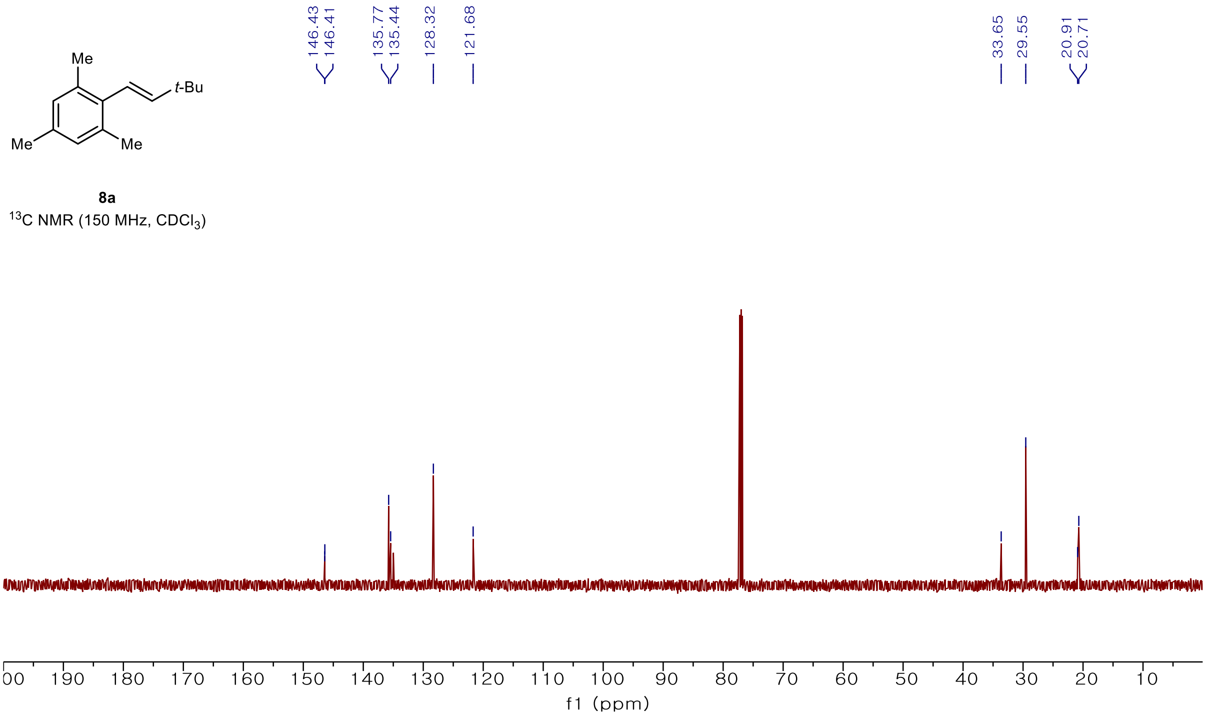

Supplementary Fig. 58. ^{13}C NMR spectrum of compound 8a.

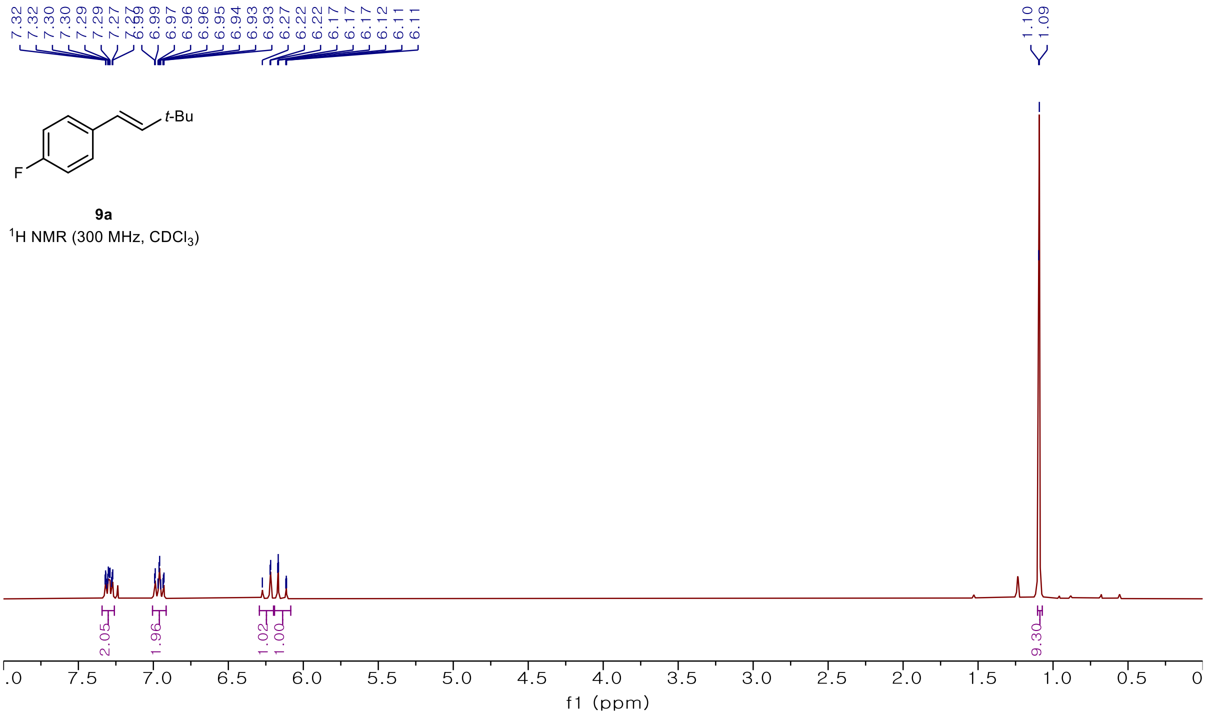

Supplementary Fig. 59. ^{1}H NMR spectrum of compound 9a.

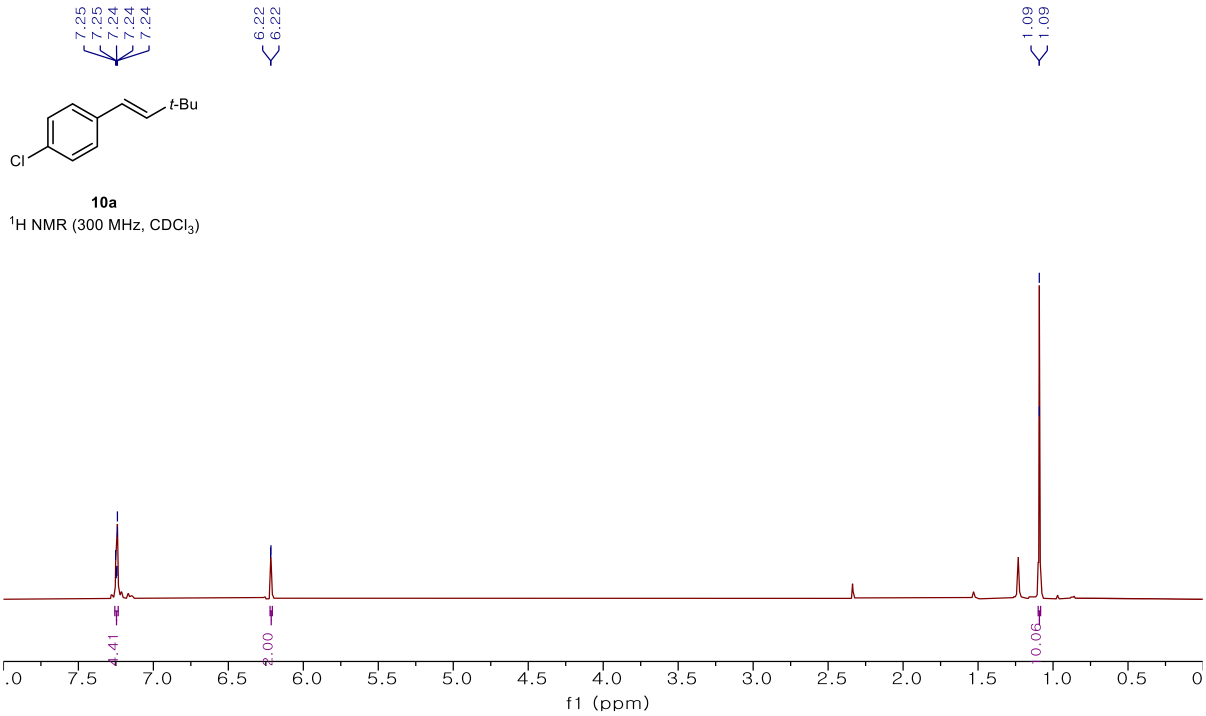

Supplementary Fig. 60. ^{1}H NMR spectrum of compound 10a.

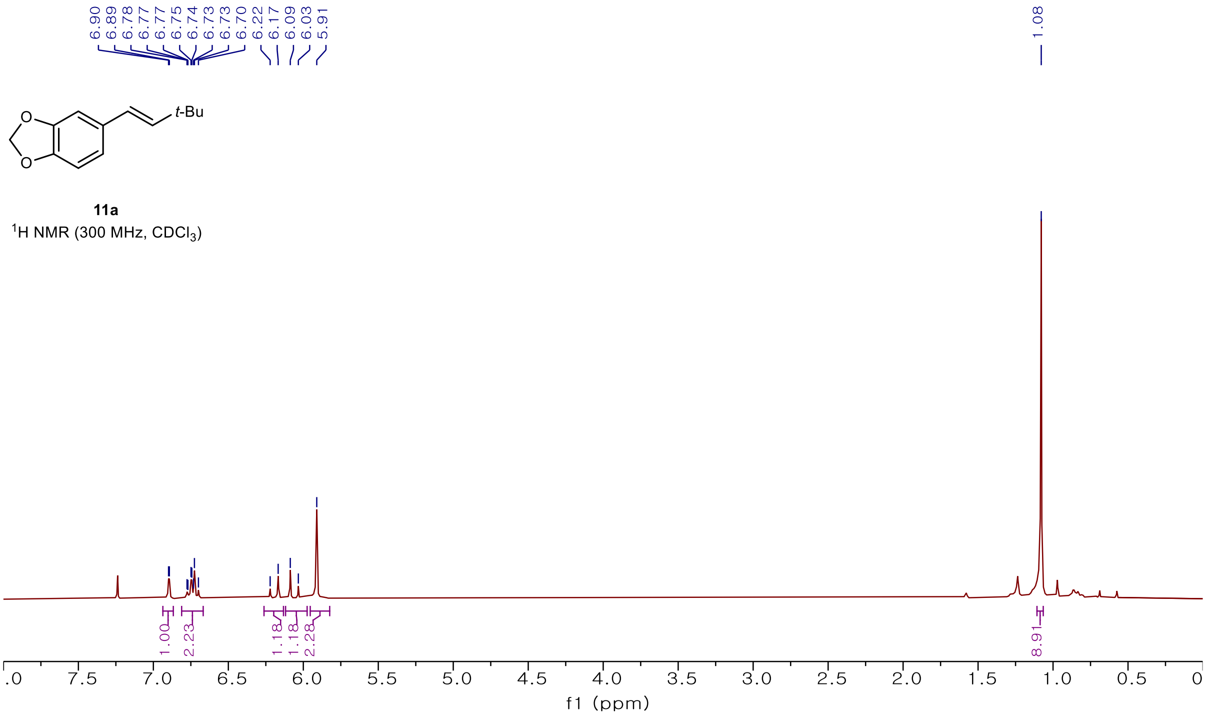

Supplementary Fig. 61. ^{1}H NMR spectrum of compound 11a.

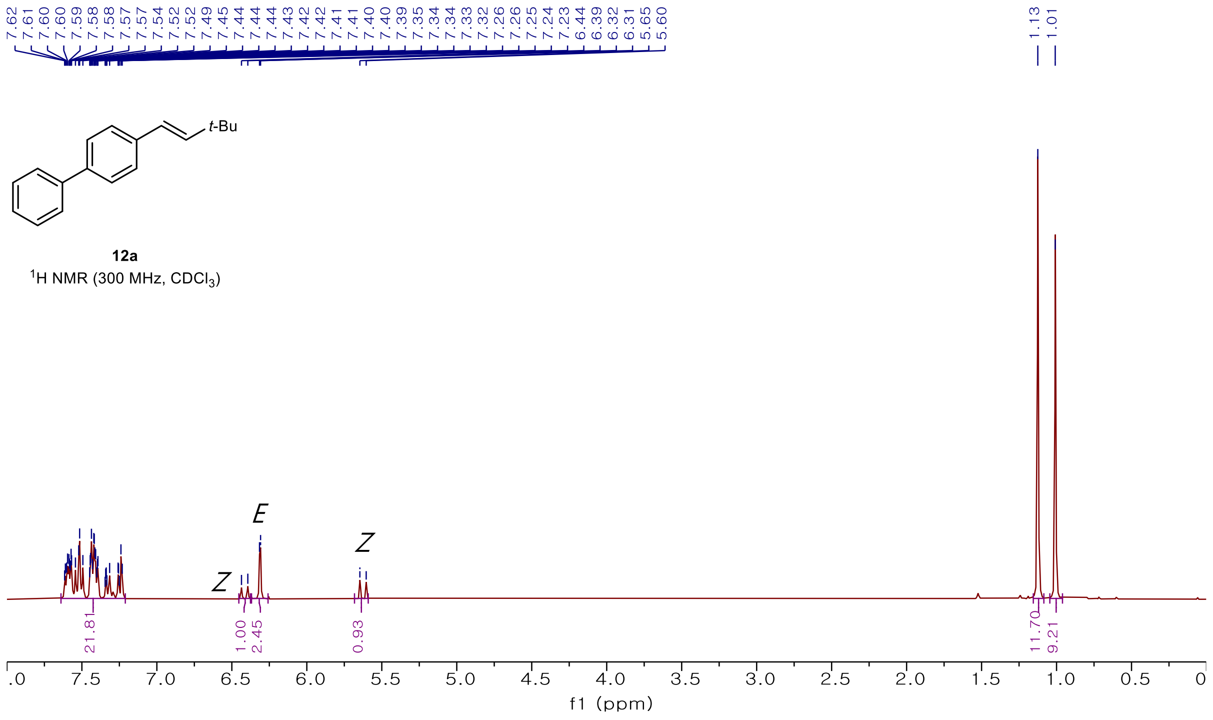

Supplementary Fig. 62. ^{1}H NMR spectrum of compound 12a.

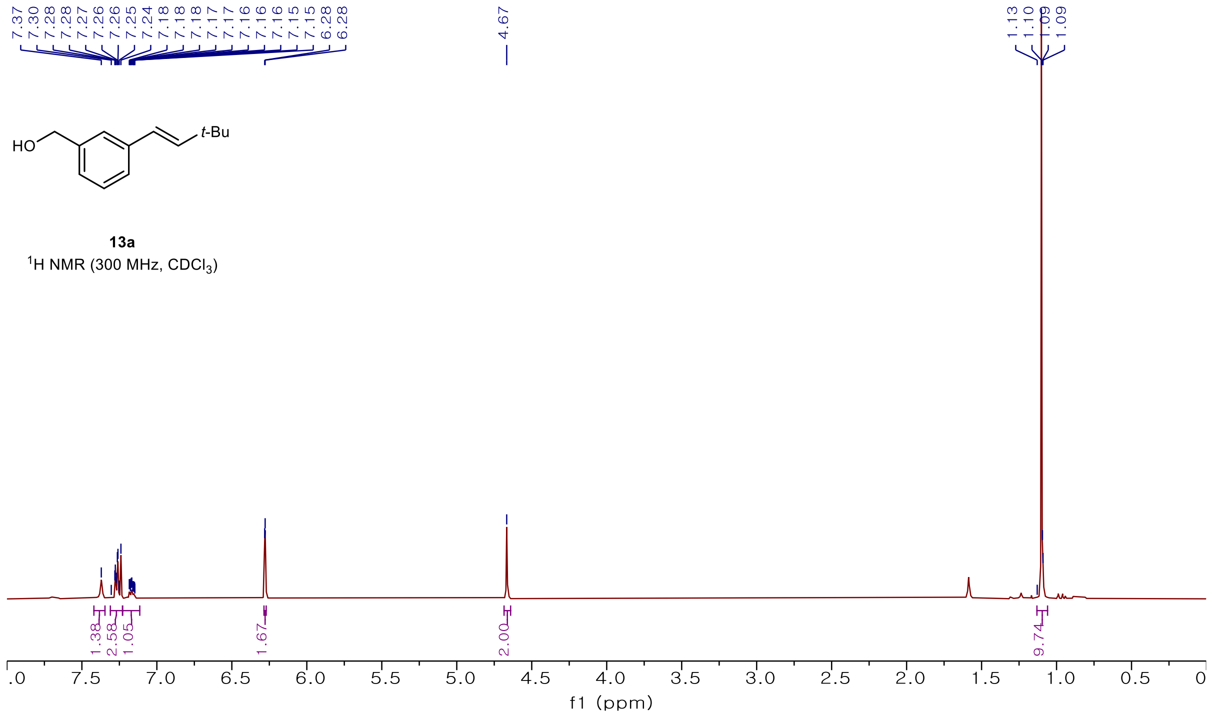

Supplementary Fig. 63. ^{1}H NMR spectrum of compound 13a.

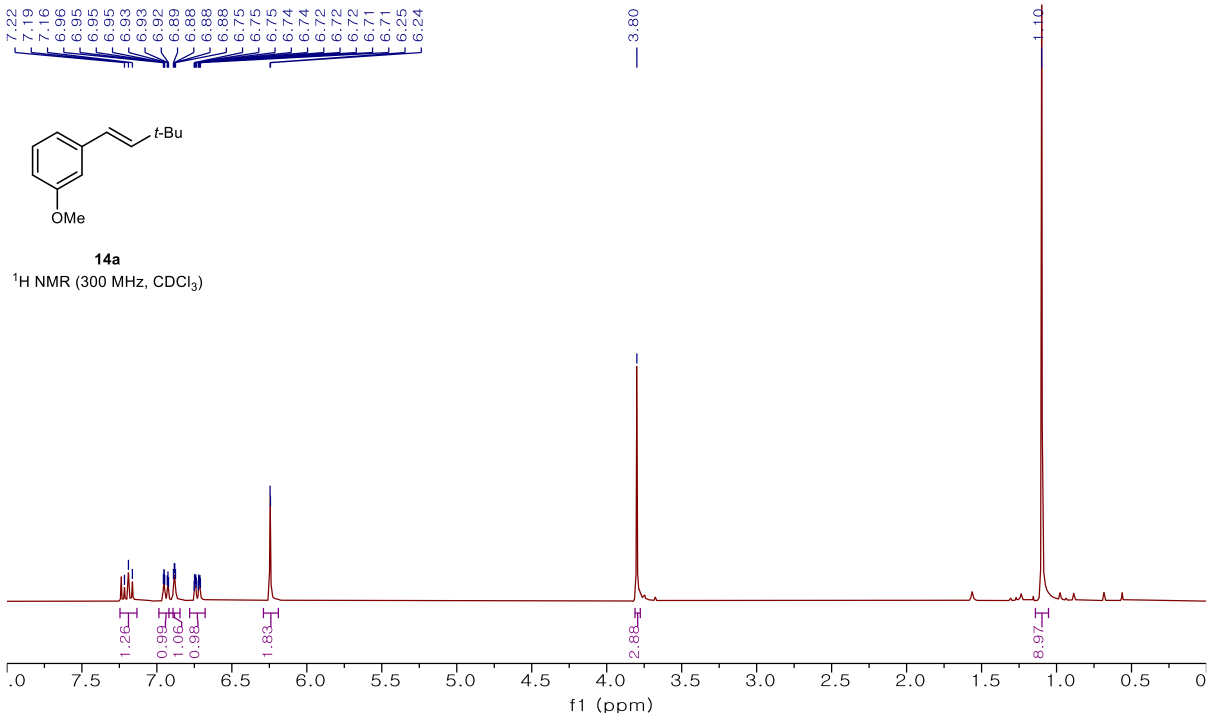

Supplementary Fig. 64. ^{1}**H NMR spectrum of compound 14a.**

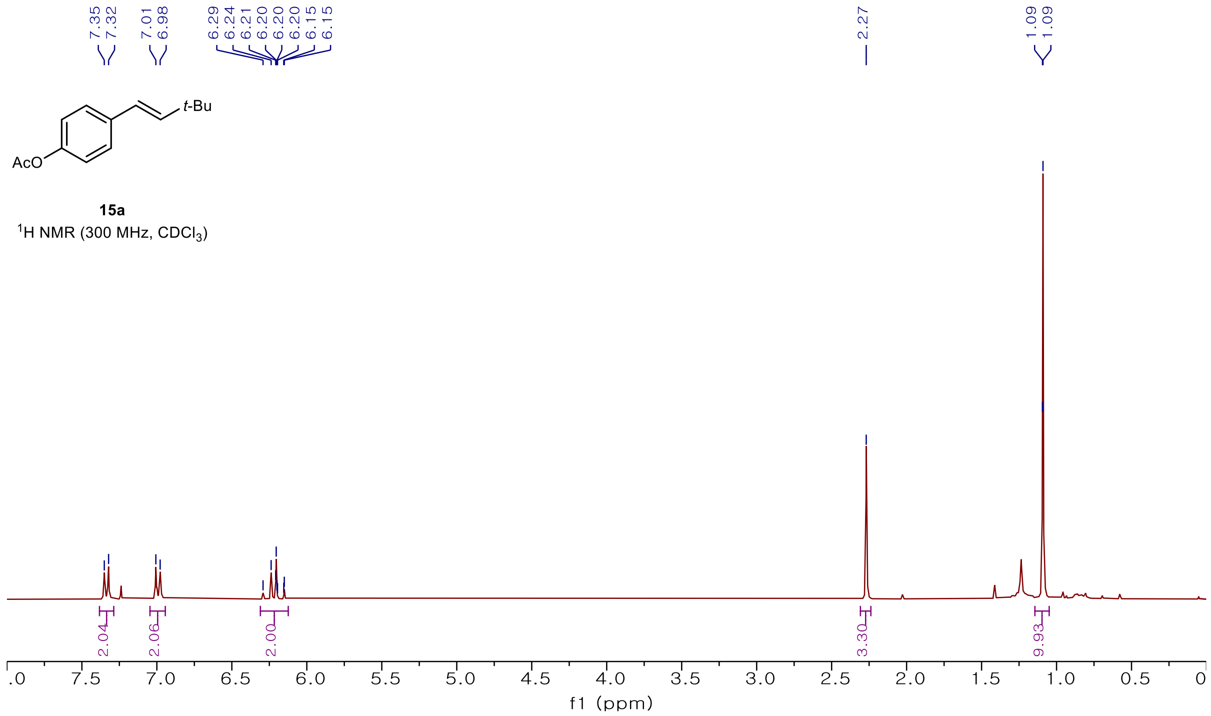

Supplementary Fig. 65. ^{1}**H NMR spectrum of compound 15a.**

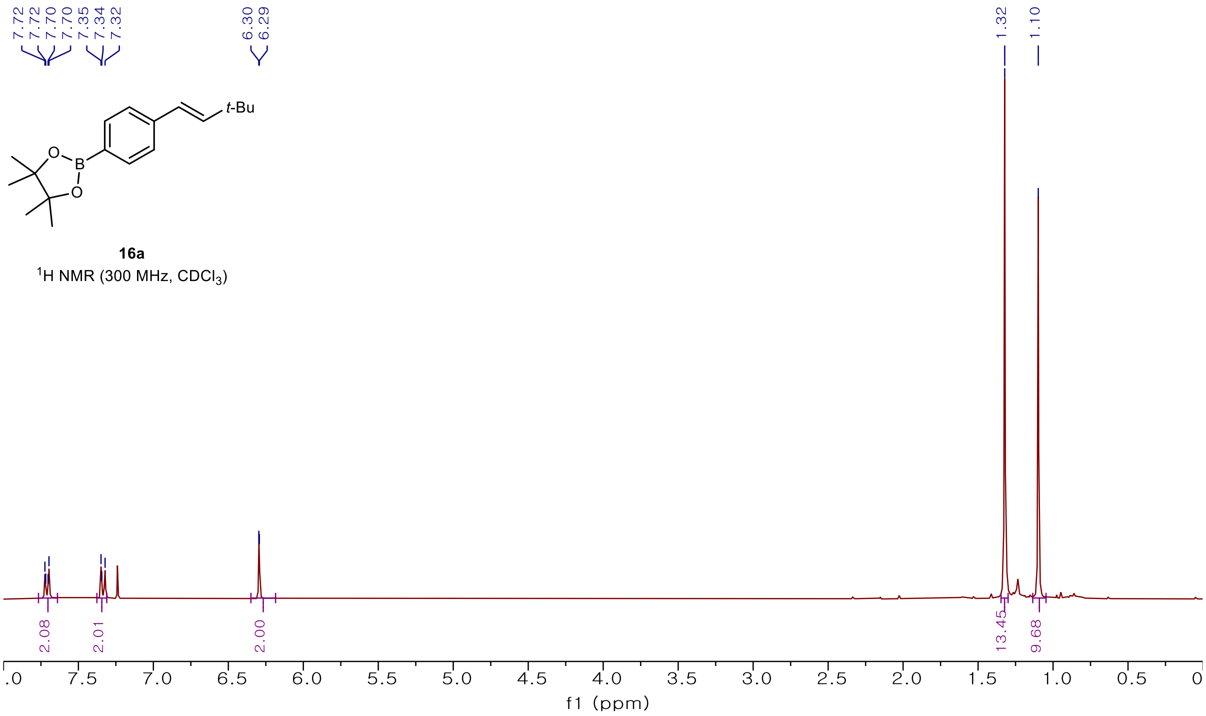

Supplementary Fig. 66. ^{1}H NMR spectrum of compound 16a.

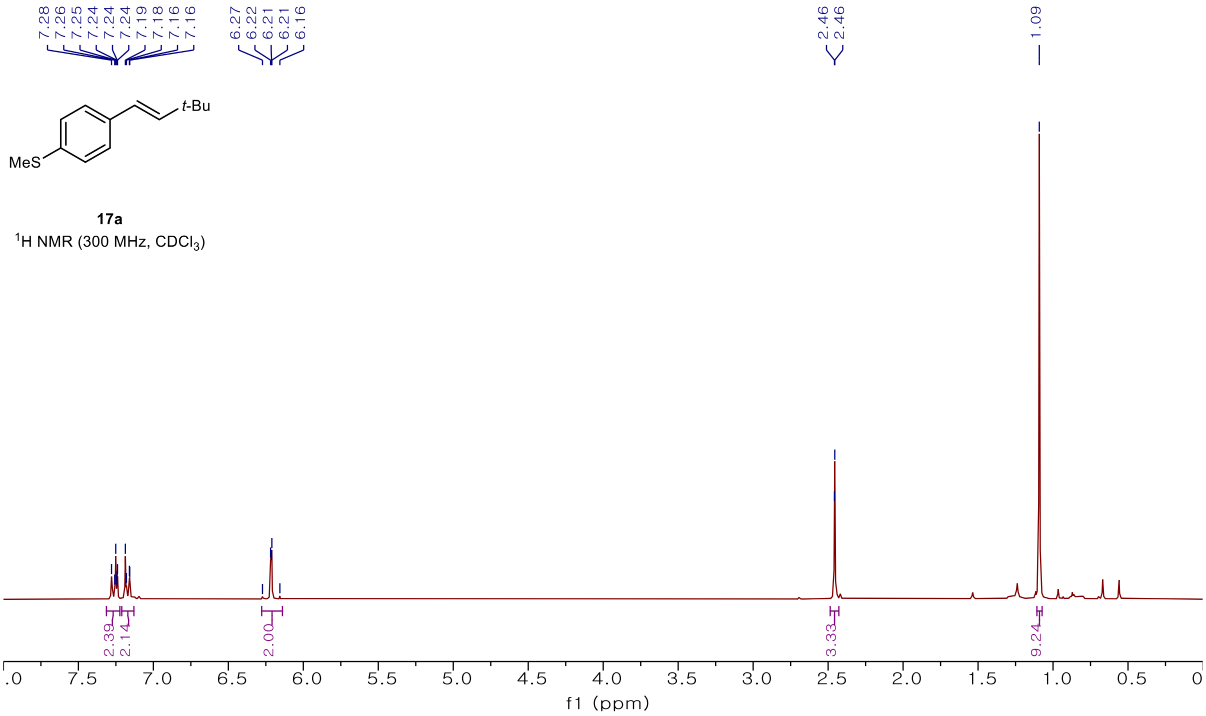

Supplementary Fig. 67. ^{1}H NMR spectrum of compound 17a.

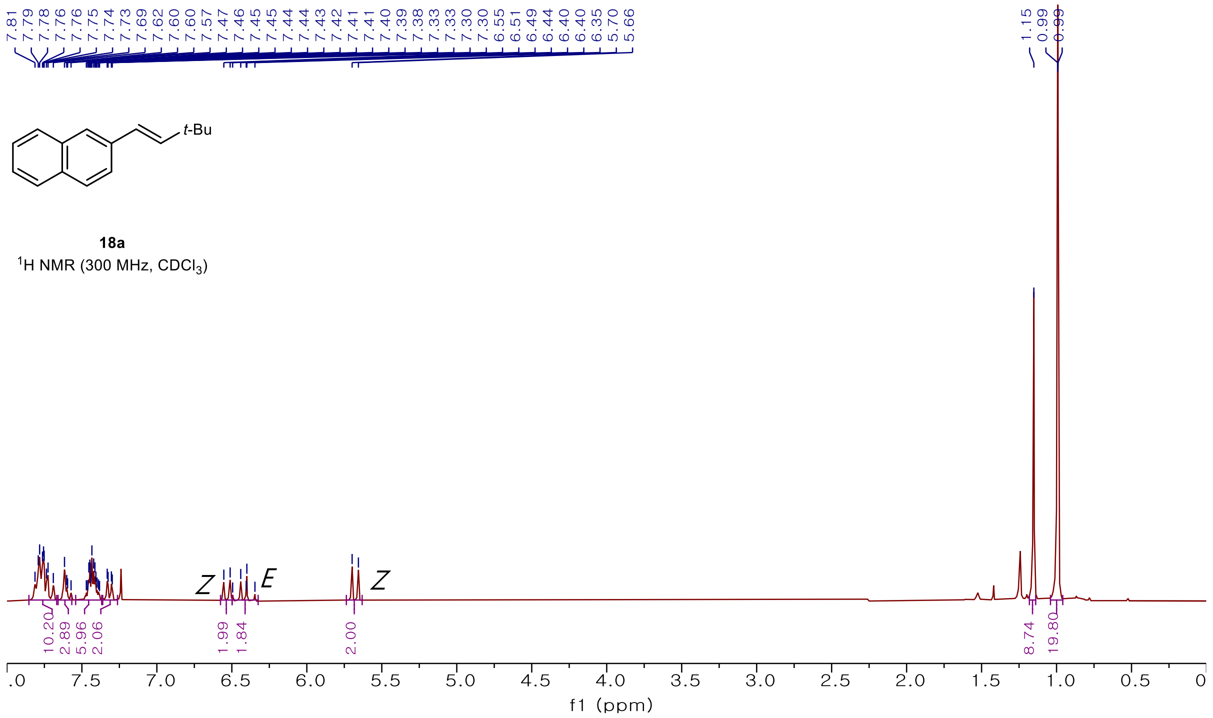

Supplementary Fig. 68. ^{1}H NMR spectrum of compound 18a.

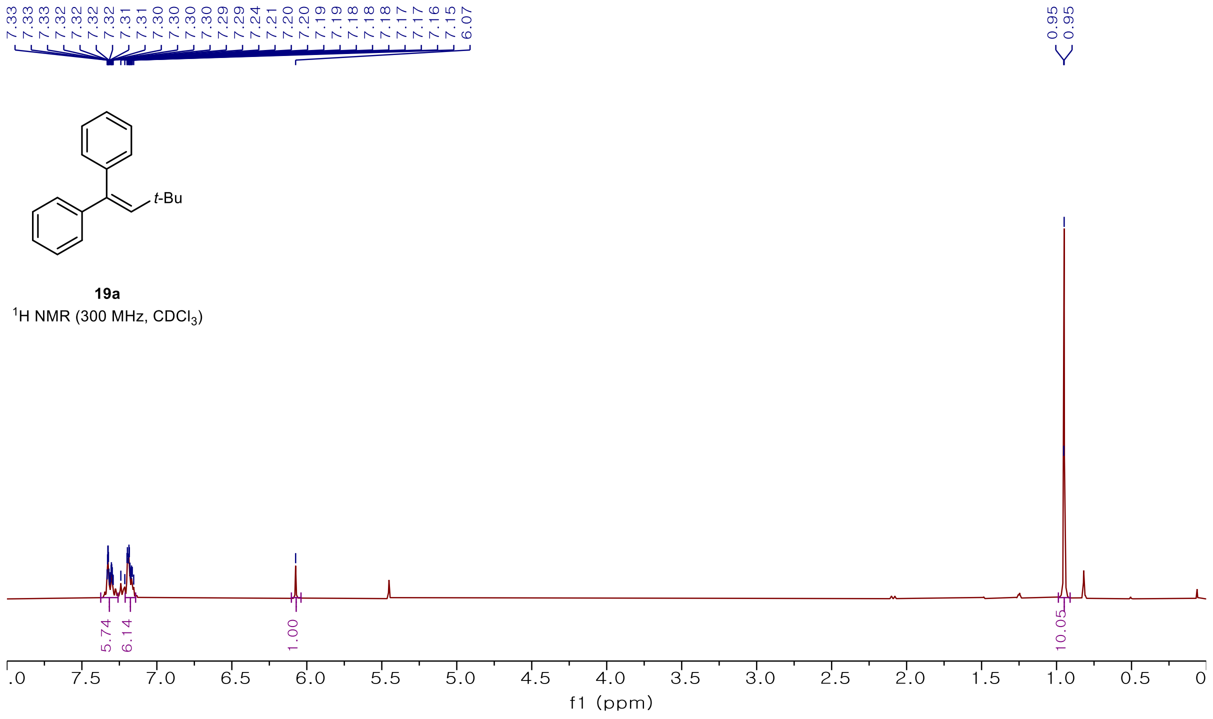

Supplementary Fig. 69. ^{1}H NMR spectrum of compound 19a.

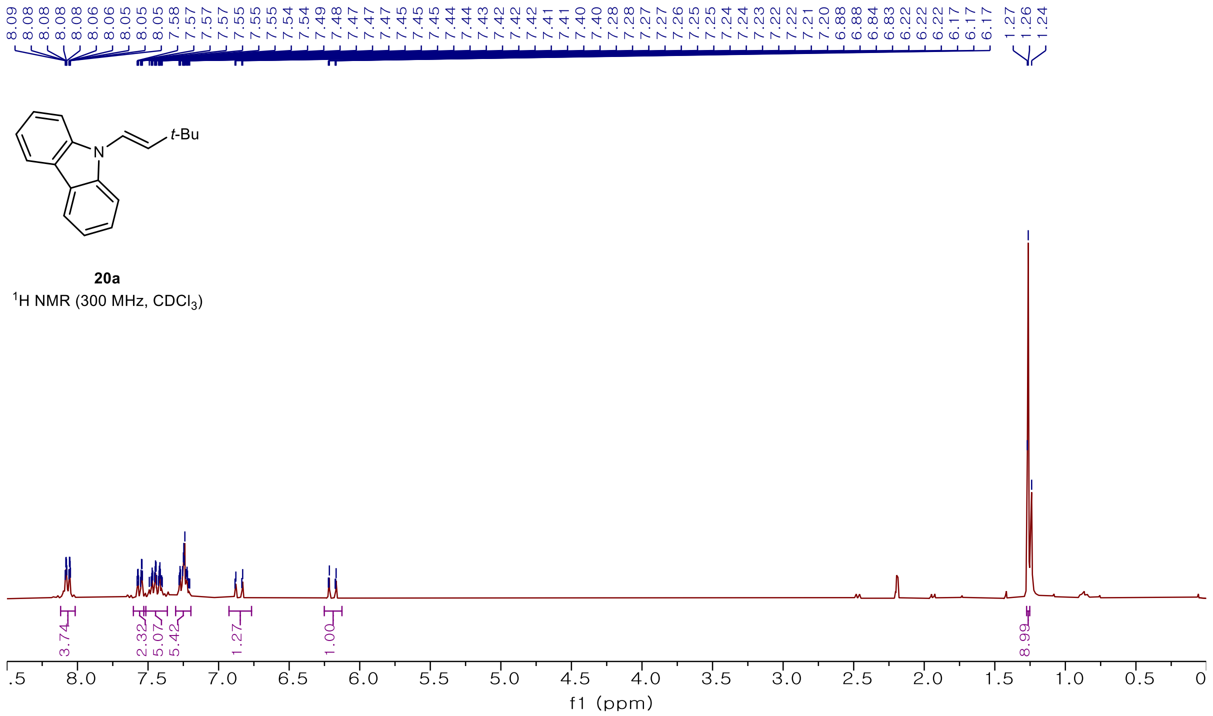

Supplementary Fig. 70. ^{1}H NMR spectrum of compound 20a.

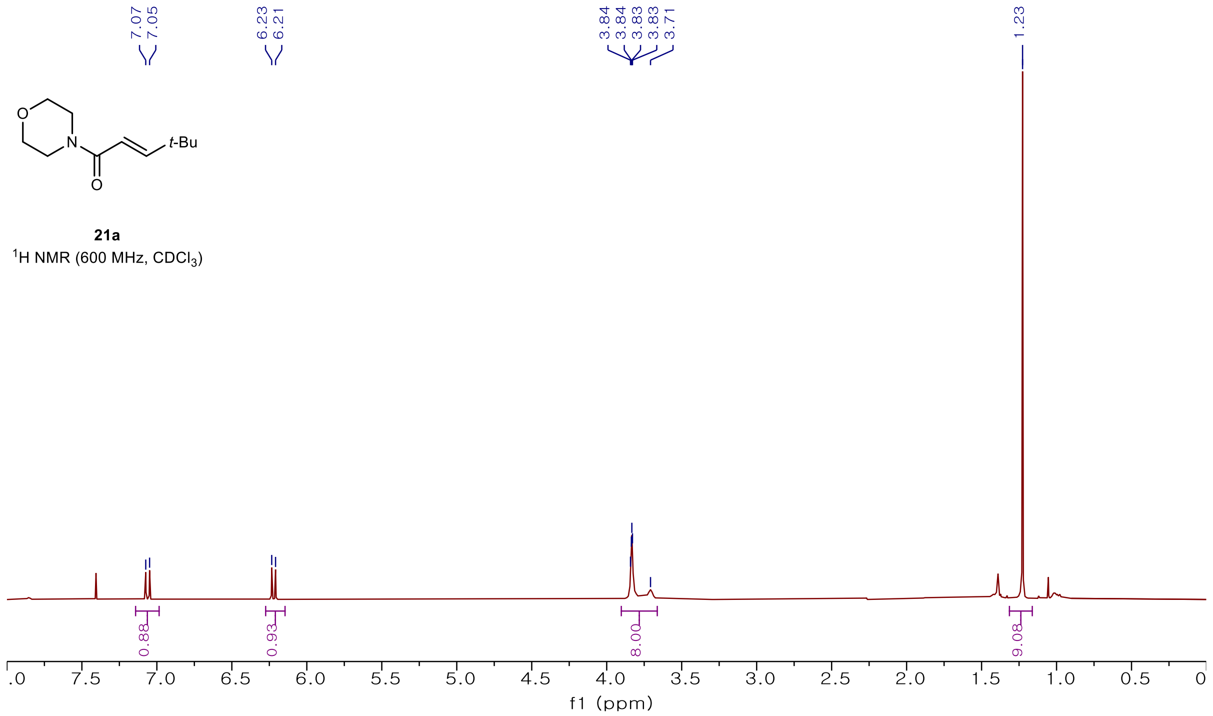

Supplementary Fig. 71. ^{1}H NMR spectrum of compound 21a.

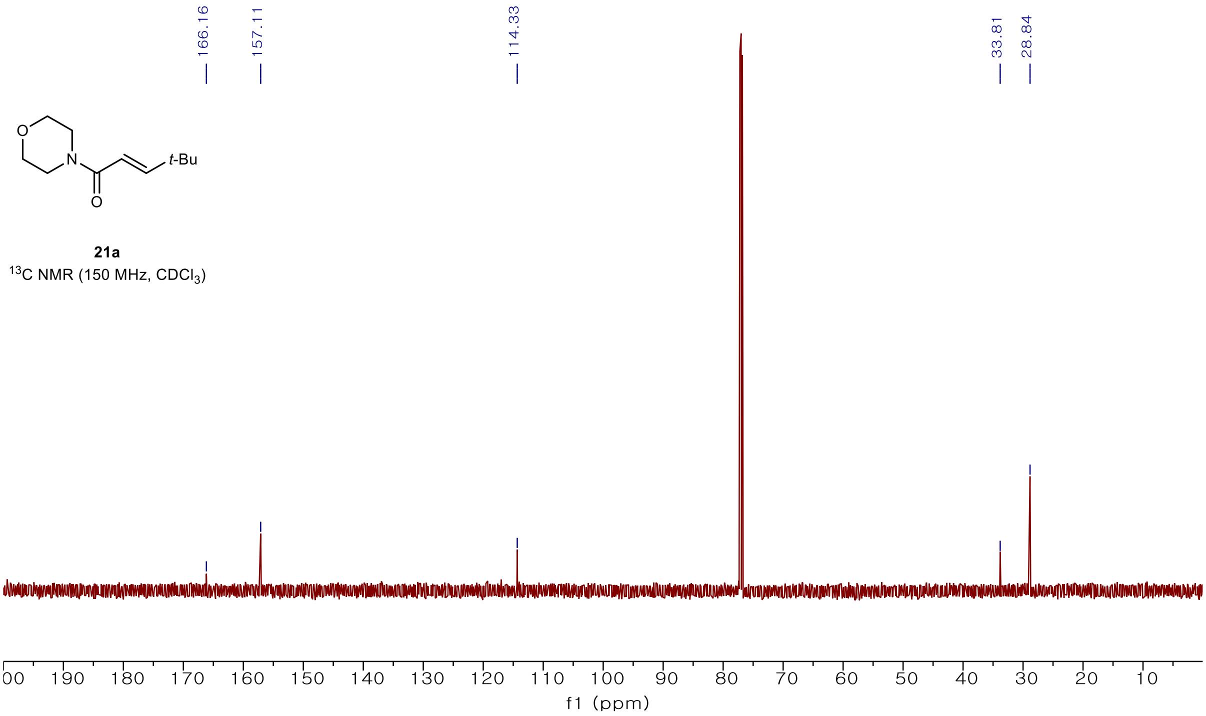

Supplementary Fig. 72. ^{13}C NMR spectrum of compound 21a.

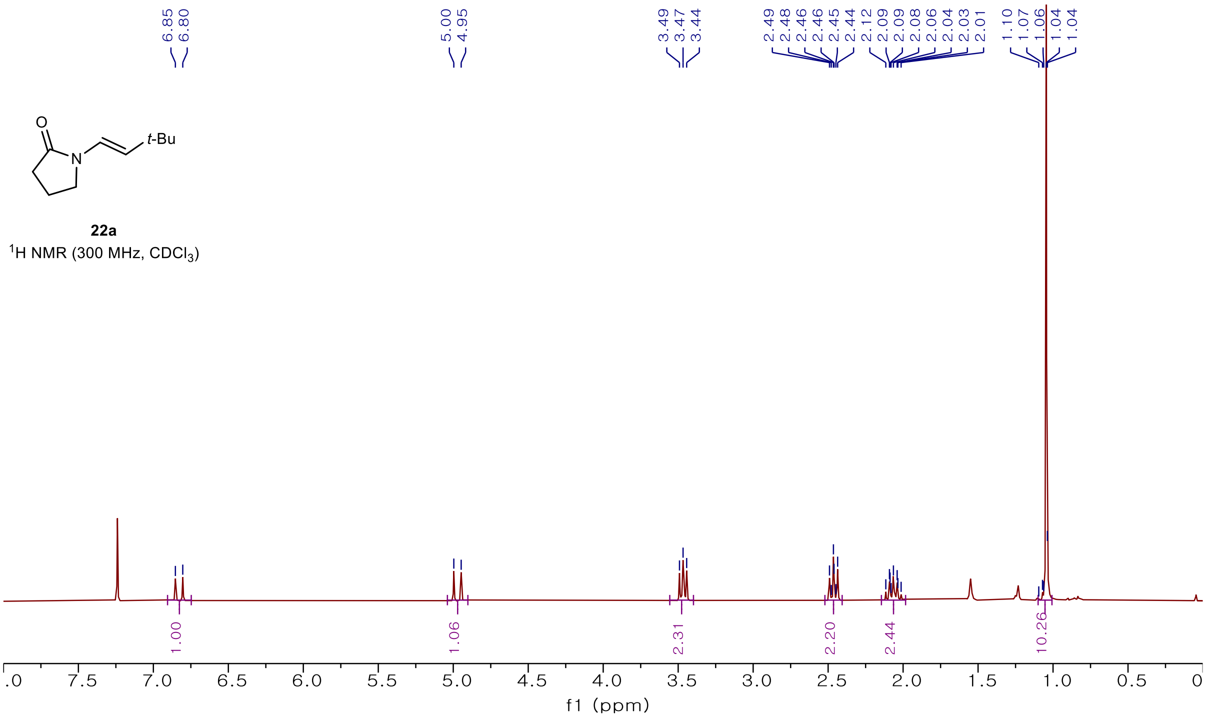

Supplementary Fig. 73. ^{1}H NMR spectrum of compound 22a.

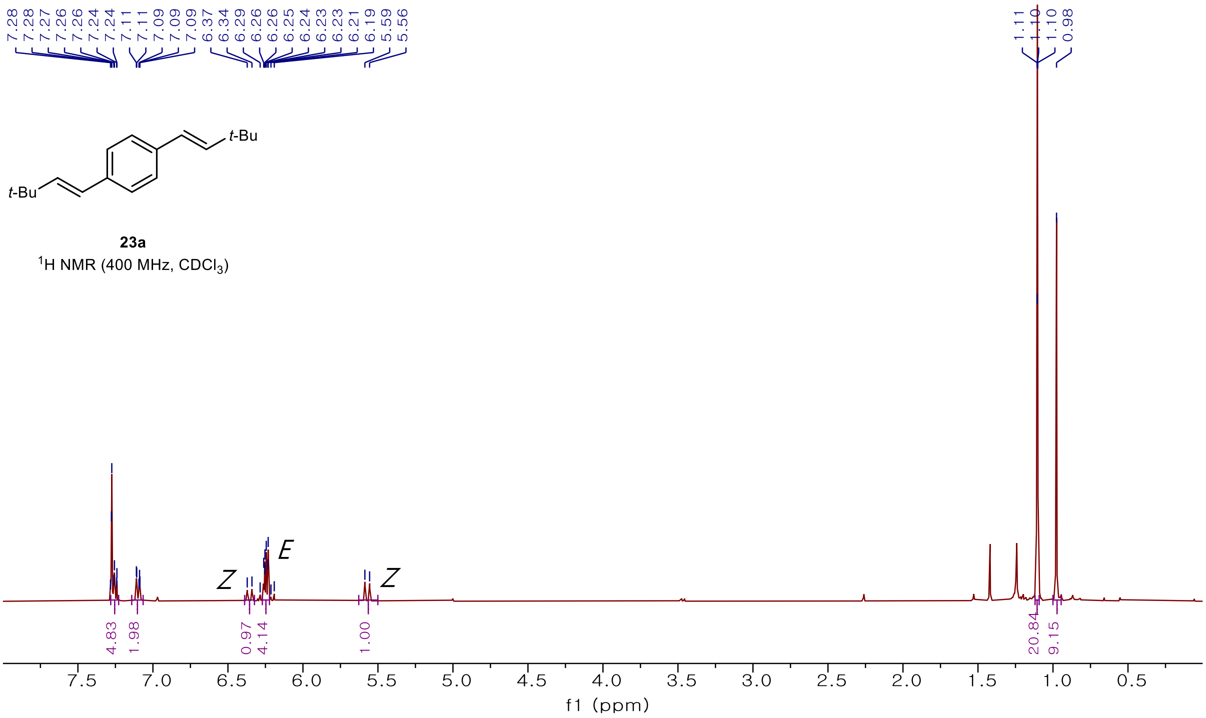

Supplementary Fig. 74. ^{1}H NMR spectrum of compound 23a.

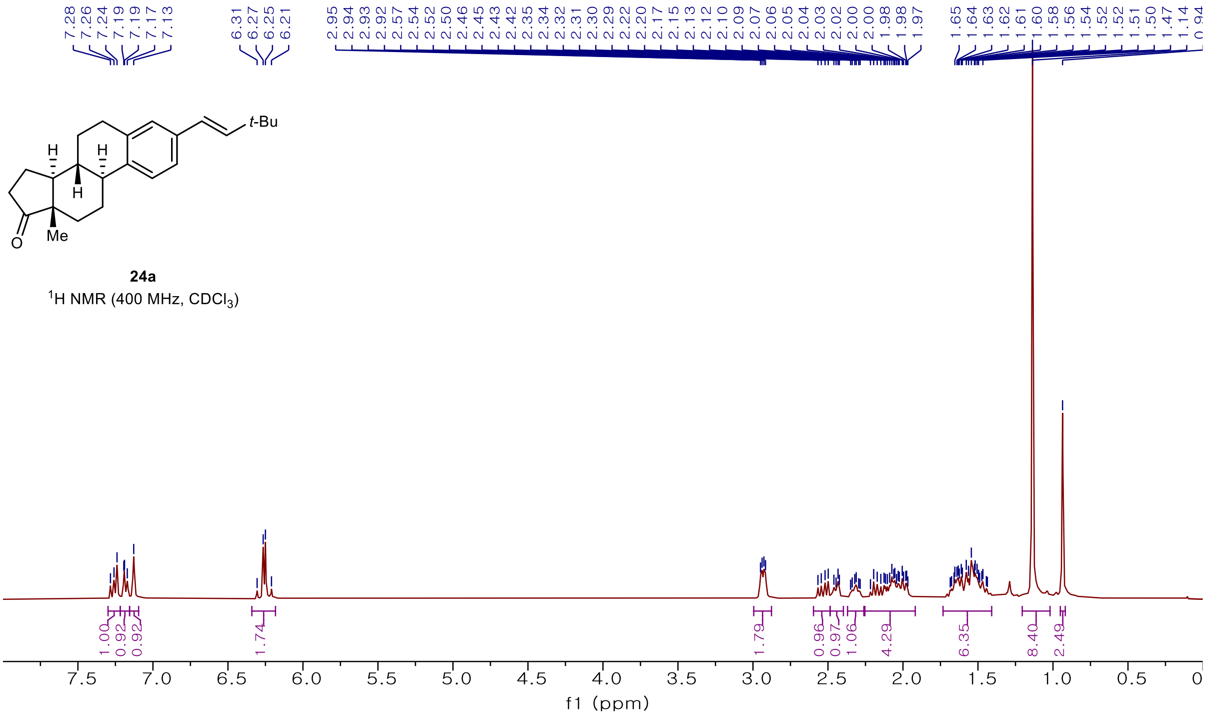

Supplementary Fig. 75. ^{1}H NMR spectrum of compound 24a.

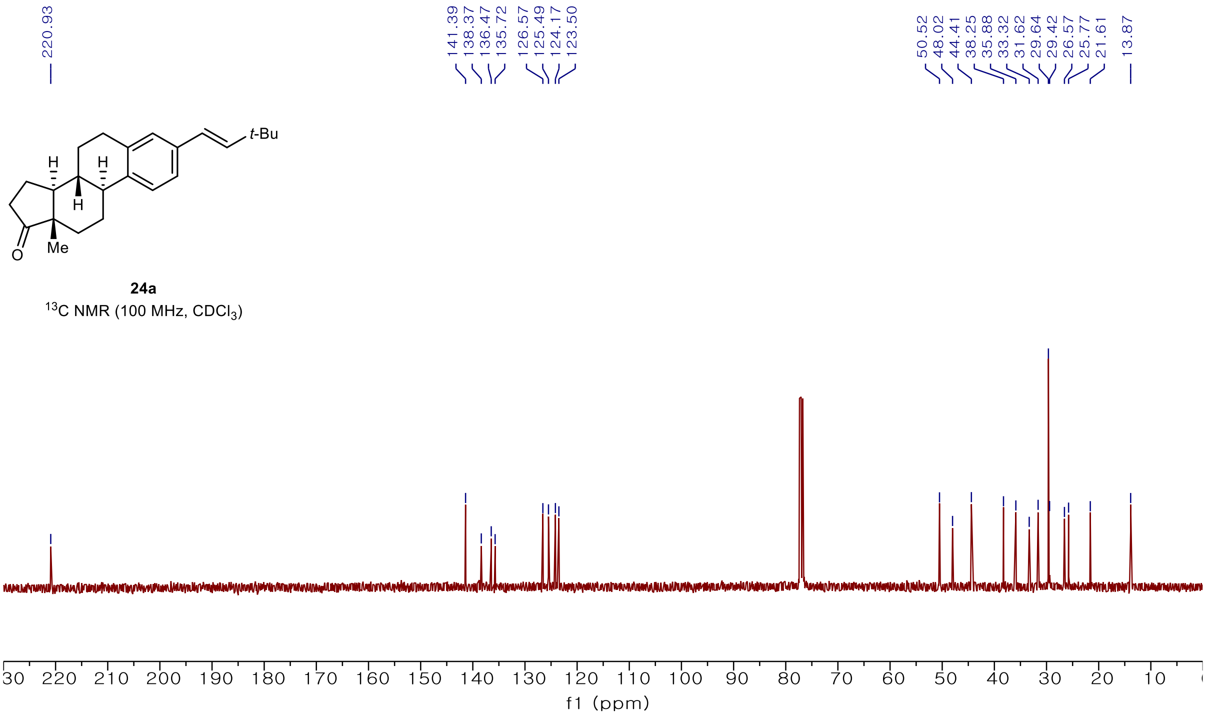

Supplementary Fig. 76. ^{13}C NMR spectrum of compound 24a.

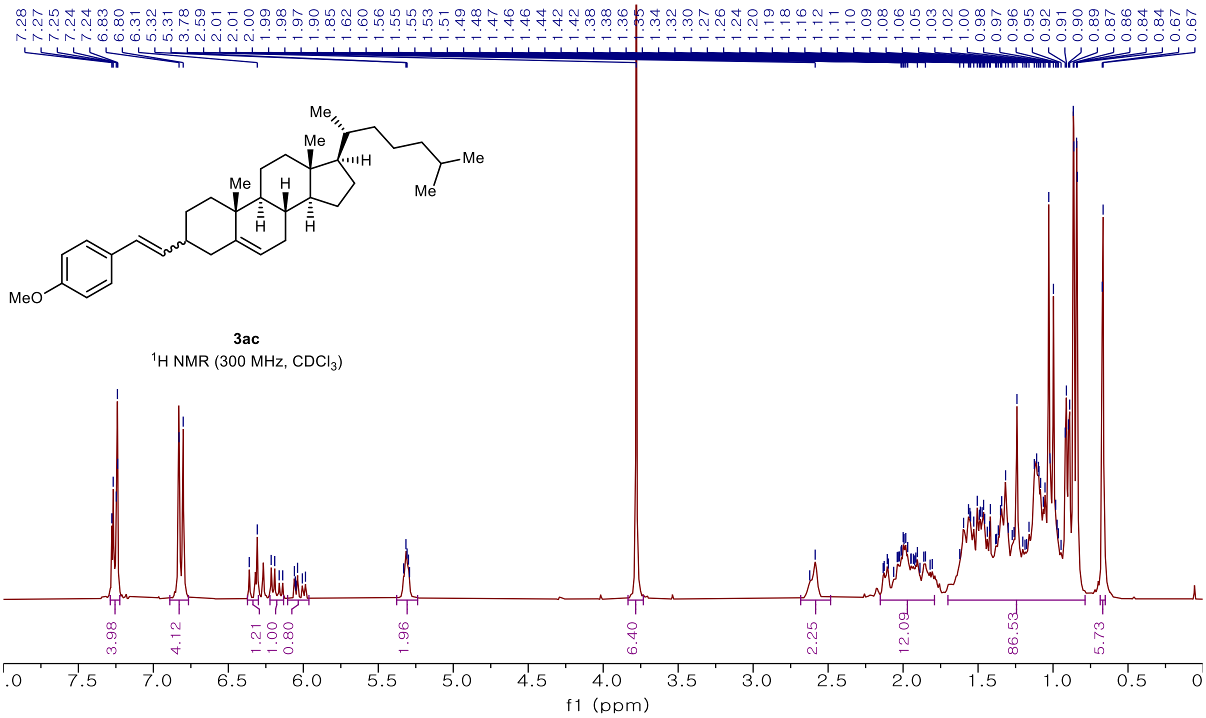

Supplementary Fig. 77. ^{1}H NMR spectrum of compound 3ac.

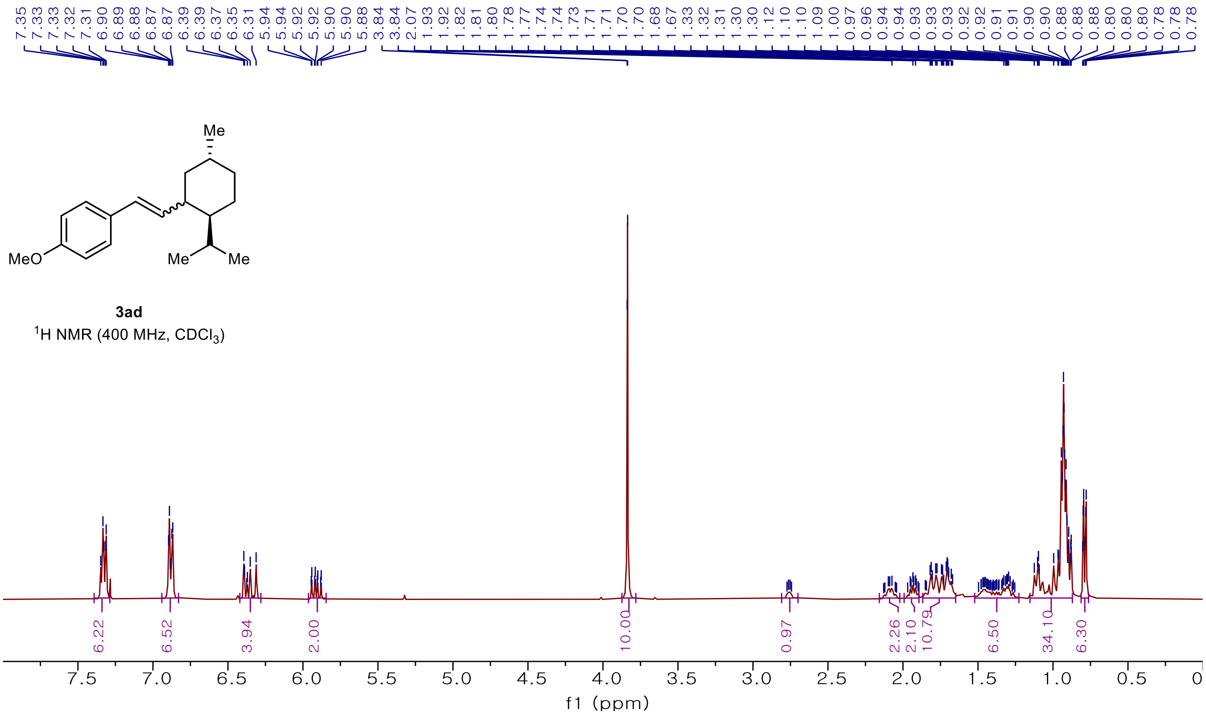

Supplementary Fig. 78. ^{1}H NMR spectrum of compound 3ad.

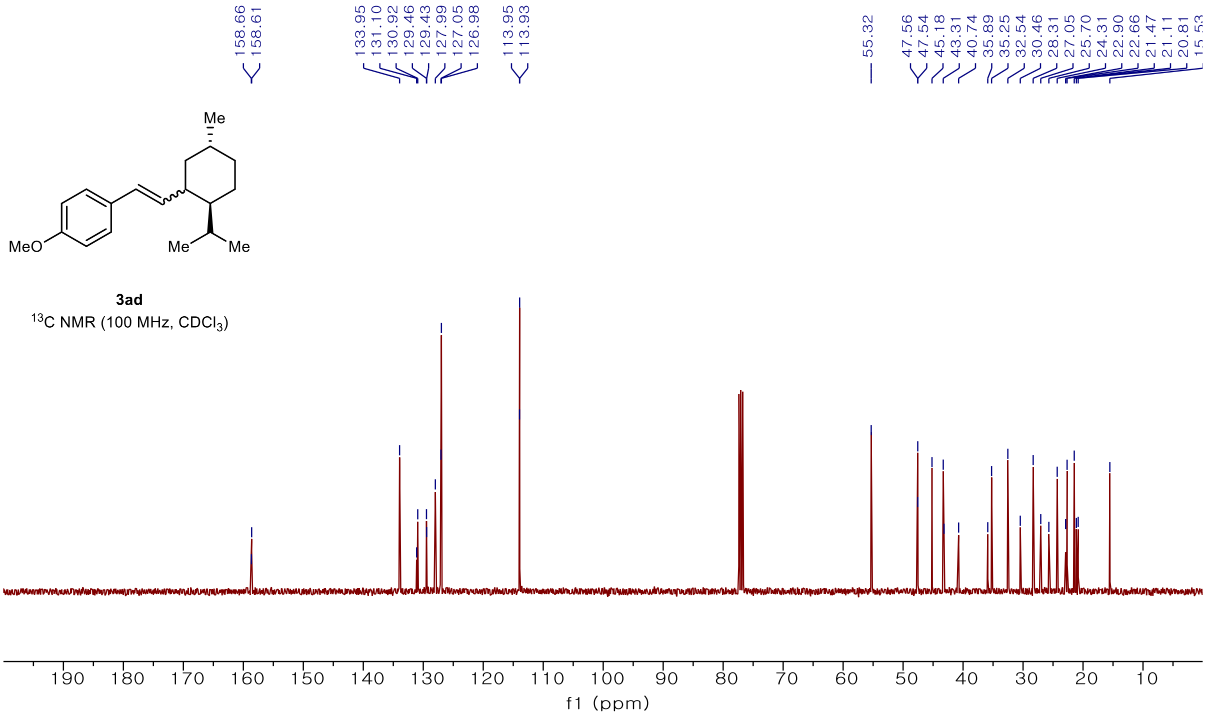

Supplementary Fig. 79. ^{13}C NMR spectrum of compound 3ad.

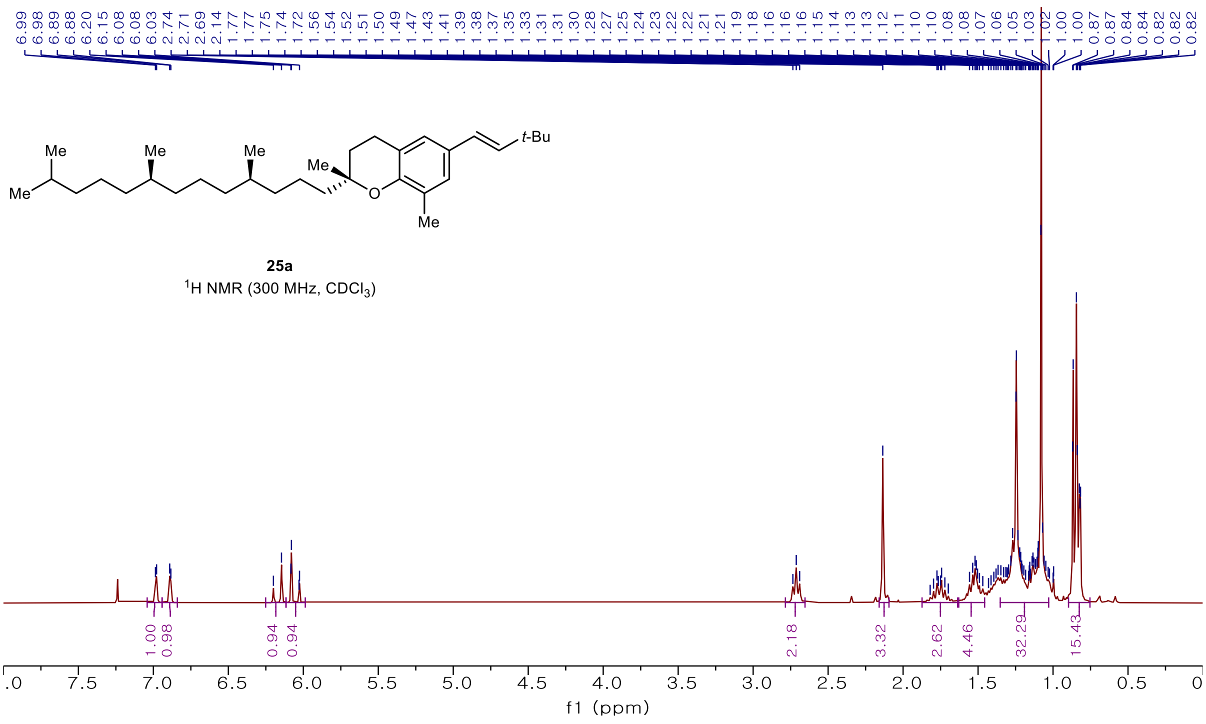

Supplementary Fig. 80. ^{1}H NMR spectrum of compound 25a.

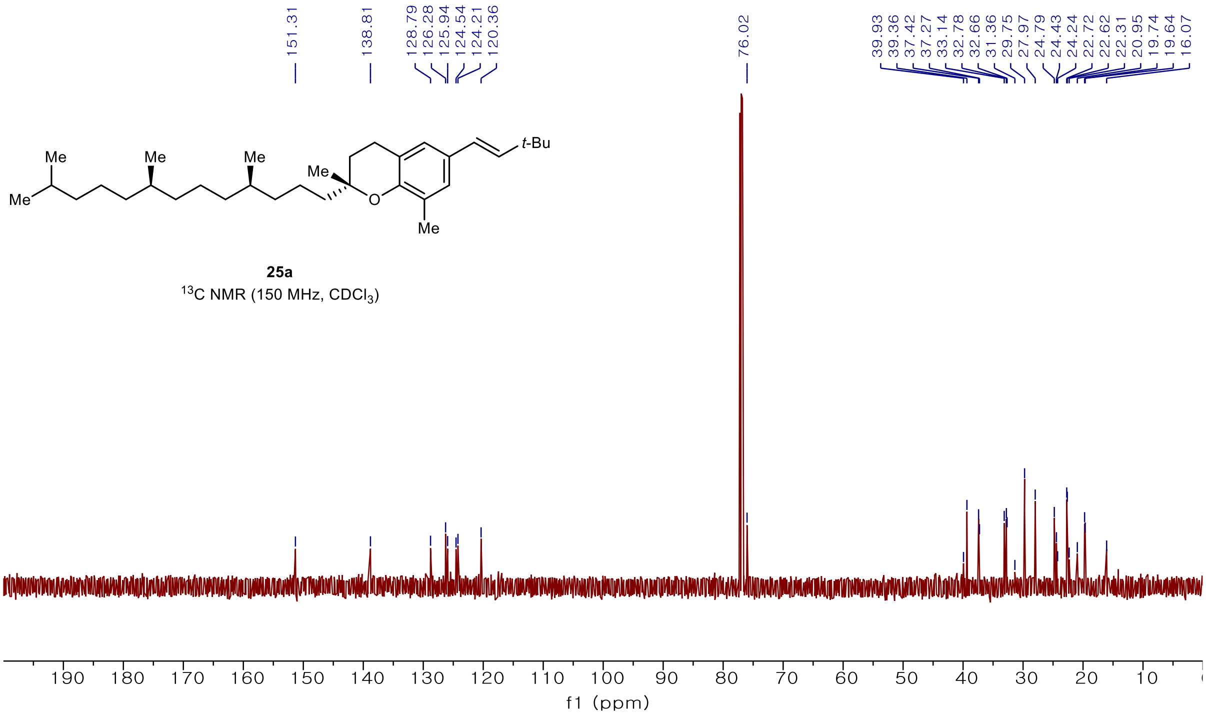

Supplementary Fig. 81. ^{13}C NMR spectrum of compound 25a.

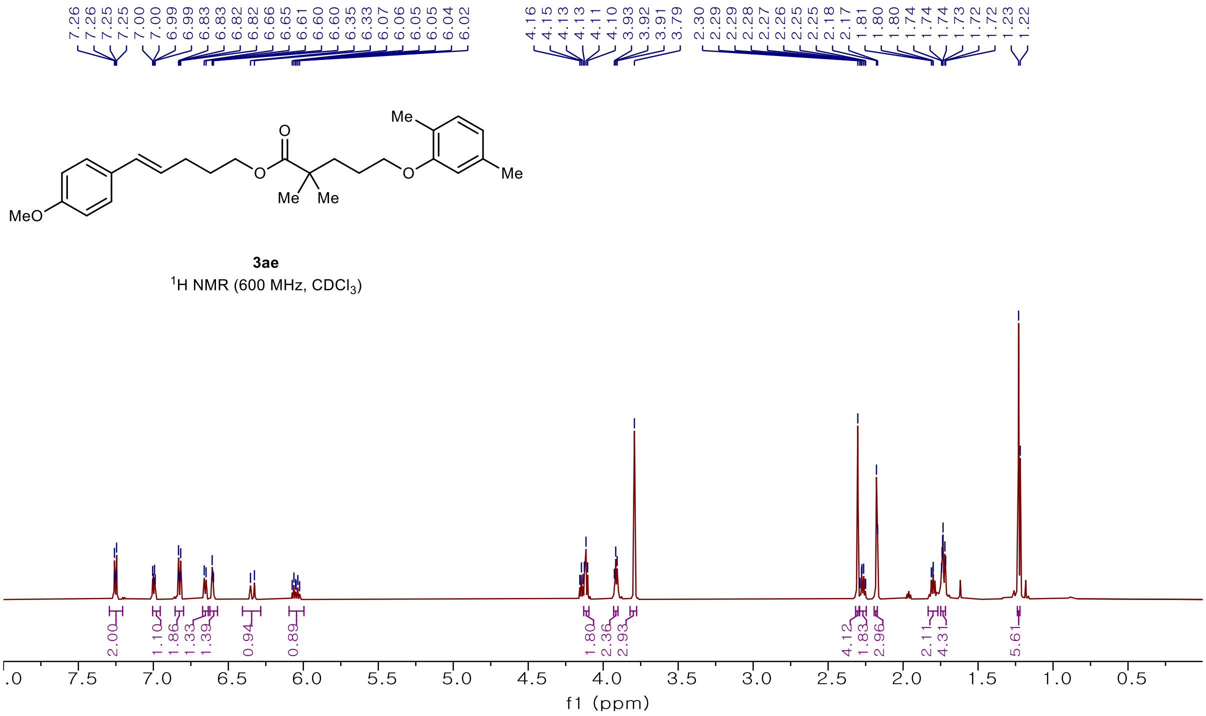

Supplementary Fig. 82. ^{1}H NMR spectrum of compound 3ae.

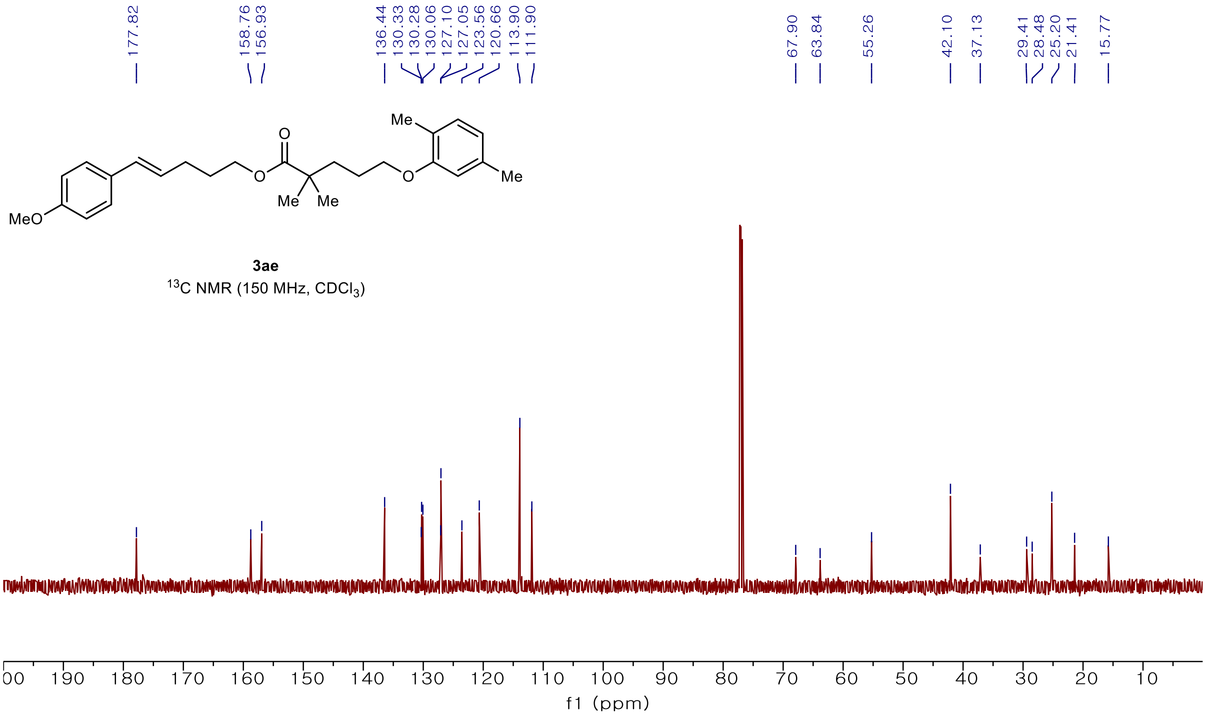

Supplementary Fig. 83. ^{13}C NMR spectrum of compound 3ae.

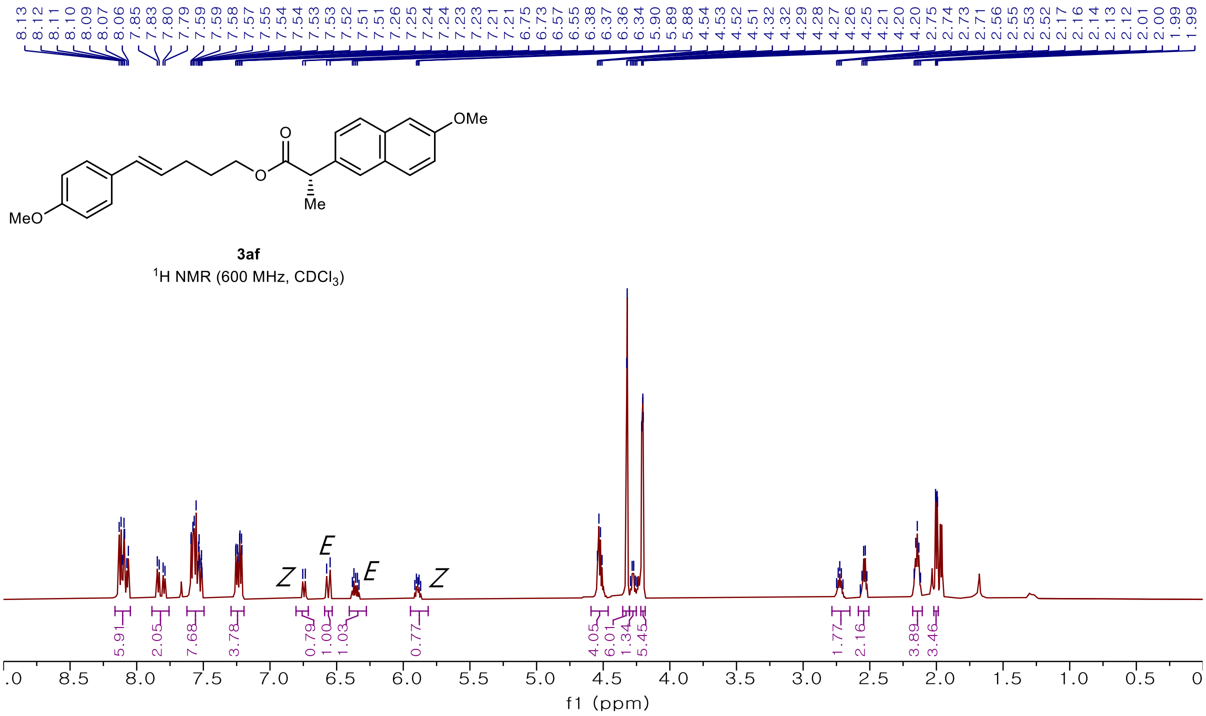

Supplementary Fig. 84. ^{1}H NMR spectrum of compound 3af

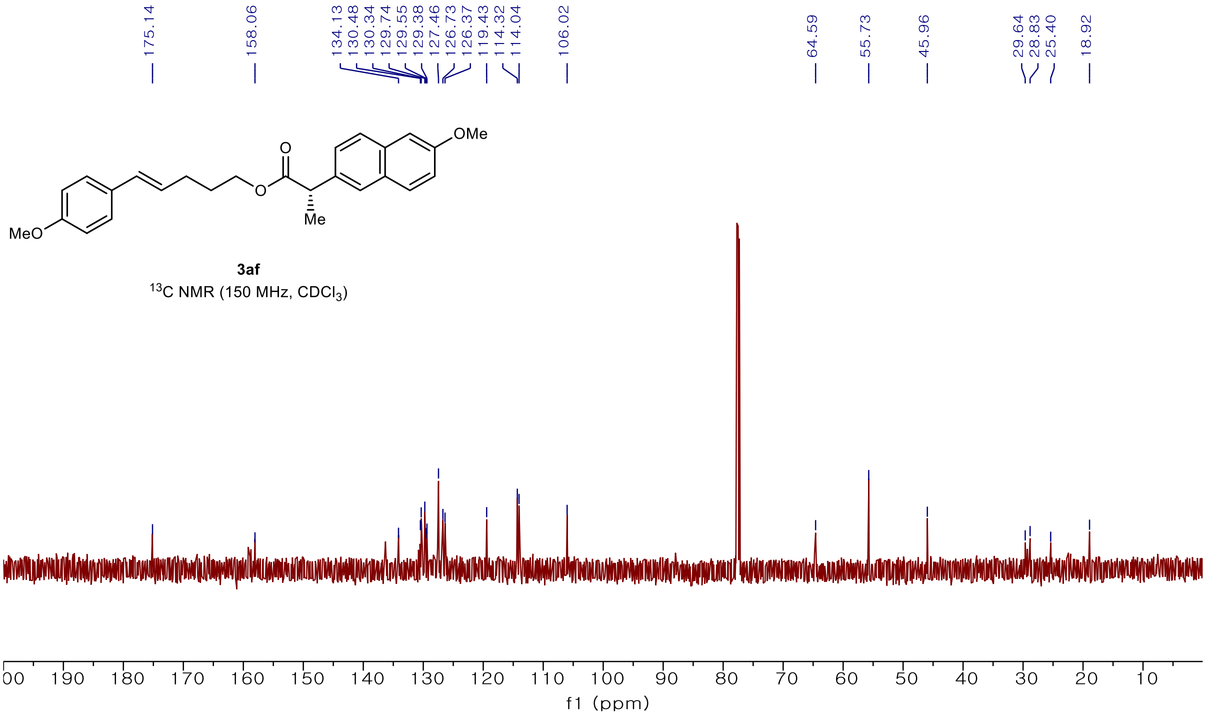

Supplementary Fig. 85. ^{13}C NMR spectrum of compound 3af.

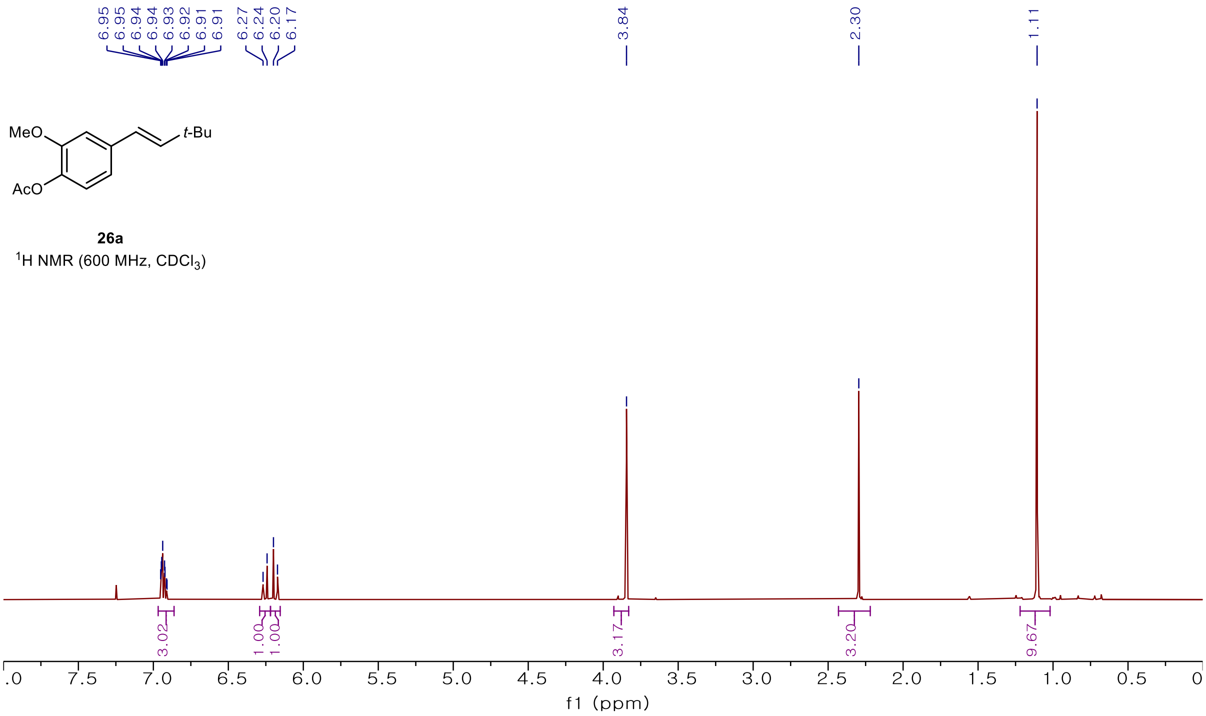

Supplementary Fig. 86. ^{1}H NMR spectrum of compound 26a.

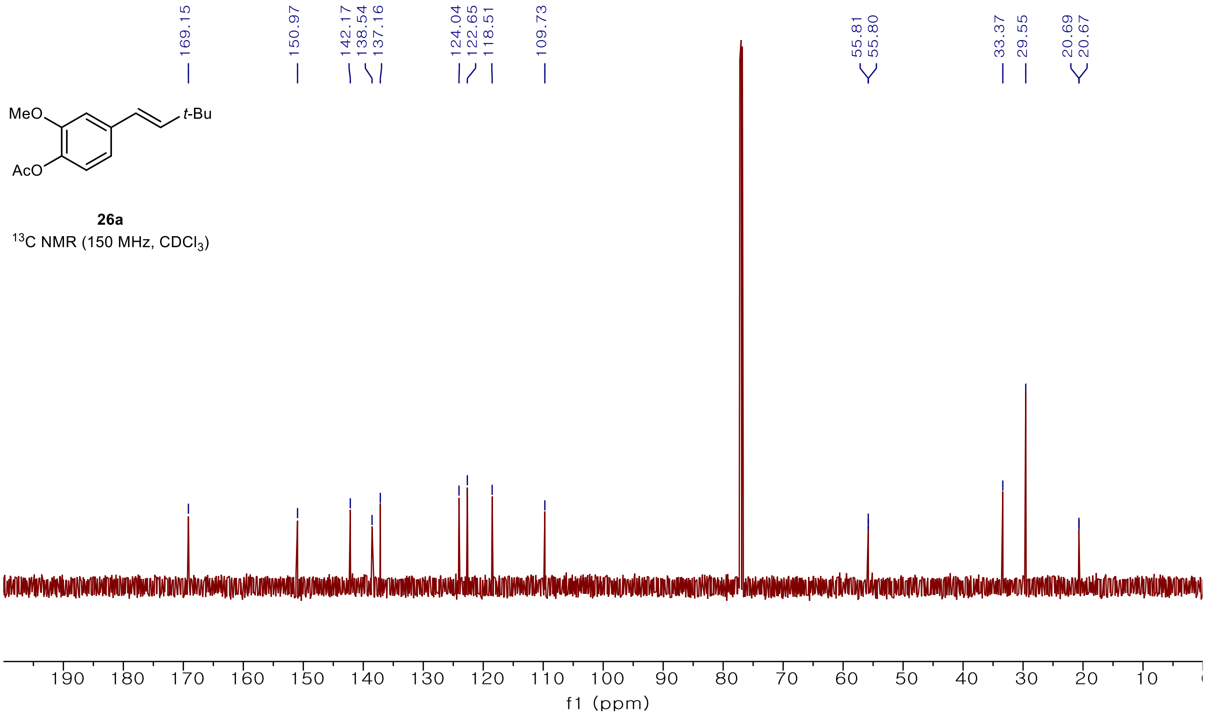

Supplementary Fig. 87. ^{13}C NMR spectrum of compound 26a.

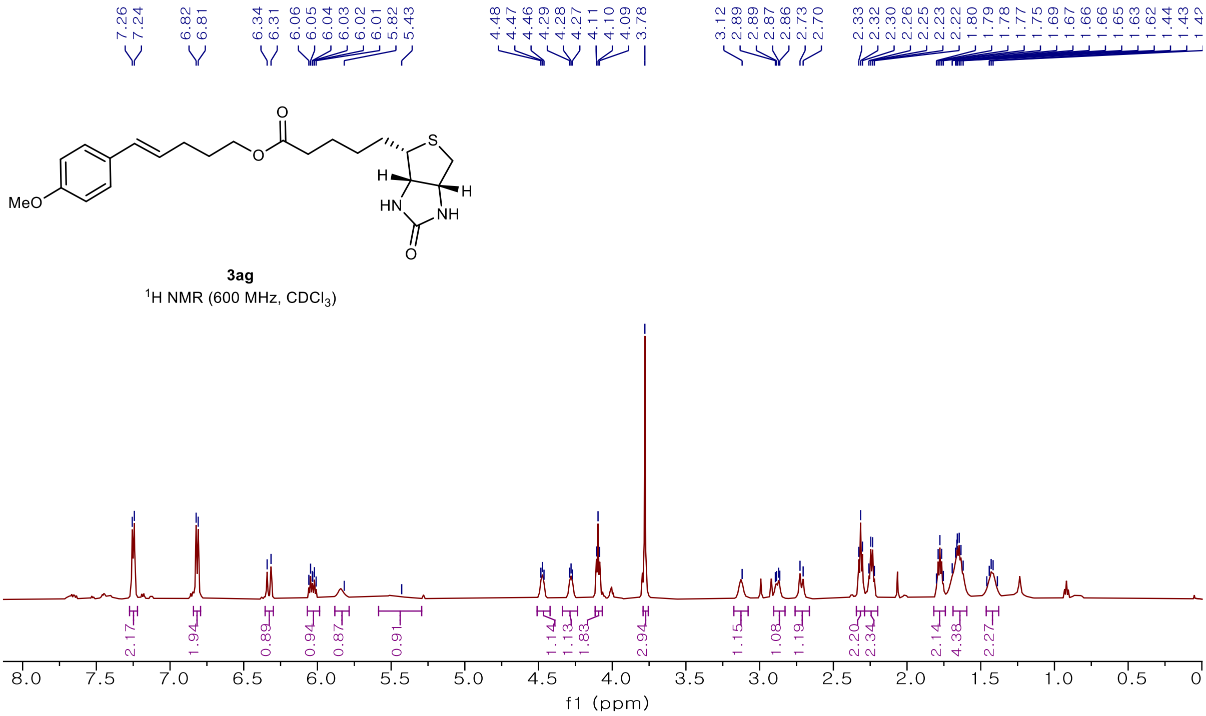

Supplementary Fig. 88. ^{1}H NMR spectrum of compound 3ag.

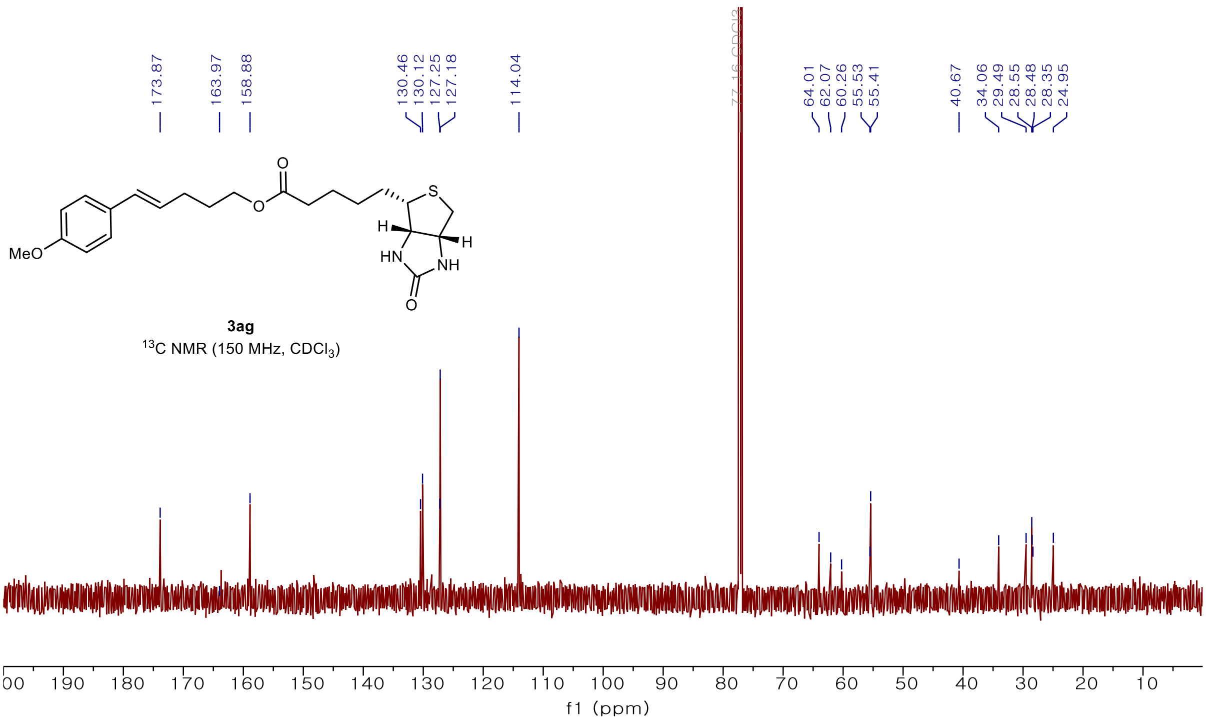

Supplementary Fig. 89. ^{13}C NMR spectrum of compound 3ag.

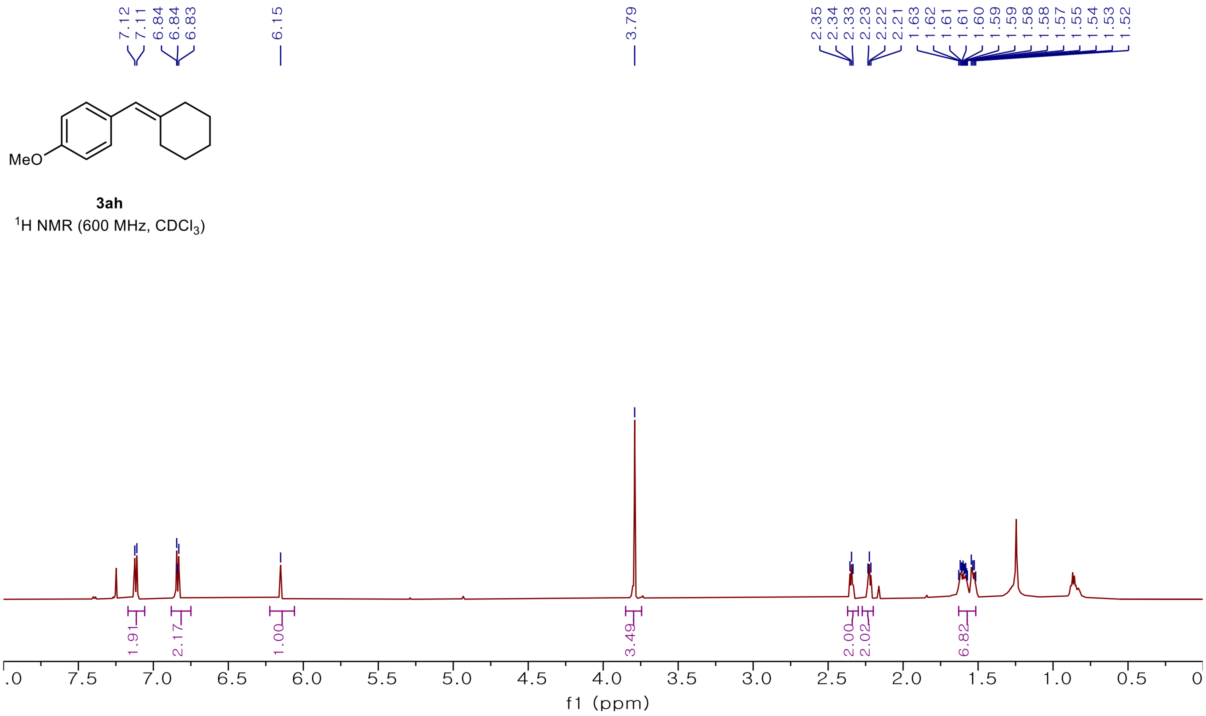

Supplementary Fig. 90. ^{1}H NMR spectrum of compound 3ah.

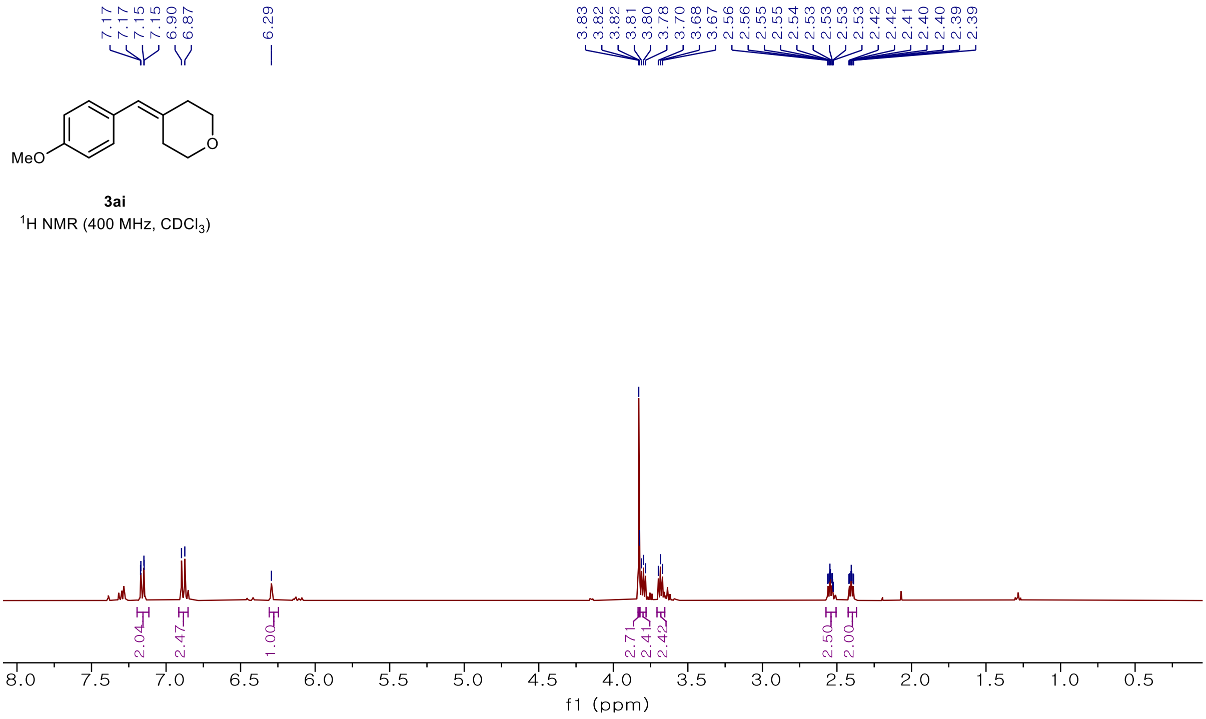

Supplementary Fig. 91. ^{1}H NMR spectrum of compound 3ai.

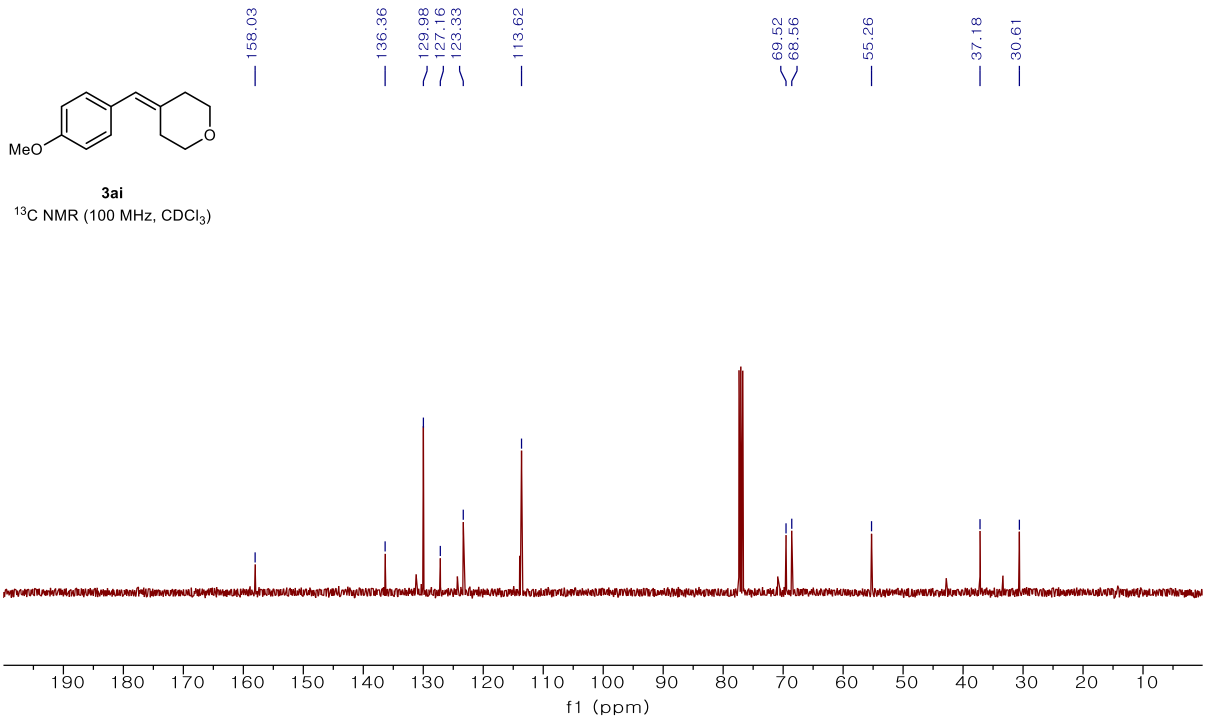

Supplementary Fig. 92. ^{1}H NMR spectrum of compound 3ai.

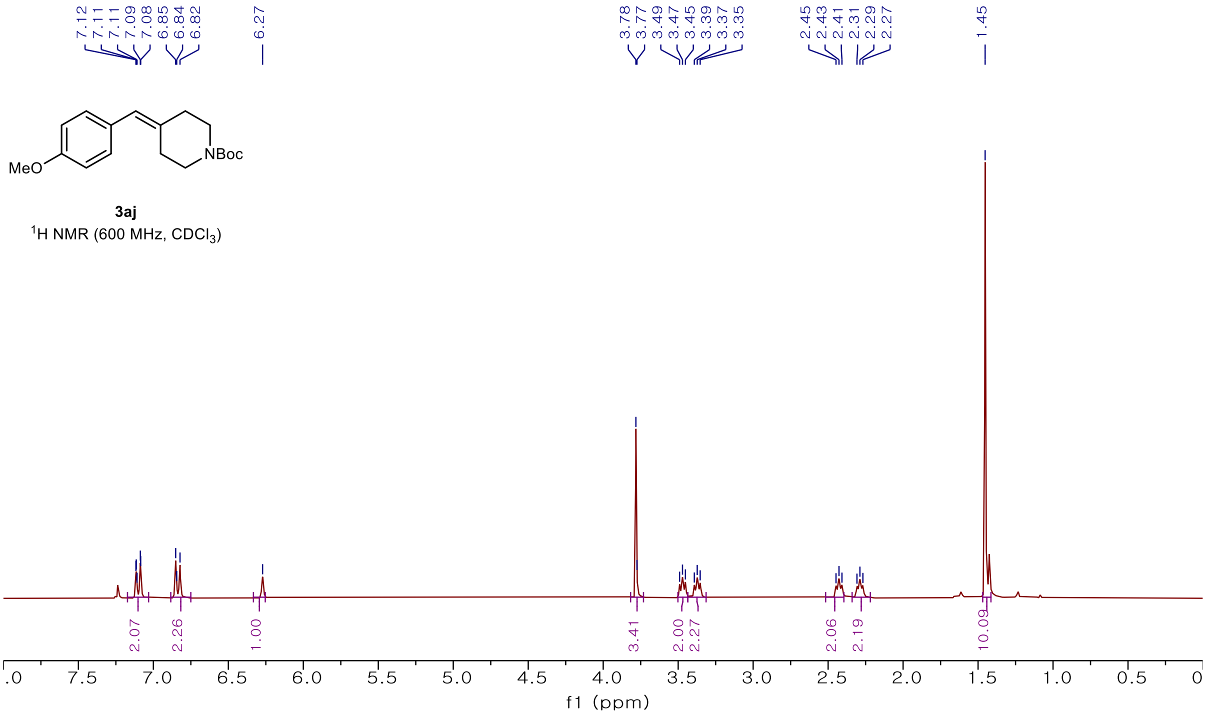

Supplementary Fig. 93. ^{1}H NMR spectrum of compound 3aj.

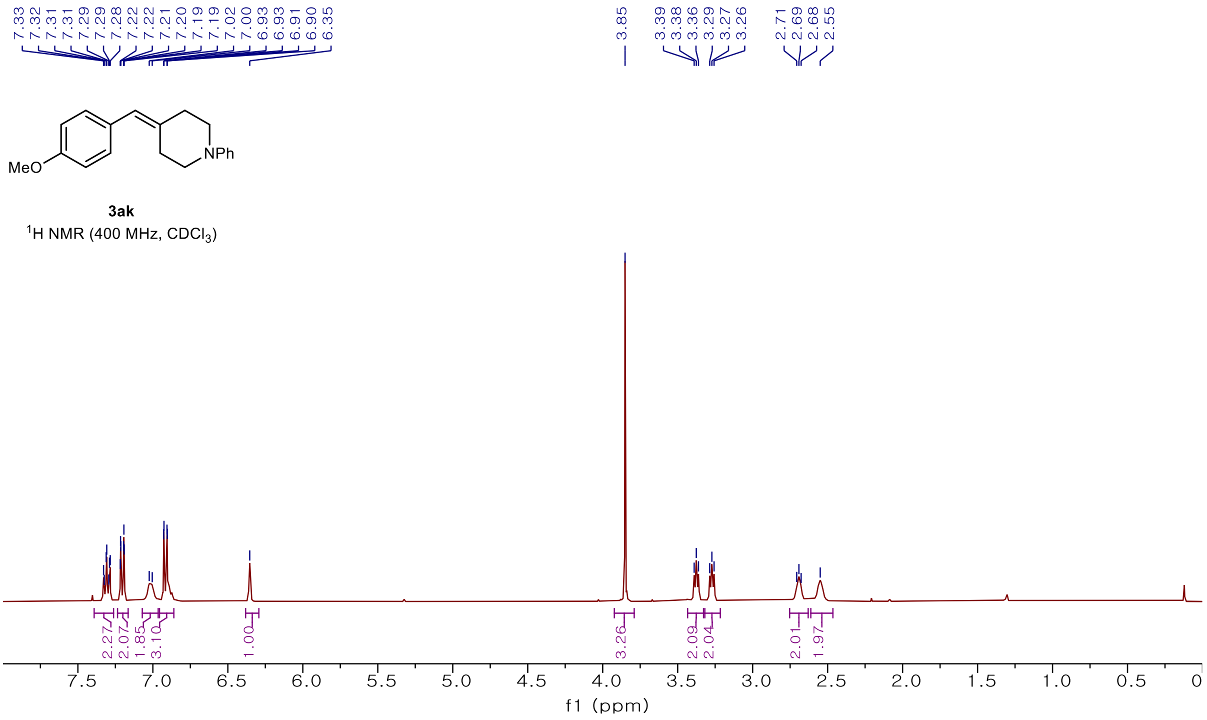

Supplementary Fig. 94. ^{1}H NMR spectrum of compound 3ak.

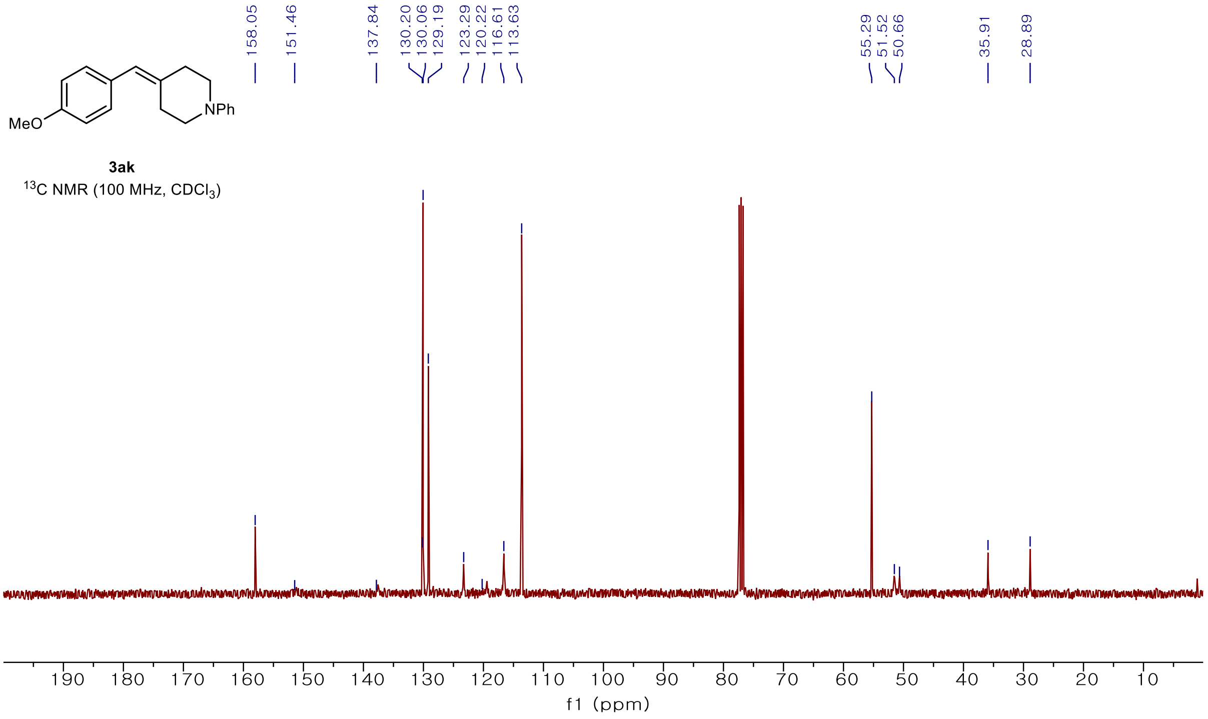

Supplementary Fig. 95. ^{13}C NMR spectrum of compound 3ak.

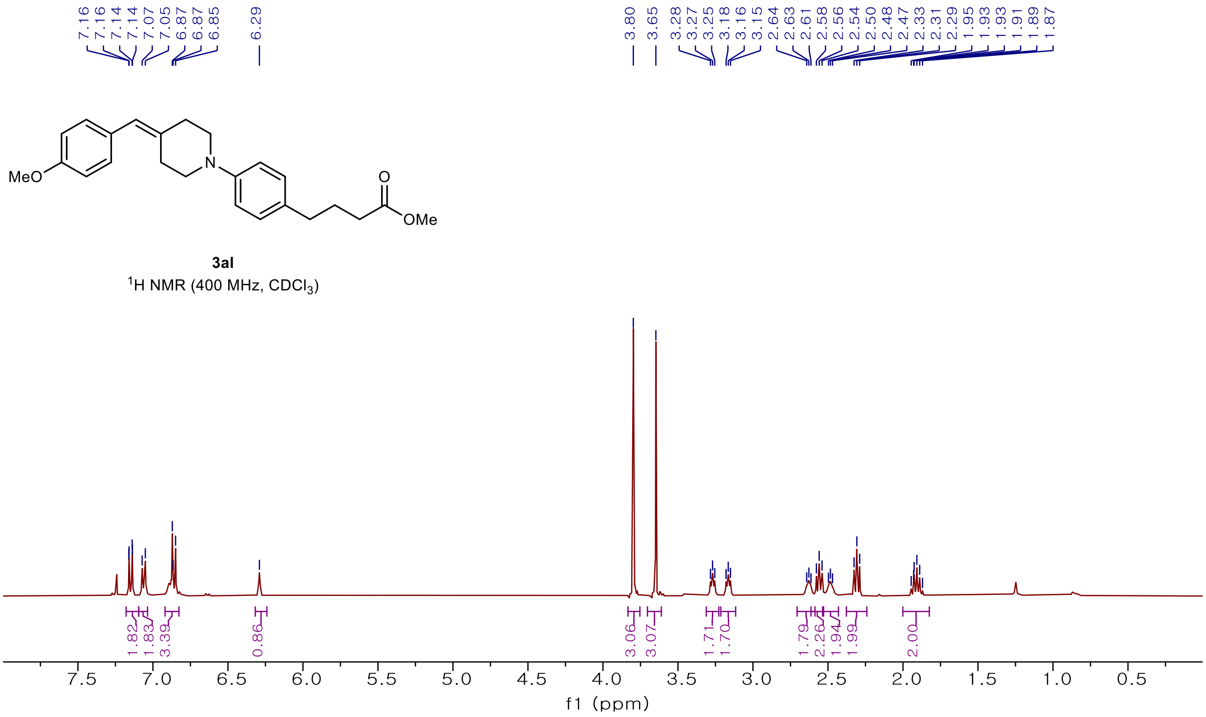

Supplementary Fig. 96. ^{1}H NMR spectrum of compound 3al.

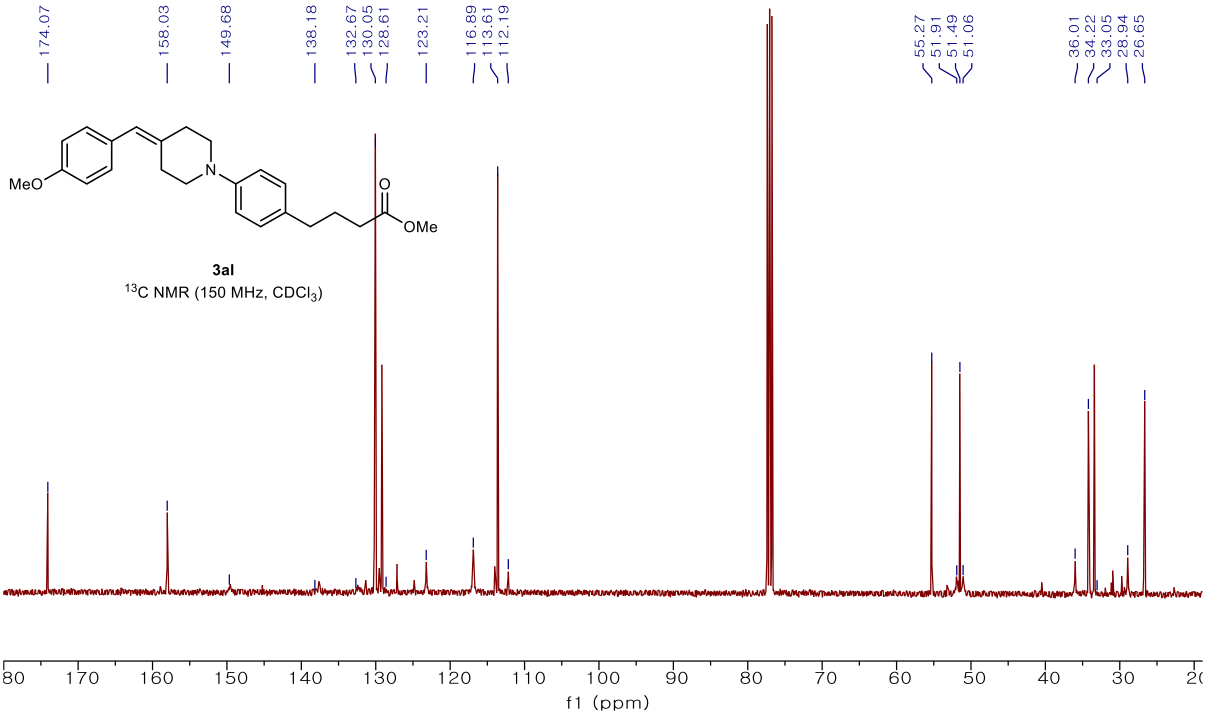

Supplementary Fig. 97. ^{13}C NMR spectrum of compound 3al.

13. Supplementary references

1. Kolocouris, A., Koch, A., Kleinpeter, E. & Stylianakis, I. 2-Substituted and 2,2-disubstituted adamantane derivatives as models for studying substituent chemical shifts and C–H_{ax}···Y_{ax} cyclohexane contacts—results from experimental and theoretical NMR spectroscopic chemical shifts and DFT structures. *Tetrahedron* **71**, 2463-2481 (2015).
2. Wu, X. *et al.* Ti-catalyzed radical alkylation of secondary and tertiary alkyl chlorides using Michael acceptors. *J. Am. Chem. Soc.* **140**, 14836-14843 (2018).
3. Villalpando, A., Ayala, C. E., Watson, C. B. & Kartika, R. Triphosgene-amine base promoted chlorination of unactivated aliphatic alcohols. *J. Org. Chem.* **78**, 3989-3996 (2013).
4. Liu, Y. *et al.* Structural optimization elaborates novel potent Akt inhibitors with promising anticancer activity. *Eur. J. Med. Chem.* **138**, 543-551 (2017).
5. van Kuijk, S. J. A. *et al.* New approach of delivering cytotoxic drugs towards CAIX expressing cells: A concept of dual-target drugs. *Eur. J. Med. Chem.* **127**, 691-702 (2017).
6. Mato, M., Herlé, B. & Echavarren, A. M. Cyclopropanation by gold- or zinc-catalyzed retro-Buchner reaction at room temperature. *Org. Lett.* **20**, 4341-4345 (2018).
7. Qing, Z. *et al.* Visible light photoredox catalyzed semisynthesis of the analogues of maclekarpine E: a series of 6-vinyl substituted dihydrobenzophenanthridine alkaloids. *Org. Chem. Front.* **5**, 353-357 (2018).
8. Wang, G.-Z., Shang, R., Cheng, W.-M. & Fu, Y. Irradiation-induced Heck reaction of unactivated alkyl halides at room temperature. *J. Am. Chem. Soc.* **139**, 18307-18312 (2017).
9. Liu, J. T. *et al.* Novel synthesis of alkenes via triethylborane-induced free-radical reactions of alkyl iodides and beta-nitrostyrenes. *J. Org. Chem.* **66**, 6021-6028 (2001).
10. Yu, W., Chen, L., Tao, J., Wang, T. & Fu, J. Dual nickel- and photoredox-catalyzed reductive cross-coupling of aryl vinyl halides and unactivated tertiary alkyl bromides. *Chem. Commun.* **55**, 5918-5921 (2019).
11. Shi, H., Dai, W., Wang, B. & Cao, S. Copper- and nickel-catalyzed cross-coupling reaction of monofluoroalkenes with tertiary, secondary, and primary alkyl and aryl grignard reagents. *Organometallics* **37**, 459-463 (2018).
12. Wang, G.-Z., Shang, R. & Fu, Y. Irradiation-induced palladium-catalyzed decarboxylative Heck reaction of aliphatic *N*-(acyloxy)phthalimides at room temperature. *Org. Lett.* **20**, 888-891 (2018).
13. Huang, H., Jia, K. & Chen, Y. Hypervalent iodine reagents enable chemoselective deboronative/decarboxylative alkenylation by photoredox catalysis. *Angew. Chem. Int. Ed.* **54**, 1881-1884 (2015).
14. Koy, M. *et al.* Palladium-catalyzed decarboxylative Heck-type coupling of activated aliphatic carboxylic acids enabled by visible light. *Chem. Eur. J.* **24**, 4552-4555 (2018).
15. Teng, S., Tessensohn, M. E., Webster, R. D. & Zhou, J. S. Palladium-catalyzed intermolecular Heck-type reaction of epoxides. *ACS Catal.* **8**, 7439-7444 (2018).
16. Armstrong, M. K., Goodstein, M. B. & Lalic, G. Diastereodivergent reductive cross coupling of alkynes through tandem catalysis: *Z*- and *E*-selective hydroarylation of terminal alkynes. *J. Am. Chem. Soc.* **140**, 10233-10241 (2018).

17. Xu, K. *et al.* Photoredox catalysis enabled alkylation of alkenyl carboxylic acids with *N*-(acyloxy)phthalimide via dual decarboxylation. *Chem. Commun.* **53**, 10719-10722 (2017).
18. Kurandina, D., Rivas, M., Radzhabov, M. & Gevorgyan, V. Heck reaction of electronically diverse tertiary alkyl halides. *Org. Lett.* **20**, 357-360 (2018).
19. Hansen, A. L., Ebran, J.-P., Gøgsig, T. M. & Skrydstrup, T. Investigations on the Suzuki-Miyaura and Negishi couplings with alkenyl phosphates: application to the synthesis of 1,1-disubstituted alkenes. *J. Org. Chem.* **72**, 6464-6472 (2007).
20. Oh, C. H., Lim, Y. M. & You, C. H. Platinum-catalyzed cross-couplings of organoboronic acids with aryl iodides. *Tetrahedron Lett.* **43**, 4645-4647 (2002).
21. Andrews, P., Latham, C. M., Magre, M., Willcox, D. & Woodward, S. $ZrCl_2(\eta\text{-}C_5Me_5)_2\text{-}AlHCl_2\cdot(THF)_2$: efficient hydroalumination of terminal alkynes and cross-coupling of the derived alanes. *Chem. Commun.* **49**, 1488-1490 (2013).
22. Zong, Z., Wang, W., Bai, X., Xi, H. & Li, Z. Manganese-catalyzed alkyl-Heck-type reaction via oxidative decarbonylation of aldehydes. *Asian J. Org. Chem.* **4**, 622-625 (2015).
23. Gooßen, L. J. *et al.* A practical and effective ruthenium trichloride-based protocol for the regio- and stereoselective catalytic hydroamidation of terminal alkynes. *Adv. Synth. Catal.* **350**, 2701-2707 (2008).
24. Lau, S. Y. W., Hughes, G., O'Shea, P. D. & Davies, I. W. Magnesiation of electron-rich aryl bromides and their use in nickel-catalyzed cross-coupling reactions. *Org. Lett.* **9**, 2239-2242 (2007).
25. Imamura, S. *et al.* CCR5 antagonists as anti-HIV-1 agents. Part 3: Synthesis and biological evaluation of piperidine-4-carboxamide derivatives. *Bioorg. Med. Chem.* **13**, 397-416 (2005).
26. Singh, K., Staig, S. J. & Weaver, J. D. Facile synthesis of Z-alkenes via uphill catalysis. *J. Am. Chem. Soc.* **136**, 5275-5278 (2014).
27. Singh, A., Fennell, C. J. & Weaver, J. D. Photocatalyst size controls electron and energy transfer: selectable E/Z isomer synthesis via C–F alkenylation. *Chem. Sci.* **7**, 6796-6802 (2016).
28. Fabry, D. C., Ronge, M. A. & Rueping, M. Immobilization and continuous recycling of photoredox catalysts in ionic liquids for applications in batch reactions and flow systems: catalytic alkene isomerization by using visible light. *Chem. Eur. J.* **21**, 5350-5354 (2015).
29. Cai, W., Fan, H., Ding, D., Zhang, Y. & Wang, W. Synthesis of Z-alkenes via visible light promoted photocatalytic E → Z isomerization under metal-free conditions. *Chem. Commun.* **53**, 12918-12921 (2017).
30. Gao, F. & Hoveyda, A. H. Alpha-selective Ni-catalyzed hydroalumination of aryl- and alkyl-substituted terminal alkynes: practical syntheses of internal vinyl aluminums, halides, or boronates. *J. Am. Chem. Soc.* **132**, 10961-10963 (2010).
31. Gaussian 09, Revision D.01, M. J. Frisch, G. W. Trucks, H. B. Schlegel, G. E. Scuseria, M. A. Robb, J. R. Cheeseman, G. Scalmani, V. Barone, B. Mennucci, G. A. Petersson, H. Nakatsuji, M. Caricato, X. Li, H. P. Hratchian, A. F. Izmaylov, J. Bloino, G. Zheng, J. L. Sonnenberg, M. Hada, M. Ehara, K. Toyota, R. Fukuda, J. Hasegawa, M. Ishida, T. Nakajima, Y. Honda, O. Kitao, H. Nakai, T. Vreven, J. A. Montgomery, Jr., J. E. Peralta, F. Ogliaro, M. Bearpark, J. J. Heyd, E. Brothers, K. N. Kudin, V. N. Staroverov, T. Keith, R. Kobayashi, J. Normand, K. Raghavachari, A. Rendell, J. C. Burant, S. S. Iyengar, J. Tomasi, M. Cossi, N. Rega, J. M. Millam, M. Klene, J. E. Knox, J. B. Cross, V. Bakken, C. Adamo, J. Jaramillo, R. Gomperts, R. E. Stratmann, O. Yazyev, A. J. Austin, R. Cammi, C. Pomelli, J. W. Ochterski, R. L. Martin, K. Morokuma,

V. G. Zakrzewski, G. A. Voth, P. Salvador, J. J. Dannenberg, S. Dapprich, A. D. Daniels, O. Farkas, J. B. Foresman, J. V. Ortiz, J. Cioslowski, and D. J. Fox, Gaussian, Inc., Wallingford CT, 2013.

32. Lee, C., Yang, W. & Parr, R. G. Development of the Colle-Salvetti correlation-energy formula into a functional of the electron density. *Phys. Rev. B Condens. Matter.* **37**, 785-789 (1988).
33. Grimme, S., Antony, J., Ehrlich, S. & Krieg, H. A consistent and accurate ab initio parametrization of density functional dispersion correction (DFT-D) for the 94 elements H-Pu. *J. Chem. Phys.* **132**, 154104 (2010).
34. Hay, P. J. & Wadt, W. R. Ab initio effective core potentials for molecular calculations. Potentials for K to Au including the outermost core orbitals. *J. Chem. Phys.* **82**, 299-310 (1985).
35. Wadt, W. R. & Hay, P. J. Ab initio effective core potentials for molecular calculations. Potentials for main group elements Na to Bi. *J. Chem. Phys.* **82**, 284-298 (1985).
36. Yanai, T., Tew, D. P. & Handy, N. C. A new hybrid exchange–correlation functional using the Coulomb-attenuating method (CAM-B3LYP). *Chem. Phys. Lett.* **393**, 51-57 (2004).
37. Trasatti, S. The absolute electrode potential: An explanatory note. *Pure Appl. Chem.* **58**, 955-966.
