# Supplementary material for: Pd-catalyzed formal Mizoroki–Heck coupling of unactivated alkyl chlorides
Source: Nat Commun. 2021 Feb 12;12:991. doi: 10.1038/s41467-021-21270-9 (PMC7881129; doi:10.1038/s41467-021-21270-9)
Supplement: Supplementary file 2 — Description of Additional Supplementary Files [file 41467_2021_21270_MOESM2_ESM.pdf]

Description of Additional Supplementary Files

File Name: Supplementary Data 1
Description: Cartesian coordinates of DFT-optimized structures
