# Supplementary material for: Pd-catalyzed formal Mizoroki–Heck coupling of unactivated alkyl chlorides
Source: Nat Commun. 2021 Feb 12;12:991. doi: 10.1038/s41467-021-21270-9 (PMC7881129; doi:10.1038/s41467-021-21270-9)
Supplement: Supplementary file 3 — Supplementary Data 1 [file 41467_2021_21270_MOESM3_ESM.zip › 277654_2_data_set_5204812_qmn8my.docx]

**Pd-catalyzed Formal Mizoroki-Heck Coupling of Unactivated Alkyl Chlorides**

Geun Seok Lee, Daeun Kim, and Soon Hyeok Hong*

Department of Chemistry, Korea Advanced Institute of Science and Technology (KAIST), Daejeon 34141, Republic of Korea

E-mail: Soon Hyeok Hong ([soonhyeok.hong@kaist.ac.kr](mailto:soonhyeok.hong@kaist.ac.kr))

**Cartesian coordinates of DFT-optimized structures**

===============================

**I-OMe-rad**

===============================

C -3.179770000 0.196570000 0.000020000

C -2.571303000 1.467481000 0.000002000

C -2.375544000 -0.952599000 -0.000062000

H -3.212412000 2.343040000 0.000027000

H -2.819296000 -1.941283000 -0.000095000

C -1.197747000 1.582197000 -0.000064000

C -0.990303000 -0.831994000 -0.000119000

H -0.744463000 2.570130000 -0.000098000

H -0.390285000 -1.736134000 -0.000195000

C -0.344909000 0.436033000 -0.000083000

C 1.060527000 0.582821000 -0.000097000

C 2.025839000 -0.563316000 0.000115000

H 1.832509000 -1.208148000 -0.873815000

C 3.529477000 -0.187576000 0.000037000

C 3.880713000 0.624429000 1.260837000

C 3.880592000 0.624275000 -1.260878000

H 3.639082000 0.060489000 -2.169040000

H 3.333063000 1.570926000 -1.301804000

H 4.950793000 0.858107000 -1.284482000

H 3.333112000 1.571043000 1.301779000

H 4.950895000 0.858380000 1.284256000

H 3.639393000 0.060718000 2.169093000

C 4.350696000 -1.490522000 0.000054000

H 4.129906000 -2.096726000 0.886012000

H 4.129910000 -2.096749000 -0.885893000

H 1.832518000 -1.207831000 0.874281000

H 5.425241000 -1.276669000 0.000047000

H 1.454383000 1.595189000 0.000001000

O -4.545105000 0.197151000 0.000104000

C -5.216585000 -1.052966000 0.000060000

H -4.975225000 -1.643703000 -0.893685000

H -4.975173000 -1.643766000 0.893753000

H -6.283214000 -0.823210000 0.000097000

===============================

**I-OMe-cat**

===============================

C -2.905445000 0.170743000 0.069283000

C -2.404264000 1.492616000 0.245964000

C -2.042945000 -0.880327000 -0.360498000

H -3.095902000 2.259719000 0.574491000

H -2.425746000 -1.883715000 -0.494842000

C -1.087262000 1.753152000 0.001683000

C -0.723990000 -0.608077000 -0.606831000

H -0.700462000 2.758787000 0.137160000

H -0.070809000 -1.408738000 -0.932711000

C -0.187276000 0.714448000 -0.433451000

C 1.144488000 1.019302000 -0.649150000

C 2.240856000 0.120023000 -1.042189000

H 2.880354000 0.655949000 -1.753823000

C 3.168719000 -0.299904000 0.182791000

C 2.370064000 -1.187516000 1.149154000

C 3.697752000 0.946710000 0.910944000

H 4.203615000 1.630725000 0.221149000

H 2.899506000 1.496904000 1.420843000

H 4.424209000 0.650368000 1.673031000

H 1.516895000 -0.657646000 1.585000000

H 3.012467000 -1.510893000 1.973559000

H 1.998699000 -2.089947000 0.650615000

C 4.340908000 -1.096631000 -0.414059000

H 3.990430000 -1.976847000 -0.962982000

H 4.937659000 -0.481945000 -1.095350000

H 1.882013000 -0.792159000 -1.524643000

H 4.999544000 -1.443365000 0.388256000

H 1.431630000 2.054005000 -0.463701000

O -4.186177000 0.019728000 0.328219000

C -4.837281000 -1.265705000 0.192629000

H -4.781186000 -1.611785000 -0.842864000

H -4.385853000 -1.993363000 0.872224000

H -5.873585000 -1.086877000 0.471217000

===============================

**I-OAc-rad**

===============================

C -2.231226000 0.549102000 -0.168083000

C -1.694888000 1.738910000 0.337351000

C -1.402032000 -0.416463000 -0.751837000

H -2.365612000 2.470606000 0.775528000

H -1.826599000 -1.335309000 -1.133067000

C -0.332159000 1.962199000 0.268709000

C -0.036542000 -0.190239000 -0.815840000

H 0.079458000 2.886924000 0.663727000

H 0.594267000 -0.951353000 -1.260367000

C 0.555933000 1.004404000 -0.308048000

C 1.943694000 1.269273000 -0.354125000

C 3.008596000 0.375287000 -0.904468000

H 3.748863000 0.996371000 -1.428414000

C 3.784339000 -0.480151000 0.152866000

C 2.830666000 -1.479961000 0.828143000

C 4.410719000 0.436795000 1.218115000

H 5.066314000 1.186153000 0.759105000

H 3.641598000 0.965045000 1.790398000

H 5.011484000 -0.146186000 1.924750000

H 2.003637000 -0.967128000 1.327647000

H 3.363752000 -2.075987000 1.577350000

H 2.400795000 -2.173398000 0.096371000

C 4.899224000 -1.248210000 -0.578426000

H 4.486762000 -1.900039000 -1.357082000

H 5.607176000 -0.560964000 -1.055822000

H 2.595988000 -0.308634000 -1.655837000

H 5.462134000 -1.876797000 0.120457000

H 2.274808000 2.207321000 0.086248000

O -3.620674000 0.445389000 -0.122311000

C -4.244242000 -0.733457000 0.193342000

O -3.674588000 -1.764306000 0.453927000

C -5.738359000 -0.529954000 0.160591000

H -6.046883000 -0.206992000 -0.838008000

H -6.023380000 0.259760000 0.861491000

H -6.234820000 -1.463264000 0.422860000

===============================

**I-OAc-cat**

===============================

C -2.191761000 0.476233000 -0.035283000

C -1.604162000 1.755833000 0.149393000

C -1.405166000 -0.637084000 -0.441599000

H -2.244988000 2.572771000 0.460060000

H -1.865417000 -1.602842000 -0.579655000

C -0.263614000 1.926855000 -0.064530000

C -0.062036000 -0.452911000 -0.656632000

H 0.187966000 2.904000000 0.078291000

H 0.536991000 -1.300531000 -0.967423000

C 0.563827000 0.825547000 -0.474897000

C 1.922256000 1.034591000 -0.662484000

C 2.956829000 0.057464000 -1.019904000

H 3.651934000 0.536647000 -1.719608000

C 3.827259000 -0.405574000 0.238682000

C 2.943550000 -1.217029000 1.197181000

C 4.426489000 0.813986000 0.957514000

H 4.991076000 1.450877000 0.268104000

H 3.658602000 1.425041000 1.444097000

H 5.116849000 0.480766000 1.737697000

H 2.126430000 -0.617462000 1.610993000

H 3.544170000 -1.579120000 2.036851000

H 2.511911000 -2.093375000 0.701186000

C 4.951274000 -1.292653000 -0.321366000

H 4.550935000 -2.153637000 -0.866519000

H 5.606269000 -0.732479000 -0.996008000

H 2.546576000 -0.836839000 -1.493895000

H 5.563633000 -1.673756000 0.501798000

H 2.277750000 2.048065000 -0.477340000

O -3.509632000 0.460678000 0.209201000

C -4.378955000 -0.673263000 0.106962000

O -3.994749000 -1.755613000 -0.218677000

C -5.759806000 -0.232591000 0.469708000

H -6.081196000 0.569719000 -0.201040000

H -5.765101000 0.168447000 1.487613000

H -6.435247000 -1.083229000 0.394137000

===============================

**I-F-rad**

===============================

C -3.305364000 -0.272523000 0.113715000

C -2.990380000 1.080601000 0.244776000

C -2.348148000 -1.215646000 -0.257779000

H -3.765243000 1.781124000 0.536793000

H -2.637250000 -2.257492000 -0.344904000

C -1.691429000 1.491212000 -0.003018000

C -1.048572000 -0.799663000 -0.504177000

H -1.436789000 2.542794000 0.095704000

H -0.307406000 -1.538430000 -0.785930000

C -0.668527000 0.571200000 -0.387038000

C 0.643948000 1.040880000 -0.630699000

C 1.830561000 0.219492000 -1.022146000

H 2.417588000 0.783174000 -1.761002000

C 2.800434000 -0.164701000 0.145331000

C 2.076251000 -1.062543000 1.162925000

C 3.310662000 1.105358000 0.848520000

H 3.808705000 1.776963000 0.139447000

H 2.490609000 1.657262000 1.318565000

H 4.033393000 0.849260000 1.630967000

H 1.200151000 -0.561151000 1.584452000

H 2.746171000 -1.328518000 1.988120000

H 1.736890000 -1.994541000 0.696718000

C 3.992833000 -0.930039000 -0.454674000

H 3.660586000 -1.836801000 -0.973099000

H 4.539792000 -0.311486000 -1.175399000

H 1.519523000 -0.705729000 -1.522181000

H 4.696491000 -1.231788000 0.329144000

H 0.813918000 2.105698000 -0.487731000

F -4.570083000 -0.679122000 0.355101000

===============================

**I-F-cat**

===============================

C -3.283443000 -0.234786000 0.136965000

C -2.934916000 1.122625000 0.232542000

C -2.375481000 -1.244271000 -0.235662000

H -3.687545000 1.845283000 0.525396000

H -2.720891000 -2.270227000 -0.288493000

C -1.636951000 1.475830000 -0.053027000

C -1.081946000 -0.884254000 -0.525362000

H -1.332656000 2.516068000 0.013068000

H -0.368584000 -1.645244000 -0.817216000

C -0.669081000 0.487883000 -0.439288000

C 0.634564000 0.899169000 -0.705075000

C 1.791775000 0.081225000 -1.060929000

H 2.371744000 0.616233000 -1.822286000

C 2.788328000 -0.139306000 0.180880000

C 2.084041000 -0.980450000 1.254607000

C 3.239119000 1.212902000 0.754856000

H 3.669955000 1.856222000 -0.019619000

H 2.419602000 1.753599000 1.240333000

H 4.010061000 1.050563000 1.513408000

H 1.218563000 -0.465092000 1.683518000

H 2.780205000 -1.188421000 2.072381000

H 1.750730000 -1.945040000 0.856092000

C 3.997547000 -0.903676000 -0.382042000

H 3.701761000 -1.861132000 -0.822419000

H 4.523240000 -0.320152000 -1.144058000

H 1.517063000 -0.900844000 -1.450168000

H 4.704487000 -1.113396000 0.426792000

H 0.829534000 1.965229000 -0.589702000

F -4.523179000 -0.583443000 0.408021000

===============================

**I-Cl-rad**

===============================

C -2.910859000 -0.079501000 0.010423000

C -2.527119000 1.254096000 0.201933000

C -1.976302000 -1.043412000 -0.385597000

H -3.263378000 1.988404000 0.509702000

H -2.291945000 -2.071356000 -0.526842000

C -1.207908000 1.616178000 -0.004708000

C -0.656397000 -0.676109000 -0.590797000

H -0.911405000 2.650865000 0.144325000

H 0.055500000 -1.436367000 -0.890768000

C -0.217946000 0.669577000 -0.408507000

C 1.116965000 1.088958000 -0.604936000

C 2.276360000 0.233849000 -1.004442000

H 2.902521000 0.801582000 -1.706878000

C 3.201830000 -0.236408000 0.168282000

C 2.418178000 -1.149217000 1.126473000

C 3.740155000 0.981574000 0.938978000

H 4.272315000 1.669283000 0.271334000

H 2.930095000 1.537138000 1.421898000

H 4.440389000 0.665311000 1.719826000

H 1.550634000 -0.633013000 1.547907000

H 3.055485000 -1.476424000 1.955434000

H 2.057034000 -2.046384000 0.610939000

C 4.379140000 -1.019751000 -0.438150000

H 4.025763000 -1.888894000 -1.004803000

H 4.966738000 -0.391369000 -1.117133000

H 1.938434000 -0.657040000 -1.547248000

H 5.050971000 -1.383070000 0.347308000

H 1.329171000 2.138627000 -0.413879000

Cl -4.583818000 -0.547430000 0.272068000

===============================

**I-Cl-cat**

===============================

C -2.892536000 -0.071791000 0.018778000

C -2.480118000 1.266239000 0.188594000

C -1.992656000 -1.081361000 -0.393355000

H -3.197419000 2.014200000 0.505206000

H -2.349723000 -2.097446000 -0.513689000

C -1.164784000 1.590415000 -0.052357000

C -0.680972000 -0.752439000 -0.638947000

H -0.830040000 2.615464000 0.076886000

H 0.009949000 -1.523491000 -0.957707000

C -0.222048000 0.594831000 -0.471108000

C 1.102671000 0.973120000 -0.681693000

C 2.239950000 0.133438000 -1.050442000

H 2.870203000 0.692322000 -1.752043000

C 3.177916000 -0.211256000 0.209660000

C 2.401836000 -1.095572000 1.195450000

C 3.653443000 1.080309000 0.893074000

H 4.136373000 1.758446000 0.181701000

H 2.834589000 1.616726000 1.384436000

H 4.387990000 0.836622000 1.665823000

H 1.533068000 -0.581001000 1.618155000

H 3.053838000 -1.376571000 2.027762000

H 2.059385000 -2.022419000 0.722321000

C 4.379952000 -0.981718000 -0.360125000

H 4.067172000 -1.894799000 -0.876428000

H 4.956325000 -0.368642000 -1.059718000

H 1.942427000 -0.809006000 -1.513640000

H 5.046294000 -1.272752000 0.458054000

H 1.330872000 2.023559000 -0.501869000

Cl -4.528660000 -0.486853000 0.317034000

===============================

**I-CF_3_-rad**

===============================

C -2.263879000 0.065139000 -0.022946000

C -1.828447000 1.384106000 0.176960000

C -1.346974000 -0.917263000 -0.423669000

H -2.539934000 2.138828000 0.494280000

H -1.688724000 -1.936602000 -0.568214000

C -0.500071000 1.712166000 -0.018028000

C -0.015437000 -0.594957000 -0.619708000

H -0.168898000 2.734185000 0.144048000

H 0.677664000 -1.372945000 -0.917533000

C 0.460250000 0.736308000 -0.424532000

C 1.809411000 1.115606000 -0.607046000

C 2.945770000 0.229731000 -1.003534000

H 3.593999000 0.785048000 -1.695555000

C 3.846382000 -0.275312000 0.174870000

C 3.028871000 -1.175629000 1.116414000

C 4.409383000 0.920968000 0.961735000

H 4.963843000 1.601699000 0.305231000

H 3.610288000 1.491869000 1.445093000

H 5.095364000 0.578888000 1.744249000

H 2.173410000 -0.639406000 1.537629000

H 3.650268000 -1.529856000 1.946239000

H 2.646260000 -2.056326000 0.588262000

C 5.007787000 -1.084259000 -0.428445000

H 4.636489000 -1.937665000 -1.007353000

H 5.619150000 -0.465380000 -1.095001000

H 2.586334000 -0.646775000 -1.555387000

H 5.661113000 -1.473746000 0.359954000

H 2.051148000 2.157081000 -0.406091000

C -3.716407000 -0.275366000 0.126738000

F -3.898630000 -1.565196000 0.494331000

F -4.323715000 0.502933000 1.052984000

F -4.398499000 -0.101773000 -1.033170000

===============================

**I-CF_3_-cat**

===============================

C -2.227879000 0.068610000 -0.035204000

C -1.777695000 1.381330000 0.168467000

C -1.361458000 -0.952133000 -0.467261000

H -2.470793000 2.140789000 0.510446000

H -1.746025000 -1.956043000 -0.604768000

C -0.449029000 1.679767000 -0.063330000

C -0.034567000 -0.662509000 -0.706401000

H -0.081713000 2.689695000 0.092927000

H 0.635079000 -1.444287000 -1.043528000

C 0.457139000 0.664940000 -0.504249000

C 1.800163000 1.011469000 -0.699465000

C 2.913967000 0.150644000 -1.062934000

H 3.590455000 0.706164000 -1.721587000

C 3.804322000 -0.254536000 0.230868000

C 2.972631000 -1.151014000 1.156213000

C 4.280244000 1.006082000 0.967717000

H 4.793793000 1.699405000 0.293602000

H 3.456693000 1.538378000 1.455536000

H 4.989926000 0.723753000 1.750507000

H 2.102430000 -0.630132000 1.567243000

H 3.590196000 -1.473809000 1.999711000

H 2.626854000 -2.053845000 0.641526000

C 5.006146000 -1.032030000 -0.328349000

H 4.691510000 -1.919954000 -0.885311000

H 5.620252000 -0.408878000 -0.985354000

H 2.605880000 -0.775009000 -1.551019000

H 5.635916000 -1.365101000 0.502887000

H 2.053251000 2.052728000 -0.499026000

C -3.702631000 -0.252671000 0.159148000

F -3.861133000 -1.513056000 0.591997000

F -4.264427000 0.582745000 1.045143000

F -4.339086000 -0.122083000 -1.016599000

===============================

**I-CHO-rad**

===============================

C -2.974890000 -0.277847000 -0.017674000

C -2.623208000 1.084648000 0.105625000

C -1.980145000 -1.214345000 -0.360184000

H -3.402708000 1.791992000 0.370113000

H -2.249588000 -2.264083000 -0.454068000

C -1.325206000 1.489743000 -0.105517000

C -0.674573000 -0.818162000 -0.574726000

H -1.060941000 2.539521000 -0.008963000

H 0.073375000 -1.559461000 -0.831074000

C -0.294405000 0.555523000 -0.454012000

C 1.019926000 1.015069000 -0.657109000

C 2.216931000 0.192642000 -1.007446000

H 2.818314000 0.749031000 -1.739740000

C 3.156455000 -0.162492000 0.194692000

C 2.407571000 -1.043482000 1.209087000

C 3.644010000 1.123502000 0.885416000

H 4.152913000 1.785751000 0.175427000

H 2.813210000 1.679176000 1.331827000

H 4.352017000 0.884711000 1.686270000

H 1.522583000 -0.535316000 1.603194000

H 3.058339000 -1.295642000 2.053410000

H 2.078347000 -1.982787000 0.750576000

C 4.366344000 -0.934343000 -0.359885000

H 4.051591000 -1.853468000 -0.867017000

H 4.929327000 -0.328316000 -1.078744000

H 1.922574000 -0.742637000 -1.496725000

H 5.050290000 -1.216177000 0.448225000

H 1.189242000 2.080391000 -0.515289000

C -4.360306000 -0.711069000 0.210080000

O -5.279872000 0.034476000 0.509121000

H -4.530931000 -1.804824000 0.089529000

===============================

**I-CHO-cat**

===============================

C -2.943678000 -0.274634000 -0.009831000

C -2.577159000 1.081832000 0.070706000

C -2.001935000 -1.267670000 -0.343813000

H -3.337512000 1.810413000 0.331055000

H -2.313070000 -2.306661000 -0.400770000

C -1.271120000 1.445593000 -0.181220000

C -0.692861000 -0.916057000 -0.603281000

H -0.970356000 2.487586000 -0.121827000

H 0.030988000 -1.677862000 -0.866566000

C -0.293999000 0.455085000 -0.523231000

C 1.022360000 0.871600000 -0.758022000

C 2.190952000 0.058105000 -1.053825000

H 2.806800000 0.582321000 -1.793656000

C 3.141067000 -0.125458000 0.245100000

C 2.390511000 -0.937758000 1.308289000

C 3.564578000 1.244287000 0.795160000

H 4.025343000 1.866131000 0.020586000

H 2.725458000 1.795870000 1.232136000

H 4.305097000 1.105042000 1.587988000

H 1.515633000 -0.405732000 1.694962000

H 3.056381000 -1.133759000 2.153881000

H 2.062829000 -1.907667000 0.918588000

C 4.369464000 -0.902909000 -0.252656000

H 4.091122000 -1.873160000 -0.675551000

H 4.924020000 -0.340312000 -1.009703000

H 1.942893000 -0.936885000 -1.426226000

H 5.044045000 -1.087543000 0.589473000

H 1.204590000 1.941664000 -0.657859000

C -4.373343000 -0.656190000 0.267025000

O -5.206820000 0.170093000 0.559279000

H -4.608719000 -1.735144000 0.186854000

===============================

**I-CN-rad**

===============================

C -3.068480000 -0.099346000 0.018860000

C -2.663457000 1.241168000 0.210614000

C -2.114308000 -1.058443000 -0.387764000

H -3.394989000 1.978846000 0.523247000

H -2.426336000 -2.087345000 -0.533652000

C -1.349595000 1.603511000 0.002446000

C -0.799373000 -0.694223000 -0.595377000

H -1.048214000 2.636259000 0.153291000

H -0.086096000 -1.450742000 -0.900485000

C -0.361531000 0.653658000 -0.407471000

C 0.969735000 1.071196000 -0.601809000

C 2.130552000 0.220335000 -1.002329000

H 2.752175000 0.791112000 -1.705848000

C 3.058312000 -0.239727000 0.173707000

C 2.279783000 -1.156261000 1.132562000

C 3.588288000 0.983611000 0.941850000

H 4.113492000 1.675136000 0.272822000

H 2.776243000 1.533176000 1.428463000

H 4.293014000 0.672674000 1.720521000

H 1.410894000 -0.644810000 1.557282000

H 2.920258000 -1.480331000 1.960024000

H 1.922195000 -2.055236000 0.617826000

C 4.240544000 -1.016285000 -0.431727000

H 3.893387000 -1.888940000 -0.996601000

H 4.824509000 -0.385228000 -1.111172000

H 1.796620000 -0.673747000 -1.541252000

H 4.913755000 -1.373334000 0.355211000

H 1.181761000 2.120280000 -0.406762000

C -4.426365000 -0.480467000 0.234616000

N -5.535328000 -0.791449000 0.411144000

===============================

**I-CN-cat**

===============================

C -3.026324000 0.082415000 -0.023018000

C -2.606295000 -1.253664000 -0.207044000

C -2.123733000 1.083543000 0.410994000

H -3.318093000 -2.000829000 -0.538593000

H -2.477814000 2.099172000 0.545622000

C -1.289586000 -1.582937000 0.037999000

C -0.810560000 0.751970000 0.661192000

H -0.951990000 -2.605305000 -0.103201000

H -0.121429000 1.516756000 0.997838000

C -0.356088000 -0.591047000 0.474850000

C 0.974864000 -0.974398000 0.686749000

C 2.107495000 -0.142283000 1.057895000

H 2.757704000 -0.710048000 1.732635000

C 3.031206000 0.222707000 -0.224959000

C 2.237109000 1.121745000 -1.180564000

C 3.493536000 -1.060125000 -0.931322000

H 3.981889000 -1.751074000 -0.236429000

H 2.668089000 -1.584979000 -1.423911000

H 4.221584000 -0.805617000 -1.706780000

H 1.365385000 0.612241000 -1.602735000

H 2.878380000 1.419985000 -2.015355000

H 1.899056000 2.039015000 -0.686571000

C 4.238595000 0.983222000 0.345180000

H 3.933245000 1.888175000 0.879421000

H 4.824997000 0.358648000 1.025656000

H 1.819712000 0.796878000 1.532414000

H 4.892579000 1.286867000 -0.478625000

H 1.200670000 -2.023573000 0.495789000

C -4.387483000 0.431792000 -0.277334000

N -5.495770000 0.718007000 -0.485840000

===============================

Pd(PPh_3_)_3_Cl

===============================

Pd 0.016438000 0.417335000 -0.468439000

P -0.005474000 -1.976059000 -0.155861000

P -2.334022000 1.039333000 -0.029164000

P 2.334761000 0.997331000 0.051866000

C -2.452797000 2.798400000 0.507583000

C -3.691422000 3.444276000 0.646055000

C -1.278576000 3.490687000 0.828736000

C -3.750188000 4.758203000 1.107603000

H -4.607661000 2.922944000 0.385931000

C -1.339423000 4.804955000 1.296800000

H -0.316722000 3.009630000 0.696046000

C -2.573972000 5.438997000 1.437471000

H -4.712760000 5.252116000 1.207859000

H -0.416917000 5.324361000 1.537261000

H -2.622854000 6.463550000 1.796187000

C -3.085019000 0.138047000 1.392131000

C -3.617255000 -1.151242000 1.212607000

C -2.985536000 0.649159000 2.697013000

C -4.036464000 -1.905775000 2.307018000

H -3.706743000 -1.568782000 0.217038000

C -3.405682000 -0.110809000 3.790765000

H -2.579687000 1.642830000 2.858460000

C -3.930802000 -1.390912000 3.600732000

H -4.436375000 -2.902207000 2.143181000

H -3.325568000 0.302712000 4.792464000

H -4.253140000 -1.982021000 4.453044000

C -3.601565000 0.902687000 -1.363589000

C -4.952284000 0.609598000 -1.118057000

C -3.176746000 1.108178000 -2.686631000

C -5.856738000 0.502438000 -2.175847000

H -5.300862000 0.448106000 -0.103436000

C -4.086937000 1.006599000 -3.739532000

H -2.135824000 1.351356000 -2.883945000

C -5.426180000 0.696635000 -3.489775000

H -6.897689000 0.267818000 -1.970868000

H -3.744218000 1.169100000 -4.757612000

H -6.130910000 0.611489000 -4.312449000

C 2.495218000 2.689473000 0.766972000

C 2.172205000 3.788868000 -0.049433000

C 2.861820000 2.914542000 2.102625000

C 2.228976000 5.083574000 0.464737000

H 1.851378000 3.621179000 -1.072772000

C 2.905432000 4.213624000 2.613293000

H 3.120347000 2.080573000 2.746388000

C 2.593234000 5.301692000 1.796889000

H 1.977291000 5.922659000 -0.177625000

H 3.192390000 4.372843000 3.649122000

H 2.633132000 6.311990000 2.194735000

C 3.475884000 1.015576000 -1.382367000

C 3.191096000 0.166232000 -2.459455000

C 4.633550000 1.807955000 -1.415641000

C 4.064402000 0.091039000 -3.543739000

H 2.283052000 -0.424700000 -2.451349000

C 5.500891000 1.736876000 -2.506330000

H 4.851618000 2.483238000 -0.593732000

C 5.219158000 0.874938000 -3.569453000

H 3.834210000 -0.575560000 -4.369306000

H 6.393590000 2.355803000 -2.527225000

H 5.894852000 0.822096000 -4.418747000

C 3.189399000 -0.069637000 1.286119000

C 4.514085000 -0.511066000 1.151344000

C 2.427410000 -0.531805000 2.370048000

C 5.052012000 -1.412465000 2.071621000

H 5.115508000 -0.172421000 0.314867000

C 2.964415000 -1.431708000 3.290130000

H 1.396008000 -0.210540000 2.469643000

C 4.277974000 -1.880936000 3.136762000

H 6.075596000 -1.756495000 1.950639000

H 2.343989000 -1.804190000 4.099272000

H 4.694779000 -2.596361000 3.839997000

C 0.059840000 -2.768200000 1.503641000

C -0.525402000 -2.075284000 2.573073000

C 0.630908000 -4.028492000 1.740557000

C -0.536134000 -2.625378000 3.854473000

H -0.969293000 -1.100884000 2.403639000

C 0.630807000 -4.572445000 3.025814000

H 1.086488000 -4.579760000 0.924526000

C 0.048202000 -3.872601000 4.085551000

H -1.004470000 -2.075648000 4.664963000

H 1.083660000 -5.544793000 3.198093000

H 0.048280000 -4.299286000 5.084685000

C -1.512030000 -2.718904000 -0.917808000

C -2.086564000 -2.052287000 -2.012000000

C -2.154544000 -3.852369000 -0.397548000

C -3.288080000 -2.496281000 -2.565239000

H -1.614056000 -1.156884000 -2.404014000

C -3.356038000 -4.295385000 -0.951938000

H -1.734965000 -4.372365000 0.456668000

C -3.929068000 -3.615234000 -2.030292000

H -3.734685000 -1.940508000 -3.383652000

H -3.851341000 -5.167097000 -0.533084000

H -4.875078000 -3.951022000 -2.445301000

C 1.388395000 -2.728558000 -1.093863000

C 1.255256000 -3.018463000 -2.461716000

C 2.654255000 -2.851872000 -0.496182000

C 2.363171000 -3.414684000 -3.211578000

H 0.287885000 -2.931499000 -2.945337000

C 3.757746000 -3.248537000 -1.250049000

H 2.783712000 -2.635756000 0.557161000

C 3.619099000 -3.526441000 -2.610775000

H 2.242479000 -3.634661000 -4.268579000

H 4.728273000 -3.325548000 -0.768911000

H 4.481694000 -3.825154000 -3.199058000

Cl 0.155891000 2.143435000 -2.380310000

===============================

Pd(PPh_3_)_3_Cl^–^

===============================

Pd -0.052523000 0.108771000 -0.698780000

P 1.829498000 1.437011000 -0.170234000

P 0.321436000 -2.124354000 -0.066404000

P -2.174095000 0.837106000 -0.105743000

C -1.208056000 -3.175989000 0.047035000

C -1.454180000 -4.166088000 1.008394000

C -2.188335000 -2.924747000 -0.925885000

C -2.670651000 -4.852890000 1.023650000

H -0.704999000 -4.386449000 1.762693000

C -3.396892000 -3.621420000 -0.924203000

H -2.008699000 -2.149277000 -1.663128000

C -3.647550000 -4.579307000 0.062308000

H -2.855662000 -5.603115000 1.789161000

H -4.148078000 -3.387528000 -1.673848000

H -4.596931000 -5.108795000 0.083231000

C 1.043098000 -2.174186000 1.628729000

C 2.437396000 -2.083030000 1.798167000

C 0.224349000 -2.033286000 2.764776000

C 2.990144000 -1.884567000 3.063008000

H 3.094816000 -2.141977000 0.936751000

C 0.782286000 -1.842954000 4.028566000

H -0.854998000 -2.044075000 2.663663000

C 2.168278000 -1.770560000 4.186579000

H 4.069303000 -1.801297000 3.161329000

H 0.122489000 -1.730313000 4.885228000

H 2.601872000 -1.604984000 5.169351000

C 1.399477000 -3.288087000 -1.031311000

C 2.080829000 -4.380693000 -0.473715000

C 1.500809000 -3.050153000 -2.411091000

C 2.866634000 -5.209719000 -1.274916000

H 2.009155000 -4.574199000 0.592716000

C 2.275967000 -3.892439000 -3.212221000

H 0.997129000 -2.188623000 -2.849873000

C 2.965646000 -4.967906000 -2.648213000

H 3.399050000 -6.046469000 -0.828314000

H 2.349106000 -3.693084000 -4.278343000

H 3.578586000 -5.614264000 -3.272210000

C -3.618602000 0.326182000 -1.158355000

C -3.326963000 0.001444000 -2.493248000

C -4.945525000 0.243063000 -0.709700000

C -4.347449000 -0.410037000 -3.354400000

H -2.296772000 0.055394000 -2.848117000

C -5.960291000 -0.180405000 -1.569145000

H -5.184282000 0.487914000 0.321194000

C -5.662705000 -0.509921000 -2.894669000

H -4.105787000 -0.662336000 -4.383549000

H -6.982497000 -0.253545000 -1.204836000

H -6.453093000 -0.842297000 -3.563668000

C -2.478158000 2.668997000 0.001336000

C -1.608454000 3.497058000 -0.721493000

C -3.525988000 3.256116000 0.728144000

C -1.781854000 4.883371000 -0.720803000

H -0.791415000 3.045378000 -1.277238000

C -3.694101000 4.640884000 0.737373000

H -4.202855000 2.628990000 1.301493000

C -2.822262000 5.458064000 0.010614000

H -1.089481000 5.503796000 -1.282483000

H -4.504987000 5.083593000 1.311385000

H -2.953472000 6.537553000 0.020167000

C -2.660650000 0.265152000 1.585675000

C -2.214728000 0.975658000 2.715493000

C -3.284971000 -0.978411000 1.791593000

C -2.386313000 0.462977000 4.000770000

H -1.715283000 1.930539000 2.585802000

C -3.460018000 -1.488616000 3.079109000

H -3.618686000 -1.563649000 0.943013000

C -3.010769000 -0.773064000 4.191772000

H -2.026909000 1.031677000 4.855028000

H -3.933366000 -2.458992000 3.203217000

H -3.140246000 -1.174636000 5.193508000

C 2.006540000 2.033220000 1.574834000

C 1.208361000 1.396970000 2.535570000

C 2.902275000 3.029971000 2.000628000

C 1.298380000 1.743167000 3.884887000

H 0.519501000 0.621120000 2.218079000

C 2.985481000 3.385181000 3.347769000

H 3.532371000 3.533220000 1.273175000

C 2.182914000 2.741336000 4.294598000

H 0.676054000 1.222122000 4.606528000

H 3.679405000 4.162421000 3.659183000

H 2.250131000 3.018049000 5.344047000

C 3.404570000 0.471922000 -0.403906000

C 3.433577000 -0.419120000 -1.492445000

C 4.473738000 0.457174000 0.504494000

C 4.502874000 -1.298285000 -1.663638000

H 2.589375000 -0.449493000 -2.178106000

C 5.539431000 -0.430830000 0.335697000

H 4.465823000 1.113488000 1.367563000

C 5.556401000 -1.313895000 -0.745694000

H 4.484297000 -2.001462000 -2.491031000

H 6.350131000 -0.439834000 1.060978000

H 6.375956000 -2.018435000 -0.864664000

C 2.103903000 2.956421000 -1.187516000

C 2.479097000 2.803100000 -2.534929000

C 1.748989000 4.240526000 -0.737992000

C 2.543412000 3.905571000 -3.384845000

H 2.676389000 1.814606000 -2.932571000

C 1.814342000 5.342708000 -1.593157000

H 1.404286000 4.384122000 0.280456000

C 2.219754000 5.182766000 -2.919169000

H 2.830692000 3.760002000 -4.422801000

H 1.542298000 6.326857000 -1.218126000

H 2.267578000 6.039750000 -3.586347000

Cl 0.256781000 0.139899000 -3.468209000

===============================

Pd(PPh_3_)_2_Cl_2_

===============================

Pd 0.000021000 0.000101000 -0.000004000

P 2.387684000 -0.035531000 -0.029434000

C 3.058712000 0.072699000 -1.736860000

C 2.310017000 -0.446476000 -2.804910000

C 4.320829000 0.636135000 -1.989916000

C 2.821515000 -0.403803000 -4.102385000

H 1.341523000 -0.896237000 -2.615885000

C 4.825530000 0.674617000 -3.289942000

H 4.904102000 1.051151000 -1.174686000

C 4.076727000 0.155766000 -4.348277000

H 2.234573000 -0.809114000 -4.921415000

H 5.802245000 1.112752000 -3.474406000

H 4.469831000 0.189799000 -5.360435000

C 3.252541000 1.305968000 0.873230000

C 3.989011000 1.054519000 2.038991000

C 3.149952000 2.623034000 0.394473000

C 4.619680000 2.103886000 2.710745000

H 4.078167000 0.043273000 2.420469000

C 3.790221000 3.663815000 1.062385000

H 2.564840000 2.831371000 -0.494063000

C 4.524641000 3.407384000 2.223240000

H 5.189844000 1.897512000 3.611978000

H 3.704849000 4.677600000 0.682687000

H 5.018184000 4.221807000 2.745955000

C 3.084347000 -1.568763000 0.693606000

C 4.299987000 -2.107939000 0.253293000

C 2.403668000 -2.174675000 1.759462000

C 4.830323000 -3.237911000 0.877394000

H 4.828465000 -1.654156000 -0.578774000

C 2.942580000 -3.296558000 2.387419000

H 1.445732000 -1.777671000 2.079981000

C 4.155620000 -3.830588000 1.946317000

H 5.769414000 -3.655628000 0.526016000

H 2.407788000 -3.761606000 3.210291000

H 4.569891000 -4.710752000 2.429766000

Cl -0.052359000 2.257511000 0.745710000

P -2.387589000 0.035608000 0.029558000

C -3.084341000 1.568390000 -0.694366000

C -4.300690000 2.106944000 -0.255191000

C -2.403110000 2.174556000 -1.759713000

C -4.831136000 3.236518000 -0.879887000

H -4.829623000 1.653012000 0.576507000

C -2.942110000 3.296079000 -2.388259000

H -1.444696000 1.778040000 -2.079391000

C -4.155829000 3.829471000 -1.948289000

H -5.770782000 3.653726000 -0.529385000

H -2.406850000 3.761324000 -3.210715000

H -4.570183000 4.709342000 -2.432200000

C -3.252333000 -1.306448000 -0.872397000

C -3.149636000 -2.623252000 -0.392945000

C -3.988745000 -1.055689000 -2.038329000

C -3.789812000 -3.664439000 -1.060304000

H -2.564499000 -2.831087000 0.495699000

C -4.619336000 -2.105463000 -2.709533000

H -4.077917000 -0.044662000 -2.420391000

C -4.524239000 -3.408684000 -2.221309000

H -3.704361000 -4.678015000 -0.680067000

H -5.189467000 -1.899616000 -3.610906000

H -5.017723000 -4.223423000 -2.743587000

C -3.058838000 -0.071824000 1.736962000

C -2.310218000 0.447658000 2.804909000

C -4.321026000 -0.635083000 1.990081000

C -2.821876000 0.405511000 4.102346000

H -1.341647000 0.897228000 2.615842000

C -4.825889000 -0.673021000 3.290054000

H -4.904203000 -1.050422000 1.174948000

C -4.077170000 -0.153838000 4.348289000

H -2.234986000 0.811057000 4.921296000

H -5.802661000 -1.111007000 3.474573000

H -4.470411000 -0.187461000 5.360408000

Cl 0.052404000 -2.257212000 -0.745950000

===============================

Pd(PPh_3_)_2_Cl_2_^–^

===============================

Pd -0.019901000 -0.052642000 -1.495818000

P -2.091515000 -0.107720000 -0.229453000

C -3.710654000 0.390277000 -0.979722000

C -3.663040000 1.259194000 -2.083802000

C -4.957812000 -0.065872000 -0.525582000

C -4.844346000 1.681098000 -2.697064000

H -2.698816000 1.611224000 -2.445040000

C -6.134422000 0.347001000 -1.153185000

H -5.013371000 -0.751361000 0.313874000

C -6.081877000 1.226066000 -2.237150000

H -4.791735000 2.360278000 -3.544035000

H -7.093180000 -0.019341000 -0.793548000

H -6.999484000 1.547867000 -2.723861000

C -2.522937000 -1.623645000 0.734272000

C -3.166057000 -1.601313000 1.983726000

C -2.166118000 -2.860726000 0.175450000

C -3.440229000 -2.788654000 2.663012000

H -3.435486000 -0.650698000 2.433508000

C -2.448210000 -4.047903000 0.855871000

H -1.630017000 -2.889084000 -0.770629000

C -3.081508000 -4.016543000 2.099836000

H -3.931318000 -2.754762000 3.632524000

H -2.156035000 -4.996775000 0.413855000

H -3.290816000 -4.941594000 2.631758000

C -1.834275000 1.166461000 1.086812000

C -2.174628000 2.509158000 0.858912000

C -1.110330000 0.843019000 2.247945000

C -1.819869000 3.495804000 1.779785000

H -2.690929000 2.789307000 -0.051713000

C -0.760373000 1.831781000 3.166770000

H -0.802080000 -0.179967000 2.431596000

C -1.114943000 3.162949000 2.938804000

H -2.085851000 4.530756000 1.581023000

H -0.188771000 1.555305000 4.048211000

H -0.831624000 3.935288000 3.649299000

Cl 0.335997000 -2.480749000 -2.245988000

P 2.074772000 0.031425000 -0.295459000

C 3.558359000 -0.866129000 -0.920980000

C 4.600045000 -1.307295000 -0.089714000

C 3.618694000 -1.135457000 -2.295143000

C 5.700036000 -1.975443000 -0.628490000

H 4.543323000 -1.133051000 0.981410000

C 4.720734000 -1.803282000 -2.832889000

H 2.781352000 -0.852030000 -2.925045000

C 5.763616000 -2.219357000 -2.003432000

H 6.502857000 -2.311212000 0.023524000

H 4.753844000 -2.013377000 -3.898475000

H 6.617707000 -2.745423000 -2.423137000

C 2.708759000 1.676375000 0.240944000

C 1.737490000 2.621547000 0.613948000

C 4.064342000 2.028738000 0.312842000

C 2.119761000 3.876682000 1.083441000

H 0.686043000 2.379874000 0.517003000

C 4.442477000 3.294961000 0.766464000

H 4.827709000 1.316524000 0.016574000

C 3.472116000 4.218499000 1.160839000

H 1.351570000 4.587325000 1.374133000

H 5.497010000 3.556488000 0.813926000

H 3.768539000 5.202338000 1.516926000

C 1.784689000 -0.805784000 1.334855000

C 1.063878000 -2.013808000 1.321144000

C 2.155928000 -0.251085000 2.568013000

C 0.701848000 -2.633684000 2.516345000

H 0.760509000 -2.442209000 0.369451000

C 1.796747000 -0.878926000 3.764141000

H 2.699088000 0.687842000 2.596669000

C 1.061116000 -2.065319000 3.742654000

H 0.107703000 -3.542506000 2.484574000

H 2.081415000 -0.430025000 4.712993000

H 0.761717000 -2.539802000 4.673886000

Cl -0.287073000 2.403247000 -2.129187000

===============================

**H-TS/D-TS**

===============================

C 5.293209000 -1.221293000 -0.060471000

C 4.934386000 -0.822142000 1.236324000

C 4.652551000 -0.640505000 -1.163114000

H 5.430783000 -1.292820000 2.078240000

H 4.906131000 -0.931545000 -2.175336000

C 3.954641000 0.137840000 1.424233000

C 3.671508000 0.329119000 -0.958808000

H 3.650933000 0.405186000 2.430677000

H 3.186041000 0.756880000 -1.830795000

C 3.302950000 0.744417000 0.330025000

C 2.257942000 1.746942000 0.596348000

C 1.567985000 2.482765000 -0.392952000

H 0.247805000 1.589246000 -0.841929000

C 0.934189000 3.858827000 -0.153440000

C 2.086365000 4.888382000 -0.235492000

C 0.239547000 3.968883000 1.213658000

H -0.564283000 3.230769000 1.303051000

H 0.933929000 3.810600000 2.043716000

H -0.193759000 4.967787000 1.330235000

H 2.827326000 4.710842000 0.550233000

H 1.695778000 5.904692000 -0.116255000

H 2.599313000 4.836952000 -1.202388000

C -0.088654000 4.151225000 -1.267901000

H 0.371302000 4.067327000 -2.259651000

H -0.929188000 3.450940000 -1.224847000

H 1.920075000 2.363425000 -1.417776000

H -0.484516000 5.167071000 -1.168151000

H 2.206010000 2.098464000 1.622894000

O 6.267154000 -2.172703000 -0.140112000

C 6.648295000 -2.638563000 -1.424678000

H 5.803780000 -3.095735000 -1.957657000

H 7.065440000 -1.831862000 -2.042672000

H 7.417508000 -3.394157000 -1.256492000

P -1.657122000 -0.438297000 -0.077241000

Pd 0.330214000 0.663428000 0.438314000

Cl 0.713788000 -0.501905000 2.546061000

C -2.679604000 0.364144000 -1.385236000

C -1.338164000 -2.125620000 -0.730603000

C -2.810699000 -0.699567000 1.321884000

C -2.031002000 0.805093000 -2.551017000

C -4.062604000 0.555493000 -1.265788000

C -2.750276000 1.412552000 -3.577603000

H -0.956816000 0.673922000 -2.648442000

C -4.780364000 1.177697000 -2.291416000

H -4.582623000 0.224864000 -0.373335000

C -4.129316000 1.604240000 -3.448322000

H -2.234730000 1.744354000 -4.474329000

H -5.850660000 1.327260000 -2.181374000

H -4.689295000 2.087722000 -4.243591000

C -2.059860000 -2.670916000 -1.802447000

C -0.339292000 -2.889118000 -0.102680000

C -1.786392000 -3.967918000 -2.241945000

H -2.829303000 -2.085240000 -2.295851000

C -0.076468000 -4.185048000 -0.545585000

H 0.220422000 -2.466600000 0.728072000

C -0.795794000 -4.725668000 -1.614767000

H -2.347395000 -4.383736000 -3.074251000

H 0.696037000 -4.770645000 -0.055630000

H -0.583417000 -5.733861000 -1.959512000

C -2.725523000 0.142529000 2.438748000

C -3.783728000 -1.710940000 1.288896000

C -3.620372000 -0.009681000 3.498483000

H -1.937457000 0.885833000 2.490096000

C -4.674330000 -1.860655000 2.351623000

H -3.839784000 -2.383547000 0.437954000

C -4.595757000 -1.007306000 3.455370000

H -3.542494000 0.640702000 4.364614000

H -5.423486000 -2.646680000 2.321340000

H -5.286249000 -1.128898000 4.285216000
